# Marine Natural Products from Indonesian Waters

**DOI:** 10.3390/md17060364

**Published:** 2019-06-19

**Authors:** Novriyandi Hanif, Anggia Murni, Chiaki Tanaka, Junichi Tanaka

**Affiliations:** 1 Department of Chemistry, Faculty of Mathematics and Natural Sciences, IPB University (Bogor Agricultural University), Bogor 16680, Indonesia; 2 Tropical Biopharmaca Research Center, IPB University (Bogor Agricultural University), Bogor 16128, Indonesia; anggia_murni@apps.ipb.ac.id; 3 Department of Natural Products Chemistry, Graduate School of Pharmaceutical Sciences, Kyushu University, Fukuoka 812–8582, Japan; ctanaka@phar.kyushu-u.ac.jp; 4 Department of Chemistry, Biology, and Marine Science, University of the Ryukyus, Nishihara, Okinawa 903-0213, Japan; jtanaka@sci.u-ryukyu.ac.jp

**Keywords:** biodiversity, structure elucidation, chemical synthesis, biosynthesis, structural revision, cytotoxicity, enzyme inhibitor, bioorganic chemistry, biogeography

## Abstract

Natural products are primal and have been a driver in the evolution of organic chemistry and ultimately in science. The chemical structures obtained from marine organisms are diverse, reflecting biodiversity of genes, species and ecosystems. Biodiversity is an extraordinary feature of life and provides benefits to humanity while promoting the importance of environment conservation. This review covers the literature on marine natural products (MNPs) discovered in Indonesian waters published from January 1970 to December 2017, and includes 732 original MNPs, 4 structures isolated for the first time but known to be synthetic entities, 34 structural revisions, 9 artifacts, and 4 proposed MNPs. Indonesian MNPs were found in 270 papers from 94 species, 106 genera, 64 families, 32 orders, 14 classes, 10 phyla, and 5 kingdoms. The emphasis is placed on the structures of organic molecules (original and revised), relevant biological activities, structure elucidation, chemical ecology aspects, biosynthesis, and bioorganic studies. Through the synthesis of past and future data, huge and partly undescribed biodiversity of marine tropical invertebrates and their importance for crucial societal benefits should greatly be appreciated.

## 1. Introduction

Natural products have been the core of the evolution of organic chemistry. About 40 molecules have been detected that have had revolutionary effects and become indispensable for human society [1]. In particular, three MNPs, (+)-palytoxin, (+)-brevetoxin B, and (–)-ecteinascidin 743, are engines for its development. The beautiful and challenging structures of MNPs have been disclosed by several methods, including classic and modern structure elucidation, even though they are often available only in invisibly small amounts and have unprecedented properties in nature. This is becoming the predominant task of organic chemistry [2].

MNPs are defined as secondary metabolites produced by marine organisms, both macro- and microorganisms, as responses that are part of their defense strategies, responses to the food chain, or as communication signals with their environment. Moreover, these functions are related to biodiversity, which is considered an important key for obtaining diverse metabolites, the structures of which are responsible for the characteristics of a vast range of chemical reactions, both in living and non-living systems.

Biodiversity generally refers to the number of species living in a particular ecosystem. The accepted number of species on land is 0.45–1.9 million [3,4], while the species richness in the oceans is estimated to be 0.30–10 million [5]. Some of the highest concentrations of species are located in the tropical region [6]. Due to high diversity of species, and hence the high competition for survival, MNPs exhibit chemical structures and biological activities that are different from those of traditionally investigated terrestrial natural products. The uniqueness of skeletons, functional groups (FGs) and remote chiral centers are some features of MNPs that are presented in this review.

To date, eight marine-derived natural products [7,8,9,10] have been recorded as approved drugs, of which seven molecules have been used to date. (–)-Ecteinascidin 743 (Yondelis^®^) was derived directly from the Caribbean ascidian *Ecteinascidia turbinata* (the true producer was recently established as γ-proteobacterial endosymbiont *Candidatus endoecteinascidia frumentensis* [11]). The molecule is used for treatment of advanced soft tissue sarcoma and for treatment of recurrent platinum-sensitive ovarian cancer when combined with liposomal doxorubicin. The peptide ω-conotoxin MVIIa (Prialt^®^) from the venom of the cone snail *Conus magus* is used for analgesic treatment. The anticancer agent (–)-eribulin mesylate (Halaven^®^) is a synthetic truncated derivative of the polyketide halichondrin B, a super-carbon chain compound isolated from the Japanese sponge *Halichondria okadai*. The last approved anticancer drug related to MNPs is the antibody−drug conjugate (ADC) brentuximab vedotin (Adcetris^®^). It consists of a tumor-specific antibody and the pentapeptide auristatin E, a derivative of dolastatin 10 derived from the Indian sea hare *Dolabella auricularia*. The molecule is used for the treatment of primary cutaneous anaplastic large cell lymphoma (pcALCL) or CD30-expressing mycosis fungoides (MF) in those who have received prior systemic therapy. In addition, the ADC is used to treat untreated stage III or IV classical Hodgkin lymphoma (cHL) in combination with chemotherapy. A mixture of two ethyl esters of fish-derived ω-3 polyunsaturated fatty acids, docosahexaenoic acid (DHA) and eicosapentanaenoic acid (EPA) was approved under the trade name Lovaza^®^ and is used for reducing serum triglycerides. *Iota*-carrageenan (Carragelose^®^), isolated from red algae *Eucheuma*/ *Cnondrus*, works as an anti-viral barrier in the nasal cavity. Two nucleosides, the anticancer agent (+)-cytarabine (Cytosar^®^) and the antiviral drug against herpes simplex virus (–)-vidarabine (Vira-A^®^) are derivatives of spongothymidine and spongouridine from the Caribbean sponge *Tethya crypta*, respectively. (–)-Vidarabine (Vira-A^®^) has been discontinued in the US and in Europe.

The structural diversities of natural products affording unique pharmacological or physiological activities and intricate structures have contributed to breakthroughs in basic (i.e., organic) chemistry and applied sciences (e.g., marine biotechnology), and have even led to a few Nobel Prizes [12]. Ultimately, the diversity of chemicals and biological activities can provide excellent tools for other scientific fields, including biology, agriculture, medicine, materials, energy, and the environment. These facts have made marine organisms attractive targets for research endeavors.

**Figure 1:**
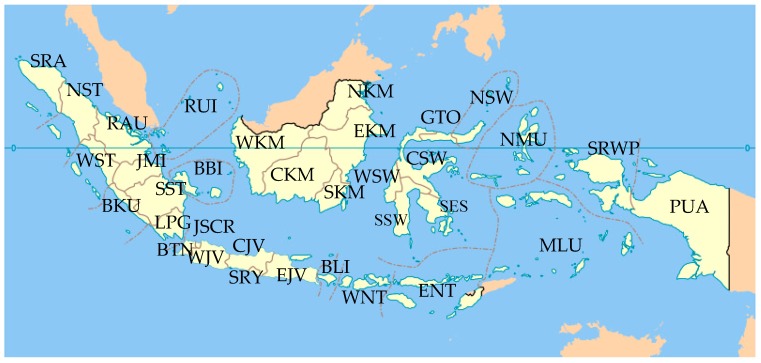
Map of Indonesia and its 34 provinces [15]: SRA (Special Region of Aceh), NST (North Sumatra), RAU (Riau), RUI (Riau Islands), WST (West Sumatra), JMI (Jambi), BKU (Bengkulu), SST (South Sumatra), BBI (Bangka–Belitung Islands), LPG (Lampung), BTN (Banten), JSCR (Jakarta Special Capital Region), WJV (West Java), CJV (Central Java), SRY (Special Region of Yogyakarta), EJV (East Java), BLI (Bali), WNT (West Nusa Tenggara), ENT (East Nusa Tenggara), WKM (West Kalimantan), CKM (Central Kalimantan), EKM (East Kalimantan), SKM (South Kalimantan), NKM (North Kalimantan), NSW (North Sulawesi), GTO (Gorontalo), WSW (West Sulawesi), CSW (Central Sulawesi), SSW (South Sulawesi), SES (Southeast Sulawesi), NMU (North Maluku), MLU (Maluku), SRWP (Special Region of West Papua), and PUA (Papua).

Indonesia is located at one of the centers of biodiversity hotspots [13], endowed with a coral reef triangle, and is an epicenter of marine biodiversity covering 10.36 million km^2^ of ocean and coastal waters surrounding Indonesia, Malaysia, Papua New Guinea, the Philippines, Timor Leste, and Australia [14]. The country consists of 34 provinces (Figure 1) [15] and surrounding water areas encompassing 5.79 million km^2^, of which 1.08% or 62,600 km^2^ is protected as national parks [16]. The features of this area include not only species richness, endemism and habitat diversity, but its relatively pristine condition. An understanding of the fundamental biodiversity [17,18] gained through investigation of bioactive molecules is required by having skills to do individual chemical investigations with small specimens to reduce costs and to minimize environmental impact. Another way is to use fermentation and culture of marine microbes. Although permission to collect and export specimens has been given, researchers must be aware and respectful of indigenous knowledge and cultural sensitivities [19]. Chemists are not yet adept at easily creating molecules with high potency for use as medicines, whereas nature has an enormous, almost incomprehensible, capacity for diversity and adaptation [20,21]. Perhaps the most important way of solving biodiversity problems is through the synthesis of past and future data not only on the basis of taxonomic and spatial distribution, but also through chemical and biological—including ecological and genetic, as well as medicinal—perspectives. This would be in tandem with national and international collaborative research programs [22,23]. The wise use of cutting-edge technology and access to biodiversity through benefit-sharing agreements are believed to conserve our global ecosystems while advancing science. 

**Figure 2:**
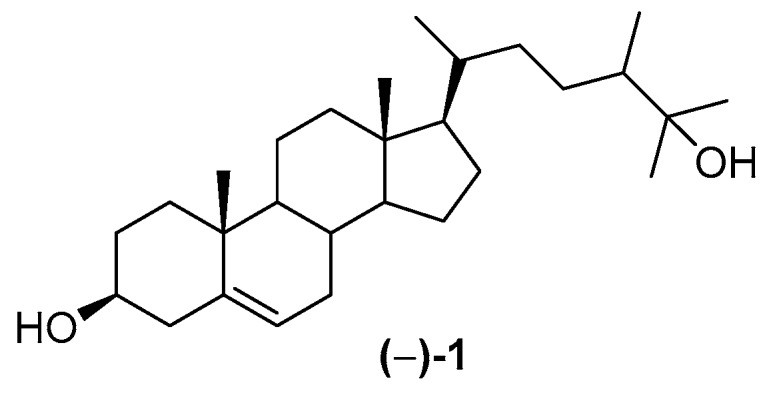
Structure of (–)-25-hydroxy-24ξ-methylcholesterol **1**.

Although Indonesian MNPs have been reviewed [24,25,26], there is no comprehensive review from the earliest research of Indonesian MNPs to the present. The earliest observation of marine products was realized by poisoning with clupeoid fish [27], while the first MNP discovered in Indonesian waters was (−)-25-hydroxy-24ξ-methylcholesterol **1** [28], isolated in 1972 (Figure 2).

**Figure 3:**
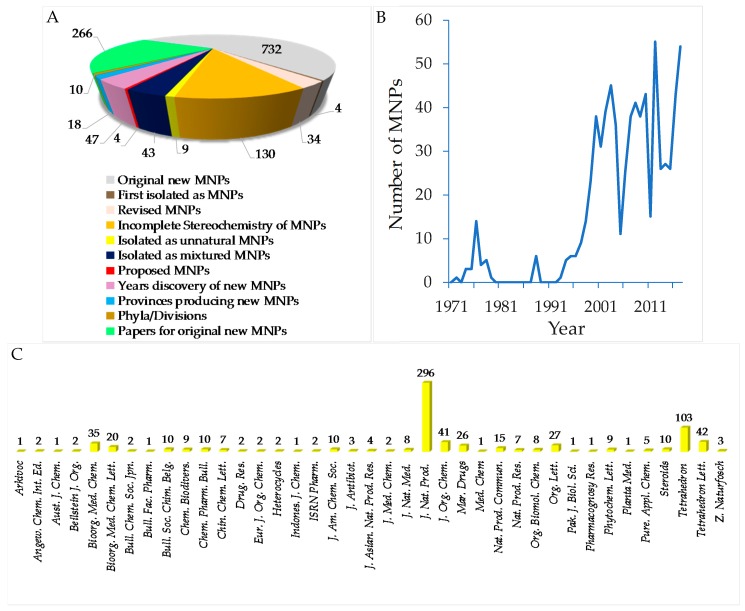
Statistics of new Indonesian MNPs from January 1970 to December 2017 (**A**). Distribution of new MNPs on the basis of their publication per year (**B**) and journal titles (**C**).

To provide a current status of Indonesian MNPs, we performed a literature review of new chemical structures found in Indonesian waters, including their chemistry, biological activities, spatio-temporal dimensions, taxonomy, and dissemination of information from marine macro- and microorganisms published during the period January 1970–December 2017. An estimated 15,500 new MNPs were discovered worldwide between 1970 and 2010 [29], among which about 486 new molecules (3.1%) were found in Indonesia. In the period 1990–2009, the number of new MNPs worldwide was 9812 [30], while that from Indonesian waters was 406 (4.1%). The present work covered a total of 732 original MNPs, with 4 known to be synthetic compounds, 34 revised, and 9 unnatural MNPs, from January 1970 to December 2017 (Figure 3A). In addition, 43 MNPs were isolated as mixtures, while 130 MNPs were reported with incomplete stereochemistry. Of 732 MNPs, none of them has been approved or is under clinical trial.

Before 1990, the exploration trend of Indonesian MNPs was relatively steady; however, after 1990 the discovery of new molecules has increased significantly (Figure 3B). More specifically, in the period of 1970–1979, 31 terpenoids were the only MNPs reported. In the next decade, 1980–1989, the structural types of Indonesian MNPs were alkaloids (4 molecules) and polyketides (2 molecules). From 1990 to 1999, the numbers of Indonesian MNPs were: 27 terpenoids, 19 alkaloids, 11 polyketides, 5 peptides, 1 fatty acid, and 1 carbohydrate. The discovery of new Indonesian MNPs was at its highest in the period 2000–2009, with 139 terpenoids, 121 alkaloids, 44 polyketides, 30 peptides, and 8 fatty acids. In the current decade, 2010–2017, the structural types of Indonesian MNPs were 116 alkaloids, 79 terpenoids, 64 polyketides, 25 peptides, 4 fatty acids, and 1 carbohydrate. Alongside improvement of techniques for structure elucidation, separation, and synthesis, the increased number of bioactive Indonesian MNPs in the last two decades may be due to logistical ease, as many Indonesian biodiversity hotspots are in remote areas. 

The original Indonesian MNPs were reported in 266 papers in 39 different journals. Among them, *J. Nat. Prod.* is placed for top tier dissemination (296 molecules, 40.4%) followed by *Tetrahedron* (103 molecules, 14.1%), *Tetrahedron Lett.* (42 molecules, 5.7%), and *J. Org. Chem.* (41 molecules, 5.6%) (Figure 3C). Of 266 papers, 117 papers (44.0%) were written by local researchers who are affiliated with Indonesian research centers/universities. 

**Figure 4:**
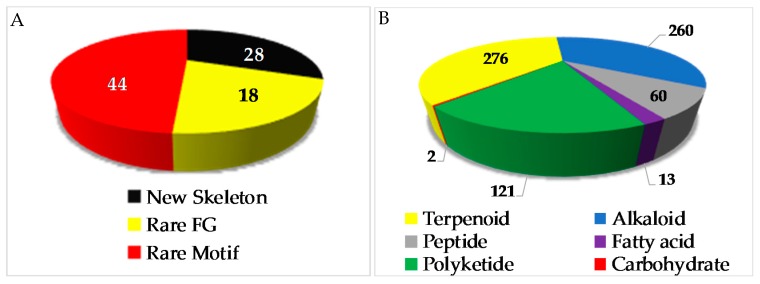

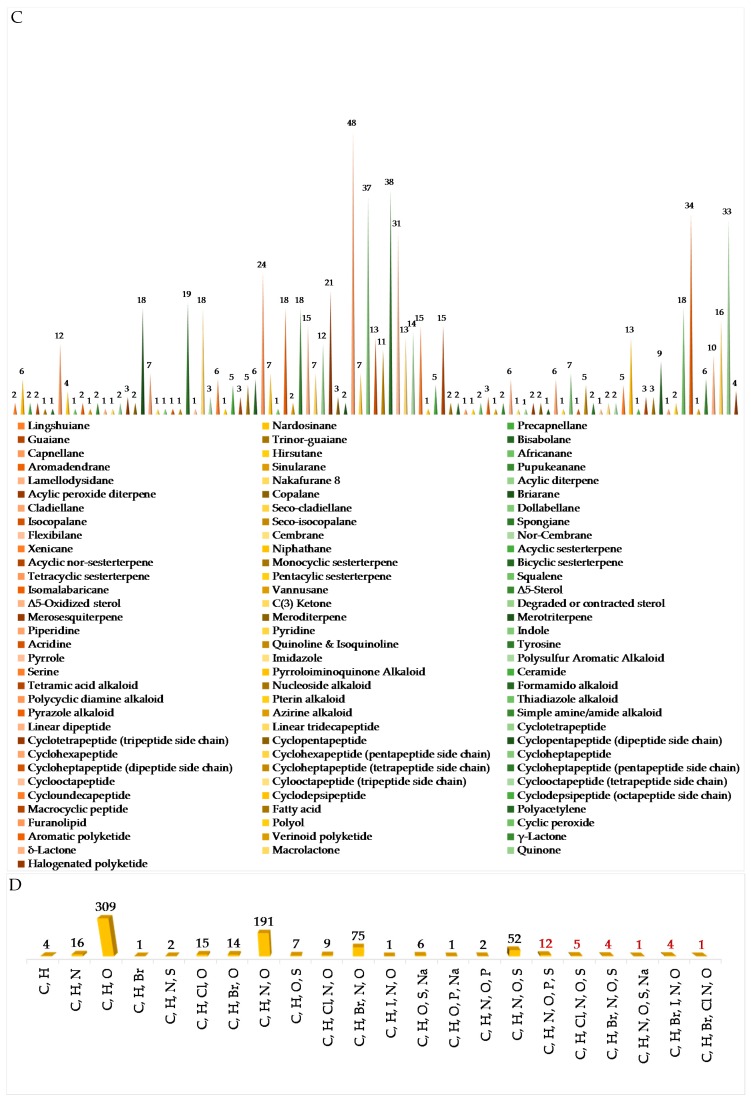
Distribution of new Indonesian MNPs on the basis of chemical skeletons (**A**), classes (**B**), chemical types (**C**), and atomic diversities (**D**).

The chemical diversity of Indonesian MNPs has been analyzed with respect to carbon skeleton, functional group, rare motif, and atomic diversity. New carbon skeletons were observed in 28 molecules, while rare FGs and motifs were found in 18 and 44 molecules, respectively (Figure 4). A few examples of new carbon skeletons can be seen in vannusal A (**198**), halioxepine (**281**), manadomanzamine A (**318**), and phormidolide (**702**), while rare FGs and motifs can be seen in sinulasulfone (**44**), polycarpaurine C (**487**), siladenoserinol A (**505**), lanesoic acid (**541**), and petroquinone A (**734**), to name a few. In this review, the MNPs are organized into their structural types, consisting of 276 terpenoids (37.7%), 260 alkaloids (35.5%), 60 peptides (8.2%), 13 fatty acids and linear molecules (1.8%), 121 polyketides (16.5%), and 2 carbohydrates (0.3%) (Figure 4B). Among 97 chemical types of Indonesian MNPs, piperidine alkaloids (48 molecules), tyrosine alkaloids (38 molecules), indole alkaloids (37 molecules), aromatic polyketides (34 molecules), and quinones (33 molecules) are listed as the top five chemical types (Figure 4C). 

The evaluation of atomic diversity within Indonesian MNPs (Figure 4D) shows that 27 molecules (written in red numbers) contain 6 different atoms in 1 molecule, while the majority (326 molecules) contain 3 different atoms in 1 molecule. Most of them contain C, H, O, N, with the addition of Br and I (4 molecules), as in enisorine E (**438**), agelanesins B (**464**) and D (**466**), 1-*O*-methylhemibastadinol 4 (**440**); Br and Cl (1 molecule), as in diazonamide E (**611**); Br and S, as in mauritamides D (**462**), B (**467**) and C (**468**); Cl and S (4 molecules), as in dysithiazolamide (**552**), biakamides A–D (**742**–**745**); P and S (12 molecules), as in siladenoserinols A–L (**505**–**516**); Na and S (1 molecule), as in cupolamide A (**564**). Of 732 molecules, 373 (51.0%) are nitrogenous molecules. Further inspection of the atomic diversity indicated that 122 molecules (16.7%) possess a ratio of H/C < 1, which is often challenging with respect to structure elucidation [31]. The smallest H/C was found in cadiolide B (**679**) (H/C = 0.4). In terms of molecular weight, the biggest MNP is kakelokelose (**747**), with an estimated molecular weight of between 3 and 500 kDa, while the smallest MNP was plakofuranolactone (**683**), at 172 Da. The biggest unsaturation number (Un) 43 of petroquinones A (**734**) and B (**735**) is composed of 16 rings and 27 double bonds, while the smallest is 1 in strepsiamides A–C (**518**–**520**).

The chemical diversity of Indonesian MNPs can also be reflected by the use of several orthogonal-tactic-classic and modern structure elucidations describing the nature of Indonesian MNPs, such as molecular size, complexity, and type and distribution of functional groups. Having a single crystal molecule, the structure elucidation task is more straightforward and secured to perform by employing X-ray crystallography to reveal the 2D and 3D molecular structure, including absolute configuration and conformation. Of 732 original MNPs, 30 MNPs were determined by X-ray crystallography in combination with other spectroscopic data. In addition, three revised MNPs, vannusal B (**199b**), *trans, trans*-[D-*allo*-ile] ceratospongamide (**566b**) and **659b**, were securely determined with the aid of X-ray diffraction.

The presence and position of nitrogen atoms and their correlations to hydrogen or carbon atoms within the molecules can be detected by ^15^N NMR and NH-HMBC as in manadomanzamine A (**318**), *neo*-kauluamine **(321**), lanesoic acid (**541**), polycarpathiamine A (**544**), sintokamide A (**553**), and *cis*, *cis*-ceratospongamide (**565**), while P-containing molecules can be evaluated by the use of ^31^P NMR as in siladenoserinol A (**505**). The planar structure of the 10-membered polysulfur ring, as in lissoclibadin 1 (**498**), could be elucidated by applying NOEs with a combination of other tactics such as quantum chemical calculation (QCC). The presence of a sulfate group can be detected by infrared (IR) and confirmed by mass spectrometry (MS)/MS fragmentation, as in polycarpaurines B and C (**497**–**498**). 

For cyclic molecules with rigid three- to six-membered rings, their relative stereochemistry can be elucidated by analyzing ^1^H–^1^H spin coupling constants (^3^*J*_HH_), chemical shifts and NOEs. For geometrically flexible molecules such as multiple stereocenters of acyclic chains or macrocycles, it cannot be concluded with NOEs. To handle such molecules, NMR-based approaches including *J*-based configuration analysis (JBCA), universal NMR database (UDB), theoretical calculation of NMR parameters and residual dipolar couplings (RDCs) [32] were applied as tools to determine the relative configuration of natural products. Of 732 molecules, relative stereochemistry of at least four molecules **541**, **552**, **702**, and **708** were elucidated with the aid of JBCA method, while the relative configuration of one molecule as in **595** was elucidated by JBCA-QCC tactics. 

The absolute configurations (ACs) of MNPs can be elucidated by NMR in two approaches: (a) substrate analysis without derivatization (i.e., by the addition of a chiral solvating agent (CSA)) and (b) analysis of the diastereomeric derivatives prepared with a chiral derivatizing agent (CDA) [33]. The ACs of 31 Indonesian MNPs were determined by applying CDA. If chiral molecules possess appropriate chromophore(s), electronic circular dichroism (ECD) can be applied, as in 56 MNPs. Comparison of the ECD calculated by the time-dependent density functional theory (TDDFT) with the experimental ECD spectra was performed for the ACs of as-exemplified lamellodysidine A (**41**), niphatheolide A (**128**), sulawesin A (**133**), and nakamuric acid (**448a**). However, nakamuric acid (**448a**) was first revised to **448b_1_** by synthesis [34], and later to **448b_2_** based on the comparison of experimental and calculated ECD spectra [35]. Molecular modelling, total synthesis, and QCC were used to determine the structure of vannusals A (**198**) and B (**199**) [36,37,38,39,40,41,42,43]. Combination of NMR and chemical degradation helped to determine the long carbon chain molecules, karatungiols A (**655**) and B (**626**) [44]. For small heterocyclic molecules with the ratio of H/C < 1, total synthesis is helpful for confirming unusual rings, as in latonduine A (**450**) [45] and polycarpathiamine A (**544**) [46].

**Figure 5:**
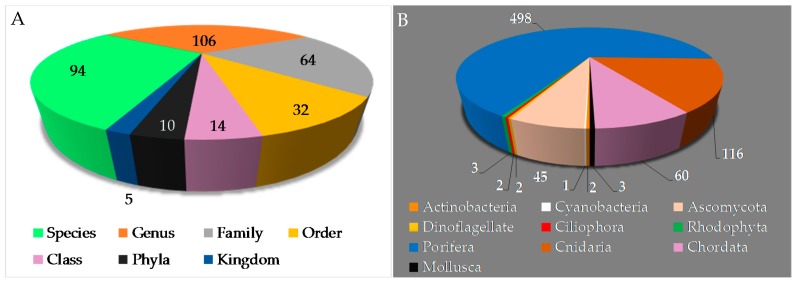
Distribution of new Indonesian MNPs on the basis of biological sources (**A**,**B**).

With respect to phylogeny, new MNPs have been discovered from 5 kingdoms, 10 phyla, 14 classes, 32 orders, 64 families, 106 genera, and 94 species (Figure 5A,B) in Indonesian waters over the past 47 years. Three phyla (Porifera, Cnidaria, and Chordata) are found to be the major sources (498 molecules, 68.0%; 116 molecules, 15.8%; and 60 molecules, 8.2%, respectively) of novel metabolites. The remaining 8.0% were discovered from the following phyla: Mollusca (3 molecules, 0.4%), Rhodophyta (3 molecules, 0.4%), Ciliophora (2 molecules, 0.3%), Dinoflagellate (2 molecules, 0.3%), Ascomycota (45 molecules, 6.1%), Cyanobacteria (1 molecule, 0.1%), and Actinobacteria (2 molecules, 0.3%). The phylum Porifera, the largest source of Indonesian MNPs, consists of three classes: Demospongiae (91.8%), Homoscleromorpha (6.6%) and Calcarea (1.6%). Among these, Demospongiae is comprised of 11 orders and 32 families. The Cnidaria is comprised of 1 class (Anthozoa), 3 orders (Alcyonacea, Pennatulacea, Actinaria), and 9 families (Alcyoniidae, Nephtheidae, Xeniidae, Briareidae, Ellisellidae, Isididae, Veretillidae, Pennatulidae, and Stichodactylidae). 

**Figure 6:**
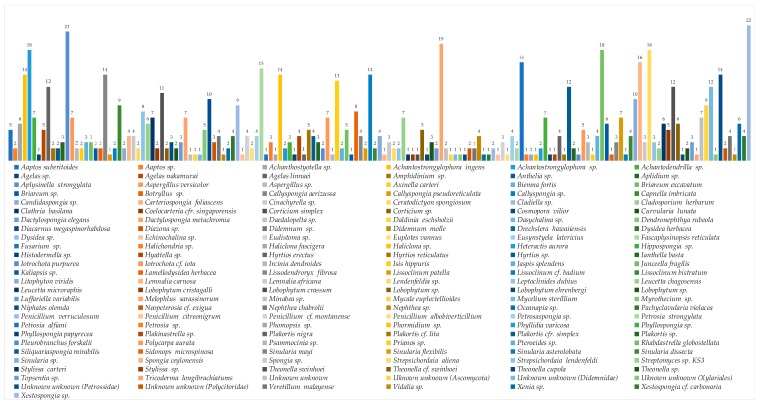
Distribution of new Indonesian MNPs on the basis of biological sources and a list of species of Indonesian marine organisms reported to contain new MNPs. *Unknown unknown* is an unidentified species from certain phyla.

The top 10 genera reported for new MNPs are Achantostrongylophora (4.4%), Xestospongia (3.6%), Sinularia (3.4%), Apysinella, Theonella and Strepsichordaia (each 2.9%), Plakortis, Petrosia, Spongia, Melophlus (each 2.6%), Agelas, Rhabdastrella, and Lissoclinum (each 2.5%). Unknown genera were the sources of 6.2% of new MNPs. Symbiotic relationships are generally found between sponges and fungi, algae and fungi, and dinoflagellates and acoel flatworms. The list of Indonesian marine organisms producing new MNPs is described in Figure 6. 

**Figure 7:**
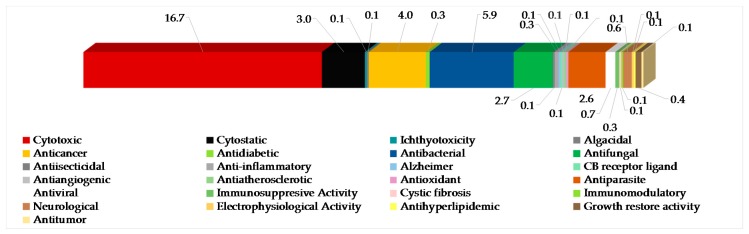
Distribution of new Indonesian MNPs on the basis of their significant biological activity.

The most frequently evaluated biological activities of Indonesian MNPs is cytotoxicity (122 molecules, 16.7%) (Figure 7). Results are generally expressed in the terms of the dose or concentration that inhibits cell growth to 50% of the control (ED_50_, EC_50_, ID_50_, IC_50_, LD_50_, LC_50_ in μg/mL or μM), and the criterion for a cytotoxic compound is ED_50_ < 4 μg/mL [47]. Cytotoxic evalution has been performed on cell-based (120 human, 26 murine, and 1 monkey *Cercopithecus aethiops* cell lines), enzyme-based (mainly protease and kinase), and brine shrimp (*Artemia salina*) assays. Of 122 molecules, four molecules—**60**, **619**, **652**, and **702**—showed significant toxicity against *A. salina*. Antibacterial activity has been the second most frequently used bioassay for Indonesian MNPs, with 43 molecules (5.9%) showing significant results followed by anticancer (4.0%), cytostatic (3.0%), antifungal (2.7%), and antiparasitic activity (2.6%). Of 4.0% anticancer molecules, only 2.3% showed anticancer activity without cytotoxicity. It is noteworthy that at least 86 out of 260 alkaloids have been isolated and tested as salt forms against various targets of assays. In addition, about 609 molecules have been used for in vitro assay, while 10 molecules had been used for in vivo evaluation. 

**Figure 8:**
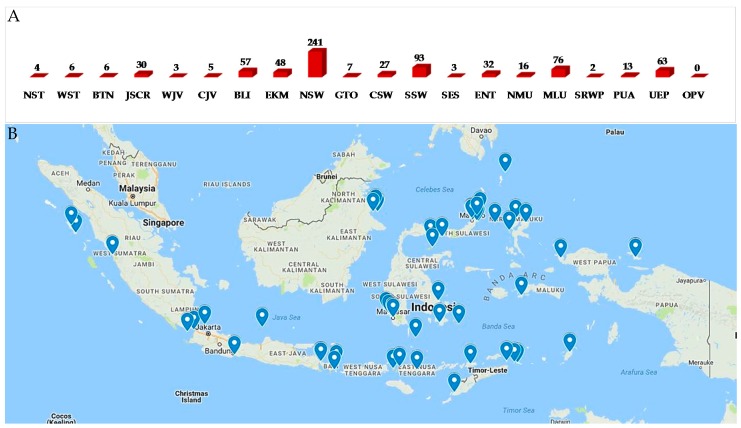
Distribution of new Indonesian MNPs on the basis of their biogeography hotspots (**A**,**B**) (OPV other provinces).

Biogeography of Indonesian MNPs shows that 18 (NST, WST, BTN, JSCR, WJV, CJV, BLI, ENT, EKM, NSW, GTO, CSW, SSW, SES, NMU, MLU, SRWP, and PUA) of 34 provinces are sources of new MNPs. Among the 18 provinces, more than 70% of the total new MNPs are supplied by five provinces: NSW (33.0%), SSW (12.7%), MLU (10.4%), BLI (7.8%), and EKM (6.6%) (Figure 8A,B). The frequent discovery of Indonesian MNPs in the Eastern part of Indonesia may be due to it being closer to the center of Coral Triangle.

From the above results, it can be seen that Indonesian marine macro- and micro-organisms are still largely underexplored and may provide viable sources and inspiration for a large number of new chemical entities. Modern structure elucidation and other strategies are keys to providing new therapeutic agents and/or new tools for life science studies. 

Data presented in Figure 1, Figure 2, Figure 3, Figure 4, Figure 5, Figure 6, Figure 7, Figure 8, Figure 9, Figure 10, Figure 11, Figure 12, Figure 13, Figure 14, Figure 15, Figure 16, Figure 17, Figure 18, Figure 19, Figure 20, Figure 21, Figure 22, Figure 23, Figure 24, Figure 25, Figure 26, Figure 27, Figure 28, Figure 29, Figure 30, Figure 31, Figure 32, Figure 33, Figure 34, Figure 35, Figure 36 and Figure 37, Figures S1–S22 and Tables S1–S22 were assembled from the SciFinder^®^, Scopus^®^, and MarinLit databases and through manual curation of all published articles from an extensive panel of journals in chemistry and chemical biology fields. We emphasized new structures and structural revisions as elucidated through a variety of modern methods. Biological activities, origins of organisms, bioorganic studies on the MNPs, and syntheses that led to revision of structures or stereochemical assignments were also highlighted. We hope this comprehensive review will provide a useful overview and will help direct future efforts in Indonesian scientific development, governance, resource management, and conservation regarding the value of marine biodiversity. 

## 2. New MNPs Discovered from Indonesian Waters in the Period 1970–2017

### 2.1. General

There are five important components in MNP research programs: (1) collection and identification of marine organisms or culture of microorganisms, (2) screening of crude extracts for bioactivity or chemical structures, (3) isolation and structure elucidation of MNPs, (4) pharmacological evaluation of isolated compounds, and (5) further development of MNPs for science and technolgy. Self-contained underwater breathing apparatus (SCUBA) diving is generally used to collect shallow-water marine biota. SCUBA is also used to take photographs for characterization of specimens and to record ecological information on marine biota. The specimens are sorted and stored either frozen in aqueous ethanol or as dried material. Voucher specimens are prepared for taxonomic studies. At greater depths, specimens are collected by rebreather diving, dredging, trawling, and submersibles.

Many challenges must be overcome for this work. The presence of inorganic salt may become a problem if the sample amount is minuscule and the compounds are water-soluble, making them more difficult to handle than lipophilic compounds. When the bioassay is based on the behavior of marine organisms, it is difficult to mimic the marine environment. A routine procedure for isolation work is to group molecules by the level of their polarity, followed by separation and purification of target fractions using a variety of chromatographic methods. Bioassay or a signature of functional groups (substructures) or even molecular weight can be used as a guide for isolation or identification of MNPs. As a rule, in vitro bioassays require very little material, and take a shorter time to perform. It is normal to screen crude extracts with in vitro assays and reserve in vivo assays for pure compounds. Thin layer chromatography (TLC), NMR, and MS spectra can be used to identify characteristic extracts or fractions as guides for isolation work.

Modern structure elucidation requires a variety of spectroscopic methods (NMR, MS, IR, ultraviolet (UV), ECD, X-ray diffraction, vibrational circular dichroism (VCD), and others), chemical transformations (derivatives or selective degradation or total synthesis), molecular modeling or computational calculations, information technology with molecular networking, and biosynthetic consideration in order to reveal the planar and stereostructure of target molecules. In general, the chemical structure of molecules with low H/C ratios (<1) is challenging to elucidate [31]. To find a new molecule, one should consider a strategy of dereplication using NMR, LC-MS, MS/MS, or DNA sequence [48], in addition to exploring new groups of marine organisms and geographical selection of collection sites [30]. On the other hand, known molecules can be screened with a set of new assays in order to find new function of molecules.

### 2.2. Terpenoids

A total of 276 marine terpenoids were discovered within the period, consisting of 38 sesquiterpenoids, 84 diterpenoids, 50 sesterterpenoids, 21 triterpenoids, 52 steroids, 5 saponins, and 26 meroterpenoids. Among them, six molecules **10a**, **33a**, **70a**, **71a**, **198a**, and **199a** have been revised. One molecule, **53**, was isolated as a natural product, while it had been synthesized previously. Five molecules, **16c**, **17c**, **18c**, **45c**, **200c**, and **252c**, were isolated as derivatives. Of 276 terpenoids, ACs of 18 molecules were determined by X-ray crystallography. In addition, ACs can also be revealed by the use of ECD as in 21 terpenoids. Modified Mosher’s method was applied for 14 chiral terpenoids, while total syntheses proved the ACs of 3 terpenoids. Since the biological activity of true natural products is preferable, one should consider replacing the FG with the original one. Alternatively, the chromatography system can be modified so that the original molecule can be obtained. Indonesian marine terpenoids were found to show cytotoxic (37 molecules), cytostatic (18 molecules), anticancer without cytotoxicity (4 molecules), antifungal (5 molecules), antibacterial (3 molecules), antidiabetic (2 molecules), ichthyotoxic, algicidal, antiinsecticidal, anti-inflammatory, anti-Alzheimer, physicoactive (CB receptor ligand), antiangiogenic, antiatherosclerotic, and antioxidant activity (each 1 molecule). 

#### 2.2.1. Sesquiterpenoids

**Figure 9:**
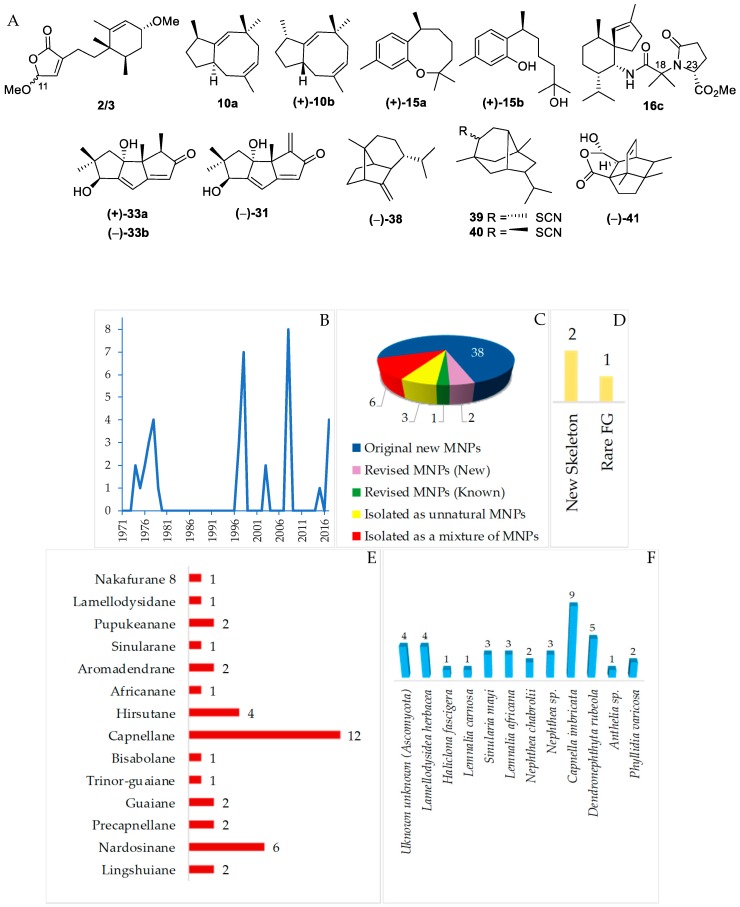

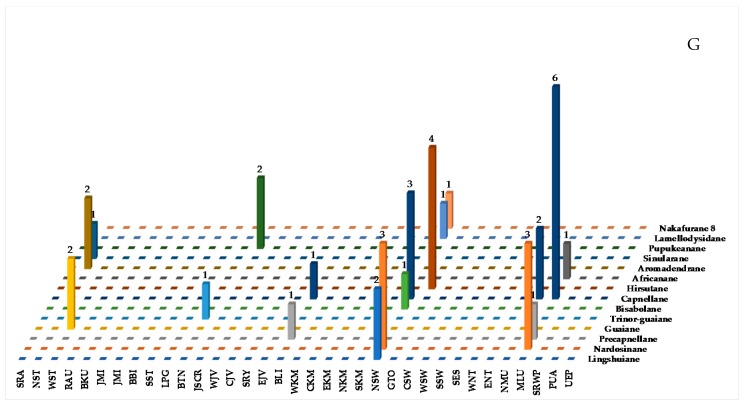
Structures of marine sesquiterpenoids from Indonesian waters found in 1970–2017 (**A**): representative. Distribution of new marine sesquiterpenoids by year (**B**). Statistics of new marine sesquiterpenoids (**C**). Distribution of new marine sesquiterpenoids on the basis of their skeletons (**D**,**E**), biological sources (**F**), and biogeography (**G**).

Sesquiterpenoids isolated from Indonesian waters from January 1970 to December 2017 are summarized in Figure 9A, Figure S1 and Table S1 of the Supplementary Materials. As shown in Figure 9B, some sesquiterpenoids had been discovered in an earlier period (1970–1980). The marine sesquiterpenoids are comprised of 38 new natural products, 3 revised molecules (2 new and 1 known), 3 derivatives **16c**, **17c** and **18c**, and 6 mixtures (Figure 9C). Two molecules, **2**/**3** and **41**, have been reported to have new skeletons, and 1 molecule, **39**/**40**, contains a rare functional group (Figure 9D). Fourteen different types of carbon skeletons (Figure 9E) are observed. Among them, capnellane (12 molecules), nardosinane (6 molecules), and hirsutane (4 molecules) are top three. Two molecules remain to be determined with respect to their stereochemistry (**2**/**3** and **39**/**40**). The ACs of six sesquiterpenoids, **4**, **7**, **22**, **31**, **35**, and **38**, and one derivative, **16c**, have been determined by X-ray crystallography. In addition, ECD was applied to 6 molecules, **2**/**3**, **12**, **38**, **41**, and **42**. The modified Mosher’s method was applied to **31**. Of 38 molecules, one molecule, **10a**, was revised to **10b** by total synthesis [49,50,51,52]. The structure of **15a** was corrected to **15b** after total synthesis [53,54,55,56,57]. Hirsutanol C (**33a**) was revised to **33b** by the work of a fungal metabolite [58]. Four phyla, Ascomycota (4 molecules), Porifera (5 molecules), Cnidaria (27 molecules), and Mollusca (2 molecules), were recorded to be sources of new sesquiterpenoids (Figure 9F). The new molecules are mainly found in NSW and MLU (Figure 9G). 

**Figure 10:**
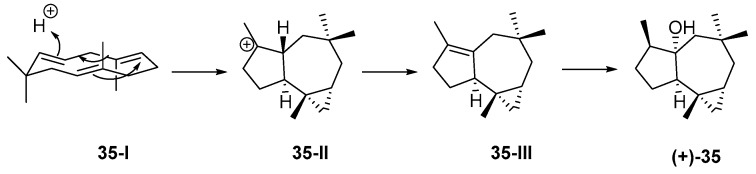
Plausible biosynthetic pathway of (+)-africanol **35.**

The first sesquitepenoid found in Indonesian waters was africanol (**35**) from *Lemnalia africana* and *Lemnalia nitida*, collected off Tanimbar, MLU. Its structure, including AC, was established by X-ray analysis [59] and confirmed by total synthesis [60,61]. It was proposed that **35** was derived from a humulene through its CT (cross and parallel rearrangements of two double bonds) conformer **35-I**, which undergoes acid-catalyzed closure to the 9-africyl cation **35-II**, followed by proton loss and hydration to provide **35** (Figure 10). Africanol (**35**) showed toxicity against guppy *Lebistes reticulatus* and unicellular algae *Chaetoceros septentrionalis*, *Astrionella japonica*, *Thallasioscira excentricus*, *Protocentrum micans*, and *Amphidinium carterae* [62].

#### 2.2.2. Diterpenoids

**Figure 11:**
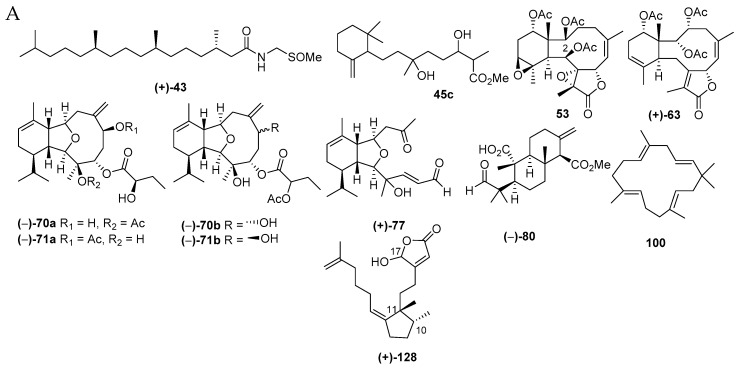

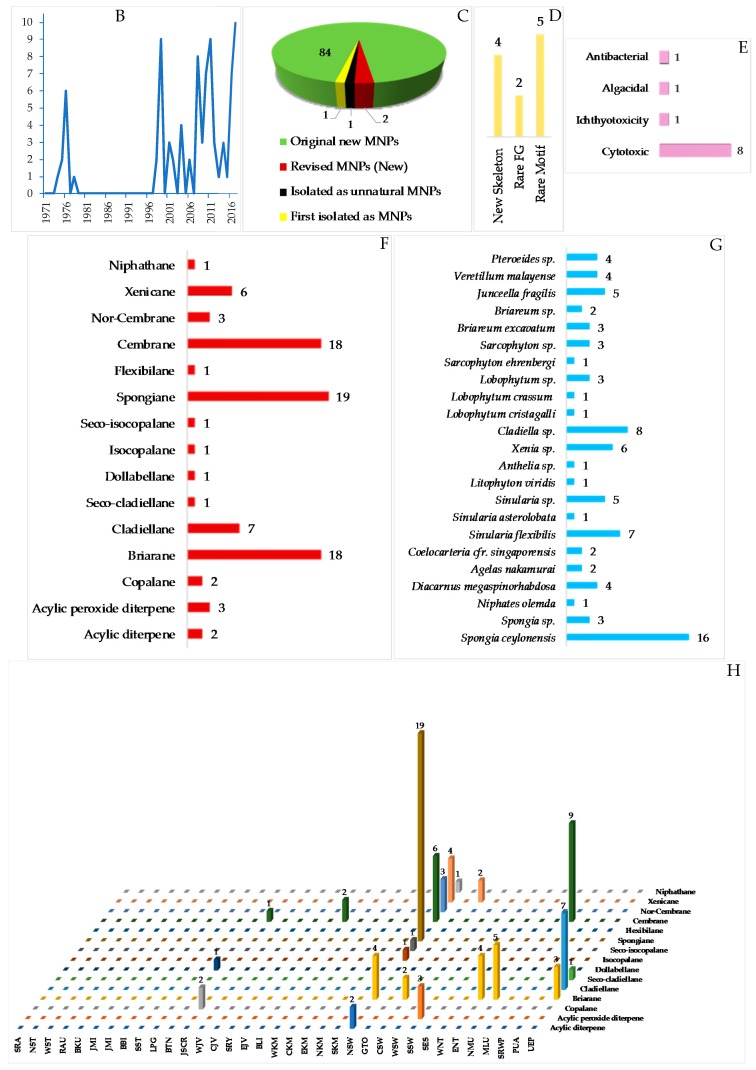
Structures of marine diterpenoids from Indonesian waters found in 1970–2017 (**A**): representative. Distribution of new marine diterpenoids by year (**B**). Statistics of new marine diterpenoids (**C**). Distribution of new marine diterpenoids on the basis of their skeletons (**D**,**F**), significant biological activity (**E**), biological sources (**G**), and biogeography (**H**).

Diterpenoids are molecules more frequently found from Cnidarian (56 molecules, 66.7%), particularly Alcyonacea (57.1%), than from Porifera (28 molecules, 33.3%). The diterpenoids found in Indonesian waters from January 1970 to December 2017 are compiled in Table S2 in the Supplementary Materials, Figure 11A and Figure S2. As for sesquiterpenoids, initial studies on diterpenoids were performed in 1970s, and publication increased after 1996 (Figure 11B). All of these efforts resulted in 84 new, 2 revised, 1 derivative, and 1 known but first from marine (Figure 11C). Among the new molecules, 4 have new skeletons, **80**, **100**, **117**, and **128** (Figure 11D), 2 molecules contain rare FGs, sulfoxide or sulfone, and 5 molecules are listed to possess a rare structural motif, **77**, **79**, **113**, **119**, and **124** (Figure 11D). The chemical diversity of Indonesian marine diterpenoids (84 molecules) was proved, as there were 15 different skeletons: 19 spongianes, 18 each of briaranes and cembranes, 7 cladiellanes, 6 xenicanes, 3 each of nor-cembranes and acyclic peroxide diterpenes, 2 each of copalanes and acyclic diterpenes, and 1 each of niphatane, flexibilane, *seco*-cladiellane, dollabelane, isocopalane, and *seco*-isocopalane (Figure 11F). Four molecules, **101**, **108**, **110**, and **112**, were determined by X-ray analysis. ECD was used to reveal the ACs of 8 molecules, **43**, **44**, **79**, **82**, **88**, **89**, **90**, and **128**. In particular, 5 molecules, **82**, **88**–**90** and **128**, were elucidated by comparing actual spectra and calculated ECD. The modified Mosher’s method was applied for 8 molecules, **54**–**57**, **63**, **70a**, **113**, and **124**. Two molecules, **70a** and **71a**, were revised to **70b** and **71b**. NSW is the most favored place for finding new marine diterpenes (41 molecules, 48.8%), followed by MLU (10 molecules, 11.9%) (Figure 11H). One molecule, **45c**, was isolated as a derivative, while molecule **53** was isolated as a natural product for the first time, but was previously known as a semisynthetic. 

The AC of niphateolide A (**128**), an inhibitor of p53-Hdm2 interaction [63] from the sponge *Niphates olemda,* was established as 10*R*,11*R* by ECD measurements in the vacuum-ultraviolet region based on theoretical calculation. The remaining stereocenter at C17 remains unsolved. 

#### 2.2.3. Sesterterpenoids

**Figure 12:**
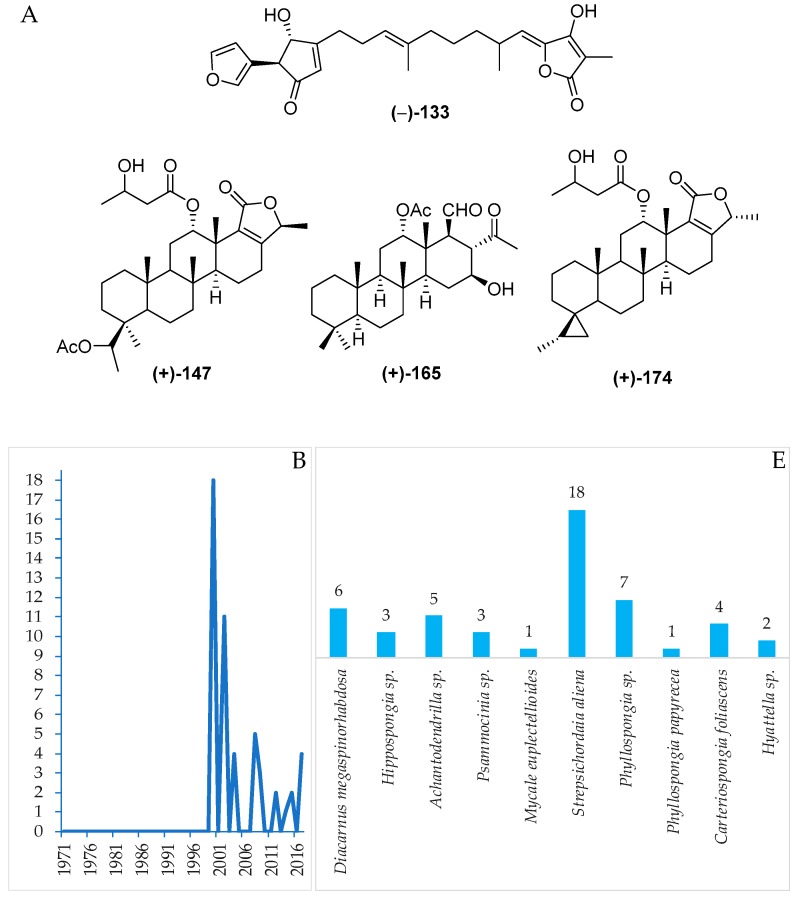

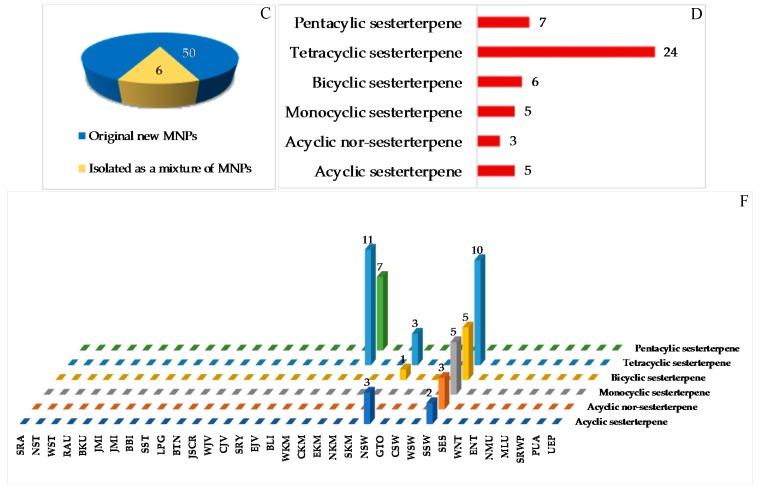
Structures of marine sesterterpenoids from Indonesian waters found in 1970–2017: (**A**) representative. Distribution of new marine sesterterpenoids by year (**B**). Statistics of new marine sesterterpenoids (**C**). Distribution of new marine sesterterpenoids on the basis of their skeletons (**D**), biological sources (**E**), and biogeography (**F**).

The sestertepenoids found in Indonesian waters from January 1970 to December 2017 are shown in Table S3 and Figure 12A, Figure S3. The first sesterterpenoids were **147**–**152** and **173**–**178**, isolated from *Strepsichordaia aliena* in 2000. Of the 56 sesterterpenoids reported, 50 were new and 6 were isolated as mixtures (Figure 12C). The molecules are comprised of 24 tetracylic followed by 7 pentacyclic, 6 bicyclic, 5 each of monocyclic and acyclic sesterterpenoids, and 5 acyclic norsesterterpenoids (Figure 12D). Three molecules, **148**, **165** and **174**, including their ACs, were revealed by X-ray crystallography, while the ACs of 2 molecules, **160** and **161**, were disclosed by comparing calculated and experimental ECD. The AC of **165** was determined by the modified Mosher’s method. The biological sources of Indonesian sesterterpenoids are exclusively from the phylum Porifera, class Demospongiae with 6, 9, and 10 differrent families, genera, and species, respectively (Figure 12E). In regard to biological activities, significant cytotoxicity was observed for 9 molecules, while others were recorded as exhibiting anticancer activity without cytotoxic (2 molecules), cytostatic, antidiabetic, and antifungal activities (1 molecule each) (Figure 12D). Marine sesterterpenoids were mainly found in specimens in SSW (25 molecules) and EKM (18 molecules) (Figure 12G). 

#### 2.2.4. Triterpenoids

**Figure 13:**
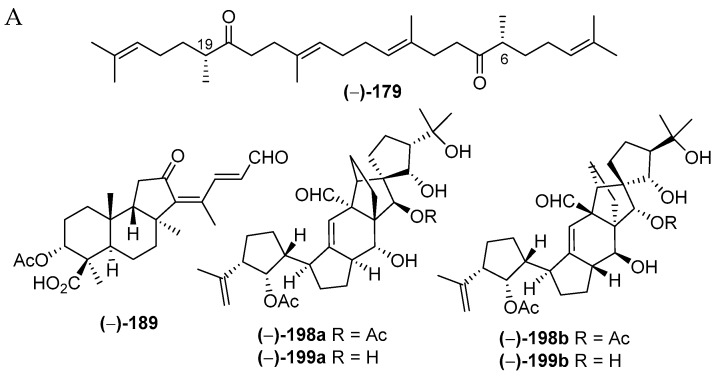

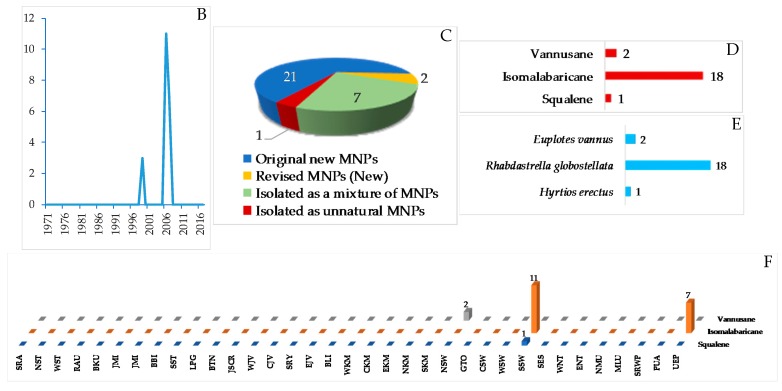
Structures of marine triterpenoids from Indonesian waters found in 1970–2017: (**A**) representative. Distribution of new marine triterpenoids by year (**B**). Statistics of new marine triterpenoids (**C**). Distribution of new marine triterpenoids on the basis of their skeletons (**D**), biological sources (**E**), and biogeography (**F**).

**Figure 14:**
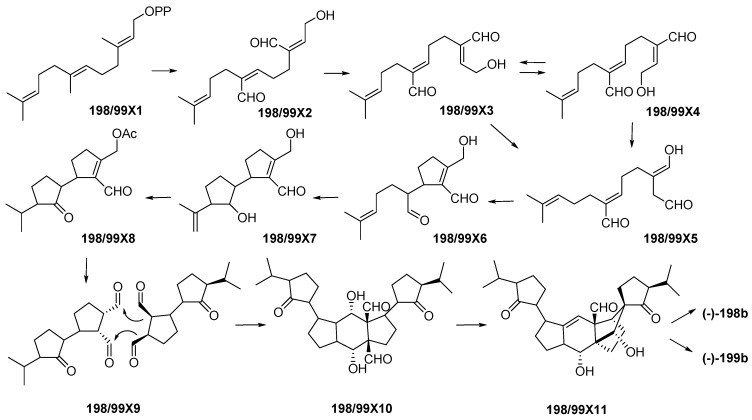
Plausible biosynthetic pathway of (–)-vannusals A **198b** and B **199b**.

Indonesian marine triterpenoids (Table S4 and Figure 13A, Figure S4) are comprised of 21 new, 7 mixtures, 1 derivative, and 2 revised molecules (Figure 13C). The triterpenes are grouped into three structural classes: squalane (1), isomalabaricanes (18) and vannusanes (2 molecules). Of 21, only vannusals A (**198**) and B (**199**) were found to have a new skeleton. The 3 skeletons were obtained from 3 species, *Euplotes vannus* for **198a** and **199a**, *Rhabdastrella globostellata* for **180**–**197**, and *Hyrtios erectus* for **179** (Figure 13C,D). The AC of 1 molecule, **199b**, was solved by X-ray analysis. Of the triterpenoids, six were found to show significant cytostatic activity. The metabolites were mainly isolated from specimens collected in SSW (12 molecules) (Figure 13F). Two molecules, **198a** and **199a**, were revised to **198b** and **199b** [36,37,38,39,40,41]. The stereochemistry of vannusal B (**199b**) was also examined by density functional theory (DFT) calculation [42]. A plausible biosynthetic pathway of vannusals A (**198b**) and B (**199b**) was proposed (Figure 14) [43].

#### 2.2.5. Steroids

**Figure 15:**
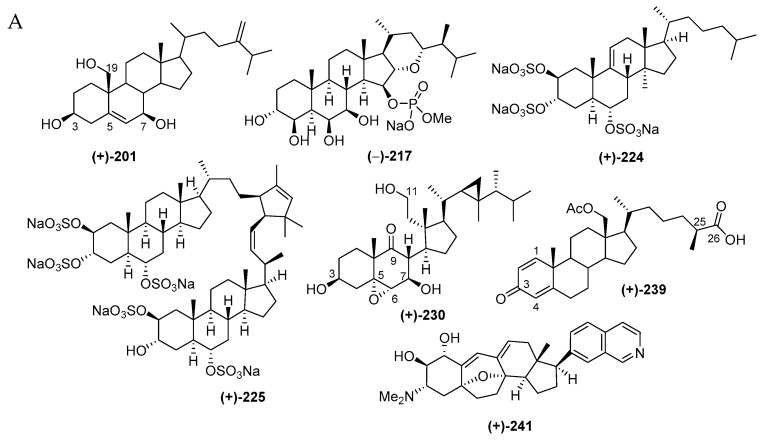

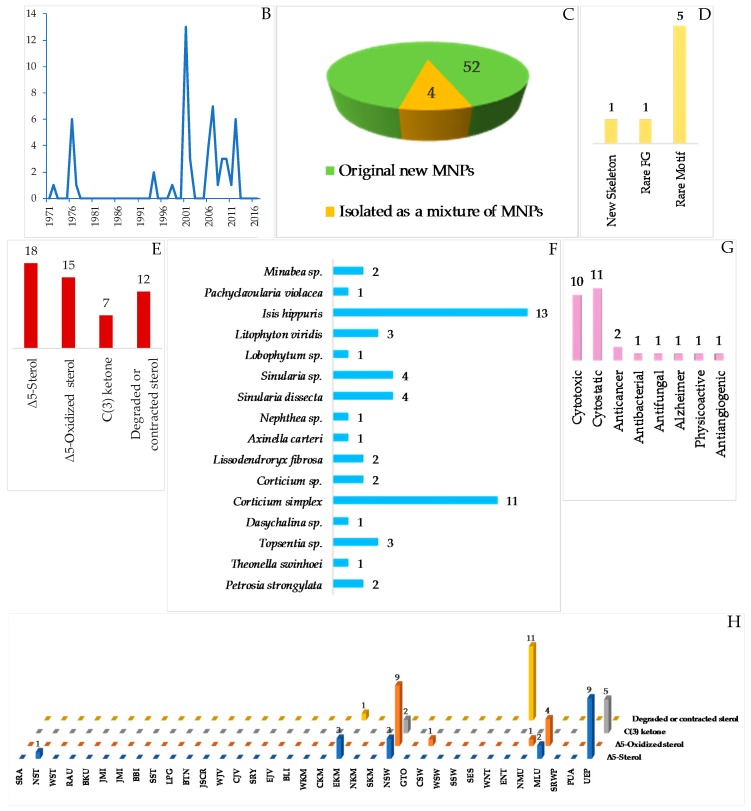
Structures of marine steroids from Indonesian waters found in 1970–2017: (**A**) representative. Distribution of new marine steroids by year (**B**). Statistics of new marine steroids (**C**). Distribution of new marine steroids on the basis of their skeletons (**D**,**E**), biological sources (**F**), significant biological activity (**G**), and biogeography (**H**).

Indonesian marine steroids found in the period of 1970–2017 are comprised of 52 pure molecules and 4 mixtures (Figure 15C) and are shown in Table S5 in Supplementary Materials and Figure 15A, Figure S5. The discovery trend increased in the first 5 years and also in the years after 1990 (Figure 15B). The ratio among new skeletons, rare functional groups, and rare motifs is 1:1:5 (Figure 15D). Four different skeletons are observed in 52 steroids (Figure 15E). Among them, Δ^5^-sterols constitute the majority (18 molecules). Sulfated steroids were reported as **203**, **206** and **224**–**226**, while a phosphated steroid was observed in **217**. From 1970–2017, Indonesian marine sulfated steroids have contributed 3.3% of the total marine sulfated steroids worldwide, while Indonesian marine phosphated steroids are expected to contribute 20% of total marine phosphated steroids. The ACs of three steroids, **201**, **204** and **241**, were disclosed by X-ray analysis, while that of **241** was determined by ECD spectrum. For two molecules, **224** and **239**, their absolute configurations were determined by MTPA esters and PGME methods, respectively. With regard to the biological sources, the steroids were mainly obtained from the phyla Cnidaria (29 molecules) and Porifera (23 molecules), as listed in Figure 15F. In regard to biological activities, 10 molecules showed significant cytotoxicity, while 11 molecules were cytostastic (Figure 15G). The molecules were isolated mainly from specimens from NSW (14 molecules) and ENT (11 molecules) (Figure 15H). 

The unique molecules in the steroids are cortistatins isolated from the sponge *Corticium simplex* collected off Flores, ENT, with a new skeleton comprised of a 9(10-19)-abeo-androstane and isoquinoline [64]. The structure of cortistatin A (**241**) was determined by X-ray analysis and the ECD excition chirality method. Molecules **241**–**244** showed selective antiproliferative properties against human umbilical vein endothelial cells (HUVECs). The most potent member, cortistatin A (**241**), showed a selectivity index of more than 3000 against HUVECs in comparison with human fibroblast (NHDF) and several other tumor cells KB31, K562 and Neuro 2A. Additional members, cortistatins E (**250**), F (**251**), G (**248**), and H (**249)** with *N*-methyl piperidine or 3-methylpyridine unit isolated from the same source, also showed antiproliferative activity against HUVECs [65]. Three additional cortistatins J (**245**), K (**246**) and L (**247**) were isolated from the same source [66]. The first synthesis of **241** verified its 3D structure, featuring an inexpensive terrestrial steroid prednisone as the starting material [67]. The second total synthesis of **241** was achieved by using intra-molecular oxa-Michael addition/aldol/dehydration cascade reaction, Sonogashira, and Suzuki-Miyaura couplings [68].

Molecules **241** and **245** were confirmed to show an antiproliferative effect on additional cancer cell lines: MCF7, NCI-H460, SF268, IA9, PTX22, and A8, including drug-resistant ones [69]. Structure–activity relationships with natural cortistatins and synthetic analogues suggested that substitution at position 7’ of isoquinoline is a key determinant of the phenotypic effects of cortistatins [69,70,71]. It is hypothesized that the biological activity of **241** is due to inhibition of one or more protein kinases. Molecule **241** inhibits the function of several different kinases in vitro. It is proposed that **241** may occupy the ATP-binding site of at least one of the following enzymes: Rho associated, protein kinase (ROCK), or cyclin-dependent kinase (CDK) 8 and 11 [72]. The X-ray crystallographic analysis of the ligand-protein complex disclosed that the isoquinoline binds to the kinase hinge, that the steroid region of the molecule is complementary to the shape of the ATP-binding cleft, that the terminal polar A ring is exposed to solvent, and that a salt bridge exists between an aspartate side chain (ROCK I only) and dimethylamino group of **241** [72].

#### 2.2.6. Saponins

**Figure 16:**
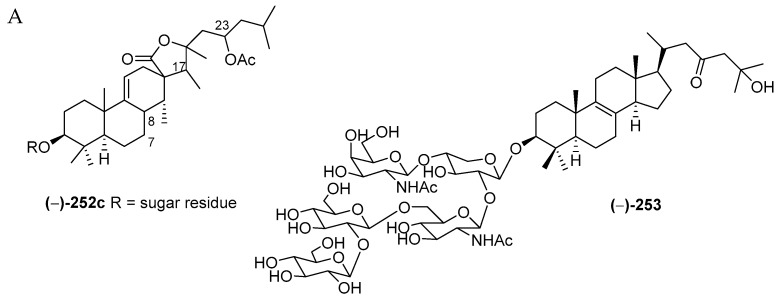
Structures of marine saponins from Indonesian waters found in 1970–2017: (**A**) representative.

Only five saponins **253**–**257** with 1 derivative **252c** were discovered in Indonesian waters in the period 1970–2017 (Table S6 and Figure 16A, Figure S6). All the saponins retain a lanostane skeleton with five sugar moieties. ECD spectrum has been used to reveal the stereochemistry of **252c**. Four molecules, **253**–**256**, were found in a specimen of the sponge *Melophlus sarassinorum* from SSW, while **257** was from a *Petrosia* sp. of NSW. Significant antifungal activity was observed for sarasinoside J (**253**).

#### 2.2.7. Meroterpenoids

**Figure 17:**
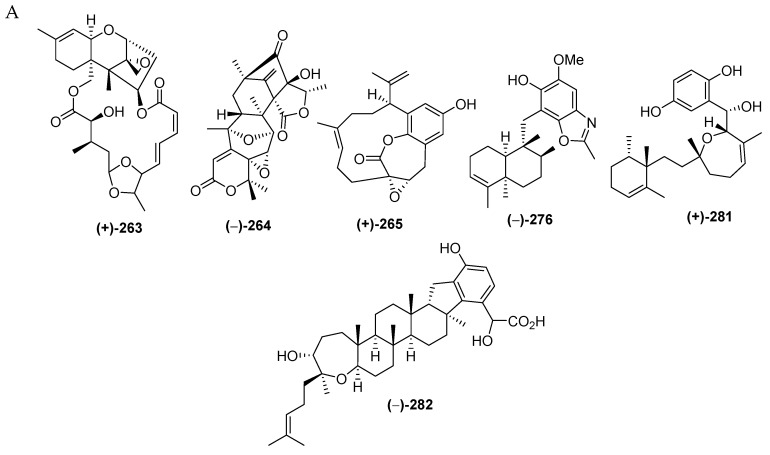

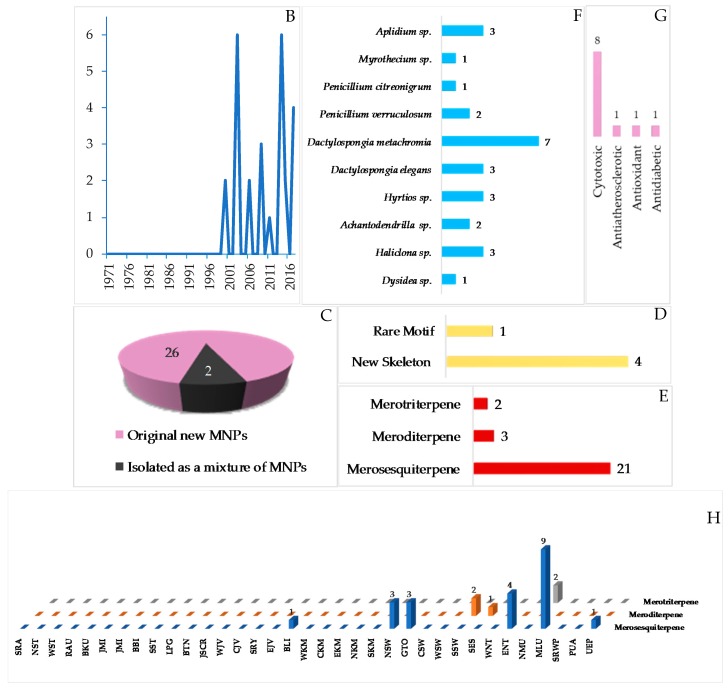
Structures of marine meroterpenoids from Indonesian waters found in 1970–2017: (**A**) representative. Distribution of new marine meroterpenoids by year (**B**). Statistics of new marine meroterpenoids (**C**). Distribution of new marine meroterpenoids on the basis of their skeletons (**D**,**E**), biological sources (**F**), significant biological activity (**G**), and biogeography (**H**).

Marine meroterpenoids, comprised of 26 pure compounds and 2 mixtures, have been discovered since 2000 (Table S7 and Figure 17A–C, Figure S7). Marine meroterpenoids are composed of various skeletons, as shown in **264**–**265**, **281** and **282**, while a rare motif is exhibited by **276**. The majority of marine meroterpenoids found in Indonesian waters are the 21 merosesquiterpenoids, followed by the 3 meroditerpenoids and the 2 merotriterpenoids (Figure 17E). X-ray crystallography was used for two molecules, **264**–**265**, to determine their ACs, while ECD spectra were used to elucidate the ACs of **261**–**262**. The marine meroterpenoids were isolated from the phyla Ascomycta (4 molecules), Porifera (19 molecules), and Chordata (3 molecules). More specifically, 19 molecules from Porifera were found in 3 families: Dysideidae (2 molecules), Chalinidae (3 molecules), and Dictyodendrillidae (2 molecules). With regard to their biological activities, 8 molecules showed significant cytotoxicity, followed by 1 molecule as antiatherosclerotic and 1 molecule as antioxidant and antidiabetic (Figure 17G). The marine merosesquiterpeoids were isolated from specimens from MLU (9 molecules), while meroditerpenoids (2 molecules) and merotriterpenoids (2 molecules) were found from SSW and NMU, respectively (Figure 17H). 

### 2.3. Alkaloids

The alkaloids from Indonesian marine sources are comprised of 260 new molecules, 5 revised molecules (**332a**, **333a**, **348a**, **403a**, and **404a**), 3 derivatives (**328c**, **452c** and **453c**), and 3 molecules that were known, but had been isolated for the first time as natural products (**397** and **546**–**547**). Of these, 102 and 91 molecules were isolated as freebases and salt forms, respectively, and 7 as mixtures. The total of 260 Indonesian marine alkaloids can be grouped into 48 piperidines, 7 pyridines, 37 indoles, 13 acridines, 11 quinolines or isoquinolines, 38 tyrosines, 31 pyrroles, 13 imidazoles, 14 polysulfur aromatics, 15 serines, and 33 others. The last group is comprised of a pyrroloimino-quinone, 5 ceramides, 15 tetramic acids, 2 nucleosides, 2 formamides, a polycyclic diamine, a pterin alkaloid, 2 thiadiazoles, a pyrazole, an azirine, and 2 simple amines/amides. Purification of alkaloids is often challenging due to the presence of nitrogen atoms and their behavior as a base. To tackle this problem, researchers often adjust the pH of mobile phases of chromatography by adding formic acid or trifluoroacetic acid, or modify the stationary phase into a suitable one. Because of their basic nature, many alkaloids are tested as salt forms. Significant activity was observed for 118 molecules consisting of cytotoxic (49), antibacterial (29), anticancer without cytotoxicity (1), antiparasitic (9), antifungal (8), immunosuppresive (2 molecules), antiviral, anti-cystic fibrosis, immunomodulatory, anti-neurodisease, and electrophysiological activity (each 1 molecule).

#### 2.3.1. Piperidine Alkaloids

**Figure 18:**
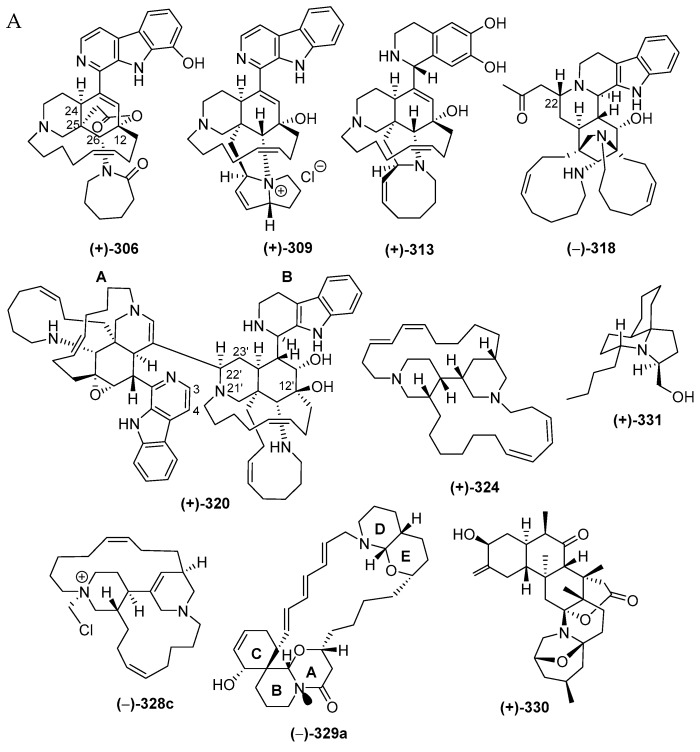

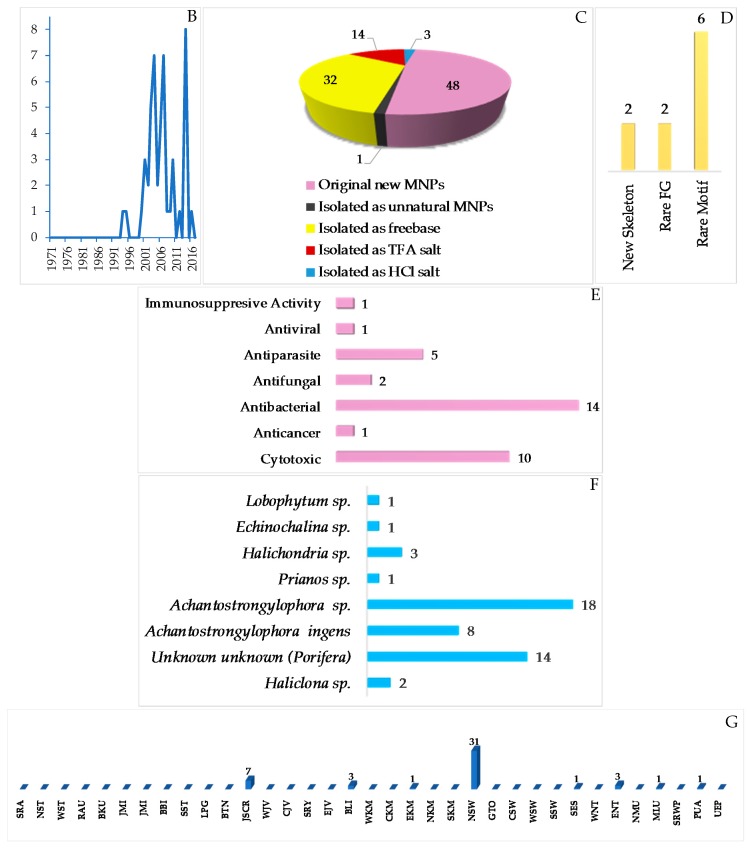
Structures of marine piperidine alkaloids from Indonesian waters found in 1970–2017: (**A**) representative. Distribution of new marine piperidines by year (**B**). Statistics of new marine piperidines (**C**). Distribution of new marine piperidine molecules on the basis of their skeletons (**D**), significant biological activity (**E**), biological sources (**F**), and biogeography (**G**).

The majority of piperidine alkaloids are manzamine-related molecules characterized by the presence of a unique polycyclic ring system. The molecules are compiled in Table S8 in Supplementary Materials and are drawn as in Figure 18A, Figure S8. The first piperidine alkaloid found in Indonesian waters was halicyclamine A (**322**), from a *Haliclona* sponge, in 1994, followed by more alkaloids after 2001 (Figure 18B). As mentioned earlier in Figure 4C, piperidine alkaloids constitute one of the dominant groups of Indonesian MNPs, with 48 molecules, which were classified into 33 manzamines, **284**–**286**, **297**–**302**, **309**–**312**, and **318**–**321**, 2 degraded β-carbolines, **313** and **314**, 3 ircinal-related molecules, **315**–**317**, 6 molecules with two piperidines, **322**–**327**, one fused piperidine-pyran, **329a**, one zoanthamine, **330**, and 2 molecules with a piperidine in a tricyclic system, **331**–**332**. Inspection of the chemical structures allowed us to identify new skeletons in **313** and **318**, rare functional groups in **309** and **329a**, and 6 molecules with rare structural motifs in **306**–**308**, **311** and **320**–**321** (Figure 18D). X-ray analysis was used to reveal ACs of molecules **289**, **324** and **327**. ACs of three **306**, **313** and **328c** were determined by comparing experimental and calculated ECD spectra, while three others, **290**, **293** and **330**, were elucidated with modified Mosher’s method. In terms of their biological activities, significant antibacterial (14), cytotoxic (11), anticancer without cytotoxicity (1), antiparasitic (5), antifungal (5), antiviral and immunosuppresive activity (each 1 molecule) were observed (Figure 18E). The sources of piperidine alkaloids are Porifera (45), Cnidaria (1), and Chordata (2 molecules). In total, 48 molecules were isolated from specimens of NSW (31), JSCR (7), ENT, BLI (3), and SES, NMU, PUA (1 molecule each) (Figure 18G).

**Figure 19:**
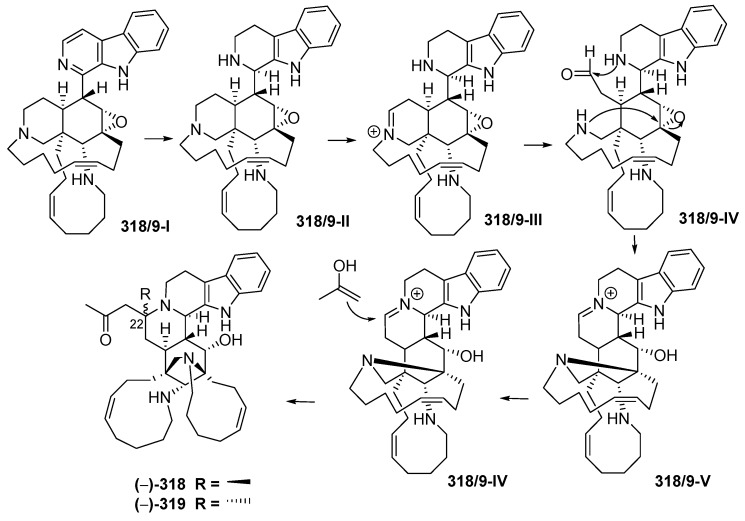
Plausible biosynthetic pathway of (–)-manadomanzamines A **318**, B **319**.

Manadomanzamines A (**318**) and B (**319**) (Figure 18A, Figure S8), obtained from a sponge *Acanthostrongylophora* sp. collected off Manado, have a novel skeleton [73]. Their ACs and conformation were determined by ECD, NOESY and molecular modelling analysis. Molecules **318** and **319** showed growth inhibition against HIV-1 and against fungi causing opportunistic infection with *acquired immune deficiency syndrome (AIDS)*. Biosynthesis of **318** and **319** is proposed, as shown (Figure 19).

#### 2.3.2. Pyridine Alkaloids 

**Figure 20:**
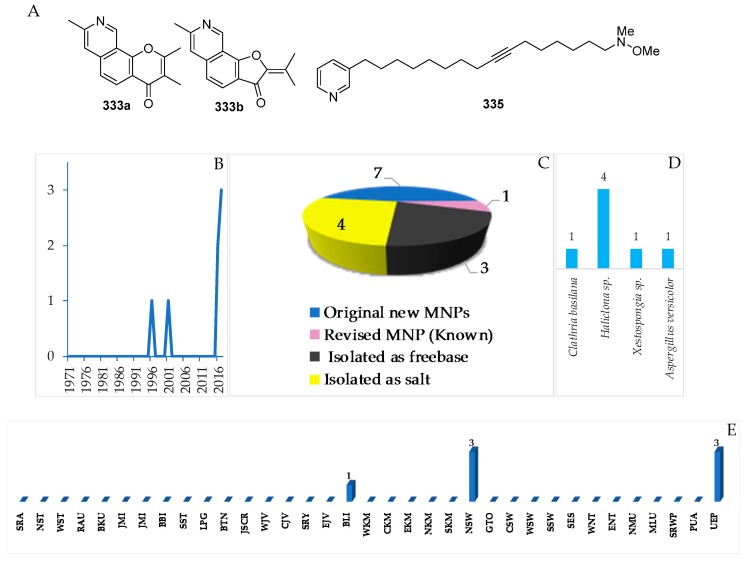
Structures of marine pyridine alkaloids from Indonesian waters found in 1970–2017: (**A**) representative. Distribution of new alkaloids by year (**B**). Statistics of new marine pyridine molecules (**C**). Distribution of new marine pyridine molecules on the basis of biological sources (**D**), and biogeography (**E**).

Only 7 molecules were classified as pyridine alkaloids, as shown in Table S9 and in Figure 20A–C, Figure S9. The molecules were reported recently, in 2016–2017 (Figure 20B). One molecule, **333a**, was revised to the known **333b** after comparing NMR data of total synthesis work [74]. The incorrect structure of **333a** was due to the misinterpretation of HMBC signals.

#### 2.3.3. Indole Alkaloids

**Figure 21:**
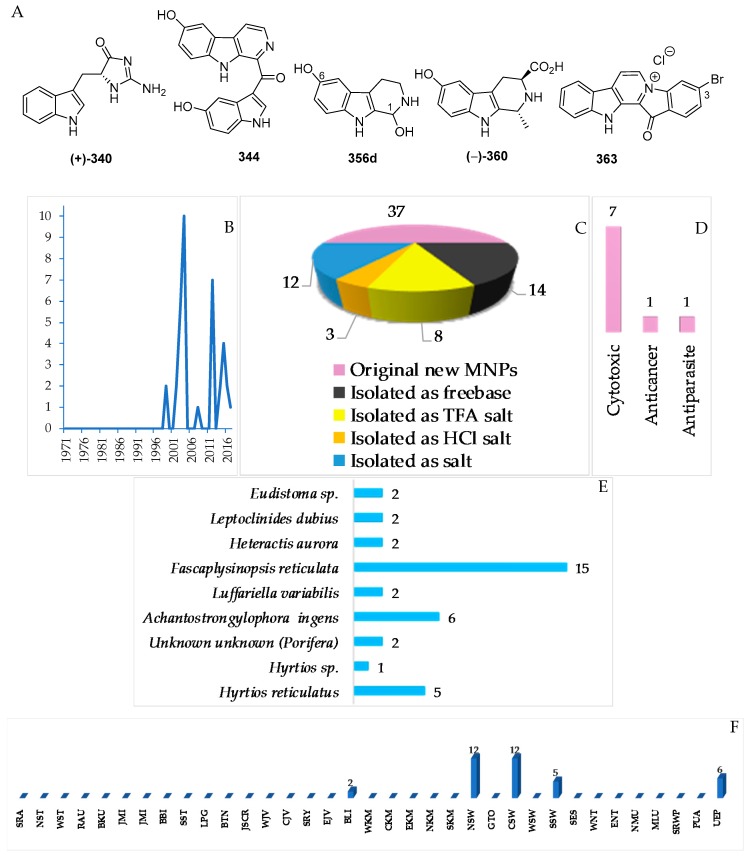
Structures of marine indole alkaloids from Indonesian waters found in 1970–2017: (**A**) representative. Distribution of new marine indole alkaloids by year (**B**). Statistics of new marine indole alkaloids (**C**). Distribution of new marine indole alkaloids, biological activity (**D**), biological sources (**E**), and biogeography (**F**).

There have been 37 new and 1 proposed **356d** indole alkaloids in the Indonesian MNPs (Table S10, Figure 21A–C, Figure S10). ECD was used to determine ACs of (–)-**360**. In addition, the ACs of two indoles, **340** and **341**, were determined by total synthesis. Cytotoxicity was the dominant biological activitiy reported for indole alkaloids (7), followed by antiparasitic and anticancer activity (1 molecule each). The alkaloids were found to be from Porifera (31), Cnidarian (2) and Chordata (4 molecules).

#### 2.3.4. Acridine Alkaloids

**Figure 22:**
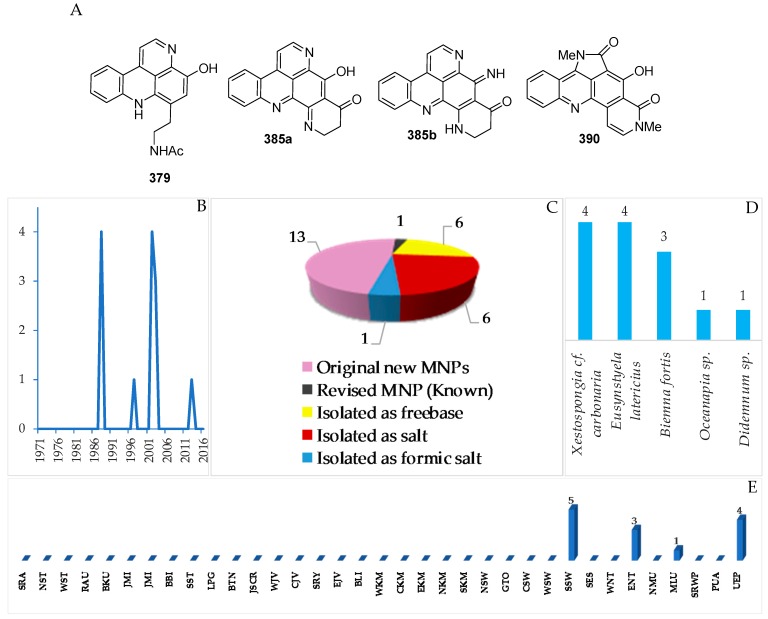
Structures of marine acridine alkaloids from Indonesian waters found in 1970–2017: (**A**) representative. Distribution of new marine acridine alkaloids by year (**B**). Statistics of new marine acridine molecules (**C**). Distribution of new marine acridine-containing molecules on the basis of their biological sources (**D**), and biogeography (**E**).

In total, 13 new and 1 revised acridine alkaloids were reported from Indonesian waters (Figure 22A–C, Figure S11 and Table S11). Of 13 molecules, six were isolated as free bases, while the remaining seven were obtained as salts. One molecule, **385a**, has been revised to **385b**. A new skeleton was proposed for **390**. Research on marine acridines began with the discovery of styelsamines A–D (**378**–**381**) in 1988 (Figure 22B). The structure of the alkaloid **390** was determined by X-ray analysis. Six molecules showed significant cytotoxicity. The acridine alkaloids were isolated from Porifera (8) and Chordata (5 molecules).

**Figure 23:**
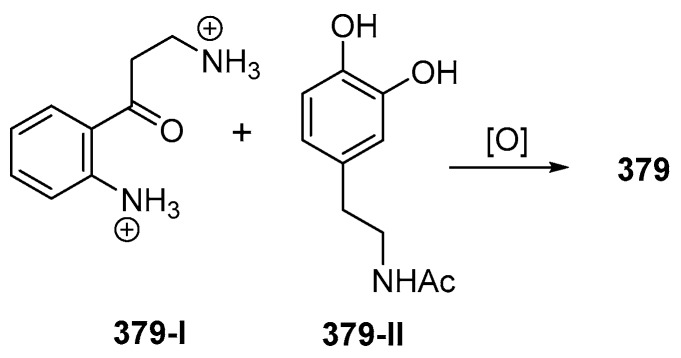
Biomimetic synthesis of styelsamine B (**379**) from kynuramine (**379-I**) and *N*-acetyl dopamine (**379-II**).

The ascidian *Eusynstyla latericius*, collected off Makassar, SSW, was found to contain styelsamines A–D (**378**–**381**, Figure 23A, Figure S11) which showed cytotoxic effect against HCT116 cells [75]. A biomimetic synthesis of styelsamine B (**379**) was conducted from kynuramine (**379-I**) and *N*-acetyl dopamine (**379-II**) (Figure 23) featuring a CeCl_3_-catalyzed oxidative coupling of **379-I** and **379-II** in the presence of silver oxide [76]. The structure of styelsamine C (**380**) was also confirmed by a total synthesis utilizing biaryl Suzuki cross-coupling [77]. The preparation of **379** and **381** was also achieved by using a simple biomimetic synthetic method [78]. Styelsamine D (**381**) could be a biosynthetic intermediate for a large subset of pyridoacridine alkaloids [79]. Styelsamines B (**379**) and D (**381**) showed high affinity to calf thymus DNA [78]. 

#### 2.3.5. Quinoline and Isoquinoline Alkaloids

**Figure 24:**
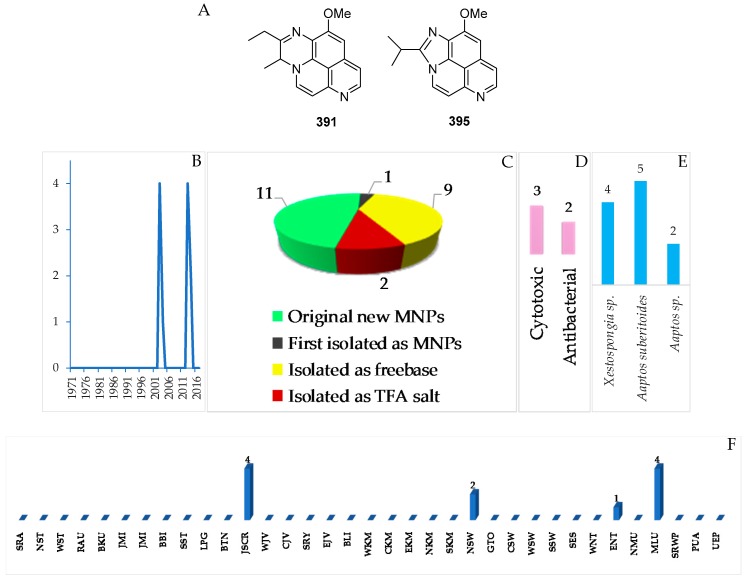
Structures of marine quinoline or isoquinoline alkaloids from Indonesian waters found in 1970–2017: (**A**) representative. Distribution of the alkaloids by year (**B**). Statistics of new marine quinoline or isoquinoline alkaloids (**C**). Distribution of new marine quinoline and isoquinoline alkaloids on the basis of their biological activity (**D**), biological sources (**E**), and biogeography (**F**).

In total, 11 natural quinoline or isoquinoline alkaloids were discovered in Indonesian waters (Figure 24A–C, Figure S12, Table S12). The very first member of this group is an aaptamine derivative obtained off Jakarta, JSCR (Figure 24B). Of 11 molecules, nine were isolated as free bases, while two were obtained as salts. Two molecules, **391** and **395**, of the class contained a rare motif. With respect to biological activity, 3 molecules showed significant cytotoxicity, while 2 molecules were antibacterial (Figure 24D). All the compounds were found from the marine sponges *Aaptos suberitoides* (5), *Aaptos* sp. (2) and *Xestospongia* sp. (4 molecules) (Figure 24E). JSCR (4 molecules) and MLU (4 molecules) were the major source areas of the group (Figure 24F).

#### 2.3.6. Tyrosine Alkaloids

**Figure 25:**
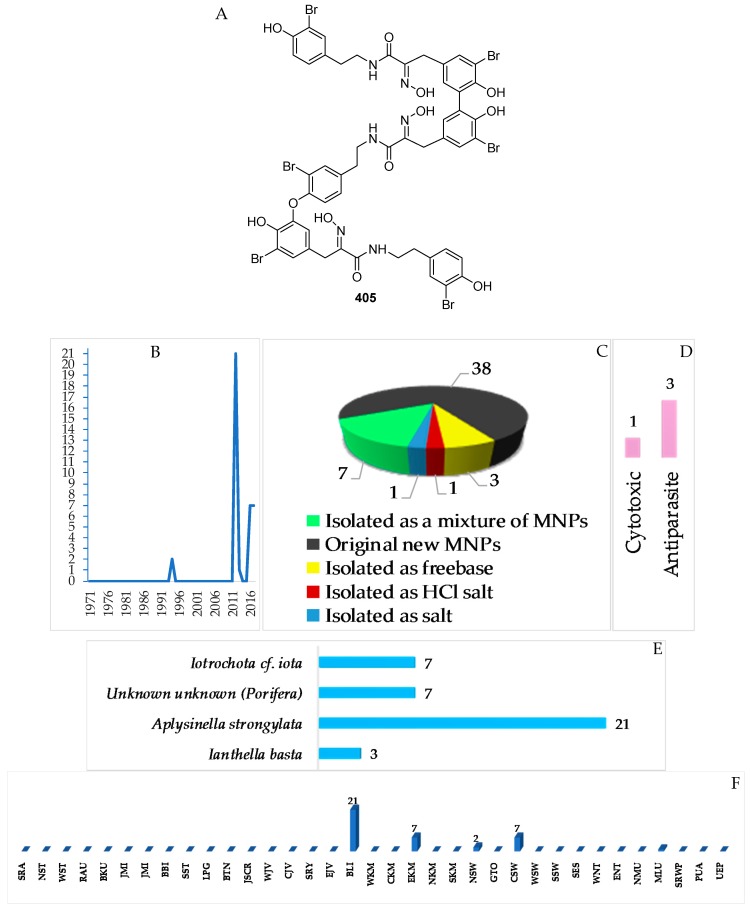
Structures of marine tyrosine alkaloids from Indonesian waters in 1970–2017: (**A**) representative. Distribution of the alkaloids by year (**B**). Statistics of new tyrosine alkaloids (**C**). Distribution of new marine tyrosine-containing alkaloids on the basis of their, biological activity (**D**), biological sources (**E**), and biogeography (**F**).

A total of 38 tyrosine alkaloids were found in Indonesian waters. Of 38 molecules, 7 molecules were isolated as mixtures (Figure 25A–C, Figure S13, Table S13). The first member of this class was bastadin reported in 1994. The chemical structures of this class contained 1 new skeleton **405**. With respect to biological activity, antiparasitic activity was seen for 3 molecules followed by one cytotoxic molecule (Figure 25E). The sources of tyrosine alkaloids are Porifera consisting of 3 species and 1 undescribed species (Figure 25F). Most molecules were found from specimens of BLI (21), EKM (7), CSW (7), and NSW (2 molecules) (Figure 25G). 

#### 2.3.7. Pyrrole Alkaloids

**Figure 26:**
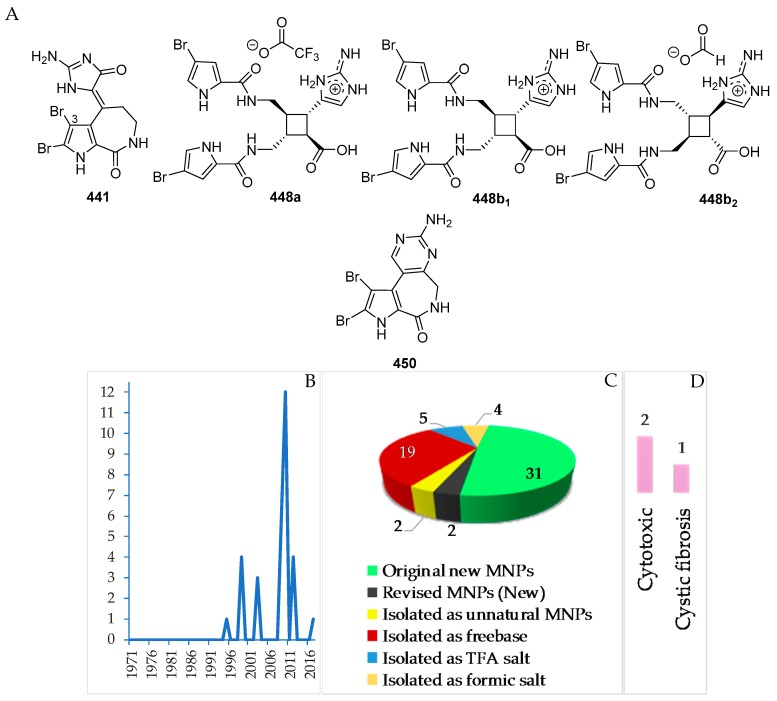

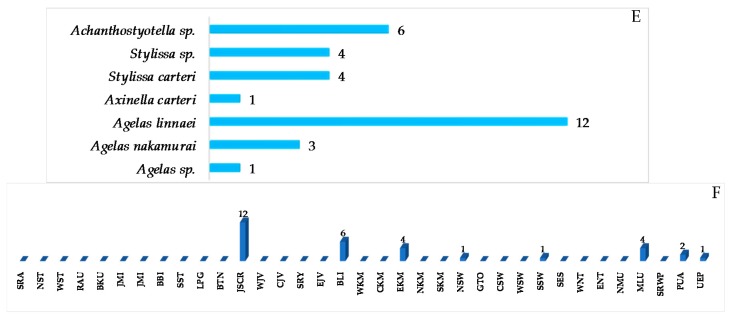
Structures of marine pyrrole alkaloids from Indonesian waters found in 1970–2017: (**A**) representative. Distribution of the alkaloids by year (**B**). Statistics of new pyrrole alkaloids (**C**). Distribution of new marine pyrrole-containing alkaloids on the basis of their, biological activity (**D**), biological sources (**E**), and biogeography (**F**).

A total of 31 pyrrole alkaloids have been discovered from the Indonesian waters in addition to 2 revised and 2 derivatives (Figure 26A–C, Figure S14 and Table S14). Nineteen molecules out of 31 were isolated as free bases, while 9 were isolated as salts. The first member, **441**, was discovered in 1995, and 12 molecules were reported in 2010 (Figure 26B). This class of alkaloids contains 1 new skeleton in **450** and 1 rare motif in **448**. The ACs of two molecules, **443** and **448b_2_**, of this class were determined by ECD spectra, while 1 molecule, **450**, was confirmed by total synthesis. Cytotoxicity (2 molecules) and anti-cystic fibrosis (1 molecule) are the signature of significant biological activity of the pyrrole alkaloids (Figure 26E). The sources of pyrrole alkaloids are exclusively Porifera, consisting of 3 families (Agelasidae 22 molecules, Axinellidae 1 molecule, and Scopalinidae 8 molecules). 

**Figure 27:**
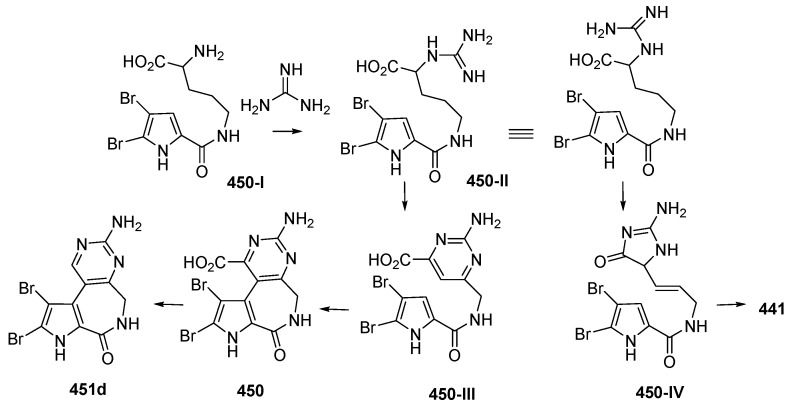
The plausible biosynthetic pathway of latonduines A **450**, B **451d**, and (*Z*)-3-bromohymenialdisine **441**.

Two dimeric bromopyrrole alkaloids, nakamuric acid (**448a**) and its methyl ester, **449**, showing antibiotic activity against *B. subtilis*, were isolated from the sponge *Agelas nakamurai*, collected in MLU [80]. A total synthesis of (9*R*,10*S*,9′*R*,10′*S*)-nakamuric acid (**449a**) was accomplished by the minimal use of protective groups with exploration of 2-aminoimidazole [34]. The AC of **448a** was established to be (9*S*,10*R*,9′*S*,10′*R*) by comparison of the experimental and calculated ECD spectra [35]. Thus, **448a** was proved to be an enantiomer of the synthetic one. The sponge *Stylissa carteri* from SSW was found to contain two unprecedented molecules, latonduines A (**450**) and B (**451d**) [45]. Their structures were elucidated by analysis of spectroscopic data and confirmed by total synthesis of **450**. It is proposed that ornithine is the biogenetic precursor to the aminopyrimidine fragment, as shown in Figure 27. 

#### 2.3.8. Imidazole Alkaloids

**Figure 28:**
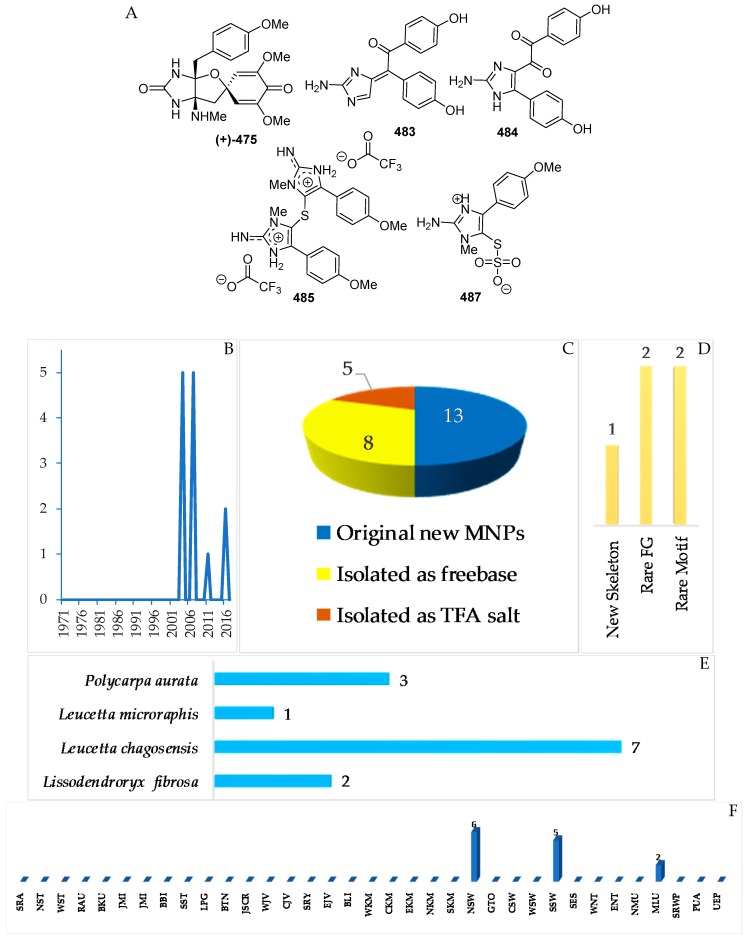
Structures of marine imidazole alkaloids from Indonesian waters found in 1970–2017: (**A**) representative. Distribution of the alkaloids by year (**B**). Statistics of imidazole alkaloids (**C**). Distribution of new marine imidazole alkaloids on the basis of their chemical skeletons (**D**), biological sources (**E**), and biogeography (**F**).

The marine sponges *Lissodendroryx fibrosa*, *Leucetta chagosensis*, and *Leucetta microraphis*, as well as the ascidian *Polycarpa aurata* (Figure 28E), are the sources of 13 imidazole alkaloids, which were isolated as free bases (8) and salts (5 molecules) (Figure 28A–C, S 15, Table S15). Compound **483** retains 1 new skeleton, while four compounds, **475**, **484**, **485**, and **487**, have rare structural motifs (Figure 28D). With regard to biological activity, significant antifungal (1) and cytotoxic activity (3 molecules) was found (Figure 28E). The sources of imidazole alkaloids were collected at 3 regions (NSW, 6; SSW, 5; MLU, 2 molecules). 

**Figure 29:**
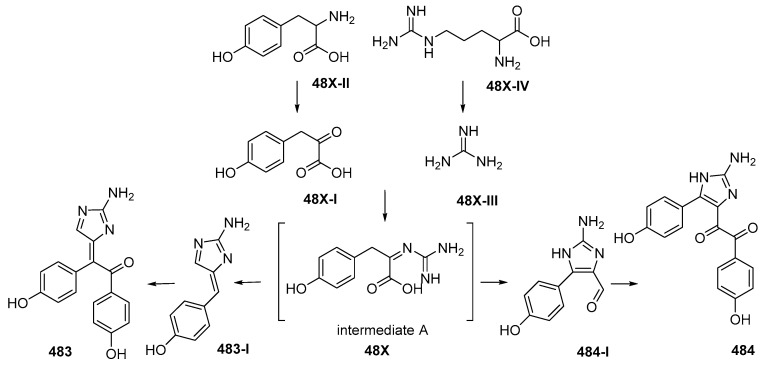
The plausible biosynthetic pathway of lissodendrins A **483** and B **484**.

Lissodendrins A (**483**) and B (**484**) (Figure 28A, Figure S15), 2-amino imidazole alkaloids, were isolated from the sponge *Lissodendoryx* (*Acanthodoryx*) *fibrosa* collected off Ambon, MLU [81]. The latter compound contains a (*p*-hydroxyphenyl)glyoxal moiety as an unprecedented skeleton. A plausible biosynthetic scheme for these compounds was proposed, as in Figure 29 [81].

#### 2.3.9. Polysulfur Aromatic Alkaloids 

**Figure 30:**
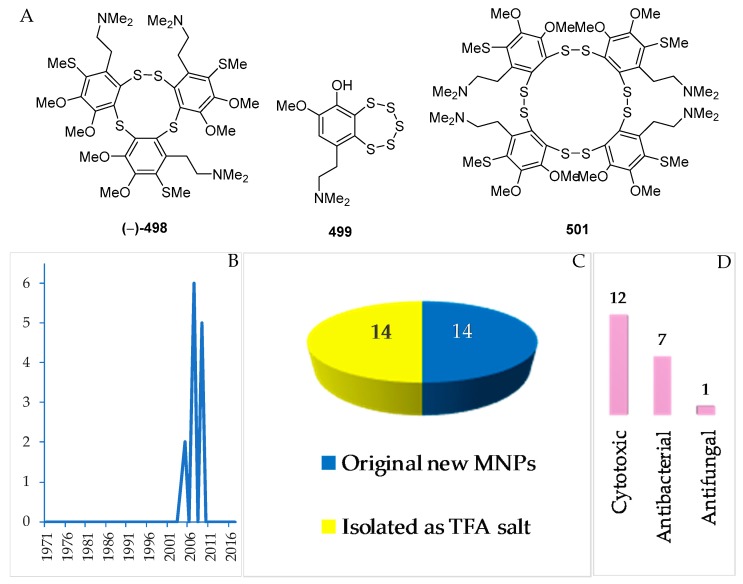
Structures of marine polysulfur aromatic alkaloids from Indonesian waters found in 1970–2017: (**A**) representative. Distribution of the alkaloids by year (**B**). Statistics of polysulfur aromatic alkaloids (**C**). Distribution of new marine polysulfur aromatic-containing alkaloids on the basis of their biological activity (**D**).

Polysulfur aromatic alkaloids are an unusual class of MNPs. To date, there have been 14 MNPs isolated as salts (Figure 30A–C, Figure S16, and Table S16). The polysulfur aromatic alkaloids were reported in 2005 (3), 2007 (6), and 2009 (5 molecules) (Figure 30B). The first discovery of this group was lissoclibadin 1 (**498**) [82]. Three molecules, **498**, **499** and **501**, of polysulfur aromatic alkaloids retain new skeletons. With respect to biological activity, significant cytotoxic (12), antibacterial (7), and antifungal activity (1 molecule) was reported (Figure 30D). All the molecules were found from *Lissoclinum* cf. *badium* collected in NSW. 

#### 2.3.10. Serine-Derived Alkaloids 

**Figure 31:**
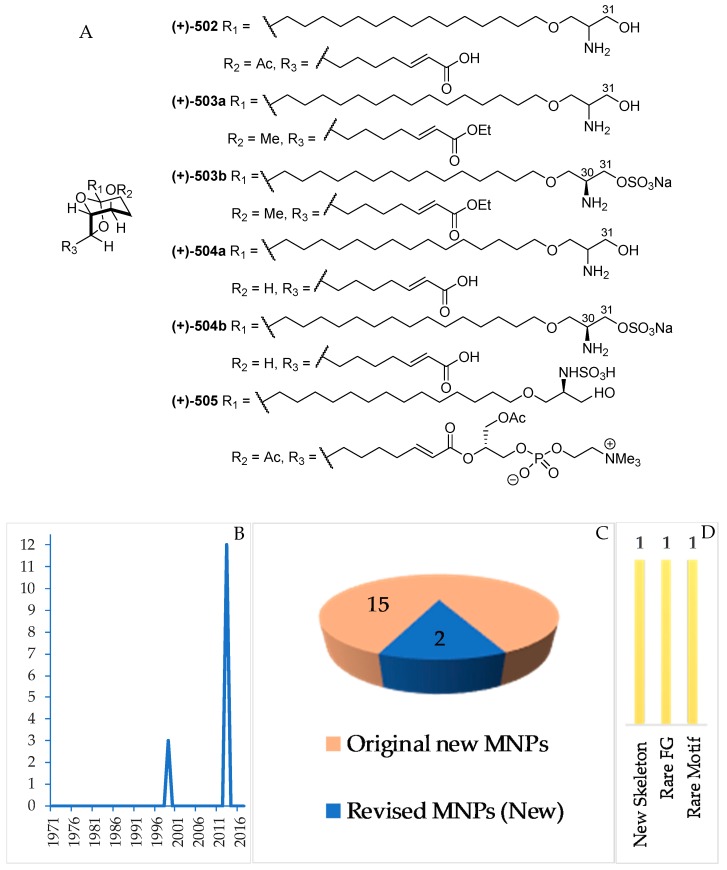

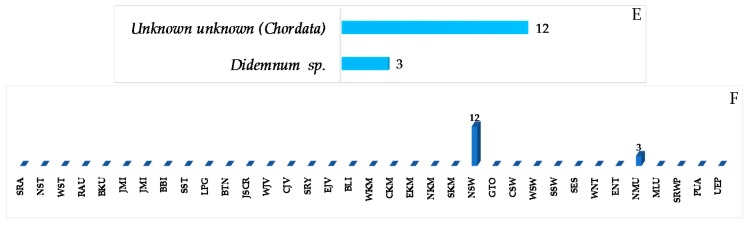
Structures of marine serine-containing alkaloids from Indonesian waters found in 1970–2017: (**A**) representative. Distribution of the alkaloids by year (**B**). Statistics of the alkaloids (**C**). Distribution of marine serine-containing alkaloids on the basis of their chemical skeleton (**D**), biological source (**E**), and biogeography (**F**).

A total of 15 serine-derived alkaloids have been discovered (Figure 31A–C, Figure S17, Table S17). Among them, 2 molecules, **503a** and **504a**, were revised to **503b** and **504b**. All the alkaloids share a common structural feature: 6,8-dioxabicyclo[3.2.1]octane (6,8-DOBCOs). One of them, **502**, possesses a new skeleton. The first discovery in this group was reported in 1999 (3 molecules), which was followed by another 12 molecules before 2013 (Figure 31B). The ACs were determined for **505** by ECD and for **503b**, **504b** and **505** by total synthesis. With regard to biological activities, significant anticancer activity without cytotoxicity was reported for 4 molecules. Tunicates were proved as the sole source of this class of MNPs. The majority of the molecules were isolated from specimens collected in NSW (12) and NMU (3 molecules).

#### 2.3.11. Other Marine Alkaloids

**Figure 32:**
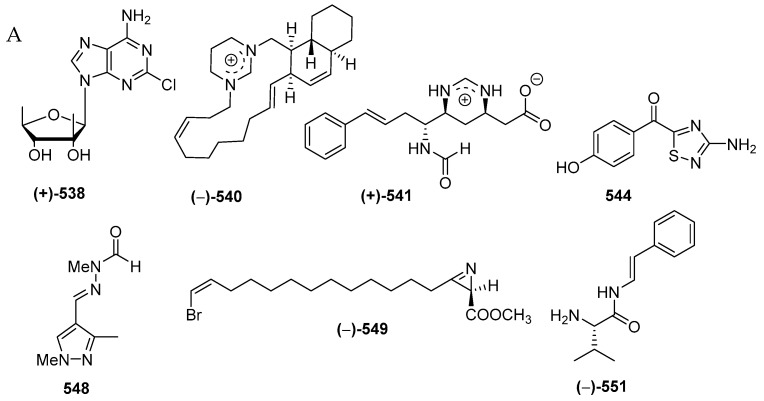

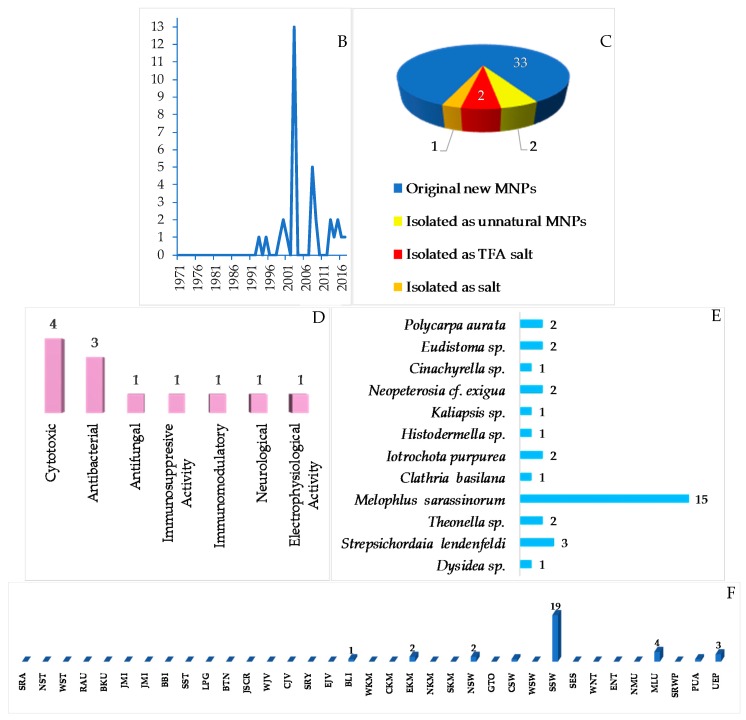
Structures of other marine alkaloids from Indonesian waters found in 1970–2017: (**A**) representative. Distribution of other marine alkaloids by year (**B**). Statistics of other marine alkaloids (**C**). Distribution of other marine alkaloids on the basis of biological activity (**D**), biological source (**E**), and biogeography (**F**).

A total of 33 alkaloids do not belong to the above structural classes. They are comprised of a pyrroloimino-quinone, five ceramides, 15 tetramic acids, two nucleosides, two formamides, one polycyclic diamine, one pterin, two thiadiazoles, one pyrazole, one azirine, two simple amine/amide alkaloids (2 molecules) (Figure 32A–C, Figure S18, Table S18). Of 33 molecules, seven, **538**, **540**, **541**, **544**, **548**, **549**, and **551**, contain rare structural motifs. ACs of three molecules, **524**, **525** and **538**, were determined by ECD. With regard to biological activities, cytotoxicity and antibacterial activity were seen in 4 and 3 molecules, respectively, whereas antifungal, immunosuppresive, immunomodulatory, neurological, and electrophysiological activity were shown in one molecule each (Figure 32D). The sources of the molecules are 10 sponge species and 2 tunicates (Figure 32E). The majority of the alkaloids were found from specimens from SSW (19), MLU (4), NSW (2), EKM (2), and BLI (1 molecule) (Figure 32F).

Bioorganic studies of melophlin A (**523)** to dynamins II and I-like proteins in cells, thereby modulating signal transduction through the Ras network, was conducted by using a surface plasmon resonance (SPR) [83]. Furthermore, Mg or Zn complexes of **523** are antiproliferative in various cancer cells, while they are less toxic to normal fibroblasts. The complexes dissolve more in water than Ca analogue [84]. Melophlin A (**523**) also exhibited anti-dormant mycobacterial activity [85] and cytotoxicity against L1210 cells [86]. The influence of **523** on the colony formation of Chinese hamster V79 lung cells and of the production of interleukin (IL)-8 in phorbol myristate acetate (PMA)-stimulated HL60 cells were examined [87].

**Figure 33:**
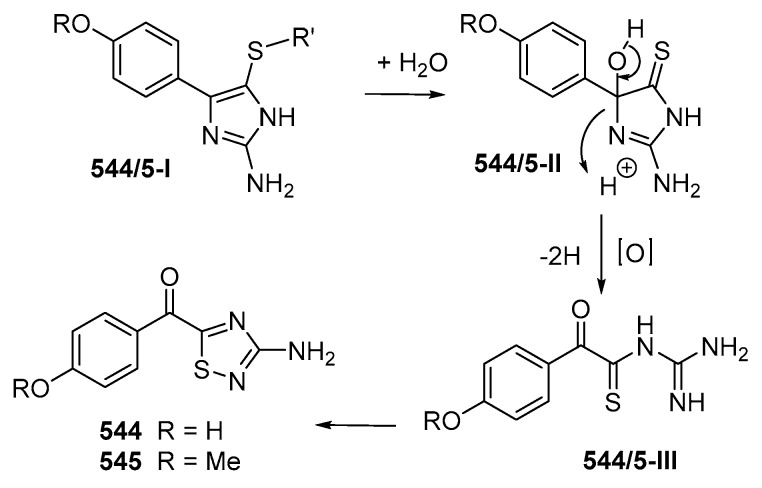
Plausible biosynthetic relation of polycarpathiamines A **544** and B **545**.

Two alkaloids containing an uncommon 1,2,4-thiadiazole ring named polycarpathiamines A (**544**) and B (**545**) (Figure 32B) were isolated from the ascidian *Polycarpa aurata* collected in MLU [46]. The structures of **544** and **545** were elucidated by spectroscopic methods and by synthesis. Polycarpathiamine A (**544**) showed cytotoxicity to L5178Y cells. The biosynthetic relation of **544** and **545** was proposed as shown in Figure 33.

### 2.4. Peptides 

**Figure 34:**
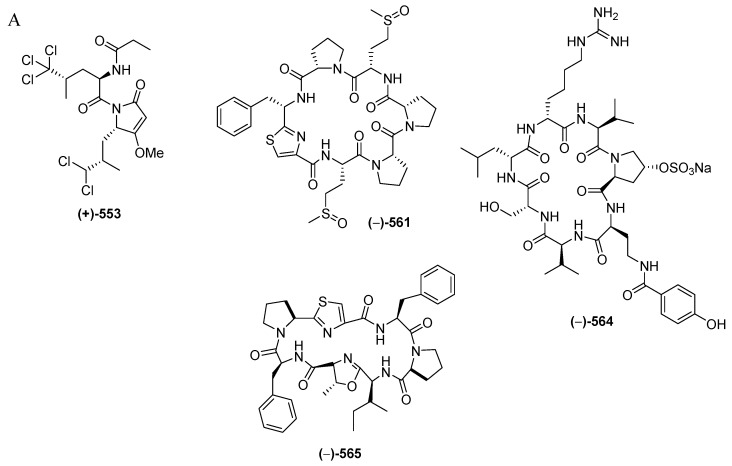

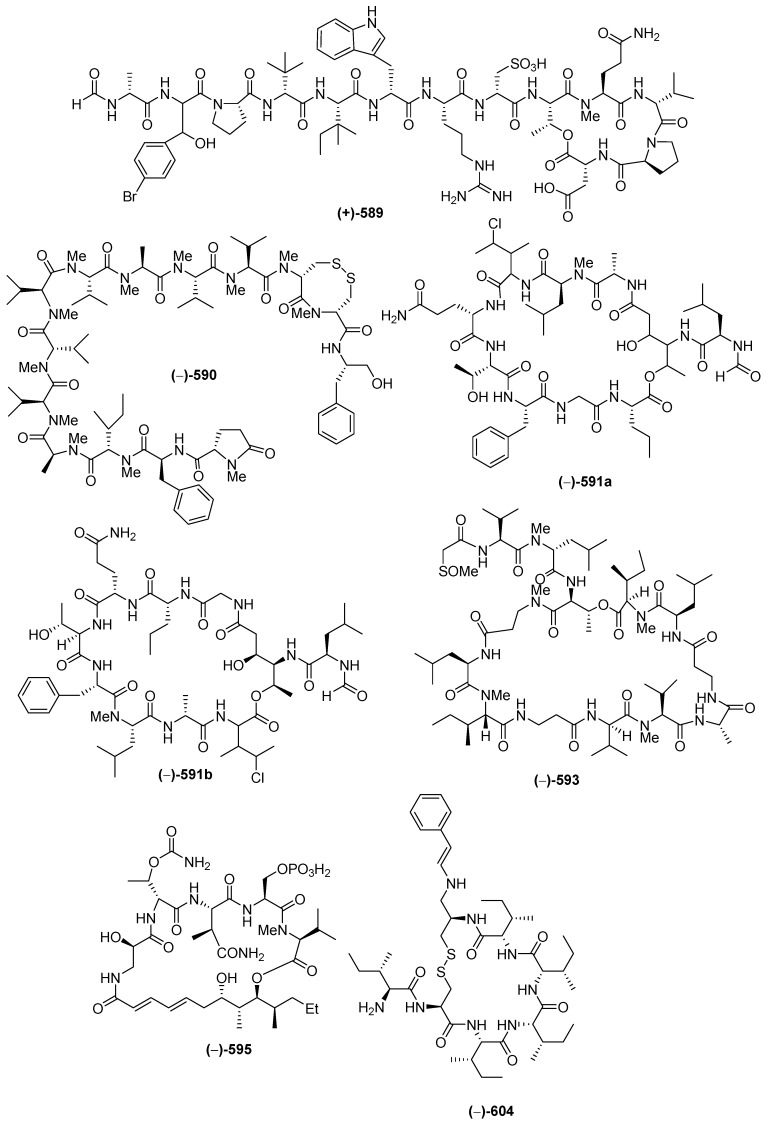

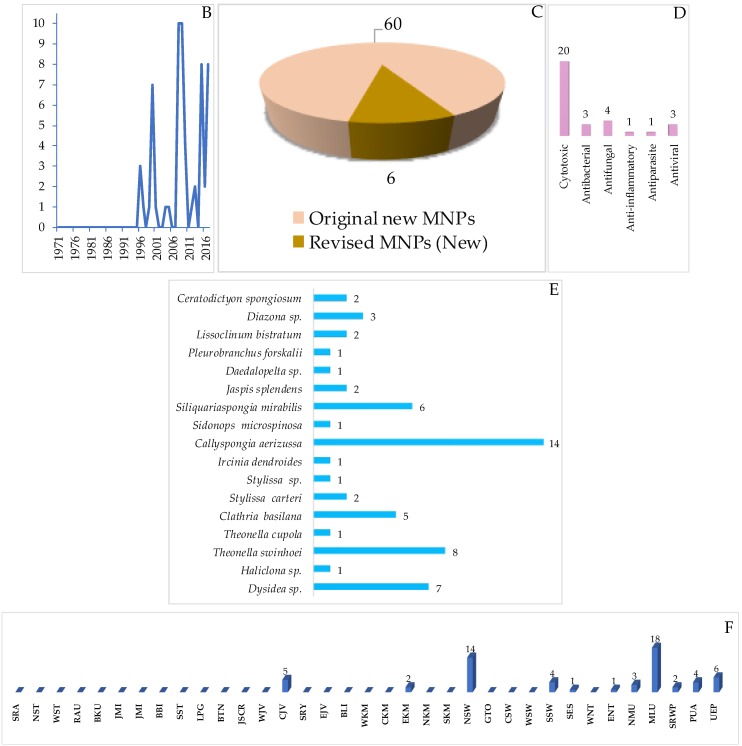
Structures of marine peptides from Indonesian waters found in 1970–2017: (**A**) representative. Distribution of marine peptides by year (**B**). Statistics of marine peptides (**C**). Distribution of marine peptides on the basis of biological activity (**D**), biological source (**E**), and biogeography (**F**).

Marine peptides have emerged as a very important class of bioactive compound in Indonesian MNPs. The class is comprised of 60 natural and 6 revised MNPs (Figure 34A–C, Figure S19, Table S19). The natural peptides can be grouped into: linear dipeptides, **552**–**557**, a linear tridecapeptide, **590**, a cyclotetrapeptide, **608**, two cyclopentapeptides, **604**–**605**, cyclohexapeptides, **558**–**563**, cyclohepta-peptides, **564**–**570**, a cyclooctapeptide, **571**, cycloundecapeptides, **572a**–**575**, cyclopeptides with a linear peptidic chain, **576a**, **577**–**579**, **580a**, **581**, **582b**, **583**–**588**, and **606**–**607**, and cyclodepsipeptide, **591a**–**603**, cyclodepsipeptide with a side chain, **589**, and macrocyclic peptides, **609**–**611**. Nine molecules, **553**, **561**, **564**, **589**, **590**, **591a**, **593**, **595**, and **602**, are categorized as having rare FG and structural motifs. After their first discovery in 1996, the number of Indonesian marine peptides has kept increasing until now. The ACs of peptides (41 molecules) have generally been determined by Marfey analysis. Only 3 peptides, **553**, **565** and **566b**, were solved by X-ray crystallography. With respect to biological activity, 20 peptides were reported to be cytotoxic, followed by 4 with antifungal, 3 each with antibacterial and antiviral, and one each with anti-inflammatory and antiparasitic activities (Figure 34E). Indonesian marine peptides have been found from 4 phyla (Rhodophyta, Porifera, Chordata, and Mollusca). On the biogeography, MLU (18 molecules) and NSW (14 molecules) are the top places for the discovery of Indonesian marine peptides (Figure 34F). 

### 2.5. Fatty Acids and Linear Molecules

**Figure 35:**
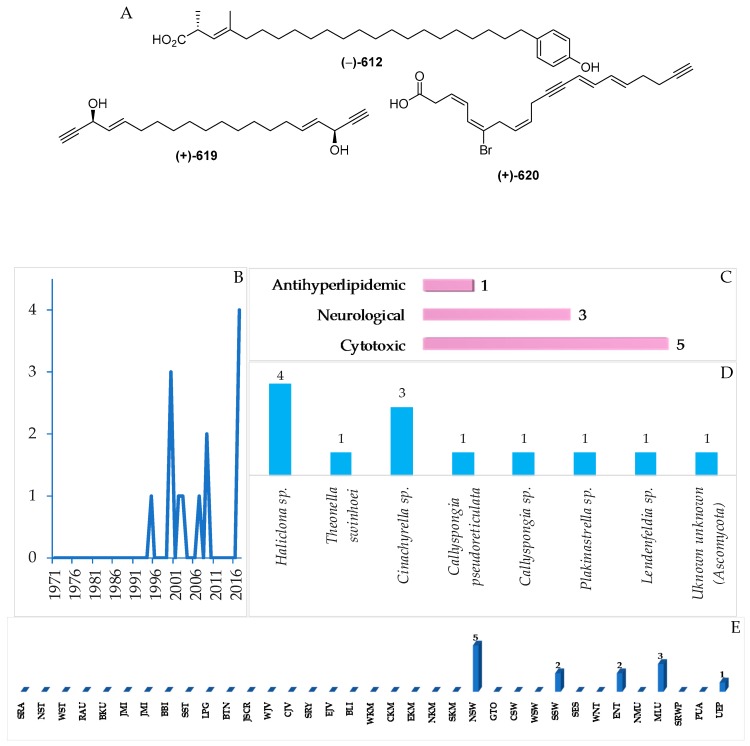
Structures of fatty acids and linear molecules from Indonesian waters found in 1970–2017: (**A**) representative. Distribution of this group of metabolites by year (**B**). Distribution of marine fatty acid on the basis of their biological activity (**C**), biological source (**D**), and biogeography (**E**).

A total of 13 (or 12) Indonesian marine fatty acids or linear molecules, probably biosynthesized through acetate pathways, have been reported, and they were characterized by the presence of double or triple bonds or their combination, ranging from 1 to 7 (Figure 35A, Figure S20, Table S20). After the first discovery of (–)-elenic acid (**612**) in 1995, this group of metabolites has been reported continuously (Figure 35B). Of 13 molecules, one showed cytotoxicity, three showed neurological, and five showed antihyperlipidemic activity (Figure 35D). The ACs of the metabolites were determined by modified Mosher’s method for **615**–**619**, and by total synthesis for **613** and **615**–**617**. With respect to biological sources, 12 molecules were found from Porifera and 1 from Ascomycota (Figure 35E). All the molecules were isolated from specimens collected in NSW (5), MLU (3), SSW and ENT (2 each), and UEP (1 molecule).

### 2.6. Polyketides

**Figure 36:**
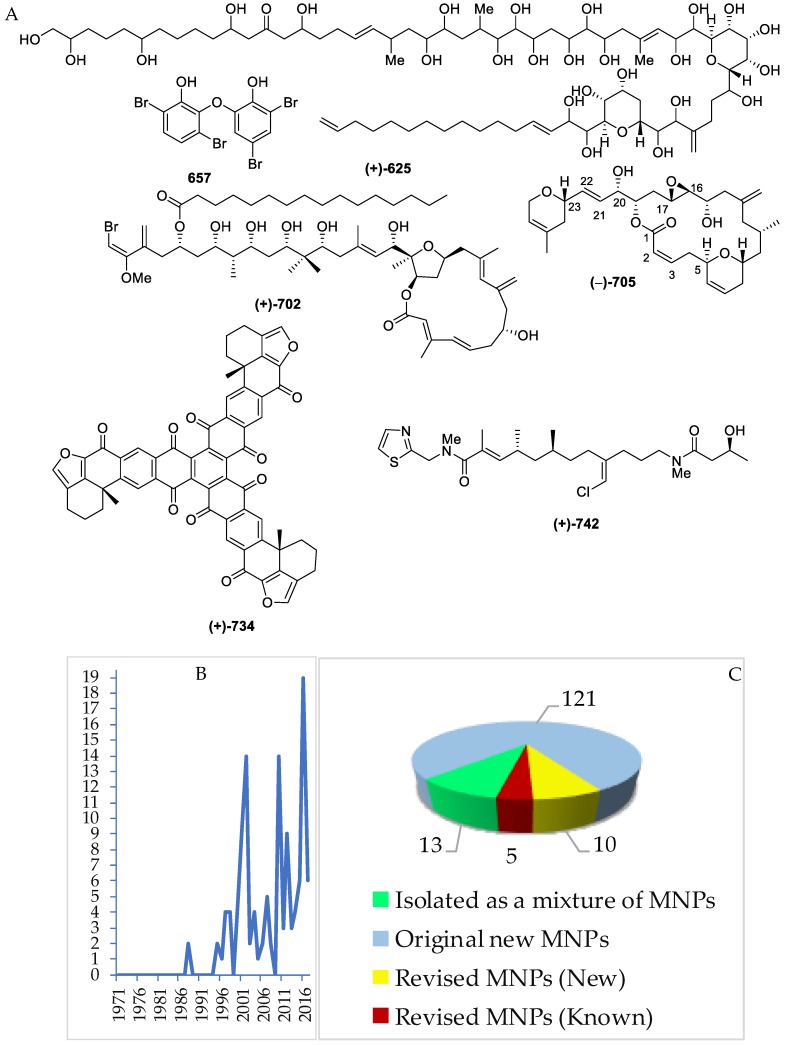

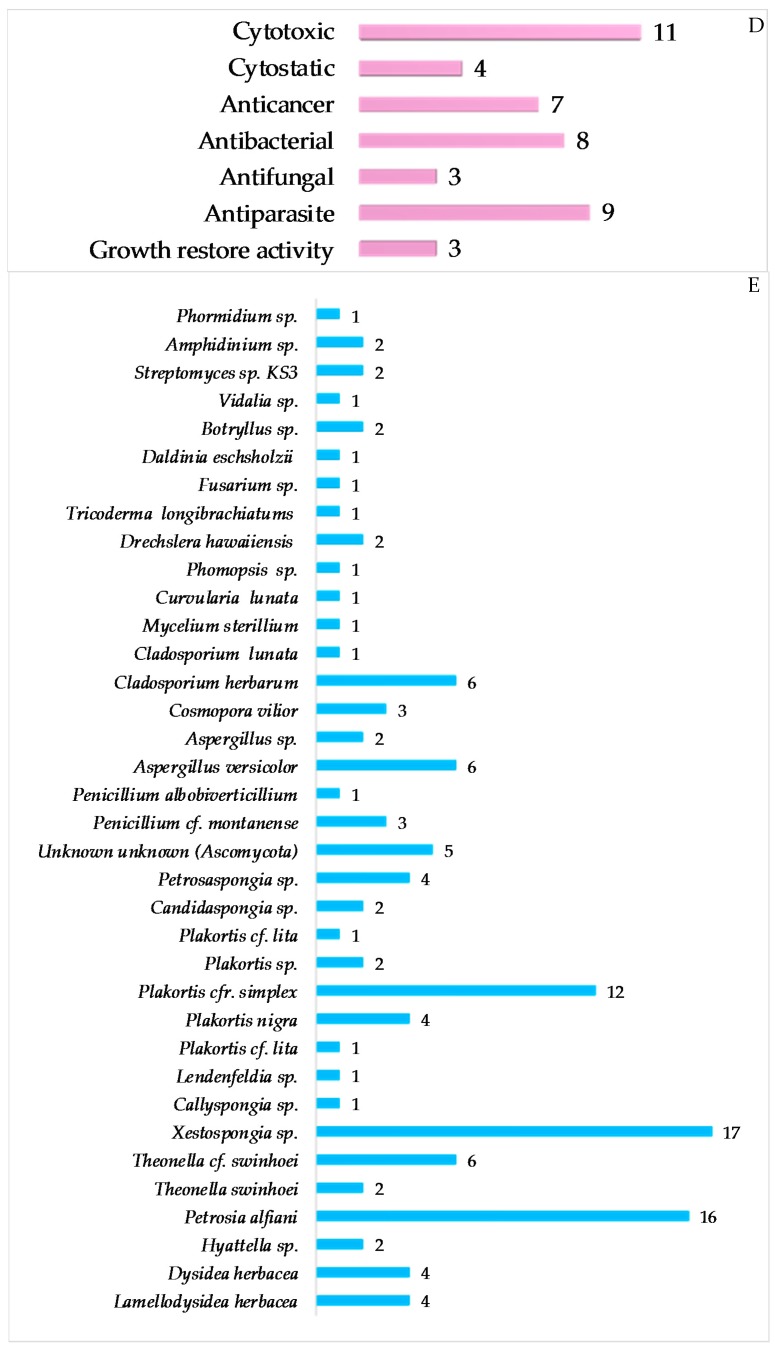

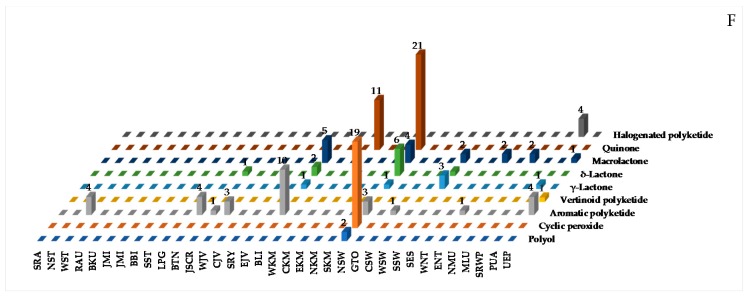
Structures of marine polyketides from Indonesian waters found in 1970–2017: (**A**) representative. Distribution of marine polyketides by year (**B**). Statistics of marine polyketides (**C**). Distribution of marine polyketide on the basis of their biological activity (**D**), biological source (**E**), and biogeography (**F**).

Several polyketides have been discovered in Indonesian waters, particularly since 1995. The molecules can be grouped into 121 natural, 13 mixtures, and 10 revised molecules (Figure 36A–C, Figure S21 and Table S21). Of 121 polyketides, significant biological activities were observed, with 11 cytotoxic, 9 antiparasitic, 8 antibacterial, 7 anticancer without cytotoxicity, 4 cytostatic, and 3 each of antifungal and growth restoring activity (Figure 36D). Polyketides can be classified into 9 different structural groups, consisting of 2 polyols, 18 cyclic peroxides, 34 aromatic polyketides, 1 vertinoid polyketide, 6 γ-lactones, 10 δ-lactones, 16 macrolides, 33 quinones, and 4 halogenated polyketides. Stereo or regiochemistry of four molecules **659b**, **687**–**688** and **705** were determined by X-ray crystallography, while the ACs of 21 molecules were determined by ECD. Seven structures, **659a**, **663a**, **664a**, **689a**, **692a**, **693a**, and **701a**, were revised to **659b**, **663b**, **664b**, **689b**, **692b**, **693b**, and **701b**. In addition, four molecules, **661a**, **662a**, **665a**, **666a**, were revised twice as **661b_1_** to **661b_2_**, **662b_1_** to **662b_2_**, **665b_1_** to **665b_2_**, and **666b_1_** to **666b_2_**. The revision was made by reisolation work, total synthesis, and X-ray analysis. For large molecules, such as **625** and **626**, structure elucidation was aided by chemical degradation. The polyketides were isolated from specimens of 5 phyla: Ascomycota (35 molecules), Ciliophora (2 molecules), Rhodophyta (1 molecule), Porifera (78 molecules), and Chordata (2 molecules).

### 2.7. Carbohydrates

**Figure 37:**
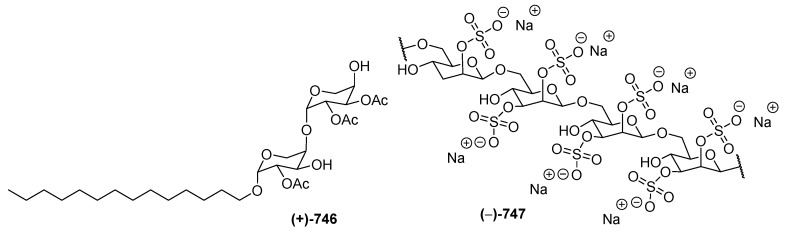
Structures of marine carbohydrates from Indonesian waters in 1970–2017.

One each of carbohydrates **746** and **747** (Figure 37) was isolated from a soft coral *Sinularia* sp. and from the tunicate *Didemnum molle* collected in NSW. Sinularioside (**746**), a triacetylated glycolipid, contains two α-D-arabinopyranoses and a myristyl alcohol [88]. The structure of **747** was solved by interpretation of MS and NMR data, along with ECD analysis of degradation products. Molecule **746** was proved to inhibit LPS-induced nitric oxide (NO) release. A polysaccharide, kakelokelose (**747**), inhibited the proliferation of HIV. Analysis of ^1^H and ^13^C NMR data of the polysaccharide and its desulfated derivative revealed that it consisted of a sequence of 2,3-disulfated mannose units joined through β-1,6 glycosidic linkages.

## 3. Conclusions

Over the past 47 years (from January 1970 to December 2017), 732 new natural products, 4 compounds isolated for the first time as natural products but known previously as synthetic entities, 34 compounds with structural revision, and 9 derivative compounds have appeared in 270 papers. Currently, have been over 29,000 MNPs [89] discovered since the first report of spongothymidine in 1950 [90,91]. Original Indonesian MNPs have largely been found in Porifera, Cnidaria and Chordata, while the global trends are Porifera, Cnidaria, and Ascomycota [89]. In addition to the three phyla, Indonesian MNPs have been found in 94 species, 106 genera, 64 families, 32 orders, and 14 classes. The chemical diversity of Indonesian MNPs has been substantiated on the basis of 28 compounds with new skeletons and 62 molecules with rare structural motifs and FGs, while atomic diversity is manifested in 27 molecules with 6 different atoms in 1 molecule. Of 732 molecules, 373 (51.0%) are nitrogenous. In addition, 122 molecules (16.7%) possessed a ratio of H/C < 1. Of 34 defined provinces, 18 (NST, WST, BTN, JSCR, WJV, CJV, BLI, ENT, EKM, NSW, GTO, CSW, SSW, SES, NMU, MLU, SRWP, and PUA) have been reported as collection sites for new MNPs. Among these, NSW, MLU, SSW, BLI, and EKM were the major regions for specimens, while there still remain underexplored regions with vast areas, like MLU, even though a certain number of MNPs have already been discovered. A large number of Indonesian marine macro- and microorganisms are still underexplored, and they may provide inspiration for many chemical entities. The significant biological activity of Indonesian MNPs is dominated by cytotoxicity (16.7%), followed by antibacterial activity (5.9%). By exploring untapped novel groups of organisms and by proposing newer biological targets, MNP researchers may be able to enhance the search for new marine drugs to treat human diseases. Moreover, careful and innovative techniques for the MNPs isolation are required for identification of new structures and activities including unstable intermediates [92]. The establishment of the Nagoya Protocols on the Convention on Biological Diversity (CBD) in 2010 has had a positive impact on global biodiversity especially in Indonesia by encouraging productive interaction between biodiversity-rich source countries and the more science and technology-advanced countries. International natural product researchers are strongly urged to be guided by the CBD principles in a fair and equitable framework that includes access and benefit-sharing [93]. These interactions will be crucial for conserving our global biodiversity [94] and providing valuable new MNPs for humankind. Therefore, the current primary issues in marine conservation, such as the loss of biodiversity through over-exploitation and habitat degradation, can be overcome. Additional information can be found in the Supplementary Materials [95,96,97,98,99,100,101,102,103,104,105,106,107,108,109,110,111,112,113,114,115,116,117,118,119,120,121,122,123,124,125,126,127,128,129,130,131,132,133,134,135,136,137,138,139,140,141,142,143,144,145,146,147,148,149,150,151,152,153,154,155,156,157,158,159,160,161,162,163,164,165,166,167,168,169,170,171,172,173,174,175,176,177,178,179,180,181,182,183,184,185,186,187,188,189,190,191,192,193,194,195,196,197,198,199,200,201,202,203,204,205,206,207,208,209,210,211,212,213,214,215,216,217,218,219,220,221,222,223,224,225,226,227,228,229,230,231,232,233,234,235,236,237,238,239,240,241,242,243,244,245,246,247,248,249,250,251,252,253,254,255,256,257,258,259,260,261,262,263,264,265,266,267,268,269,270,271,272,273,274,275,276,277,278,279,280,281,282,283,284,285,286,287,288,289,290,291,292,293,294,295,296,297,298,299,300,301,302,303,304,305,306,307,308,309,310,311,312,313,314,315,316,317,318,319,320,321,322,323,324,325,326,327,328,329,330,331,332,333,334,335,336,337,338,339,340,341,342,343,344,345,346,347,348,349,350,351,352,353,354,355,356,357,358,359,360,361,362,363,364,365,366,367,368,369,370,371,372,373,374,375,376,377,378,379,380,381,382,383,384,385,386,387,388,389,390,391,392,393,394,395,396,397,398,399,400,401,402,403,404,405,406,407,408,409,410,411,412,413,414,415,416,417,418,419,420,421,422,423,424,425,426,427,428,429,430,431,432,433,434,435,436,437,438,439,440,441,442,443,444,445,446,447,448,449,450,451,452,453,454,455,456,457,458,459,460,461,462,463,464,465,466,467,468,469,470,471,472,473,474,475,476,477,478,479,480,481,482,483,484,485,486,487,488].

## Supplementary Materials

The following are available online at https://www.mdpi.com/1660-3397/17/6/364/s1, Figure S1: Structures of marine sesquiterpenoids from Indonesian waters found in 1970–2017, Table S1: Marine sesquiterpenoids from Indonesian waters found in 1970–2017, Figure S2: Structures of marine diterpenoids from Indonesian waters found in 1970–2017, Table S2: Marine diterpenoids from Indonesian waters found in 1970–2017, Figure S3: Structures of marine sesterterpenoids from Indonesian waters found in 1970–2017, Table S3: Marine sesterterpenoids from Indonesian waters found in 1970–2017, Figure S4: Structures of marine triterpenoids from Indonesian waters found in 1970–2017, Table S4: Marine triterpenoids from Indonesian waters found in 1970–2017, Figure S5: Structures of marine steroids from Indonesian waters found in 1970–2017, Table S5: Marine steroids from Indonesian waters found in 1970–2017, Figure S6: Structures of marine saponins from Indonesian waters found in 1970–2017, Table S6: Marine saponins from Indonesian waters found in 1970–2017, Figure S7: Structures of marine meroterpenoids from Indonesian waters found in 1970–2017, Table S7: Marine meroterpe-noids from Indonesian waters found in 1970–2017, Figure S8: Structures of marine piperidine alkaloids from Indonesian waters found in 1970–2017, Table S8: Marine piperidine alkaloids from Indonesian waters found in 1970–2017, Figure S9: Structures of marine pyridine alkaloids from Indonesian waters found in 1970–2017, Table S9: Marine pyridine alkaloids from Indonesian waters found in 1970–2017, Figure S10: Structures of marine indole alkaloids from Indonesian waters found in 1970–2017, Table S10: Marine indole alkaloids from Indonesian waters found in 1970–2017, Figure S11: Structures of marine acridine alkaloids from Indonesian waters found in 1970–2017, Table S11: Marine acridine alkaloids from Indonesian waters found in 1970–2017, Figure S12: Structures of marine quinoline and isoquinoline alkaloids from Indonesian waters found in 1970–2017,Table S12: Marine quinoline and isoquinoline alkaloids from Indonesian waters found in 1970–2017, Figure S13: Structures of marine tyrosine alkaloids from Indonesian waters found in 1970–2017, Table S13: Marine tyrosine alkaloids from Indonesian waters found in 1970–2017, Figure S14: Structures of marine pyrrole alkaloids from Indonesian waters found in 1970–2017, Table S14: Marine pyrrole alkaloids from Indonesian waters found in 1970–2017, Figure S15: Structures of marine imidazole alkaloids from Indonesian waters found in 1970–2017, Table S15: Marine imidazole alkaloids from Indonesian waters found in 1970–2017, Figure S16: Structures of marine polysulfur aromatic alkaloids from Indonesian waters found in 1970–2017, Table S16: Marine polysulfur aromatic alkaloids from Indonesian waters found in 1970–2017, Figure S17: Structures of marine serine-derived alkaloids from Indonesian waters found in 1970–2017, Table S17: Marine serine-derived alkaloids from Indonesian waters found in 1970–2017, Figure S18: Structures of other marine alkaloids from Indonesian waters found in 1970–2017, Table S18: Other marine alkaloids from Indonesian waters found in 1970–2017, Figure S19: Structures of marine peptides from Indonesian waters found in 1970–2017, Table S19: Marine peptides from Indonesian waters found in 1970–2017, Figure S20: Structures of marine fatty acids and linear molecules from Indonesian waters found in 1970–2017, Table S20: Marine fatty acids and linear molecules from Indonesian waters found in 1970–2017, Figure S21: Structures of polyketides from Indonesian waters found in 1970–2017, Table S21: Marine polyketides from Indonesian waters found in 1970–2017, Figure S22: Structures of carbohydrates from Indonesian waters found in 1970–2017, Table S22: Marine carbohydrates from Indonesian waters found in 1970–2017.
