# Supplementary material for: Marine Natural Products from Indonesian Waters
Source: Mar Drugs. 2019 Jun 19;17(6):364. doi: 10.3390/md17060364 (PMC6627775; doi:10.3390/md17060364)
Supplement: Supplementary file 1 [file marinedrugs-17-00364-s001.pdf]

Supplementary Materials

Marine Natural Products from Indonesian Waters

Novriyandi Hanif [1,*], Anggia Murni [2], Chiaki Tanaka [3] and Junichi Tanaka [4]

[1] Department of Chemistry, Faculty of Mathematics and Natural Sciences, IPB University (Bogor Agricultural University), Bogor 16680, Indonesia
[2] Tropical Biopharmaca Research Center, IPB University (Bogor Agricultural University), Bogor 16128, Indonesia
[3] Department of Natural Products Chemistry, Graduate School of Pharmaceutical Sciences, Kyushu University, Fukuoka 812–8582, Japan
[4] Department of Chemistry, Biology, and Marine Science, University of the Ryukyus, Nishihara, Okinawa 903-0213, Japan

* Email: nhanif@apps.ipb.ac.id

Table of Contents

Title	Page
Figure S1: Structures of marine sesquiterpenoids from Indonesian waters found in 1970–2017	4
Table S1: Marine sesquiterpenoids from Indonesian waters found in 1970–2017	5
Figure S2: Structures of marine diterpenoids from Indonesian waters found in 1970–2017	12
Table S2: Marine diterpenoids from Indonesian waters found in 1970–2017	13
Figure S3: Structures of marine sesterterpenoids from Indonesian waters found in 1970–2017	24
Table S3: Marine sesterterpenoids from Indonesian waters found in 1970–2017	25
Figure S4: Structures of marine triterpenoids from Indonesian waters found in 1970–2017	30
Table S4: Marine triterpenoids from Indonesian waters found in 1970–2017	31
Figure S5: Structures of marine steroids from Indonesian waters found in 1970–2017	35
Table S5: Marine steroids from Indonesian waters found in 1970–2017	36
Figure S6: Structures of marine saponins from Indonesian waters found in 1970–2017	42
Table S6: Marine saponins from Indonesian waters found in 1970–2017	43
Figure S7: Structures of marine meroterpenoids from Indonesian waters found in 1970–2017	44
Table S7: Marine meroterpenoids from Indonesian waters found in 1970–2017	45
Figure S8: Structures of marine piperidine alkaloids from Indonesian waters found in 1970–2017	49
Table S8: Marine piperidine alkaloids from Indonesian waters found in 1970–2017	50
Figure S9: Structures of marine pyridine alkaloids from Indonesian waters found in 1970–2017	59
Table S9: Marine pyridine alkaloids from Indonesian waters found in 1970–2017	60
Figure S10: Structures of marine indole alkaloids from Indonesian waters found in 1970–2017	61
Table S10: Marine indole alkaloids from Indonesian waters found in 1970–2017	62
Figure S11: Structures of marine acridine alkaloids from Indonesian waters found in 1970–2017	67
Table S11: Marine acridine alkaloids from Indonesian waters found in 1970–2017	68
Figure S12: Structures of marine quinoline and isoquinoline alkaloids from Indonesian waters found in 1970–2017	70
Table S12: Marine quinoline and isoquinoline alkaloids from Indonesian waters found in 1970–2017	71
Figure S13: Structures of marine tyrosine alkaloids from Indonesian waters found in 1970–2017	73
Table S13: Marine tyrosine alkaloids from Indonesian waters found in 1970–2017	74
Figure S14: Structures of marine pyrrole alkaloids from Indonesian waters found in 1970–2017	78
Table S14: Marine pyrrole alkaloids from Indonesian waters found in 1970–2017	79
Figure S15: Structures of marine imidazole alkaloids from Indonesian waters found in 1970–2017	83

Table S15: Marine imidazole alkaloids from Indonesian waters found in 1970–2017 84
Figure S16: Structures of marine polysulfur aromatic alkaloids from Indonesian waters found in 1970–2017 87
Table S16: Marine polysulfur aromatic alkaloids from Indonesian waters found in 1970–2017 88
Figure S17: Structures of marine serine-derived alkaloids from Indonesian waters found in 1970–2017 92
Table S17: Marine serine-derived alkaloids from Indonesian waters found in 1970–2017 93
Figure S18: Structures of other marine alkaloids from Indonesian waters found in 1970–2017 95
Table S18: Other marine alkaloids from Indonesian waters found in 1970–2017 96
Figure S19: Structures of marine peptides from Indonesian waters found in 1970–2017 106
Table S19: Marine peptides from Indonesian waters found in 1970–2017 107
Figure S20: Structures of marine fatty acids and linear molecules from Indonesian waters found in 1970–2017 116
Table S20: Marine fatty acids and linear molecules from Indonesian waters found in 1970–2017 117
Figure S21: Structures of polyketides from Indonesian waters found in 1970–2017 124
Table S21: Marine polyketides from Indonesian waters found in 1970–2017 125
Figure S22: Structures of carbohydrates from Indonesian waters found in 1970–2017 140
Table S22: Marine carbohydrates from Indonesian waters found in 1970–2017 141

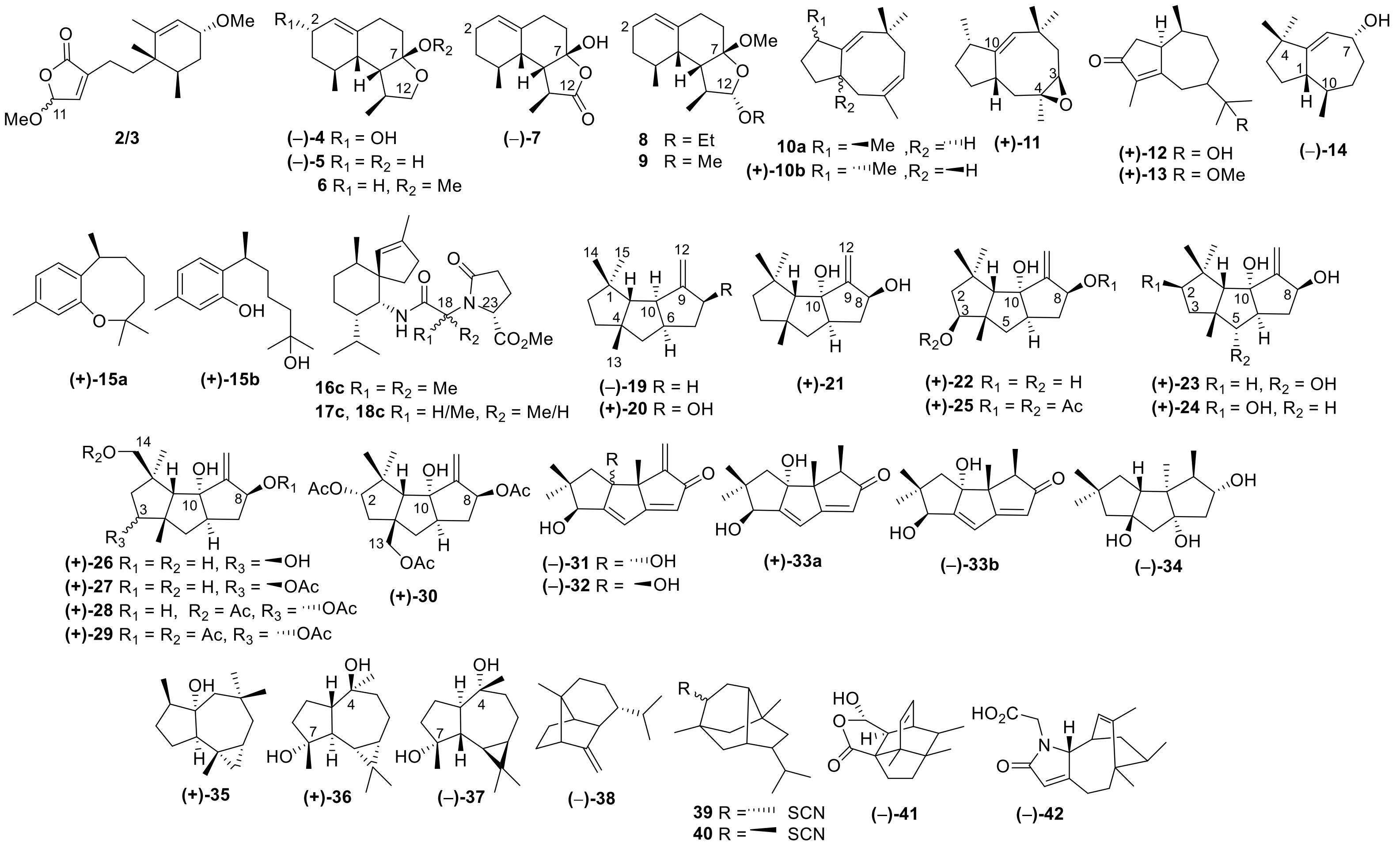

Figure S1: Structures of marine sesquiterpenoids from Indonesian waters found in 1970–2017.

Table S1: Marine sesquiterpenoids from Indonesian waters found in 1970–2017.

Compound	Structure Elucidation	Chemistry Type	Drug Class	Biological Activity		Source of Organism	Province	Ref
				Cell/Enzyme/Micro-organism/Insect/Others	Activity			
O,*O*-Dimethyl lingshuiolide A **2**/11-*epi* **3**[α,β] $[C_{17}H_{26}O_4]$	UV, IR, MS, NMR ECD, $[\alpha]_D$	Lingshuiane♣	Cytotoxic, Anti-microbial, Antiathero-sclerotic, Antiosteo-clastic, Anticancer	Several cell lines, microorganisms, enzymes	NA	*L. herbacea*	NSW	[95]
(–)-Lemnacarnol **4**[β] $[C_{15}H_{24}O_3]$	IR, MS, NMR, $[\alpha]_D$, CT, X-ray	Nardosinane	Toxic	*P. micans, A. carterae*	Quan. Undetm.	*L. carnosa*	MLU	[96–99]
(–)-2-Desoxylemnacarnol **5**[β] $[C_{15}H_{24}O_2]$	IR, MS, NMR, $[\alpha]_D$, CT	Nardosinane	Undetm.	Undetm.	Undetm.	*L. africana*, *L. laevis*, *P. thyrsoides*	MLU	[100]
2-Deoxy-7-*O*-methyllemnacarnol **6**[β] $[C_{16}H_{26}O_2]$	UV, IR, MS, NMR	Nardosinane	Undetm.	Undetm.	Undetm.	*Nephthea* sp.	NSW	[101]
(–)-2-Desoxy-12-oxolemnacarnol **7**[β] $[C_{15}H_{22}O_3]$	IR, MS, NMR, $[\alpha]_D$, CT, X-ray	Nardosinane	Undetm.	Undetm.	Undetm.	*L. africana*, *L. laevis*, *P. thyrsoides*	MLU	[100]
2-Deoxy-12α-ethoxy-7-*O*-methyllemnacarnol **8**[β] $[C_{18}H_{30}O_3]$	UV, IR, MS, NMR	Nardosinane	Undetm.	Undetm.	Undetm.	*Nephthea* sp.	NSW	[101]
2-Deoxy-12α-methoxy-7-*O*-methyllemnacarnol **9**[β] $[C_{17}H_{28}O_3]$	UV, IR, MS, NMR	Nardosinane	Undetm.	Undetm.	Undetm.	*Nephthea* sp.	NSW	[101]
Precapnelladiene **10a**[β] $[C_{15}H_{24}]$	IR, GCMS, NMR, Mol. Mod. (CONGEN)	Precapnellane	Undetm.	Undetm.	Undetm.	*C. imbricata*	MLU	[102]
(+)-Precapnelladiene **10b**[γ] $[C_{15}H_{24}]$	TS	Precapnellane	Undetm.	Undetm.	Undetm.			[49–52]
(+)-3α,4α-Epoxyprecapnell-10-ene **11**[β] $[C_{15}H_{24}O]$	MS, NMR, $[\alpha]_D$	Precapnellane	Cytostatic<br>Cytotoxic	L-929, K562<br>HeLa	GI_{50} = 227.3 ± 1.8–4.3 μM<br>CC_{50} = 193.2 ± 4.3 μM	*D. rubeola*	BLI	[103]
(+)-Hydroxycolorenone **12**[β] $[C_{15}H_{24}O_2]$	UV, MS, NMR, $[\alpha]_D$	Guaiane	Antiinsecticidal	*S. littoralis*	EC_{50} = 8.8 ± 0.26 μg/mL<br>LC_{50} = 453 ± 0.43 μg/mL	*N. chabrolii*	WST	[104]
(+)-Methoxycolorenone **13**[β] $[C_{16}H_{26}O_2]$	UV, MS, NMR, $[\alpha]_D$	Guaiane	Antiinsecticidal	*S. littoralis*	NA	*N. chabrolii*	WST	[104]

Table S1: *Cont.*

Compound	Structure Elucidation	Chemistry Type	Drug Class	Biological Activity: Cell/Enzyme/Micro-organism/Insect/Others	Biological Activity: Activity	Source of Organism	Province	Ref
(–)-**14**[β] [$C_{13}H_{22}O_2$]	IR, MS, NMR, [α]D, QCC	*Trinor*-guaiane	Cytotoxic	NBT-T2	NA (10 μg/mL)	*Anthelia* sp.	BTN	[105]
(+)-Helianane **15a**[β] [$C_{15}H_{22}O_2$]	UV, MS, NMR, [α]D	Helianane	Undetm.	Undetm.	Undetm.	*H. fascigera*	NSW	[106]
(+)-Curcudiol **15b**[δ] [$C_{15}H_{24}O_2$]	TS	Bisabolane	Undetm.	Undetm.	Undetm.			[53–57]
Boneratamide A methyl ester **16c**[ε] [$C_{24}H_{40}N_2O_4$]	MS, NMR, X-ray	Spiroaxane	Cytostatic	Antimitotic assay	NA	*A. aplysinoides*	SSW	[107]
Boneratamides B **17c**/C **18c**[α,ε] methyl esters [$C_{24}H_{38}N_2O_4$]	MS, NMR	Spiroaxane	Cytostatic	Antimitotic assay	NA	*A. aplysinoides*	SSW	[57]
(–)-$\Delta^{9(12)}$-Capnellene **19**[β] [$C_{15}H_{24}$]	IR, MS, NMR, [α]D, CT	Capnellane	Undetm.	Undetm.	Undetm.	*C. imbricata*	MLU	[108]
(+)-Capnellene-8β-ol **20**[β] [$C_{15}H_{24}O$]	IR, MS, NMR, [α]D	Capnellane	Cytotoxic	MCF7, HT-115<br>HL-60<br>K562, A-2780<br>G-402	IC_{50} > 4500 μM<br>IC_{50} = 68 μM<br>IC_{50} = 4.6 – 6.6 μM<br>IC_{50} > 4.5 μM	*C. imbricata*	NMU	[109, 62]
			Anti-inflammatory	RAW 264.7 (LPS/iNOS, COX-2)	NA			
			Toxic	*C. septentrionalis*, *A. japonica*, *T. excentricus*, *P. micans*, *A. carterae*	Quan. Undetm.			
(+)-$\Delta^{9(12)}$-Capnellene-8β,10α-diol **21**[β] [$C_{15}H_{24}O_2$]	IR, MS, NMR, [α]D, CT	Capnellane	Cytotoxic	KB, HL-60, G-402, HT-115, MCF7<br>K562, WIDr, A2780<br>HeLa	IC_{50} = 25.6 – 93 μM<br>IC_{50} = 0.7 – 9.7 μM<br>CC_{50} = 7.6 ± 0.8 μM<br>IC_{50} = 15.1 μM	*C. imbricata*	MLU	[62, 103, 109 – 112]
			Cytostatic	L-929	GI_{50} = 6.8 ± 0.8 μM			
			Anti-inflammatory	RAW 264.7 (LPS/ iNOS, COX-2)<br>BV2 (IFN-γ/iNOS, COX-2)	1.2 ± 0.1 – 24.8 ± 7.5% (10 μM)<br>IC_{50} = 6.21 ± 2.5 – 17.1 ± 2.8 μM			
			Antinociception	Murine neuropathy	10 mg/kg (CCI therm. hyperalgesia behav.)			

Table S1: *Cont.*

Compound	Structure Elucidation	Chemistry Type	Drug Class	Biological Activity: Cell/Enzyme/Micro-organism/Insect/Others	Biological Activity: Activity	Source of Organism	Province	Ref
(+)-$\Delta^{9(12)}$-Capnellene-3β,8β,10α-triol **22**$^{\beta}$ [$C_{15}H_{24}O_3$]	UV, IR, MS, NMR, ECD, $[\alpha]_D$, CT, X-ray	Capnellane	Toxic	*P. micans*, *A. carterae*	Quan. Undetm.	*C. imbricata*	MLU	[62, 111, 113]
(+)-$\Delta^{9(12)}$-Capnellene-5α,8β,10α-triol **23**$^{\beta}$ [$C_{15}H_{24}O_3$]	IR, MS, NMR, $[\alpha]_D$, CT	Capnellane	Undetm.	Undetm.	Undetm.	*C. imbricata*	MLU	[111]
$\Delta^{9(12)}$-Capnellene-2ξ,8β,10α-triol **24**$^{\beta}$ [$C_{15}H_{24}O_3$]	IR, MS, NMR, $[\alpha]_D$, CT	Capnellane	Undetm.	Undetm.	Undetm.	*C. imbricata*	MLU	[111]
(+)-3α,8β-Diacetoxycapnell-9(12)-ene-10α-ol **25**$^{\beta}$ [$C_{19}H_{28}O_5$]	MS, NMR, $[\alpha]_D$	Capnellane	Cytotoxic<br>Cytostatic	HeLa<br>K562, L-929	CC_{50} > 125 μM<br>GI_{50} = 62.2 ± 2.7 – 99.1 ± 1.8 μM	*D. rubeola*	BLI	[103]
(+)-$\Delta^{9(12)}$-Capnellene-3β,8β,10α,14-tetraol **26**$^{\beta}$ [$C_{15}H_{24}O_4$]	IR, MS, NMR, $[\alpha]_D$, CT	Capnellane	Undetm.	Undetm.	Undetm.	*C. imbricata*	MLU	[108, 114]
(+)-3β-Acetoxycapnellene-8β,10α,14β-triol **27**$^{\beta}$ [$C_{17}H_{26}O_5$]	IR, MS, NMR, $[\alpha]_D$	Capnellane	Cytotoxic	HL-60, MCF7<br>K562, G-402, A2780<br>HT-115	IC_{50} = 713 − 1029 μM<br>IC_{50} = 24 – 52 μM<br>NA	*C. imbricata*	NMU	[109]
(+)-3α,14-Diacetoxycapnell-9(12)-ene-8β,10α-diol **28**$^{\beta}$ [$C_{19}H_{28}O_6$]	MS, NMR, $[\alpha]_D$	Capnellane	Cytostatic	K562	GI_{50} = 142.0 ± 4.7 μM	*D. rubeola*	NSW	[103]
(+)-3α,8β,14-Triacetoxy-capnell-9(12)-ene-10α-ol **29**$^{\beta}$ [$C_{21}H_{30}O_7$]	MS, NMR, $[\alpha]_D$	Capnellane	Cytostatic<br>Cytotoxic	K562<br>HeLa	GI_{50} = 126.9 ± 0.2 μM<br>CC_{50} > 125 μM	*D. rubeola*	NSW	[103]
(–)-2α,8β,13-Triacetoxy-capnell-9(12)-ene-10α-ol **30**$^{\beta}$ [$C_{21}H_{30}O_7$]	MS, NMR, $[\alpha]_D$	Capnellane	Cytostatic<br>Cytotoxic	K562<br>HeLa	GI_{50} = 126.9 ± 3.0 μM<br>CC_{50} > 125 μM	*D. rubeola*	NSW	[103]
(–)-Hirsutanol A **31**$^{\beta}$ [$C_{15}H_{18}O_3$]	IR, MS, NMR, $[\alpha]_D$, X-ray, CT	Hirsutane	Cytotoxic	SW480, CNE2<br>SW620, LoVo, Hep3B, SUNE1<br>MCF7, CNE1, CNE2, A549	ED_{50} = 3.03 ± 0.07 μg/mL<br>ED_{50} = 0.58 ± 0.09 – 0.90 ± 0.19 μg/mL<br>ED_{50} = 2.55 ± 0.41 − 3.13 ± 0.29 μg/mL	A fungus (symbiont) *Haliclona* sp. (host)	GTO	[58, 115 – 117]

Table S1: *Cont.*

Compound	Structure Elucidation	Chemistry Type	Drug Class	Biological Activity: Cell/Enzyme/Micro-organism/Insect/Others	Biological Activity: Activity	Source of Organism	Province	Ref
(−)-Hirsutanol A **31**[β] [$C_{15}H_{18}O_3$]	IR, MS, NMR, $[\alpha]_D$, X-ray, CT	Hirsutane	Cytotoxic	MDA-MB-231, MDA-MB-435<br>HepG2<br>Bel-7402<br>HeLa<br>B16	ED_{50} = 1.34 ± 0.19 − 1.82 ± 0.37 μg/mL<br>IC_{50} = 2.45 ± 0.13 μg/mL<br>IC_{50} = 6.11 ± 0.41 μg/mL<br>ED_{50} = 8.27 ± 0.71 μg/mL<br>ED_{50} = 25.6 μM	A fungus (symbiont) *Haliclona* sp. (host)	GTO	[58, 115–117]
			Antibacterial	*B. subtilis*<br>*B. subtilis* ATCC 70385, *S. aureus* 6538, *E. coli* ATCC 8739	200 μg/disk<br>MIC > 100 μM			
Hirsutanol B **32**[β] [$C_{15}H_{18}O_3$]	NMR	Hirsutane	Undetm.	Undetm.	Undetm.	A fungus (symbiont) *Haliclona* sp. (host)	GTO	[58, 115–117]
(+)-Hirsutanol C **33a**[β] [$C_{15}H_{18}O_3$]	IR, MS, NMR, $[\alpha]_D$	Hirsutane	Cytotoxic	LoVo	IC_{50} > 200 μM	A fungus (symbiont) *Haliclona* sp. (host)	GTO	[58, 115–117]
(−)-Hirsutanol C **33b**[γ] [$C_{15}H_{18}O_3$]	CT	Hirsutane	Cytotoxic	B16	IC_{50} > 200 μM	A fungus (symbiont) *G. incarnatum* (host)	JPN	[58, 115–117]
			Antibacterial	*B. subtilis* ATCC 70385, *S. aureus* 6538, *E. coli* ATCC 8739	MIC > 100 μM			
ent-Gloeosteretriol [(−)-Hirsutanol F] **34**[β] [$C_{15}H_{26}O_3$]	UV, IR, MS, NMR, $[\alpha]_D$	Hirsutane	Cytotoxic	SW480, MCF7, MDA-MB-231, MDA-MB-435, MDA-MB-453, CNE1, CNE2, SUNE1, A549, Hep3B, HepG2, Bel-7402,HeLa	ED_{50} > 50 μg/mL	A fungus (symbiont) *Haliclona* sp. (host)	GTO	[58, 115–117]
			Antibacterial	*B. subtilis*	NA (200 μg/disk)			
(+)-Africanol **35**[β] [$C_{15}H_{26}O$]	IR, MS, NMR, $[\alpha]_D$, X-ray	Africanane	Toxic	*L. reticulatus*, *C. septentrionalis*, *A. japonica*, *T. excentricus*, *P. micans*, *A. carterae*	Quan. Undetm.	*L. africana*, *L. nitida*	MLU	[59, –62, 118]
(+)-4α,7β-Aromadendranediol **36**[β] [$C_{15}H_{26}O_2$]	IR, MS, NMR, $[\alpha]_D$, CT	Aromaden-drane	Undetm.	Undetm.	Undetm.	*S. mayi*	NST	[119]

Table S1: *Cont.*

Compound	Structure Elucidation	Chemistry Type	Drug Class	Biological Activity: Cell/Enzyme/Micro-organism/Insect/Others	Biological Activity: Activity	Source of Organism	Province	Ref
(−)-4α,7α-Aromadendranediol **37**[β]<br>[$C_{15}H_{26}O_2$]	IR, MS, NMR, $[\alpha]_D$, CT	Aromaden-drane	Undetm.	Undetm.	Undetm.	*S. mayi*	NST	[119]
(−)-Sinularane **38**[β]<br>[$C_{15}H_{24}$]	IR, MS, NMR, $[\alpha]_D$, ORD, ECD, X-ray, CT	Sinularane	Undetm.	Undetm.	Undetm.	*S. mayi*	NST	[119, 120]
9-Thiocyanatopupukeanane **39**/9-*epi* **40**[α,β]<br>[$C_{16}H_{25}NS$]	MS, NMR	Pupukeanane♦	Cytotoxic<br>Antibacterial<br>Antifungal	*A. salina*<br>*B. subtilis*<br>*C. albicans*	90% (**39**:**40** = 30:70)<br>35% (**39**:**40** = 50:50)<br>W (**39**:**40** = 30:70, 20 μg)<br>M (**39**:**40** = 30:70, 20 μg)	*P. varicosa*, *A. aculeata*	JSCR	[121]
(−)-Lamellodysidine A **41**[β]<br>[$C_{15}H_{20}O_3$]	UV, IR, MS, NMR, ECD, $[\alpha]_D$	Lamello-dysidane♣	Cytotoxic, Anti-microbial, Antiathero-sclerotic, Antiosteo-clastic, Anticancer	Several cell lines, microorganisms, enzymes	NA	*L. herbacea*	NSW	[36]
(−)-Lamellodysidine B **42**[β]<br>[$C_{17}H_{23}NO_3$]	UV, IR, MS, NMR, ECD, $[\alpha]_D$	Nakafurane 8	Cytotoxic, Anti-microbial, Antiathero-sclerotic, Antiosteo-clastic, Anticancer	Several cell lines, microorganisms, enzymes	NA	*L. herbacea*	NSW	[36]

Footnote: **1. Structure** ([α]molecule isolated as a mixture, [β]original molecule, [γ]revised molecule (new), [δ]revised molecule (known), [ε]molecule isolated from its derivative using chemical reaction, skeleton names in black were already reported in the literature; those in blue were named in this review, ♣new skeleton, ♦rare FG, ♠rare motif); **2. Structure Elucidation** (**UV** ultraviolet spectroscopy, **IR** infrared spectroscopy, **MS** mass spectrometry, **NMR** nuclear magnetic resonance, **ECD** electronic circular dichroism, **X-ray** single-crystal X-ray diffraction, **Mol. Mod.** molecular modeling, **QCC** quantum chemical calculation, **CT** chemical transformation, **TS** total synthesis, **$[\alpha]_D$** specific optical rotation); **3. Statistic** (**CC_{50}** 50% of the cytotoxic concentration, **EC_{50}** 50% of the effective concentration, **ED_{50}** 50% of the effective dose, **GI_{50}** 50% of the growth inhibition which emphasizes the correction for the cell count at time zero, **IC_{50}** 50% of the inhibitory concentration, **LC_{50}** 50% of the lethal concentration, **MIC** minimum inhibitory concentration); **4. Activity** (**NA** no activity or not active, **B16** murine melanoma, **BV2** murine microglia, **L-929** murine fibroblast, **NBT-T2** murine bladder epithelial tumor, **RAW 264.7** murine macrophage, **A549** human lung carcinoma, **A2780** human ovarian carcinoma, **Bel-7402** human hepatocarcinoma, **CNE1** human nasopharyngeal carcinoma, **CNE2** human nasopharyngeal carcinoma, **G-402** human renal leiomyoblastoma, **HeLa** human cervical carcinoma, **Hep3B** human hepatocarcinoma, **HepG2** human hepatocarcinoma, **HL-60** human acute promyelocytic leukemia, **HT-115** human colorectal adenocarcinoma, **K562** human erythro myeloblastoid leukemia, **KB** human nasopharyngeal epidermoid carcinoma, **LoVo** human colorectal adenocarcinoma, **MCF7** human breast adenocarcinoma, **MDA-MB-231** human breast adenocarcinoma, **MDA-MB-435** human breast adenocarcinoma, **SUNE1** human nasopharyngeal carcinoma, **SW480** human colorectal adenocarcinoma, **SW620** human colorectal adenocarcinoma, **WIDr** human colorectal adenocarcinoma, **COX-2** cyclooxygenase-2 enzyme, **iNOS** inducible nitric oxide synthase enzyme, **LPS** lipopolysaccharide, **IFN-γ** interferon gamma, **CCI** chronic constriction injury, **behav**. behaviour, **Quan. Undetm** quantitatively undetermined, **therm** thermal, **Undetm.** undetermined, **W** weak, **M** Moderate); 5. Geography (**JPN** Japan).

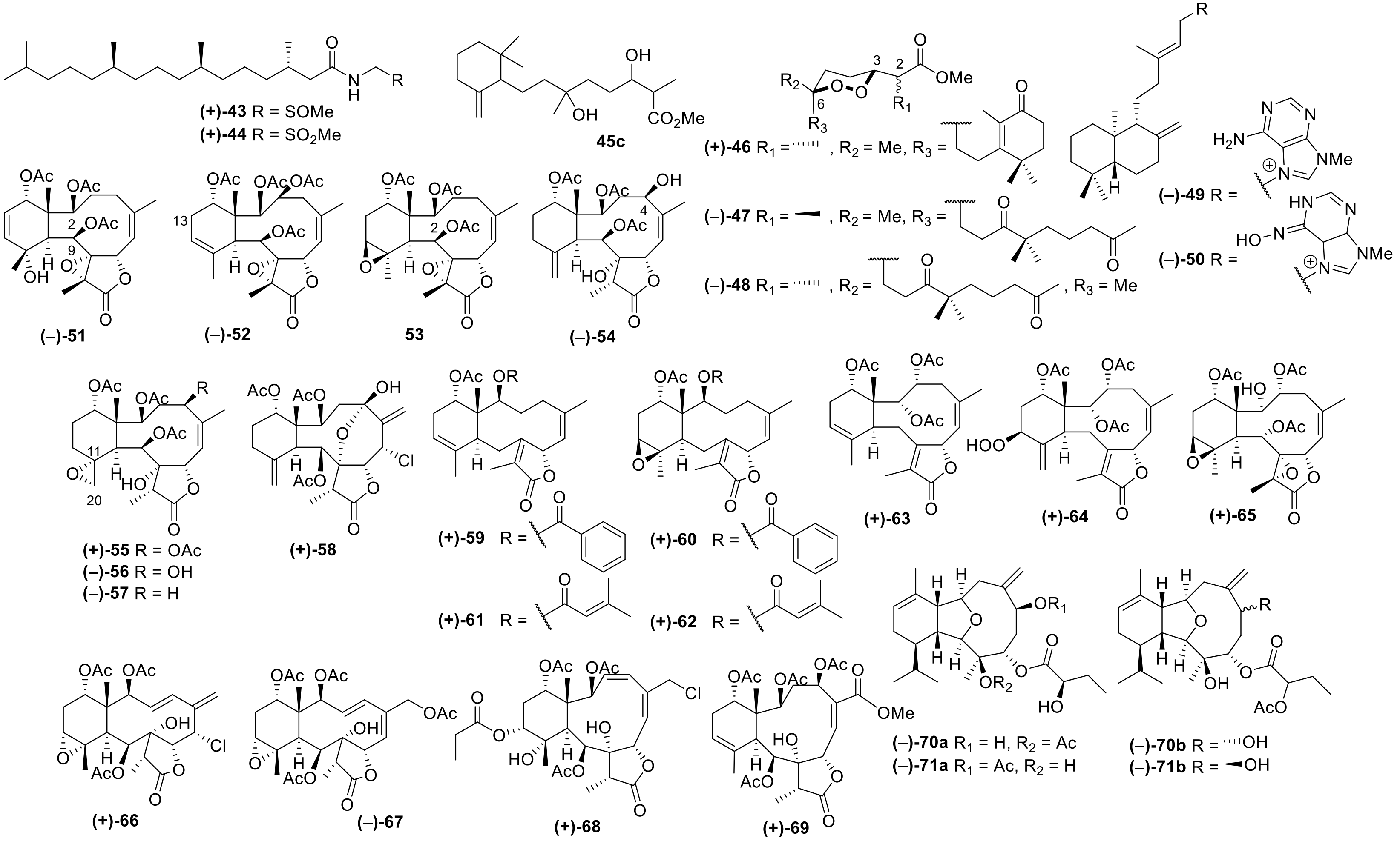

Figure S2: *Cont.*

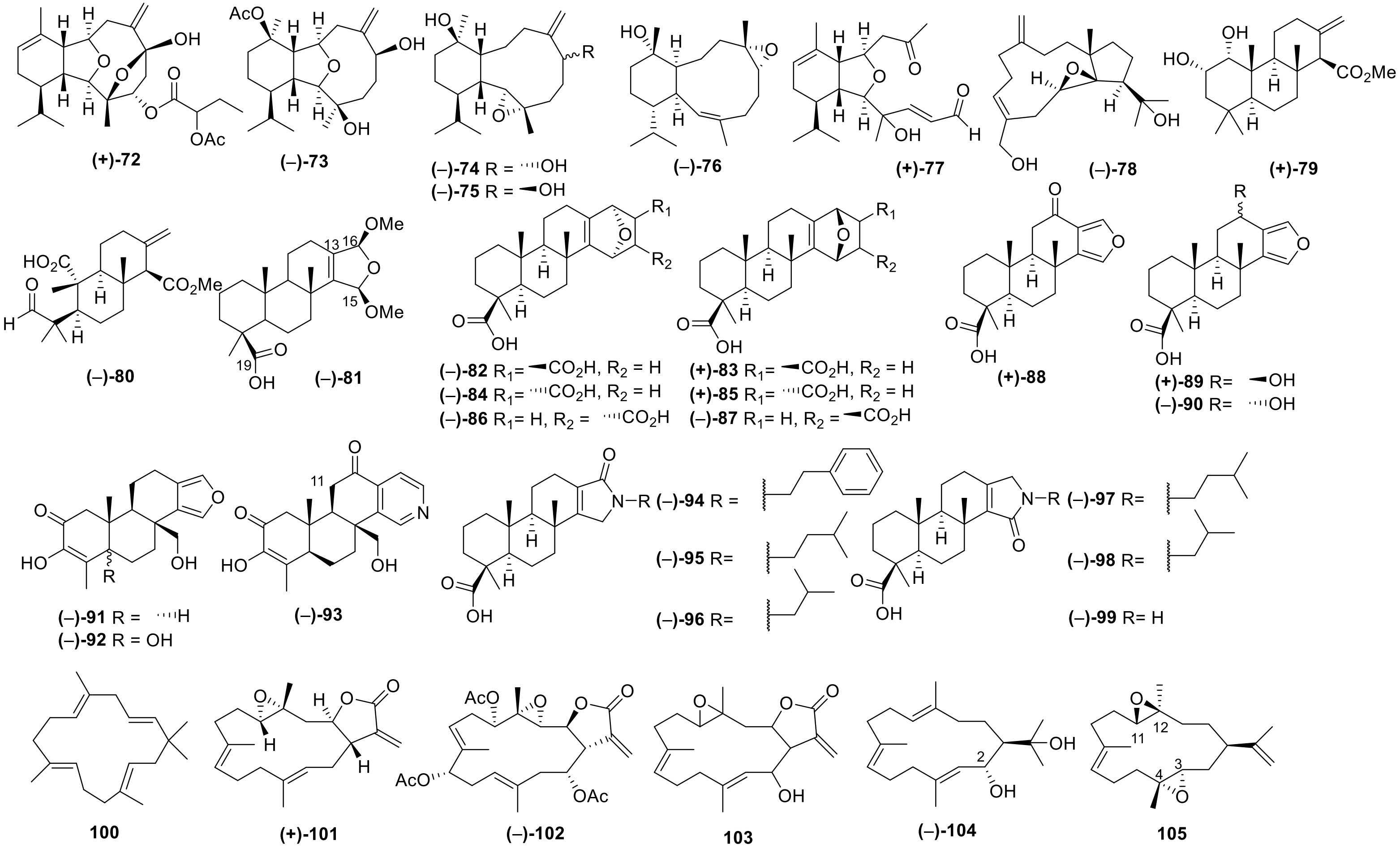

Figure S2: *Cont.*

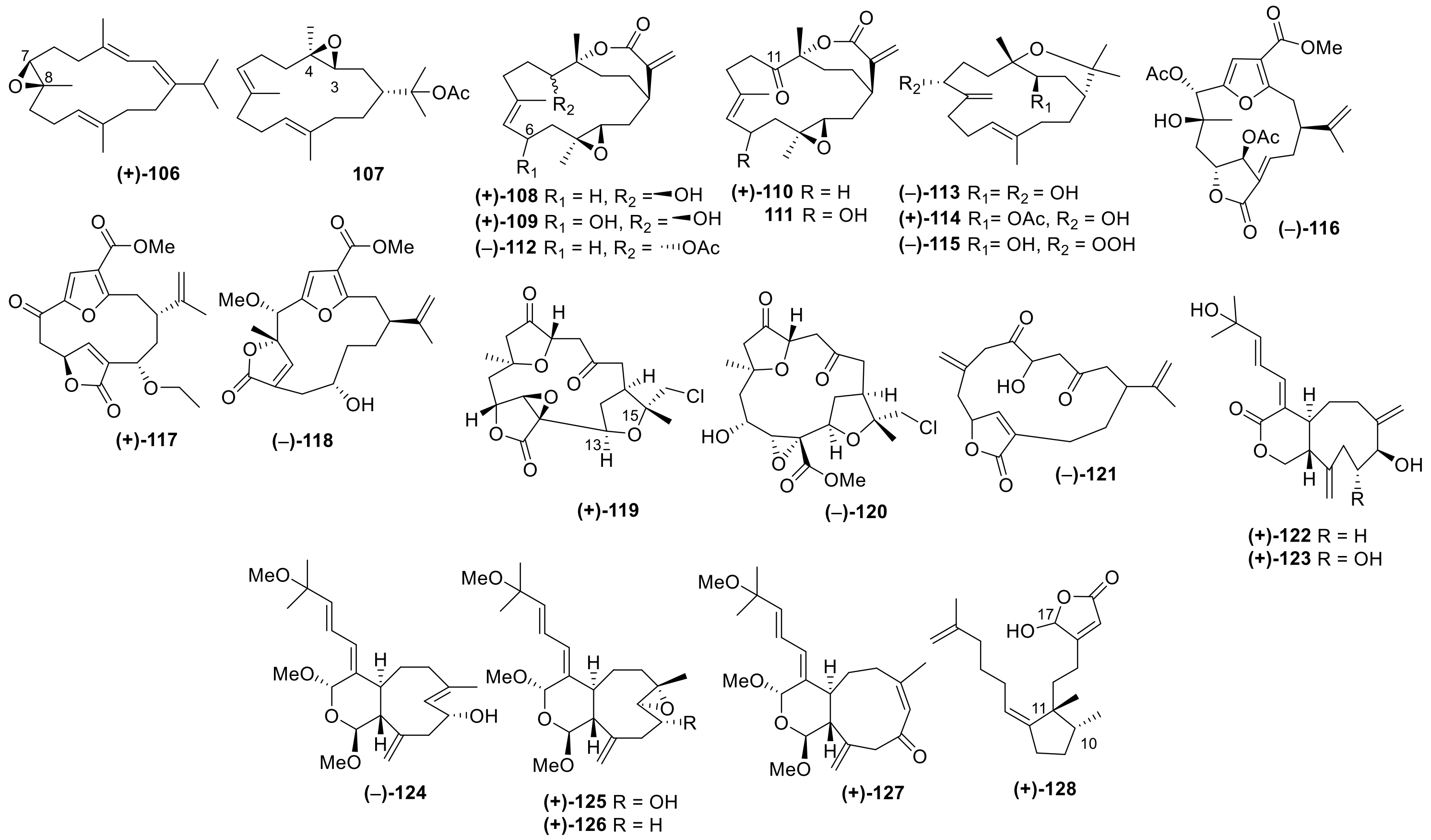

Figure S2: Structures of marine diterpenoids from Indonesian waters found in 1970–2017.

Table S2: Marine diterpenoids from Indonesian waters found in 1970–2017.

Compound	Structure Elucidation	Chemistry Type	Drug Class	Biological Activity: Cell/Enzyme/Micro-organism/Insect/Others	Biological Activity: Activity	Source of Organism	Province	Ref
(+)-Sinulasulfoxide **43**[β]<br>[$C_{23}H_{47}NO_2S$]	IR, MS, NMR, ECD, [α]D, CT	Acyclic diterpene[♦]	Anti-inflammatory	J774 (LPS/iNOS)	22.6% (30 μM)	*Sinularia* sp.	NSW	[122]
(+)-Sinulasulfone **44**[β]<br>[$C_{23}H_{47}NO_3S$]	NMR, MS, ECD, [α]D, CT	Acyclic diterpene[♦]	Undetm.	Undetm.	Undetm.	*Sinularia* sp.	NSW	[122]
nor-Diterpene **45c**[ε]<br>[$C_{20}H_{36}O_4$]	IR, MS, NMR, CT	Acyclic diterpene	Undetm.	Undetm.	Undetm.	*D. megaspi-norhabdosa*	SSW	[123]
(+)-Diacaperoxide A **46**[β]<br>[$C_{20}H_{32}O_5$]	MS, NMR, [α]D	Acyclic peroxide diterpene	Cytotoxic	L5178Y<br>HeLa, PC12<br>*A. salina*	EC_{50}> 10 μg/mL<br>NA<br>35 – 55%<br>(10 μg/mL, 24 – 48 h)	*D. megaspi-norhabdosa*	SSW	[124]
(–)-Diacaperoxide B **47**[β]<br>[$C_{20}H_{34}O_6$]	MS, NMR, [α]D	Acyclic peroxide diterpene	Cytotoxic	L5178Y<br>HeLa, PC12<br>*A. salina*	EC_{50}= 10 μg/mL<br>NA<br>45 – 55%<br>(10 μg/mL, 24 – 48 h)	*D. megaspi-norhabdosa*	SSW	[124]
(–)-Diacaperoxide C **48**[β]<br>[$C_{20}H_{34}O_6$]	MS, NMR, [α]D	Acyclic peroxide diterpene	Cytotoxic	L5178Y<br>HeLa, PC12<br>*A. salina*	EC_{50}> 10 μg/mL<br>NA<br>45 – 55%<br>(10 μg/mL, 24 – 48 h	*D. megaspi-norhabdosa*	SSW	[124]
(–)-Agelasine D **49**[β]<br>[$C_{26}H_{40}N_5$]	UV, MS, NMR, [α]D	Copalane	Cytotoxic<br>Antibacterial	L5178Y<br>*S. epidermidis*	IC_{50} = 4.03 μM<br>MIC < 0.0877 μM	*A. nakamurai*	JSCR	[125]
(–)-Ageloxime D **50**[β]<br>[$C_{26}H_{42}N_5O$]	UV, MS, NMR, [α]D	Copalane	Cytotoxic<br>Antibacterial	L5178Y<br>*S. epidermidis*	IC_{50} = 12.5 μM<br>MIC > 45 μM	*A. nakamurai*	JSCR	[125]
(–)-2,9-Diacetyl-2-debutyrylstecholide H **51**[β]<br>[$C_{26}H_{34}O_{10}$]	UV, MS, NMR, [α]D	Briarane	Cytotoxic	P-388, A549, HT-29, MEL-28	IC_{50}> 10 μg/mL	*Briareum* sp.	CSW	[126, 127]
(–)-13-Dehydroxystecholide J **52**[β]<br>[$C_{26}H_{38}O_{11}$]	UV, MS, NMR, [α]D	Briarane	Cytotoxic	P-388, A549, HT-29, MEL-28	IC_{50}> 10 μg/mL	*Briareum* sp.	CSW	[126, 127]
2β-Acetoxy-2-(debutyryloxy)-stecholide E acetate **53**[ζ]<br>[$C_{26}H_{34}O_{10}$]	MS, NMR, [α]D	Briarane	Cytotoxic	P-388<br>A549, HT-29, MEL-28	IC_{50}> 10 μg/mL<br>EC_{50} = 1.59 μg/mL<br>IC_{50}> 10 μg/mL	*Briareum* sp.	CSW	[126, 127]

Table S2: *Cont.*

Compound	Structure Elucidation	Chemistry Type	Drug Class	Biological Activity: Cell/Enzyme/Micro-organism/Insect/Others	Biological Activity: Activity	Source of Organism	Province	Ref
(–)-4-Deacetyljunceellolide D **54**[β] [$C_{26}H_{36}O_{10}$]	MS, NMR, $[\alpha]_D$, CT	Briarane	Cytotoxic	P-388, A549, HT-29, MEL-28	NA	*J. fragilis*	NMU	[128]
(+)-11α,20α-Epoxyjunceellolide D **55**[β] [$C_{28}H_{38}O_{12}$]	MS, NMR, $[\alpha]_D$, CT	Briarane	Cytotoxic	P-388, A549, HT-29, MEL-28	NA	*J. fragilis*	NMU	[128]
(–)-11α,20α-Epoxy-4-deacetyljunceellolide D **56**[β] [$C_{28}H_{36}O_{11}$]	MS, NMR, $[\alpha]_D$, CT	Briarane	Cytotoxic	P-388, A549, HT-29, MEL-28	NA	*J. fragilis*	NMU	[128]
(–)-11α,20α-Epoxy-4-deacetoxyjunceellolide D **57**[β] [$C_{28}H_{36}O_{10}$]	MS, NMR, $[\alpha]_D$, CT	Briarane	Cytotoxic	P-388, A549, HT-29, MEL-28	NA	*J. fragilis*	NMU	[128]
(+)-Junceellolide A **58**[β] [$C_{26}H_{33}ClO_{10}$]	MS, NMR, $[\alpha]_D$, CT	Briarane	Cytotoxic	P-388, A549, HT-29, MEL-28	NA	*J. fragilis*	NMU	[128]
(+)-Malayenolide A **59**[β] [$C_{29}H_{34}O_6$]	UV, MS, NMR, $[\alpha]_D$	Briarane	Cytotoxic	*A. salina*	LC_{50} = 100 µg/mL	*V. malayense*	NSW	[129]
(+)-Malayenolide B **60**[β] [$C_{29}H_{34}O_7$]	UV, MS, NMR, $[\alpha]_D$	Briarane	Cytotoxic	*A. salina*	LC_{50} < 2 µg/mL	*V. malayense*	NSW	[129]
(+)-Malayenolide C **61**[β] [$C_{27}H_{36}O_6$]	UV, MS, NMR, $[\alpha]_D$	Briarane	Cytotoxic	*A. salina*	LC_{50} = 20 µg/mL	*V. malayense*	NSW	[129]
(+)-Malayenolide D **62**[β] [$C_{27}H_{36}O_7$]	UV, MS, NMR, $[\alpha]_D$	Briarane	Cytotoxic	*A. salina*	LC_{50} = 20 µg/mL	*V. malayense*	NSW	[129]
(+)-Brianthein A **63**[β] [$C_{26}H_{34}O_8$]	MS, NMR, $[\alpha]_D$, CT, Mol. Mod.	Briarane	Cytotoxic	KB-3-1	11 ± 6 − 27 ± 5% (3 − 10 µg/mL)	*B. excavatum*	UEP	[130]
			Cytostatic	KB-C2, P-gp type	60 ± 2 − 84 ± 3% (0.1 µg/mL colchicine 3 − 10 µg/mL)			
(+)-Brianthein B **64**[β] [$C_{26}H_{34}O_{10}$]	MS, NMR, $[\alpha]_D$, CT	Briarane	Cytotoxic	KB-3-1	5 ± 1 − 26 ± 4% (3 − 10 µg/mL)	*B. excavatum*	UEP	[130]
			Cytostatic	KB-C2, P-gp type	26 ± 4 − 37 ± 6% (0.1 µg/mL colchicine 3 − 10 µg/mL)			

Table S2: *Cont.*

Compound	Structure Elucidation	Chemistry Type	Drug Class	Biological Activity: Cell/Enzyme/Micro-organism/Insect/Others	Biological Activity: Activity	Source of Organism	Province	Ref
(+)-Brianthein C **65**[β] [$C_{26}H_{34}O_{11}$]	MS, NMR, $[\alpha]_D$, CT	Briarane	Cytotoxic	KB-3-1	11 ± 1 − 17 ± 6% (3 − 10 µg/mL)	*B. excavatum*	UEP	[130]
			Cytostatic	KB-C2, P-gp type	0 ± 0 − 15 ± 2% (0.1 µg/mL colchicine 3 − 10 µg/mL)			
(+)-**66**[β] [$C_{26}H_{33}ClO_{10}$]	IR, MS, NMR, $[\alpha]_D$	Briarane	Cytostatic	KB-C2, P-gp type KB-CV60, MRP-1 type	NA	*Pteroeides* sp	ENT	[131]
(−)-**67**[β] [$C_{28}H_{36}O_{12}$]	IR, MS, NMR, $[\alpha]_D$	Briarane	Cytostatic	KB-C2, P-gp type KB-CV60, MRP-1 type	NA	*Pteroeides* sp	ENT	[131]
(+)-**68**[β] [$C_{29}H_{39}ClO_{12}$]	IR, MS, NMR, $[\alpha]_D$	Briarane	Cytostatic	KB-C2, P-gp type KB-CV60, MRP-1 type	NA	*Pteroeides* sp	ENT	[131]
(+)-**69**[β] [$C_{29}H_{38}O_{13}$]	IR, MS, NMR, $[\alpha]_D$	Briarane	Cytostatic	KB-C2, P-gp type KB-CV60, MRP-1 type	NA	*Pteroeides* sp.	ENT	[131]
(−)-Cladielloide A **70a**[β] [$C_{26}H_{40}O_7$]	IR, MS, NMR, $[\alpha]_D$, CT	Cladiellane	Cytotoxic	DLD-1, HL-60, CCRF-CEM, P388D1	IC_{50} > 40 µg/mL	*Cladiella* sp.	UEP	[132]
			Anti-inflammatory	Human neutrophils	20.5 ± 5.0% SA gen. FMLP/CB (10 µg/mL) 27.1 ± 4.8% elastase rel. FLMP/CB (10 µg/mL)			
(−)-Cladielloide A **70b**[γ] [$C_{26}H_{40}O_7$]	NMR 2D (NOESY)	Cladiellane	Undetm.	Undetm.	Undetm.	*Cladiella* sp.	UEP	[133]
(−)-Cladielloide B **71a**[β] [$C_{26}H_{40}O_7$]	IR, MS, NMR, $[\alpha]_D$	Cladiellane	Cytotoxic	DLD-1, CCRF-CEM	IC_{50} = 4.7 − 10.2 µg/mL	*Cladiella* sp.	UEP	[132]
				HL-60, P388D1	IC_{50} > 40 µg/mL			
			Anti-inflammatory	Human neutrophils	5.9 ± 0.7% SA gen. FMLP/CB (10 µg/mL) 6.5 ± 1.9% elastase rel. FMLP/CB (10 µg/mL)			
(−)-Cladielloide B **71b**[γ] [$C_{26}H_{40}O_7$]	NMR 2D (NOESY)	Cladiellane	Undetm.	Undetm.	Undetm.	*Cladiella* sp.	UEP	[133]
(+)-Cladielloide C **72**[β] [$C_{25}H_{38}O_7$]	IR, MS, NMR, $[\alpha]_D$, Mol. Mod.	Cladiellane	Cytotoxic	DLD-1, P388D1, HL-60	IC_{50} = 8.5 − 12.6 µg/mL	*Cladiella* sp.	UEP	[134]
				CCRF-CEM	IC_{50} = 3.6 µg/mL			

Table S2: *Cont.*

Compound	Structure Elucidation	Chemistry Type	Drug Class	Biological Activity: Cell/Enzyme/Micro-organism/Insect/Others	Biological Activity: Activity	Source of Organism	Province	Ref
(+)-Cladielloide C **72**$^{\beta}$<br>$[C_{25}H_{38}O_7]$	IR, MS, NMR, $[\alpha]_D$,<br>Mol. Mod.	Cladiellane	Anti-inflammatory	Human neutrophils	36.7 ± 7.6% SA gen.<br>FMLP/CB (10 μg/mL)<br>27.2 ± 3.6% elastase rel.<br>FMLP/CB (10 μg/mL)	*Cladiella* sp.	UEP	[134]
(–)-Cladieunicellin G **73**$^{\beta}$<br>$[C_{22}H_{36}O_5]$	IR, MS, NMR, $[\alpha]_D$,<br>Mol. Mod.	Cladiellane	Anti-inflammatory	Human neutrophils	1.97 ± 2.44% SA gen.<br>FMLP/CB (10 μg/mL)<br>12.89 ± 5.03% elastase rel.<br>FMLP/CB (10 μg/mL)	*Cladiella* sp.	UEP	[135]
(–)-Cladieunicellin F **74**$^{\beta}$<br>$[C_{20}H_{34}O_3]$	IR, MS, NMR, $[\alpha]_D$	Cladiellane	Anti-inflammatory	Human neutrophils	6.46 ± 1.28% SA gen.<br>FMLP/CB (10 μg/mL)<br>12.91 ± 3.56% elastase rel.<br>FMLP/CB (10 μg/mL)	*Cladiella* sp.	UEP	[133]
(–)-6-*epi*-Cladieunicellin F **75**$^{\beta}$<br>$[C_{20}H_{34}O_3]$	IR, MS, NMR, $[\alpha]_D$,<br>Mol. Mod.	Cladiellane	Anti-inflammatory	Human neutrophils	6.57 ± 0.85 % SA gen.<br>FMLP/CB (10 μg/mL)<br>41.98 ± 3.26% elastase rel.<br>FMLP/CB (10 μg/mL)	*Cladiella* sp.	UEP	[133]
(–)-Solenopodin C **76**$^{\beta}$<br>$[C_{20}H_{34}O_2]$	IR, MS, NMR, $[\alpha]_D$	Cladiellane	Anti-inflammatory	Human neutrophils	45.82 ± 2.49% SA gen.<br>FLMP/CB (10 μg/mL)<br>40.45 ± 5.80% elastase rel.<br>FLMP/CB (10 μg/mL)	*Cladiella* sp.	UEP	[133]
(+)-Cladielloide D **77**$^{\beta}$<br>$[C_{20}H_{30}O_4]$	IR, MS, NMR, $[\alpha]_D$,<br>Mol. Mod	*seco*-<br>cladiellane$^{\clubsuit}$	Cytotoxic	DLD-1, CCRF-CEM<br>HL-60, P388D1	IC_{50} = 11.6 – 35.1 μg/mL<br>IC_{50} > 40 μg/mL	*Cladiella* sp.	UEP	[134]
			Anti-inflammatory	Human neutrophils	31.4 ± 6.9% SA gen.<br>FMLP/CB (10 μg/mL)<br>10.7 ± 5.6% elastase rel.<br>FMLP/CB (10 μg/mL)			
(–)-**78**$^{\beta}$<br>$[C_{20}H_{32}O_3]$	IR, MS, NMR, $[\alpha]_D$	Dollabellane	Cytotoxic	NBT-T2	10 μg/mL	*Anthelia* sp.	BTN	[136]
(+)-Coelodiol **79**$^{\beta}$<br>$[C_{21}H_{34}O_4]$	IR, MS, NMR, $[\alpha]_D$,<br>ECD, CT	Isocopalane$^{\clubsuit}$	Cytostatic	MKN-45	20 μg/mL	*C.* cfr.<br>*singaporensis*	NSW	[137]
(–)-Coeloic acid **80**$^{\beta}$<br>$[C_{20}H_{30}O_5]$	IR, MS, NMR, $[\alpha]_D$	*seco*-nor<br>isocopalane$^{\clubsuit}$	Cytostatic	MKN-45	40 μg/mL	*C.* cfr.<br>*singaporensis*	NSW	[137]

Table S2: *Cont.*

Compound	Structure Elucidation	Chemistry Type	Drug Class	Biological Activity		Source of Organism	Province	Ref
				Cell/Enzyme/Micro-organism/Insect/Others	Activity			
(–)-15α,16-Dimethoxyspongi-13-en-19-oic acid **81**[β] [$C_{22}H_{34}O_5$]	IR, MS, NMR, $[\alpha]_D$, Mol. Mod	Spongiane	Antiosteoclastic	RAW264 (RANKL/TRAP)<br>RAW264 cell survival ratio	IC_{50} > 50 μM<br>98% (5 μM)	*S. ceylonensis*	NSW	[138]
(–)-Ceylonin A **82**[β] [$C_{22}H_{32}O_5$]	UV, IR, MS, NMR, $[\alpha]_D$, ECD	Spongiane	Antiosteoclastic	RAW264 (RANKL/TRAP)	70% (50 μM)	*S. ceylonensis*	NSW	[139]
(+)-Ceylonin B **83**[β] [$C_{23}H_{32}O_5$]	UV, IR, MS, NMR, $[\alpha]_D$	Spongiane	Antiosteoclastic	RAW264 (RANKL/TRAP)	<28% (50 μM)	*S. ceylonensis*	NSW	[139]
(–)-Ceylonin C **84**[β] [$C_{23}H_{32}O_5$]	UV, IR, MS, NMR, $[\alpha]_D$	Spongiane	Antiosteoclastic	RAW264 (RANKL/TRAP)	<28% (50 μM)	*S. ceylonensis*	NSW	[139]
(+)-Ceylonin D **85**[β] [$C_{23}H_{32}O_5$]	UV, IR, MS, NMR, $[\alpha]_D$	Spongiane	Antiosteoclastic	RAW264 (RANKL/TRAP)	28% (50 μM)	*S. ceylonensis*	NSW	[139]
(–)-Ceylonin E **86**[β] [$C_{23}H_{32}O_5$]	UV, IR, MS, NMR, $[\alpha]_D$	Spongiane	Antiosteoclastic	RAW264 (RANKL/TRAP)	47% (50 μM)	*S. ceylonensis*	NSW	[139]
(–)-Ceylonin F **87**[β] [$C_{23}H_{32}O_5$]	UV, IR, MS, NMR, $[\alpha]_D$	Spongiane	Antiosteoclastic	RAW264 (RANKL/TRAP)	31% (50 μM)	*S. ceylonensis*	NSW	[139]
(+)-Ceylonin G **88**[β] [$C_{20}H_{26}O_4$]	UV, MS, NMR, $[\alpha]_D$, ECD	Spongiane	Anticancer	USP7	IC_{50} > 50 μM	*S. ceylonensis*	NSW	[140]
(+)-Ceylonin H **89**[β] [$C_{20}H_{28}O_4$]	UV, MS, NMR, $[\alpha]_D$, ECD, Mol. Mod.	Spongiane	Anticancer	USP7	IC_{50} > 50 μM	*S. ceylonensis*	NSW	[140]
(–)-Ceylonin I **90**[β] [$C_{20}H_{28}O_4$]	UV, MS, NMR, $[\alpha]_D$, ECD, Mol. Mod.	Spongiane	Anticancer	USP7	IC_{50} > 50 μM	*S. ceylonensis*	NSW	[140]
(–)-18-*nor*-3,17-Dihydroxyspongia-3,13(16),14-trien-2-one **91**[β] [$C_{19}H_{24}O_4$]	UV, IR, MS, NMR, $[\alpha]_D$	Spongiane	Alzheimer	BACE1	IC_{50} < 100 μM	*Spongia* sp.	NSW	[141]
(–)-18-*nor*-3,5,17-Trihydroxyspongia 3,13(16), 14-trien-2-one **92**[β] [$C_{19}H_{24}O_5$]	UV, IR, MS, NMR, $[\alpha]_D$	Spongiane	Alzheimer	BACE1	IC_{50} < 100 μM	*Spongia* sp.	NSW	[141]
(–)-Spongiapyridine **93**[β] [$C_{20}H_{23}NO_4$]	UV, IR, MS, NMR, $[\alpha]_D$	Spongiane	Alzheimer	BACE1	IC_{50} < 100 μM	*Spongia* sp.	NSW	[141]
(–)-Ceylonamide A **94**[β] [$C_{28}H_{37}NO_3$]	UV, MS, NMR, $[\alpha]_D$, Mol. Mod	Spongiane	Antiosteoclastic	RAW264 (RANKL/TRAP)<br>RAW264 cell survival ratio	IC_{50} = 13 μM<br>100% (5 μM)	*S. ceylonensis*	NSW	[138]

Table S2: *Cont.*

Compound	Structure Elucidation	Chemistry Type	Drug Class	Biological Activity: Cell/Enzyme/Micro-organism/Insect/Others	Biological Activity: Activity	Source of Organism	Province	Ref
(–)-Ceylonamide B **95**[β] [$C_{25}H_{39}NO_3$]	UV, MS, NMR, [α]D	Spongiane	Antiosteoclastic	RAW264 (RANKL/TRAP)<br>RAW264 cell survival ratio	IC_{50} = 18 μM<br>87% (5 μM)	*S. ceylonensis*	NSW	[138]
(–)-Ceylonamide C **96**[β] [$C_{24}H_{37}NO_3$]	UV, MS, NMR, [α]D	Spongiane	Antiosteoclastic	RAW264 (RANKL/TRAP)<br>RAW264 cell survival ratio	IC_{50} > 50 μM<br>100% (5 μM)	*S. ceylonensis*	NSW	[138]
(–)-Ceylonamide D **97**[β] [$C_{25}H_{39}NO_3$]	UV, MS, NMR, [α]D	Spongiane	Antiosteoclastic	RAW264 (RANKL/TRAP)<br>RAW264 cell survival ratio	IC_{50} > 50 μM<br>100% (5 μM)	*S. ceylonensis*	NSW	[138]
(–)-Ceylonamide E **98**[β] [$C_{24}H_{37}NO_3$]	UV, MS, NMR, [α]D	Spongiane	Antiosteoclastic	RAW264 (RANKL/TRAP)<br>RAW264 cell survival ratio	IC_{50} > 50 μM<br>100% (5 μM)	*S. ceylonensis*	NSW	[138]
(–)-Ceylonamide F **99**[β] [$C_{20}H_{29}NO_3$]	UV, MS, NMR, [α]D	Spongiane	Antiosteoclastic	RAW264 (RANKL/TRAP)<br>RAW264 cell survival ratio	IC_{50} > 50 μM<br>100% (5 μM)	*S. ceylonensis*	NSW	[138]
Flexibilene **100**[β] [$C_{20}H_{32}$]	IR, MS, NMR, CT	Flexibilane[♣]	Undetm.	Undetm.	Undetm.	*S. flexibilis*	MLU	[112, 143]
(+)-Lobophytolide **101**[β] [$C_{20}H_{28}O_3$]	IR, MS, NMR, [α]D, X-ray	Cembrane	Undetm.	Undetm.	Undetm.	*L. cristagalli*	MLU	[144]
(–)-Crassolide **102**[β] [$C_{26}H_{34}O_9$]	UV, IR, MS, NMR, [α]D, CT	Cembrane	Ichthyotoxic<br>Cytotoxic	*L. reticulatus*<br>A549, KB<br>P-388<br>HT-29	LD_{50} = 7 mg/L<br>ED_{50} = 0.39 – 0.85 μg/mL<br>ED_{50} = 0.04 – 0.08 μg/mL<br>ED_{50} = 0.05 – 0.26 μg/mL	*L. crassum*	MLU	[145 – 148]
2-Hydroxycrassocolide E **103**[β] [$C_{20}H_{28}O_4$]	MS, NMR	Cembrane	Cytotoxic	MCF7	IC_{50} = 18.13 μg/mL	*Sarcophyton sp.*	NSW	[149]
(–)-2-Hydroxynephthenol **104**[β] [$C_{20}H_{34}O_2$]	IR, MS, NMR, [α]D, CT	Cembrane	Cytotoxic	KB, A549, HT-29, P-388	ED_{50} = 0.23 – 1.80 μg/mL	*L. viridis*	MLU	[150 – 152]
3,4,11,12-Diepoxycembrane A **105**[β] [$C_{20}H_{32}O_2$]	IR, MS, NMR, CT	Cembrane	Undetm.	Undetm.	Undetm.	*S. flexibilis*	MLU	[153]
(+)-7,8-Epoxy-7,8 dihydrocembrene C **106**[β] [$C_{20}H_{32}O$]	MS, NMR, [α]D	Cembrane	Cytostatic<br>Cytotoxic	HUVEC, K562<br>HeLa	GI_{50} = 38.9 ± 1.8 – 52.5 ± 0.8 μM<br>CC_{50} = 83.8 ± 0.8 μM	*S. ehrenbergi*	BLI	[154]
3,4-Epoxynephthenol acetate **107**[β] [$C_{22}H_{36}O_3$]	MS, NMR	Cembrane	Cytostatic	SF268, MCF7, H-460	GI_{50} > 100 μM	*Nephthea* sp.	JSCR	[155]

Table S2: Cont.

Compound	Structure Elucidation	Chemistry Type	Drug Class	Biological Activity: Cell/Enzyme/Micro-organism/Insect/Others	Biological Activity: Activity	Source of Organism	Province	Ref
(+)-Sinulariolide **108**[β] [$C_{20}H_{30}O_4$]	IR, MS, NMR, $[\alpha]_D$, CT, X-ray	Cembrane	Cytotoxic	KB, MCF7	ED_{50} = 7.6 – 16.9 μg/mL	*S. flexibilis*	MLU	[156 – 176]
				A549, P388, HT-29	ED_{50} = 3.0 – 3.9 μg/mL			
				HL-60	ED_{50} = 0.7 μg/mL			
				CCRF-CEM, DLD-1	IC_{50} > 20 μg/mL			
				Huh7, HepG2, Hep3B, HA22T	IC_{50} = 8.46 ± 0.05 – 16.52 ± 0.13 μg/mL			
				B. neritina, B. albicostatus	EC_{50} = 21 − 33.18 μg/mL			
			Anticancer	A549 (mitochondria path.)	25 μg/mL (nanoparticle hyaluronan/(+)-**116**)			
				A375 (cytotoxic)	1 − 20 μg/mL			
				A375 (anti-migratory)	21 – 72% (5 – 15 μg/mL)			
				A375 (mitochondrial apop.)	15 μg/mL (caspase dependent)			
				TSGH (cytostatic)	15 – 30 μM			
				TSGH (anti-migratory)	24 − 71% (10 – 25 μM)			
				TSGH (mitochondrial apop.), RT4, T24, BFTC905	10 – 15 μM (Caspase-dependent)			
				TSGH (p38MAPK-ATF2 activation)	10 – 15 μM			
				HA22T (mitochondrial and ER apop.)	Active PERK/eIF2α/ATF4/CHOP			
				HA22T (anti-migratory)	50% (8 μg/mL, 24 h) 78% (8 μg/mL, 48 h)			
				HA22T (MMP-2/-9)	8 – 10 μg/mL			
				HA22T (MAPK – P13K/Akt)	10 μg/mL			
				HA22T (GRB2, FAK)	10 μg/mL			
				TSGH-8301 (anti-migratory)	7.5 – 10 μM			
				TSGH-8301 (MMP-2/-9)	7.5 – 10 μM			
				TSGH-8301 (mTOR)	10 μM			
				TSGH-8301 (P13K, MMP-2/-9)	5 μM			
				TSGH-8301 (GRB2, MKK7, MKK3)	10 μM			

Table S2: Cont.

Compound	Structure Elucidation	Chemistry Type	Drug Class	Biological Activity		Source of Organism	Province	Ref
				Cell/Enzyme/Micro-organism/Insect/Others	Activity			
(+)-Sinulariolide **108**β [$C_{20}H_{30}O_4$]	IR, MS, NMR, [α]D, CT, X-ray	Cembrane	Toxic	*M. digitata*, *A. tenuis*.	> 5 μg/mL	*S. flexibilis*	MLU	[156–176]
				Coral bleaching (*S. flexibilis*, *L. compactum*)	Decreased 8%			
			Ecology	Size and domination	Increasing (+)-**108**			
			Anti-inflammatory	RAW 264.7 (LPS/iNOS, COX-2)	47.7 ± 6.3 − 52.2 ± 5.1% (10 μM)			
				DC (LPS, phenotypes, cytokine secretion, mixlympocyte, CD40, CD80, CD86, TNF-α, IL-6, IL-12, NO, nuclear-κB path.)	Conc. dependent manner.			
			Cardiovascular	Atrial muscle (rat)	EC_{50} = 83.1 ± 1 μM (*ca.*)			
			Antibacterial	*B. subtilis*, *S. aureus*	10 μg/mL			
			Antifeedant	*G. affinis*	1−10%			
			Algacidal	*C. codii*	4.4 mg/L			
6ξ-Hydroxysinulariolide **109**β [$C_{20}H_{30}O_5$]	UV, IR, MS, NMR, CT	Cembrane	Undetm.	Undetm.	Undetm.	*S. flexibilis*	MLU	[62, 177]
(+)-11-Dehydrosinulariolide (5-Dehydrosinulariolide) **110**β [$C_{20}H_{28}O_4$]	MS, NMR, [α]D, CT, X-ray	Cembrane	Cytotoxic	KB	ED_{50} = 5.4 μg/mL	*S. flexibilis*	MLU	[62, 178]–182]
				Hep2, P-388, HT-29, Daoy, A549	ED_{50} = 1.58 − 2.9 μg/mL			
				HeLa	IC_{50} = 3.04 – 3.14 μg/mL			
				CCRF-CEM, DLD-1	IC_{50} > 20 μg/mL			
11-Dehydroxysinulariolide **111**β [$C_{20}H_{28}O_5$]	MS, NMR, CT	Cembrane	Undetm.	Undetm.	Undetm.	*S. flexibilis*	MLU	[62, 177]
(−)-11-*epi*-Sinulariolide acetate (5-*epi*-Sinulariolide) **112**β [$C_{20}H_{32}O_5$]	UV, IR, MS, NMR, [α]D, X-ray	Cembrane	Cytotoxic	HL-60, HT-29	ED_{50} = 0.8 − 1.9 μg/mL	*S. flexibilis*	MLU	[62, 177–181, 183–186, 188]
				HA22T	54% via. (9 μg/mL)			
			Anticancer	HA22T (anti-migratory)	2.66 – 7.98 μM			
				Apop. (mitochondria, ER)	Conc. dependent manner			
				MMP-2, MMP-9, uPA, TIMP-1, TIMP-2	1.33 – 7.98 μM (24 h)			
				P13K/AKT/mTOR	1.33 – 7.98 μM (conc. dependent manner)			

Table S2: Cont.

Compound	Structure Elucidation	Chemistry Type	Drug Class	Biological Activity: Cell/Enzyme/Micro-organism/Insect/Others	Biological Activity: Activity	Source of Organism	Province	Ref
(−)-11-*epi*-Sinulariolide acetate (5-*epi*-Sinulariolide) **112**$^{\beta}$ [$C_{20}H_{32}O_5$]	UV, IR, MS, NMR, [α]D, X-ray	Cembrane	Anti-inflammatory	RAW 264.7 (LPS/iNOS)<br>RAW 264.7 (LPS/COX-2)<br>Female Lewis rat (AIA)<br>Cathepsin K, MMP-9, TRAP, TNF-α (rat)	1.4 ± 1.74 − 84.89 ± 8.23% (1 – 50 μM)<br>42.13 ± 3.25 − 82.69 ± 1.63% (10 – 50 μM)<br>9 mg/kg every 2 days (day 7 – day 28)<br>Attenuated protein expression	*S. flexibilis*	MLU	[62, 177 – 181, 183 – 186, 188]
(−)-Decaryiol B **113**$^{\beta}$ [$C_{20}H_{34}O_3$]	NMR, MS, [α]D, CT	Cembrane♠	Cytostatic	C6, HeLa, H9c2	$IC_{50} \geq$ 200 μg/mL	*Lobophytum* sp.	NSW	[187]
(+)-Decaryiol C **114**$^{\beta}$ [$C_{22}H_{36}O_4$]	NMR, MS, [α]D, CT	Cembrane	Cytostatic	C6, HeLa, H9c2	$IC_{50} \geq$ 200 μg/mL	*Lobophytum* sp.	NSW	[187]
(−)-Decaryiol D **115**$^{\beta}$ [$C_{20}H_{34}O_4$]	NMR, MS, [α]D, CT	Cembrane	Cytostatic	C6<br>H9c2	IC_{50} = 40 ± 3 − 150 ± 15 μg/mL<br>$IC_{50} \geq$ 200 μg/mL	*Lobophytum* sp.	NSW	[187]
(−)-Danielid **116**$^{\beta}$ [$C_{25}H_{30}O_{10}$]	NMR, MS, [α]D	Cembrane	Undetm.	Undetm.	Undetm.	*S. asterolobata*	BLI	[188]
(+)-Sarcofuranocembranolide A **117**$^{\beta}$ [$C_{21}H_{24}O_7$]	UV, IR, NMR, MS, [α]D	Cembrane♣	Cytostatic<br>Anti-inflammatory	V79<br>RAW264.7 (LPS/TNF-α)	ED_{50} = 3.88 μg/mL<br>(10 μM)	*Sarcophyton* sp.	NSW	[189]
(−)-Sarcofuranocembranolide B **118**$^{\beta}$ [$C_{22}H_{28}O_7$]	UV, IR, NMR, MS, [α]D	Cembrane	Cytostatic<br>Anti-inflammatory	V79<br>RAW264.7 (LPS/TNF-α)	ED_{50} = 4.04 μg/mL<br>NA (10 μM)	*Sarcophyton* sp.	NSW	[189]
(+)-Chloroscabrolide A **119**$^{\beta}$ [$C_{19}H_{23}ClO_7$]	IR, NMR, MS, [α]D, Mol. Mod.	*Nor*-cembrane♠	Anti-inflammatory	J774 (LPS/iNOS)	NA (10 μM)	*Sinularia* sp.	NSW	[190]
(−)-Chloroscabrolide B **120**$^{\beta}$ [$C_{20}H_{27}ClO_8$]	IR, NMR, MS, [α]D, Mol. Mod.	*Nor*-cembrane	Anti-inflammatory	J774 (LPS/iNOS)	NA (10 μM)	*Sinularia* sp.	NSW	[190]
(−)-Prescabrolide C **121**$^{\beta}$ [$C_{19}H_{24}O_5$]	IR, NMR, MS, [α]D, Mol. Mod.	*Nor*-cembrane	Anti-inflammatory	J774 (LPS/iNOS)	NA (10 μM)	*Sinularia* sp.	NSW	[190]
(+)-Xeniolide F **122**$^{\beta}$ [$C_{20}H_{28}O_4$]	UV, IR, NMR, MS, [α]D	Xenicane	Cytotoxic	P-388, A-549, HT-29, MEL-28	IC_{50} > 1 μg/mL	*Xenia* sp.	CSW	[191]
(+)-9-Hydroxyxeniolide F **123**$^{\beta}$ [$C_{20}H_{28}O_5$]	UV, IR, NMR, MS, [α]D	Xenicane	Cytotoxic	P-388, A-549, HT-29, MEL-28	IC_{50} > 1 μg/mL	*Xenia* sp.	CSW	[191]

Table S2: Cont.

Compound	Structure Elucidation	Chemistry Type	Drug Class	Biological Activity		Source of Organism	Province	Ref
				Cell/Enzyme/Micro-organism/Insect/Others	Activity			
(–)-Xenimanadin A **124**[β] [$C_{23}H_{36}O_5$]	UV, IR, NMR, MS, $[\alpha]_D$, CT	Xenicane[♣]	Cytotoxic	P-388	$IC_{50} > 20$ µg/mL	*Xenia* sp.	NSW	[192]
(+)-Xenimanadin B **125**[β] [$C_{23}H_{36}O_6$]	UV, IR, NMR, MS, $[\alpha]_D$	Xenicane	Cytotoxic	P-388	$IC_{50} > 20$ µg/mL	*Xenia* sp.	NSW	[192]
(+)-Xenimanadin C **126**[β] [$C_{23}H_{36}O_5$]	UV, IR, NMR, MS, $[\alpha]_D$	Xenicane	Cytotoxic	P-388	$IC_{50} > 20$ µg/mL	*Xenia* sp.	NSW	[192]
(+)-Xenimanadin D **127**[β] [$C_{23}H_{34}O_5$]	UV, IR, NMR, MS, $[\alpha]_D$	Xenicane	Cytotoxic	P-388	$IC_{50} > 20$ µg/mL	*Xenia* sp.	NSW	[192]
(+)-Niphatheolide A **128**[β] [$C_{20}H_{30}O_3$]	UV, IR, NMR, MS, $[\alpha]_D$, ECD	Niphathane[♣]	Anticancer	*E. coli* BL21 (DE3) cells transf. pGEX6P1-p53 or pGEX6P1-HDM2	$IC_{50} = 16$ µM	*N. olemda*	NSW	[63]

__Footnote:__ **1. Structure** ([ς]molecule isolated as a natural product for the first time and found also in semisynthetic compound before). **2. Statistic** (**LD_{50}** 50% of the lethal dose); **3. Activity** (**C6** murine glioma, **H9c2** murine cardiacmycoblasts, **J774** murine macrophage, **L5178Y** murine lymphoblastic leukemia, **P-388** murine leukemia lymphoma, **P388D1** macrophage-like murine lymphoma, **PC12** murine adrenal gland phaeochromocytoma, **V79** Chinese hamster fibroblast, **A375** human melanoma, **CCRF-CEM** human T cell acute lymphoblastic lymphoma, **Daoy** human desmoplastic cerebellar medulloblastoma, **DLD-1** human colorectal adenocarcinoma, **H-460** human lung carcinoma, **HA22T** human hepatocellular carcinoma, **HeLa** human cervical carcinoma, **Hep2** human hepatocarcinoma, **Huh7** human hepatocarcinoma, **HT-29** human colorectal adenocarcinoma, **HUVEC** human umbilical vein endothelial, **KB-3-1** human nasopharyngeal epidermoid carcinoma, **KB-C2** human nasopharyngeal epidermoid carcinoma, **KB-CV60** human nasopharyngeal epidermoid carcinoma, **MEL-28** human melanoma, **MKN-45** human gastric adenocarcinoma, **SF268** human glioblastoma, **TSGH, TSGH-8031, RT4**, **T24**, **BFTC** human urinary bladder carcinoma, **BACE1** beta-secretase 1, **TRAP** tartrate-resistant acid phosphatase, **USP7** ubiquitin-specific protease 7, **uPA** urokinase plasminogen activator, **CB** cytochalasin B, **CCI** chronic constriction injury, **FMLP** formyl-L-methionyl-L-leucyl-L-phenylanalanine, **IFN-γ** interferon gamma, **LPS** lipopolysaccharide, **MRP-1** multidrug resistance protein 1, **P-gp** P-glycoprotein, **RANKL** receptor activator of nuclear factor kappa-B ligand, **SA** superoxide anion, **ER** endoplasmic reticulum, **gen.** generated, **DC** dendritic cell, **AIA** adjuvant-induced arthritis, **path.** pathway, **via.** viability, **rel.** release, **transf.** transformed).

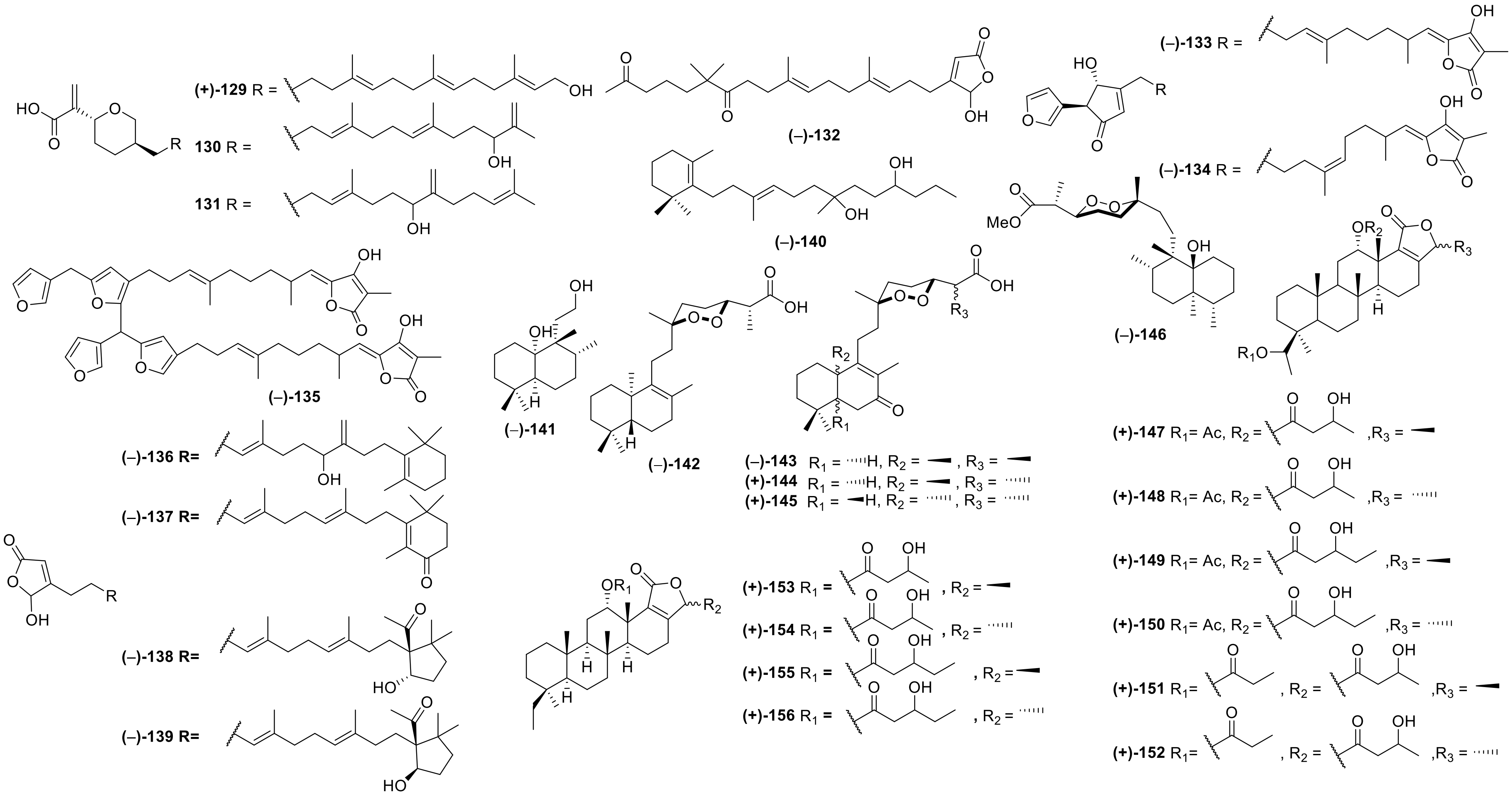

Figure S3: *Cont.*

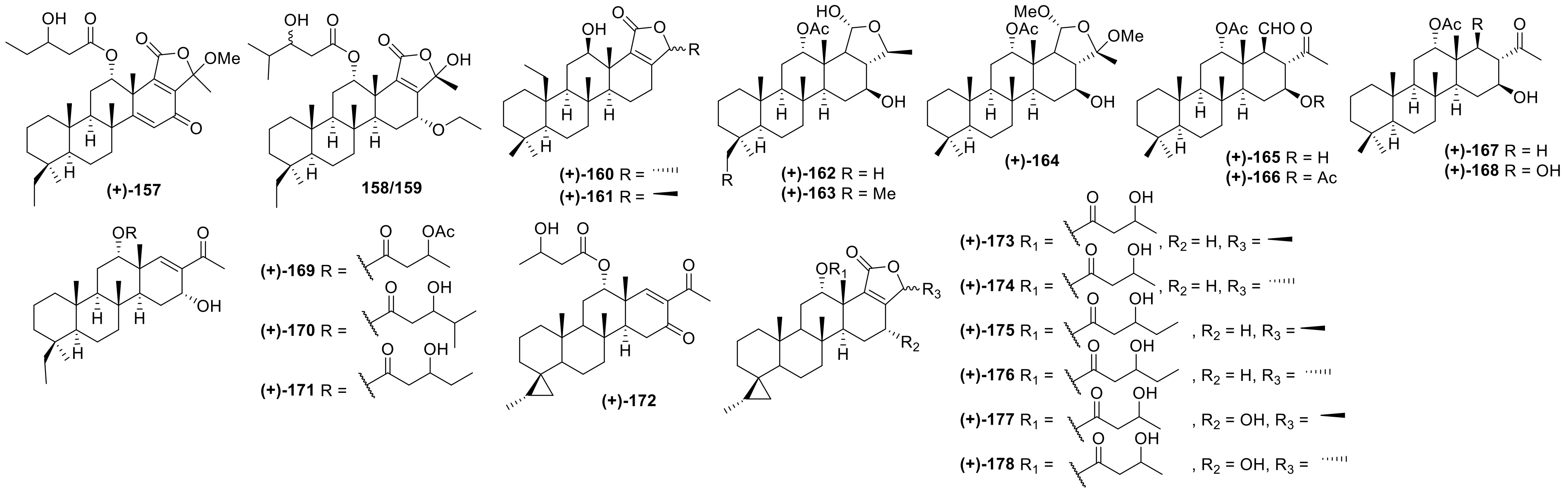

Figure S3: Structures of marine sesterterpenoids from Indonesian waters found in 1970–2017.

Table S3: Marine sesterterpenoids from Indonesian waters in 1970–2017.

Compound	Structure Elucidation	Chemistry Type	Drug Class	Biological Activity		Source of Organism	Province	Ref
				Cell/Enzyme/Micro-organism/Insect/Others	Activity			
(+)-Barangcadoic acid **129**[β] [$C_{25}H_{40}O_4$]	IR, MS, NMR, $[\alpha]_D$	Acyclic	Cytotoxic	LoVo (mut. Ras, sens.)<br>CaCo (normal Ras, resist.)	$IC_{50} \approx$ 1–2 μg/mL<br>3–4-fold less activity	*Hippospongia* sp.	SSW	[193]
			Anticancer	RCE	$IC_{50} \approx$ 10 μg/mL			
Rhopaloic acid D **130**/E **131**[α,β] [$C_{24}H_{38}O_4$]	MS, NMR	Acyclic nor-sesterterpene	Cytotoxic	LoVo (mut. Ras, sens.)<br>CaCo (normal Ras, res.)	$IC_{50} \approx$ 1–2 μg/mL<br>3–4-fold less activity	*Hippospongia* sp.	SSW	[193]
			Anticancer	RCE	$IC_{50} \approx$ 10 μg/mL			
(–)-Achantholide C **132**[β] [$C_{25}H_{38}O_5$]	UV, MS, NMR, $[\alpha]_D$	Acyclic	Undetm.	Undetm.	Undetm.	*Acantho-dendrilla* sp.	SSW	[194]
(–)-Sulawesin A **133**[α,β] [$C_{25}H_{30}O_6$]	UV, MS, NMR, $[\alpha]_D$	Acyclic[♣]	Anticancer	USP7	IC_{50} = 2.8 μM	*Psammocinia* sp.	NSW	[195]
(–)-Sulawesin B **134**[α,β] [$C_{25}H_{30}O_6$]	UV, MS, NMR, $[\alpha]_D$	Acyclic	Anticancer	USP7	IC_{50} = 4.6 μM	*Psammocinia* sp.	NSW	[195]
(–)-Sulawesin C **135**[β] [$C_{50}H_{58}O_{10}$]	UV, MS, NMR, $[\alpha]_D$	Acyclic	Anticancer	USP7	Undetm.	*Psammocinia* sp.	NSW	[195]
(–)-Achantholide A **136**[β] [$C_{25}H_{38}O_4$]	UV, MS, NMR, $[\alpha]_D$	Monocyclic	Undetm.	Undetm.	Undetm.	*Acantho-dendrilla* sp.	SSW	[194]
(–)-Achantholide B **137**[β] [$C_{25}H_{36}O_4$]	UV, MS, NMR, $[\alpha]_D$	Monocyclic	Cytotoxic	L5178Y	ED_{50} > 10 μg/mL	*Acantho-dendrilla* sp.	SSW	[194]
			Antibacterial	*S. aureus*	NA (5 μg)<br>10 mm (10 μg)			
				B. subtilis	NA (5 μg)<br>12 mm (10 μg)			
				E. coli	NA (5 μg)<br>9 mm (10 μg)			
			Antifungal	*C. albicans*	NA (5 μg)<br>10 mm (10 μg)			
				C. herbarum	NA (5 μg)<br>10 mm (10 μg)			
(–)-Achantholide D **138**[β] [$C_{25}H_{38}O_5$]	UV, MS, NMR, $[\alpha]_D$	Monocyclic	Cytotoxic	L5178Y	ED_{50} > 10 μg/mL	*Acantho-dendrilla* sp.	SSW	[194]
(–)-Achantholide E **139**[β] [$C_{25}H_{38}O_5$]	UV, MS, NMR, $[\alpha]_D$	Monocyclic	Cytotoxic	L5178Y	ED_{50} = 7 μg/mL	*Acantho-dendrilla* sp.	SSW	[194]

Table S3: *Cont.*

Compound	Structure Elucidation	Chemistry Type	Drug Class	Biological Activity: Cell/Enzyme/Micro-organism/Insect/Others	Biological Activity: Activity	Source of Organism	Province	Ref
(−)-Diacardiol A **140**[β] [$C_{24}H_{44}O_2$]	MS, NMR, [α]D	Monocyclic	Cytotoxic	L5178Y<br>HeLa, PC12<br>*A. salina*	EC_{50} = 4.80 µg/mL<br>EC_{50} > 10 µg/mL<br>20 − 40 % (10 µg/mL, 24 − 48 h)	*D. megaspi-norhabdosa*	SSW	[124]
(−)-Euplectellodiol **141**[β] [$C_{16}H_{30}O_2$]	MS, NMR, [α]D	Bicyclic	Undetm.	Undetm.	Undetm.	*M. euplec-tellioides*	NSW	[123]
(−)-Diacarperoxide D **142**[β] [$C_{24}H_{40}O_4$]	MS, NMR, [α]D	Bicyclic	Cytotoxic	L5178Y<br>HeLa, PC12	EC_{50} < 0.10 µg/mL<br>EC_{50} = 0.17 − 0.80 µg/mL	*D. megaspi-norhabdosa*	SSW	[124]
(−)-Diacarperoxide E **143**[β] [$C_{24}H_{38}O_5$]	MS, NMR, [α]D	Bicyclic	Cytotoxic	L5178Y	EC_{50} < 3.0 µg/mL	*D. megaspi-norhabdosa*	SSW	[124]
(+)-Diacarperoxide F **144**[β] [$C_{24}H_{38}O_5$]	MS, NMR, [α]D	Bicyclic	Cytotoxic	L5178Y<br>HeLa, PC12	EC_{50} = 0.06 µg/mL<br>EC_{50} = 0.60 − 0.80 µg/mL	*D. megaspi-norhabdosa*	SSW	[124]
(+)-Diacarperoxide G **145**[β] [$C_{25}H_{40}O_5$]	MS, NMR, [α]D	Bicyclic	Cytotoxic	L5178Y<br>HeLa, PC12<br>*A. salina*	EC_{50} = 2 µg/mL<br>EC_{50} > 10 µg/mL<br>50 − 60% (10 µg/mL, 24 − 48 h)	*D. megaspi-norhabdosa*	SSW	[124]
(−)-Diacarperoxide S = (−)-Megaspinoxide A **146**[β] [$C_{25}H_{44}O_5$]	IR, MS, NMR, [α]D	Bicyclic	Antibacterial	*S. aureus* (AUMC No. B-54), *B. cereus* (AUMC No. B-52)	29 − 35 mm (100 µg)	*D. megaspi-norhabdosa*	SSW	[196, 197]
				E. coli (AUMC No. B-53), *P. aeruginosa* (AUMC No. B-73), *S. marcescens* (AUMC No. B-55)	4 − 6 mm (100 µg)			
			Antifungal	*C. albicans* (AUMC No. 418)	12 − 16 mm (100 µg)			
(+)-Honulactone C **147**[β] [$C_{33}H_{50}O_7$]	IR, MS, NMR, [α]D	Tetracyclic	Cytotoxic	P-388 (ATCC: CCL 46), HT-29 (ATCC: CCL 8), MEL-28 (ATCC: HTB 72)	IC_{50} = 1 µg/mL	*S. aliena*	EKM	[198]
(+)-Honulactone D **148**[β] [$C_{33}H_{50}O_7$]	IR, MS, NMR, [α]D, X-ray	Tetracyclic	Cytotoxic	P-388 (ATCC: CCL 46), HT-29 (ATCC: CCL 8), MEL-28 (ATCC: HTB 72)	IC_{50} = 1 µg/mL	*S. aliena*	EKM	[198]
(+)-Honulactone I **149**[β] [$C_{34}H_{52}O_7$]	IR, MS, NMR, [α]D	Tetracyclic	Cytotoxic	P-388 (ATCC: CCL 46), HT-29 (ATCC: CCL 8), MEL-28 (ATCC: HTB 72)	NA	*S. aliena*	EKM	[198]

Table S3: *Cont.*

Compound	Structure Elucidation	Chemistry Type	Drug Class	Biological Activity: Cell/Enzyme/Micro-organism/Insect/Others	Biological Activity: Activity	Source of Organism	Province	Ref
(+)-Honulactone J **150**[β] [$C_{34}H_{52}O_7$]	IR, MS, NMR, [α]D	Tetracyclic	Cytotoxic	P-388 (ATCC: CCL 46), HT-29 (ATCC: CCL 8), MEL-28 (ATCC: HTB 72)	NA	*S. aliena*	EKM	[198]
(+)-Honulactone K **151**[β] [$C_{34}H_{52}O_7$]	IR, MS, NMR, [α]D	Tetracyclic	Cytotoxic	P-388 (ATCC: CCL 46), HT-29 (ATCC: CCL 8), MEL-28 (ATCC: HTB 72)	NA	*S. aliena*	EKM	[198]
(+)-Honulactone L **152**[β] [$C_{34}H_{52}O_7$]	IR, MS, NMR, [α]D	Tetracyclic	Cytotoxic	P-388 (ATCC: CCL 46), HT-29 (ATCC: CCL 8), MEL-28 (ATCC: HTB 72)	NA	*S. aliena*	EKM	[198]
(+)-Phyllofolactone H **153**[β] [$C_{31}H_{48}O_5$]	MS, NMR, [α]D	Tetracyclic	Undetm.	Undetm.	Undetm.	*S. aliena*	EKM	[199]
(+)-Phyllofolactone I **154**[β] [$C_{31}H_{48}O_5$]	MS, NMR, [α]D	Tetracyclic	Undetm.	Undetm.	Undetm.	*S. aliena*	EKM	[199]
(+)-Phyllofolactone J **155**[β] [$C_{32}H_{50}O_5$]	MS, NMR, [α]D	Tetracyclic	Undetm.	Undetm.	Undetm.	*S. aliena*	EKM	[199]
(+)-Phyllofolactone K **156**[β] [$C_{32}H_{50}O_5$]	MS, NMR, [α]D	Tetracyclic	Undetm.	Undetm.	Undetm.	*S. aliena*	EKM	[199]
(+)-Phyllactone H **157**[β] [$C_{33}H_{48}O_7$]	UV, IR, MS, NMR, [α]D	Tetracyclic	Cytotoxic	MCF7, A549, HeLa, HL-7702	IC_{50} = 16 − 27 μM (*ca.*)	*P. papyrecea*	NSW	[200]
(+)-**158**/(+)-**159**[α,β] [$C_{35}H_{56}O_7$]	UV, MS, NMR, [α]D	Tetracyclic	Anticancer<br>Cytotoxic	RCE, CACO2<br>LoVo, PC3<br>MDA468	IC_{50} = 4.2 μg/mL<br>IC_{50} = 2.9 − 3.2 μg/mL<br>IC_{50} = 4.4 μg/mL	*C. foliascens*	SSW	[201]
(+)-Hyattellactone A **160**[β] [$C_{27}H_{42}O_3$]	UV, IR, MS, NMR, ECD, [α]D, Mol. Mod.	Tetracyclic	Antidiabetic<br>Cytotoxic	PTP1B<br>Jurkat	IC_{50} = 7.45 μM<br>NA (24.2 μM)	*Hyattella* sp.	NSW	[202]
(+)-Hyattellactone B **161**[β] [$C_{27}H_{42}O_3$]	UV, IR, MS, NMR, ECD, [α]D	Tetracyclic	Antidiabetic<br>Cytotoxic	PTP1B<br>Jurkat	42% (24.2 μM)<br>NA (24.2 μM)	*Hyattella* sp.	NSW	[202]
(+)-**162**[β] [$C_{28}H_{46}O_5$]	MS, NMR, [α]D	Tetracyclic	Cytotoxic	KB	30–95% (10 μg/mL)	*Phyllospongia* sp.	SSW	[203]
(+)-**163**[β] [$C_{29}H_{48}O_5$]	MS, NMR, [α]D	Tetracyclic	Cytotoxic	KB	30–95% (10 μg/mL)	*Phyllospongia* sp.	SSW	[203]
(+)-**164**[β] [$C_{30}H_{50}O_6$]	MS, NMR, [α]D	Tetracyclic	Cytotoxic	KB	30–95% (10 μg/mL)	*Phyllospongia* sp.	SSW	[203]
(+)-**165**[β] [$C_{28}H_{44}O_5$]	MS, NMR, [α]D, X-ray, CT	Tetracyclic	Cytotoxic	KB	30–95% (10 μg/mL)	*Phyllospongia* sp.	SSW	[203]

Table S3: *Cont.*

Compound	Structure Elucidation	Chemistry Type	Drug Class	Biological Activity: Cell/Enzyme/Micro-organism/Insect/Others	Biological Activity: Activity	Source of Organism	Province	Ref
(+)-**166**$^{\beta}$ [$C_{30}H_{46}O_6$]	MS, NMR, $[\alpha]_D$, CT	Tetracyclic	Cytotoxic	KB	30–95% (10 µg/mL)	*Phyllospongia* sp.	SSW	[203]
(+)-**167**$^{\beta}$ [$C_{27}H_{44}O_4$]	MS, NMR, $[\alpha]_D$	Tetracyclic	Cytotoxic	KB	30–95% (10 µg/mL)	*Phyllospongia* sp.	SSW	[203]
(+)-**168**$^{\beta}$ [$C_{27}H_{44}O_5$]	MS, NMR, $[\alpha]_D$	Tetracyclic	Cytotoxic	KB	30–95% (10 µg/mL)	*Phyllospongia* sp.	SSW	[203]
(+)-Phyllofenone C **169**$^{\beta}$ [$C_{32}H_{50}O_6$]	MS, NMR, $[\alpha]_D$	Tetracyclic	Undetm.	Undetm.	Undetm.	*S. aliena*	EKM	[199]
**170**$^{\beta}$ [$C_{32}H_{52}O_5$]	UV, MS, NMR, $[\alpha]_D$	Tetracyclic	Anticancer<br>Cytostatic	RCE<br>LoVo<br>CACO2, MDA468, PC3	IC_{50} = 38 µg/mL<br>IC_{50} = 7.6 µg/mL<br>IC_{50} = 3.4 – 3.8 µg/mL	*C. foliascens*	SSW	[201]
**171**$^{\beta}$ [$C_{31}H_{50}O_5$]	UV, MS, NMR, $[\alpha]_D$	Tetracyclic	Anticancer<br>Cytostatic	RCE<br>PC3, LoVo, CACO2	IC_{50} > 100 µg/mL<br>IC_{50} > 10 µg/mL	*C. foliascens*	SSW	[201]
(+)-Honu'enone **172**$^{\beta}$ [$C_{30}H_{44}O_5$]	MS, NMR, $[\alpha]_D$	Pentacyclic	Undetm.	Undetm.	Undetm.	*S. aliena*	EKM	[199]
(+)-Honulactone A **173**$^{\beta}$ [$C_{31}H_{46}O_5$]	IR, MS, NMR, $[\alpha]_D$	Pentacyclic	Cytotoxic	P-388 (ATCC: CCL 46), HT-29 (ATCC: CCL 8), MEL-28 (ATCC: HTB 72)	IC_{50} = 1 µg/mL	*S. aliena*	EKM	[199]
(+)-Honulactone B **174**$^{\beta}$ [$C_{31}H_{46}O_5$]	IR, MS, NMR, $[\alpha]_D$, X-ray	Pentacyclic	Cytotoxic	P-388 (ATCC: CCL 46), HT-29 (ATCC: CCL 8), MEL-28 (ATCC: HTB 72)	IC_{50} = 1 µg/mL	*S. aliena*	EKM	[198]
(+)-Honulactone E **175**$^{\beta}$ [$C_{32}H_{48}O_5$]	IR, MS, NMR, $[\alpha]_D$	Pentacyclic	Cytotoxic	P-388 (ATCC: CCL 46), HT-29 (ATCC: CCL 8), MEL-28 (ATCC: HTB 72)	NA	*S. aliena*	EKM	[198]
(+)-Honulactone F **176**$^{\beta}$ [$C_{32}H_{48}O_5$]	IR, MS, NMR, $[\alpha]_D$	Pentacyclic	Cytotoxic	P-388 (ATCC: CCL 46), HT-29 (ATCC: CCL 8), MEL-28 (ATCC: HTB 72)	NA	*S. aliena*	EKM	[198]
(+)-Honulactone G **177**$^{\beta}$ [$C_{31}H_{46}O_6$]	IR, MS, NMR, $[\alpha]_D$	Pentacyclic	Cytotoxic	P-388 (ATCC: CCL 46), HT-29 (ATCC: CCL 8), MEL-28 (ATCC: HTB 72)	NA	*S. aliena*	EKM	[198]
(+)-Honulactone H **178**$^{\beta}$ [$C_{31}H_{46}O_6$]	IR, MS, NMR, $[\alpha]_D$	Pentacyclic	Cytotoxic	P-388 (ATCC: CCL 46), HT-29 (ATCC: CCL 8), MEL-28 (ATCC: HTB 72)	NA	*S. aliena*	EKM	[198]

<u>Footnote:</u> 1. Activity (CaCo human colorectal adenocarcinoma, **CACO2** human colon carcinoma, **HL-7702** human hepatocarcinoma cell, **Jurkat** human T lymphoma, **MDA468** human breast adenocarcinoma, **PC3** human prostate carcinoma, **PTP1B** protein tyrosine phosphatase 1B, **RCE** ras converting enzyme, **migr.** migration, **mut**. mutated, **resist.** resistant).

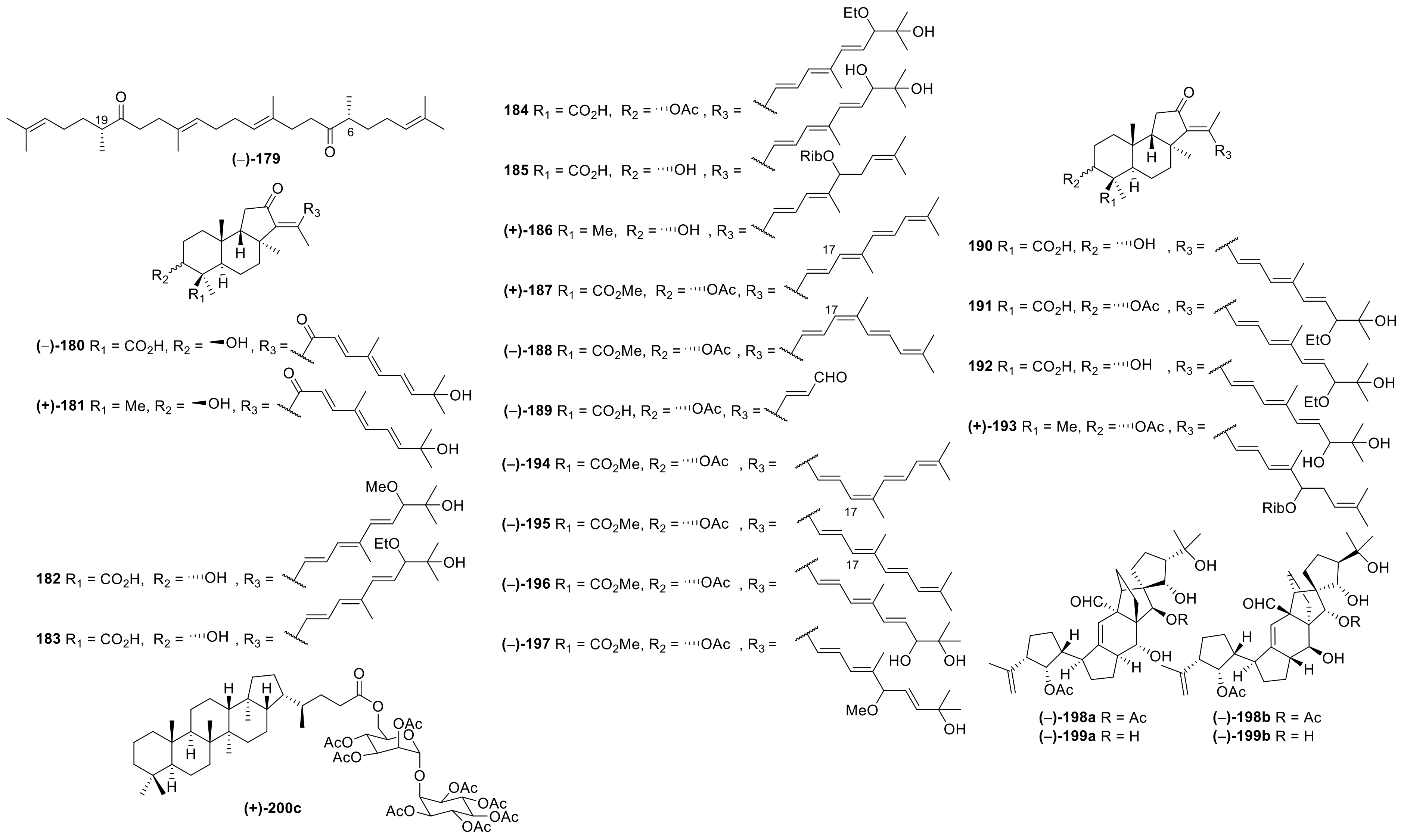

Figure S4: Structures of marine triterpenoids from Indonesian waters found in 1970–2017.

Table S4: Marine triterpenoids from Indonesian waters found in 1970–2017.

Compound	Structure Elucidation	Chemistry Type	Drug Class	Biological Activity		Source of Organism	Province	Ref
				Cell/Enzyme/Micro-organism/Insect/Others	Activity			
(–)-**179**[β] [$C_{30}H_{50}O_2$]	MS, NMR, $[\alpha]_D$	Squalene	Undetm.	Undetm.	Undetm.	*H. erectus*	SSW	[204, 205]
(–)-Globostellatic acid F **180**[β] [$C_{30}H_{42}O_6$]	MS, NMR, $[\alpha]_D$	Isomala-baricane	Cytotoxic	L5178Y	42 – 100% (3 – 10 µg/mL) ED_{50} = 10.36 nmol	*R. globostellata*	SSW	[206]
				HeLa, PC12	NA (3 – 10 µg/mL)			
(+)-Globostelletin **181**[β] [$C_{30}H_{44}O_4$]	MS, NMR, $[\alpha]_D$	Isomala-baricane	Cytotoxic	L5178Y	66 – 94% (3 – 10 µg/mL) ED_{50} = 5.34 nmol	*R. globostellata*	SSW	[206]
				HeLa, PC12	NA (3 – 10 µg/mL)			
				HeLa	ED_{50} > 60 nmol			
Globostellatic acid G **182**[α,β] [$C_{31}H_{46}O_6$]	UV, MS, NMR	Isomala-baricane	Cytotoxic	L5178Y	100% (3 – 10 µg/mL) ED_{50} = 0.39 nmol	*R. globostellata*	SSW	[206]
				HeLa	8 – 25% (3 – 10 µg/mL) ED_{50} = 46.69 nmol			
				PC12	18 – 42% (3 – 10 µg/mL) ED_{50} = 30.33 nmol			
Globostellatic acid I **183**[α,β] [$C_{32}H_{48}O_6$]	UV, MS, NMR	Isomala-baricane	Cytotoxic	L5178Y	100% (3 – 10 µg/mL) ED_{50} = 0.31 nmol	*R. globostellata*	SSW	[206]
				HeLa	8 – 23% (3 – 10 µg/mL) ED_{50} = 46.05 nmol			
				PC12	16 – 41% (3 – 10 µg/mL) ED_{50} = 28.07 nmol			
Globostellatic acid K **184**[α,β] [$C_{34}H_{50}O_7$]	UV, MS, NMR	Isomala-baricane	Cytotoxic	L5178Y	100% (3 – 10 µg/mL) ED_{50} = 8.28 nmol	*R. globostellata*	SSW	[206]
				HeLa, PC12	NA (3 – 10 µg/mL)			
Globostellatic acid M **185**[α,β] [$C_{30}H_{44}O_6$]	UV, MS, NMR	Isomala-baricane	Cytotoxic	L5178Y	100% (3 – 10 µg/mL) ED_{50} = 0.92 nmol	*R. globostellata*	SSW	[206]
				HeLa	42% (3 – 10 µg/mL) ED_{50} = 27.87 nmol			
				PC12	0 – 30% (3 – 10 µg/mL) ED_{50} = 27.52 nmol			

Table S4: *Cont.*

Compound	Structure Elucidation	Chemistry Type	Drug Class	Biological Activity: Cell/Enzyme/Micro-organism/Insect/Others	Biological Activity: Activity	Source of Organism	Province	Ref
(–)-3-*O*-Deacetyl-13*Z*-stelliferin riboside **186**[β] [$C_{35}H_{54}O_7$]	UV, MS, NMR, $[\alpha]_D$	Isomala-baricane	Cytotoxic	L5178Y<br>HeLa<br>PC12	100% (3 – 10 µg/mL) ED_{50} = 2.40 nmol<br>26 – 100% (3 – 10 µg/mL) ED_{50} = 8.14 nmol<br>33 – 54% (3 – 10 µg/mL) ED_{50} = 27.63 nmol	*R. globostellata*	SSW	[206]
(+)-13*Z*, 17*E*-Globostellatic acid X methyl ester **187**[β] [$C_{33}H_{46}O_5$]	MS, NMR, $[\alpha]_D$	Isomala-baricane	Cytostatic	HUVEC<br>KB-3-1, Neuro2A<br>K562	IC_{50} = 0.064 µM<br>IC_{50} = 3.1 – 3.2 µM<br>IC_{50} = 9 µM	*R. globostellata*	UEP	[207]
(–)-13*Z*, 17*Z*-Globostellatic acid X methyl ester **188**[β] [$C_{33}H_{46}O_5$]	MS, NMR, $[\alpha]_D$	Isomala-baricane	Cytostatic	HUVEC<br>KB-3-1, K562, Neuro2A	IC_{50} = 0.4 µM<br>IC_{50} = 7.9 – 22 µM	*R. globostellata*	UEP	[207]
(–)-Acetyljaspiferal E **189**[β] [$C_{24}H_{32}O_6$]	MS, NMR, $[\alpha]_D$	Isomala-baricane	Cytostatic	HUVEC<br>KB-3-1, K562, Neuro2A	IC_{50} = 2.2 µM<br>IC_{50} = 28–34 µM	*R. globostellata*	UEP	[207]
Globostellatic acid H **190**[α,β] [$C_{32}H_{48}O_6$]	UV, MS, NMR	Isomala-baricane	Cytotoxic	L5178Y<br>HeLa<br>PC12	100% (3 – 10 µg/mL) ED_{50} = 0.31 nmol<br>8 – 23% (3 – 10 µg/mL) ED_{50} = 46.05 nmol<br>41% (10 µg/mL)	*R. globostellata*	SSW	[206]
Globostellatic acid J **191**[α,β] [$C_{34}H_{50}O_7$]	UV, MS, NMR	Isomala-baricane	Cytotoxic	L5178Y<br>HeLa, PC12	100% (3 – 10 µg/mL) ED_{50} = 8.28 nmol<br>NA (3 – 10 µg/mL)	*R. globostellata*	SSW	[206]
Globostellatic acid L **192**[α,β] [$C_{30}H_{44}O_6$]	UV, MS, NMR	Isomala-baricane	Cytotoxic	L5178Y<br>HeLa<br>PC12	100% (3 – 10 µg/mL) ED_{50} = 0.92 nmol<br>0 – 42% (3 – 10 µg/mL) ED_{50} = 27.87 nmol<br>30% (10 µg/mL)	*R. globostellata*	SSW	[206]
(+)-13*E*-Stelliferin riboside **193**[β] [$C_{37}H_{56}O_8$]	UV, NMR, MS, $[\alpha]_D$	Isomala-baricane	Cytotoxic	L5178Y<br>HeLa<br>PC12	100% (3 – 10 µg/mL) ED_{50} = 0.22 nmol<br>0 – 56% (3 – 10 µg/mL) ED_{50} = 22.76 nmol<br>0 – 38% (10 µg/mL) ED_{50} = 21.54 nmol	*R. globostellata*	SSW	[206]

Table S4: *Cont.*

Compound	Structure Elucidation	Chemistry Type	Drug Class	Biological Activity: Cell/Enzyme/Micro-organism/Insect/Others	Biological Activity: Activity	Source of Organism	Province	Ref
(–)-13*E*, 17*Z*-Globostellatic acid X methyl ester **194**[β] [$C_{33}H_{46}O_5$]	MS, NMR, [α]D	Isomala-baricane	Cytostatic	HUVEC<br>KB-3-1, K562<br>Neuro2A	IC_{50} = 0.06 μM<br>IC_{50} = 18 – 22 μM<br>IC_{50} = 4.7 μM	*R. globostellata*	UEP	[207]
(–)-13*E*, 17*E*-Globostellatic acid X methyl ester **195**[β] [$C_{33}H_{46}O_5$]	MS, NMR, [α]D	Isomala-baricane	Cytostatic<br><br><br>Anti-migratory<br>Anticancer	HUVEC<br>KB-3-1, K562<br>Neuro2A<br>HUVEC<br>HUVEC (apop. caspase 3/7)	IC_{50} = 0.09 μM<br>IC_{50} = 14 – 23 μM<br>IC_{50} = 7.5 μM<br>1 μM<br>1 – 10 μM (48 h)	*R. globostellata*	UEP	[207]
(–)-Globostellatic acid F methyl ester **196**[β] [$C_{33}H_{48}O_7$]	MS, NMR, [α]D	Isomala-baricane	Cytostatic	HUVEC<br>KB-3-1, K562<br>Neuro2A	IC_{50} = 0.98 μM<br>IC_{50} = 8.6 – 12 μM<br>IC_{50} = 5.0 μM	*R. globostellata*	UEP	[207]
(–)-13*E*-Globostellatic acid B methyl ester **197**[β] [$C_{34}H_{50}O_7$]	MS, NMR, [α]D	Isomala-baricane	Cytostatic	HUVEC<br>KB-3-1, K562<br>Neuro2A	IC_{50} = 1.1 μM<br>IC_{50} = 6.5 – 9.3 μM<br>IC_{50} = 6.0 μM	*R. globostellata*	UEP	[207]
(–)-Vannusal A **198a**[β] [$C_{34}H_{48}O_8$]	UV, MS, NMR, ECD, [α]D, Mol. Mod., CT	Vannusane[♣]	Undetm.	Undetm.	Undetm.	*E. vannus*	NSW	[36 – 43]
(–)-Vannusal A **198b**[γ] [$C_{34}H_{48}O_8$]	CT	Vannusane	Undetm.	Undetm.	Undetm.	*E. vannus*	NSW	[36 – 43]
(–)-Vannusal B **199a**[β] [$C_{32}H_{46}O_7$]	UV, MS, NMR, ECD, [α]D, Mol. Mod., CT	Vannusane	Undetm.	Undetm.	Undetm.	*E vannus*	NSW	[36 – 43]
(–)-Vannusal B **199b**[γ] [$C_{32}H_{46}O_7$]	UV, MS, NMR, [α]D, X-ray, TS	Vannusane	Undetm.	Undetm.	Undetm.	*E vannus*	NSW	[36 – 43]
(+)-Plakohopanoid peracetate **200c**[ε] [$C_{37}H_{56}O_8$]	MS, NMR, [α]D, CT, ECD	Hopane	Undetm.	Undetm.	Undetm.	*P. cf lita*	NSW	[208]

Footnote: **1. Activity** (**Neuro 2A** murine neuroblastoma).

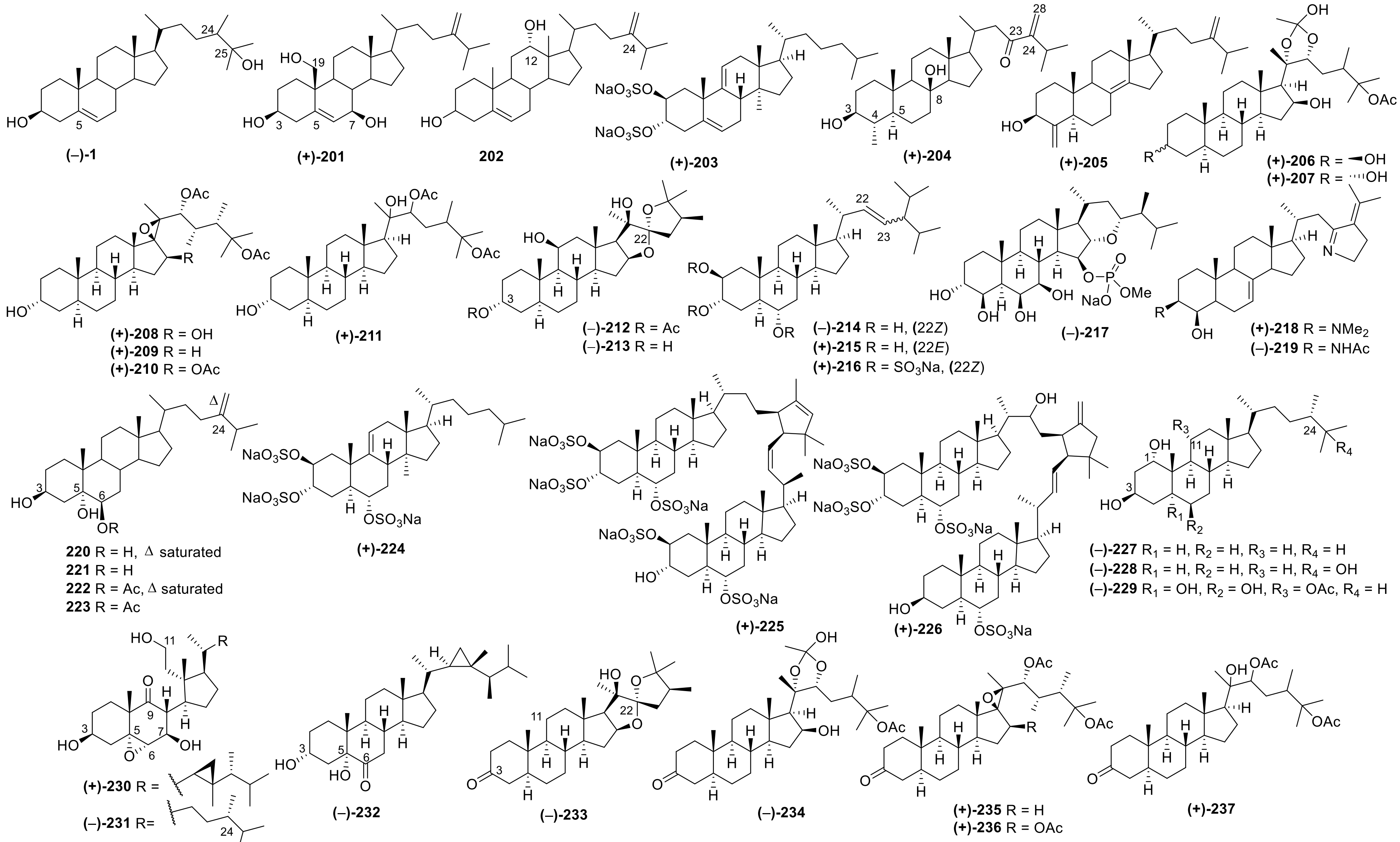

Figure S5: *Cont.*

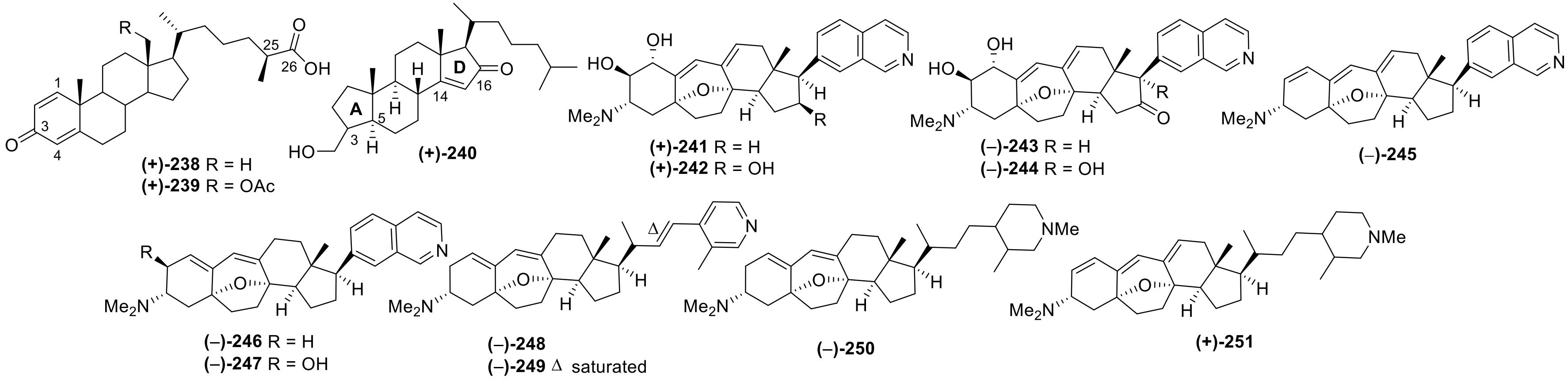

Figure S5: Structures of marine steroids from Indonesian waters found in 1970–2017.

Table S5: Marine steroids from Indonesian Indonesian waters found in 1970–2017.

Compound	Structure Elucidation	Chemistry Type	Drug Class	Biological Activity		Source of Organism	Province	Ref
				Cell/Enzyme/Micro-organism/Insect/Others	Activity			
(–)-25-Hydroxy-24ξ-methylcholesterol **1**[β] [$C_{28}H_{48}O_2$]	IR, MS, NMR, [α]D, CT	Δ^5 Sterol	Undetm.	Undetm.	Undetm.	*Nephtea sp.*	NST	[28]
(+)-24-Methylenecholest-5-en-3β,7β,19-triol **201**[β] [$C_{28}H_{46}O_3$]	IR, MS, NMR, [α]D, X-ray, CT	Δ^5 Sterol	Undetm.	Undetm.	Undetm.	*L. viridis*	MLU	[209]
12α-hydroxy-24-methylene cholesterol **202**[β] [$C_{28}H_{46}O_2$]	MS, NMR, [α]D	Δ^5 Sterol	Undetm.	Undetm.	Undetm.	*L. viridis*	MLU	[62]
(+)-Lembehsterol B **203**[β] [$C_{28}H_{44}Na_2O_8S_2$]	IR, MS, NMR, [α]D	Δ^5 Sterol	Anticancer	TP	IC_{50} = 45 μM	*P. strongylata*	NSW	[210]
(+)-4α-Methyl-3β, 8β-dihydroxy-5α-ergost-24(28)-en-23-one **204**[β] [$C_{29}H_{48}O_3$]	UV, IR, MS, NMR, [α]D X-ray	Δ^5 Sterol	Undetm.	Undetm.	Undetm.	*L. viridis*	UEP	[211]
(+)-Dehydroconicasterol **205**[β] [$C_{29}H_{46}O$]	UV, MS, NMR, [α]D	Δ^5 Sterol	Cytotoxic	C6, HeLa, H9c2	IC_{50} > 70 μM	*T. swinhoei*	NSW	[212]
(+)-Orthohippurinsterol A **206**[β] [$C_{32}H_{54}O_7$]	MS, NMR, [α]D	Δ^5 Sterol[♦]	Cytotoxic	P-388<br>A549, HT-29, MEL-28	IC_{50} = 2.5 μg/mL<br>IC_{50} = 5 μg/mL	*I. hippuris*	UEP	[213]
(+)-Orthohippurinsterol B **207**[β] [$C_{32}H_{54}O_7$]	MS, NMR, [α]D	Δ^5 Sterol	Cytotoxic	P-388, MEL-28<br>A549<br>HT-29	IC_{50} > 10 μg/mL<br>IC_{50} = 5 μg/mL<br>IC_{50} = 1 μg/mL	*I. hippuris*	UEP	[213]
(+)-Hippuristerol A **208**[β] [$C_{33}H_{54}O_7$]	MS, NMR, [α]D	Δ^5 Sterol	Cytotoxic	P-388, A549, HT-29, MEL-28	IC_{50} = 1 μg/mL	*I. hippuris*	UEP	[213]
(+)-Hippuristerol B **209**[β] [$C_{33}H_{54}O_6$]	MS, NMR, [α]D	Δ^5 Sterol	Cytotoxic	P-388, A549, HT-29, MEL-28	IC_{50} = 1.25 μg/mL	*I. hippuris*	UEP	[213]
(+)-Hippuristerol C **210**[β] [$C_{33}H_{56}O_8$]	MS, NMR, [α]D	Δ^5 Sterol	Undetm.	Undetm.	Undetm.	*I. hippuris*	UEP	[213]
(+)-Hippuristerol D **211**[β] [$C_{32}H_{54}O_6$]	MS, NMR, [α]D	Δ^5 Sterol	Cytotoxic	P-388, A549, HT-29, MEL-28	IC_{50} = 1 μg/mL	*I. hippuris*	UEP	[213]

Table S5: *Cont.*

Compound	Structure Elucidation	Chemistry Type	Drug Class	Biological Activity		Source of Organism	Province	Ref
				Cell/Enzyme/Micro-organism/Insect/Others	Activity			
(–)-3-Acetyl-22-*epi*-hippuristanol **212**[β] [$C_{30}H_{48}O_6$]	MS, NMR, $[\alpha]_D$	Δ^5 Sterol	Cytotoxic	P-388<br>A549, MEL-28<br>HT-29	IC_{50} = 1 µg/mL<br>IC_{50} = 0.125 µg/mL<br>IC_{50} = 0.5 µg/mL	*I. hippuris*	UEP	[213]
(–)-11-Dehydroxy-22-*epi*-hippuristanol **213**[β] [$C_{28}H_{46}O_4$]	MS, NMR, $[\alpha]_D$	Δ^5 Sterol	Cytotoxic	P-388, A549, HT-29, MEL-28	IC_{50} = 5 µg/mL	*I. hippuris*	UEP	[213]
(–)-Topsentinol K **214**[β] [$C_{30}H_{52}O_3$]	UV, IR, MS, NMR, $[\alpha]_D$	Δ^5 Sterol	Alzheimer	BACE1	NA	*Topsentia* sp.	EKM	[214]
(+)-Topsentinol L **215**[β] [$C_{30}H_{52}O_3$]	UV, IR, MS, NMR, $[\alpha]_D$	Δ^5 Sterol	Alzheimer	BACE1	NA	*Topsentia* sp.	EKM	[214]
(+)-Topsentinol K trisulfate **216**[β] [$C_{30}H_{49}Na_3O_{12}S_3$]	UV, IR, MS, NMR, $[\alpha]_D$	Δ^5 Sterol	Alzheimer	BACE1	IC_{50} = 1.2 µM	*Topsentia* sp.	EKM	[214]
(–)-Desulfohaplosamate **217**[β] [$C_{30}H_{50}NaO_9P$]	MS, NMR, $[\alpha]_D$	Δ^5 Sterol[♠]	Physicoactive (CB receptor ligand)	HEK-293 transf. CB_1 and CB_2	K_i hCB_1 = 19.47 µM<br>K_i hCB_2 = 2.82 µM	*Dasychalina* sp.	NSW	[215]
(+)-Lokysterolamine A **218**[β] [$C_{31}H_{50}N_2O$]	MS, NMR, $[\alpha]_D$	Δ^5 Oxidized sterol	Cytotoxic<br><br><br><br><br>Antibacterial<br>Antifungal	P-388, A549<br>HT-29<br>MEL-28<br>MLR<br>LcV<br>*B. subtilis*<br>*C. albicans*	IC_{50} = 0.5 µg/mL<br>IC_{50} = 1.0 µg/mL<br>IC_{50} = 5.0 µg/mL<br>0.13 mm (50 µg/disk)<br>>25.0 mm (50 µg/disk)<br>19 mm (50 µg/disk)<br>11 mm (50 µg/disk)	*Corticium* sp.	NSW	[216]
(–)-Lokysterolamine B **219**[β] [$C_{31}H_{48}N_2O_2$]	MS, NMR, $[\alpha]_D$	Δ^5 Oxidized sterol	Cytotoxic<br><br><br><br><br>Antibacterial<br>Antifungal	P-388, HT-29<br>A549<br>MEL-28<br>MLR<br>LcV<br>*B. subtilis*<br>*C. albicans*	IC_{50} = 1.0 µg/mL<br>IC_{50} = 0.5 µg/mL<br>IC_{50} > 2 µg/mL<br>0.48 mm (50 µg/disk)<br>>12.5 mm (50 µg/disk)<br>8 mm (50 µg/disk)<br>0 mm (50 µg/disk)	*Corticium* sp.	NSW	[216]
24ξ-Methylcholestane-3β,5α,6β–triol **220** [$C_{28}H_{50}O_3$]/**221**[α,β] [$C_{28}H_{48}O_3$]	IR, MS, NMR, CT	Δ^5 Oxidized sterol	Undetm.	Undetm.	Undetm.	*S. dissecta*	MLU	[217]

Table S5: *Cont.*

Compound	Structure Elucidation	Chemistry Type	Drug Class	Biological Activity: Cell/Enzyme/Micro-organism/Insect/Others	Biological Activity: Activity	Source of Organism	Province	Ref
24ξ-Methylcholestane-3β,5α,6β–triol-6-monoacetate **222** [$C_{30}H_{52}O_4$]/**223**^{α,β}[$C_{30}H_{50}O_4$]	IR, MS, NMR, CT	$Δ^5$ Oxidized steroid	Undetm.	Undetm.	Undetm.	*S. dissecta*	MLU	[217]
(+)-Lembehsterol A **224**^{β} [$C_{28}H_{45}Na_3O_{12}S_3$]	IR, MS, NMR, [α]D, CT	$Δ^5$ Oxidized steroid	Anticancer	TP	IC_{50} = 41 μM	*P. strongylata*	NSW	[210]
(+)-Manadosterol A **225**^{β} [$C_{54}H_{83}Na_5O_{21}S_5$]	IR, MS, NMR, [α]D	$Δ^5$ Oxidized steroid♠	Anticancer	Ubc13-Uev1A	IC_{50} = 0.09 μM	*L. fibrosa*	NSW	[218]
(+)-Manadosterol B **226**^{β} [$C_{54}H_{84}Na_4O_{18}S_4$]	IR, MS, NMR, [α]D	$Δ^5$ Oxidized steroid	Anticancer	Ubc13-Uev1A	IC_{50} = 0.13 μM	*L. fibrosa*	NSW	[218]
(−)-24*S*-Methylcholestan-1α, 3β-diol **227**^{β} [$C_{28}H_{50}O_2$]	MS, NMR, [α]D	$Δ^5$ Oxidized steroid	Anti-inflammatory	HepG2 transf. pCMV-FXR, pSG5-RXR, p(hsp27)TKLUC, pCMV-β-gal	NA as antagonized FXR (10 μM **234** + 50 μM CDCA)	*Sinularia* sp.	NSW	[219]
(−)-24*S*-Methylcholestan-1α, 3β, 25-triol **228**^{β} [$C_{28}H_{50}O_3$]	MS, NMR, [α]D	$Δ^5$ Oxidized steroid	Anti-inflammatory	HepG2 transf. pCMV-FXR, pSG5-RXR, p(hsp27)TKLUC, pCMV-β-gal	NA as antagonized FXR (10 μM **235** + 50 μM CDCA)	*Sinularia* sp.	NSW	[219]
(−)-24*S*-Methylcholestan-11-acetoxy-1α, 3β, 5α, 6β-tetraol **229**^{β} [$C_{30}H_{52}O_6$]	MS, NMR, [α]D	$Δ^5$ Oxidized steroid	Anti-inflammatory	HepG2 transf. with pCMV-FXR, pSG5-RXR, p(hsp27)TKLUC, pCMV-β-gal	NA as antagonized FXR (10 μM **236** + 50 μM CDCA)	*Sinularia* sp.	NSW	[219]
(+)-3β,7β,11-Trihydroxy-5α,6α-epoxy-9,11-secogorgostan-9-one **230**^{β} [$C_{30}H_{50}O_5$]	IR, MS, NMR, [α]D	$Δ^5$ Oxidized steroid♠	Cytotoxic	A2780<br>K562	IC_{50} = 6.3 μM<br>IC_{50} = 7.1 μM	*Lobophytum* sp.	NMU	[220]
(−)-24*S**,3β,11-Dihydroxy-5β,6β-epoxy-24-methyl-9,11-secocholestan-9-one **231**^{β} [$C_{28}H_{48}O_4$]	IR, MS, NMR, [α]D	$Δ^5$ Oxidized steroid♠	Cytotoxic	P-388, A549, HT-29, MEL-28	IC_{50} > 1 μg/mL	*P. violacea*	CSW	[221]
(−)-3α,5β-Dihydroxy-gorgostan-6-one **232**^{β} [$C_{30}H_{50}O_3$]	MS, NMR, [α]D	$Δ^5$ Oxidized steroid	Undetm.	Undetm.	Undetm.	*Sinularia* sp.	NSW	[219]
(−)-11-Dehydroxy-22-*epi*-hippuristan-3-one **233**^{β} [$C_{28}H_{44}O_4$]	MS, NMR, [α]D	C(3) ketone steroid♠	Cytotoxic	P-388, A549, HT-29, MEL-28	IC_{50} = 5 μg/mL	*I. hippuris*	UEP	[213]

Table S5: *Cont.*

Compound	Structure Elucidation	Chemistry Type	Drug Class	Biological Activity		Source of Organism	Province	Ref
				Cell/Enzyme/Micro-organism/Insect/Others	Activity			
(–)-Orthohippurinsterone A **234**$^{\beta}$ [$C_{32}H_{52}O_7$]	MS, NMR, [α]D	C(3) ketone steroid	Cytotoxic	P-388	IC$_{50}$ = 2.5 μg/mL	*I. hippuris*	UEP	[213]
				A549, HT-29, MEL-28	IC$_{50}$ = 5 μg/mL			
(+)-Hippuristerone B **235**$^{\beta}$ [$C_{33}H_{52}O_6$]	MS, NMR, [α]D	C(3) ketone steroid	Cytotoxic	P-388, A549, HT-29, MEL-28	IC$_{50}$ > 10 μg/mL	*I. hippuris*	UEP	[213]
(+)-Hippuristerone C **236**$^{\beta}$ [$C_{35}H_{54}O_8$]	MS, NMR, [α]D	C(3) ketone steroid	Undetm.	Undetm.	Undetm.	*I. hippuris*	UEP	[213]
(+)-Hippuristerone D **237**$^{\beta}$ [$C_{32}H_{52}O_6$]	MS, NMR, [α]D	C(3) ketone steroid	Cytotoxic	P-388, A549, HT-29, MEL-28	IC$_{50}$ = 1 μg/mL	*I. hippuris*	UEP	[213]
(+)-25*S*-3-Oxocholesta-1,4 dien-26-oic acid **238**$^{\beta}$ [$C_{27}H_{40}O_3$]	MS, NMR, [α]D	C(3) ketone steroid	Antibacterial	*S. aureus* IAM 12544T, *E coli* IAM 12119T	NA (100 μg/disk)	*Minabea* sp.	NSW	[222]
			Antifungal	*S. cerevisiae* IAM 14383T, *M. hiemalis* IAM 6088	NA (100 μg/disk)			
			Cytostatic	V79	NA (10 μM)			
			Cytotoxic	L1210	NA (50 μg/mL)			
(+)-25*S*-18-Acetoxy-3-oxocholesta-1,4-dien-26-oic acid **239**$^{\beta}$ [$C_{29}H_{42}O_5$]	MS, NMR, [α]D, CT	C(3) ketone steroid	Antibacterial	*S. aureus* IAM 12544T, *E coli* IAM 12119T	NA (100 μg/disk)	*Minabea* sp.	NSW	[222]
			Antifungal	*S. cerevisiae* IAM 14383T, *M. hiemalis* IAM 6088	NA (100 μg/disk)			
			Cytostatic	V79	NA (10 μM)			
			Cytotoxic	L1210	NA (50 μg/mL)			
(+)-3-(Hydroxymethyl)-A-nor-5α-cholest-14-en-16-one **240**$^{\beta}$ [$C_{27}H_{44}O_2$]	MS, NMR, [α]D	Degraded/con-tracted steroid	Undetm.	Undetm.	Undetm.	*A. carteri*	EKM	[223]
(+)-Cortistatin A **241**$^{\beta}$ [$C_{30}H_{36}N_2O_3$]	UV, MS, NMR, ECD, [α]D, X-ray	Degraded/ contracted steroid$^{♣}$	Cytostatic	HUVEC	IC$_{50}$ = 0.0018 μM GI$_{50}$ = 0.002 ± 0.001 μM	*C. simplex*	ENT	[64 – 72, 224]
				KB-3-1, K562, Neuro2A, NHDF	IC$_{50}$ = 6.0 − 7.0 μM			
				MCF7	GI$_{50}$ > 10 μM			
				SF268, IA9, PTX22, A8, NCI-H460	GI$_{50}$ = 4.429 ± 0.664 − 7.786 ± 0.001 μM			
			Antiangiogenic	bFGF/VEGF-induced HUVEC migr. and tube form.	2 nM			

Table S5: *Cont.*

Compound	Structure Elucidation	Chemistry Type	Drug Class	Biological Activity: Cell/Enzyme/Micro-organism/Insect/Others	Biological Activity: Activity	Source of Organism	Province	Ref
(+)-Cortistatin B **242**[β] [$C_{30}H_{36}N_2O_4$]	UV, MS, NMR, $[\alpha]_D$	Degraded/contracted steroid	Cytostatic	HUVEC<br>KB-3-1, K562, Neuro2A<br>NHDF	IC_{50} = 1.1 μM<br>IC_{50} = 120 − 200 μM<br>IC_{50} > 300 μM	*C. simplex*	ENT	[64 – 72, 224]
(–)-Cortistatin C **243**[β] [$C_{30}H_{34}N_2O_4$]	UV, MS, NMR, $[\alpha]_D$	Degraded/contracted steroid	Cytostatic	HUVEC<br>KB-3-1, Neuro2A<br>K562, NHDF	IC_{50} = 0.019 μM<br>IC_{50} = 150 − 180 μM<br>IC_{50} > 300 μM	*C. simplex*	ENT	[64 – 72, 224]
(–)-Cortistatin D **244**[β] [$C_{30}H_{34}N_2O_5$]	UV, MS, NMR, $[\alpha]_D$	Degraded/contracted steroid	Cytostatic	HUVEC<br>KB-3-1<br>K562, Neuro2A, NHDF	IC_{50} = 0.15 μM<br>IC_{50} = 55 μM<br>IC_{50} > 300 μM	*C. simplex*	ENT	[64 – 72, 224]
(–)-Cortistatin J **245**[β] [$C_{30}H_{34}N_2O$]	UV, MS, NMR, $[\alpha]_D$	Degraded/contracted steroid	Cytostatic	HUVEC<br>MCF7, IA9<br>SF268, NCI-H460, PTX22, A8	IC_{50} = 8 nM<br>GI_{50} = 0.070 ± 0.013 μM<br>GI_{50} = 27.439 − 43.22 μM<br>GI_{50} = 4.340 ± 0.161 − 8.538 ± 0.588 μM	*C. simplex*	ENT	[64 – 72, 224]
(–)-Cortistatin K **246**[β] [$C_{30}H_{36}N_2O$]	UV, MS, NMR, $[\alpha]_D$	Degraded/contracted steroid	Cytostatic	HUVEC	IC_{50} = 40 nM	*C. simplex*	ENT	[64 – 72, 224]
(–)-Cortistatin L **247**[β] [$C_{30}H_{36}N_2O_2$]	UV, MS, NMR, $[\alpha]_D$	Degraded/contracted steroid	Cytostatic	HUVEC	IC_{50} = 23 nM	*C. simplex*	ENT	[64 – 72, 224]
(–)-Cortistatin G **248**[β] [$C_{31}H_{42}N_2O$]	UV, MS, NMR, $[\alpha]_D$	Degraded/contracted steroid	Cytostatic	HUVEC	IC_{50} = 0.35 – 1.9 μM	*C. simplex*	ENT	[64 – 72, 224]

Table S5: *Cont.*

Compound	Structure Elucidation	Chemistry Type	Drug Class	Biological Activity: Cell/Enzyme/Micro-organism/Insect/Others	Biological Activity: Activity	Source of Organism	Province	Ref
(–)-Cortistatin H **249**[β] [$C_{31}H_{44}N_2O$]	UV, MS, NMR, $[\alpha]_D$	Degraded/contracted steroid	Cytostatic	HUVEC	IC_{50} = 0.35 – 1.9 μM	*C. simplex*	ENT	[64 – 72, 224]
(–)-Cortistatin E **250**[β] [$C_{32}H_{52}N_2O$]	UV, MS, NMR, $[\alpha]_D$	Degraded/contracted steroid	Cytostatic	HUVEC	IC_{50} = 0.35 – 1.9 μM	*C. simplex*	ENT	[64 – 72, 224]
(+)-Cortistatin F **251**[β] [$C_{30}H_{52}N_2O$]	UV, MS, NMR, $[\alpha]_D$	Degraded/contracted steroid	Cytostatic	HUVEC	IC_{50} = 0.35 – 1.9 μM	*C. simplex*	ENT	[64 – 72, 224]

Footnote: **1. Statistic** (***Ki*** inhibition constant); **2. Activity** (**L1210** murine lymphocytic leukemia, **MLR** murine mixed lymphocyte reaction, **A8** epothilone-resistant human ovarian carcinoma, **HEK-293** human embryonic kidney, **LcV** human leukocytoclastic vasculities, **NCI-H460** human nonsmall cell lung cancer, **NHDF** normal human dermal fibroblast, **PTX22** paclitaxel-resistant human ovarian carcinoma, **CB_1** cannabinoid receptor type 1, **CB_2** Cannabinoid receptor type 2, **FXR** farnesoid X receptor, **RXR** retinoid X receptor, **Ubc13** E2 ubiqutin-conjugating protein ubiquitin-conjugating enzyme complex, **bFGF** basic fibroblast growth factor, **TP** Thymidine phosphorylase, **CDCA** chenodeoxycholic acid, **VEGF** Vascular endothelial growth factor).

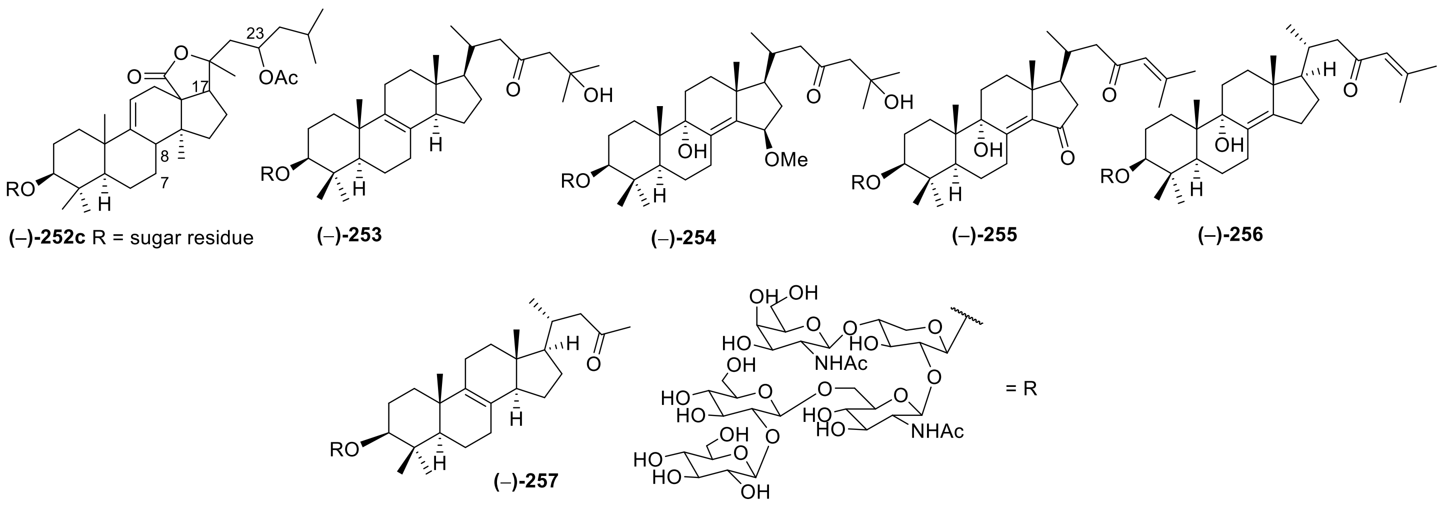

Figure S6: Structures of marine saponins from Indonesian waters found in 1970–2017.

Table S6: Marine saponins from Indonesian waters found in 1970–2017.

Compound	Structure Elucidation	Chemistry Type	Drug Class	Biological Activity		Source of Organism	Province	Ref
				Cell/Enzyme/Micro-organism/Insect/Others	Activity			
(–)-23ξ-Acetoxy-17-deoxy-7,8-dihydroholothurinogenin **252c**[ε] [$C_{32}H_{49}O_5R$]	IR, MS, NMR, [α]D, ORD, ECD, CT	Saponin	Undetm.	Undetm.	Undetm.	*S.chloronotus*	NST	[225]
(–)-Sarasinoside J **253**[β] [$C_{62}H_{102}N_2O_{27}$]	MS, NMR, [α]D	Saponin	Antifungal<br>Antibacterial<br>Cytotoxic<br>Cardiovascular<br>Antidiabetic	*S. cerevisiae*<br>*B. subtilis* DSM2109<br>K562, A549<br>Na^+/K^+–ATPase<br>PTP1B	13 mm (10 µg/disk)<br>9 mm (10 µg/disk)<br>LC_{50} = 10.3 – 20.8 µM<br>IC_{50} > 100 µg/mL<br>NA (IC_{50} = 15.2 – 16.0 µM)	*M. sarassinorum*	SSW	[226 – 228]
(–)-Sarasinoside K **254**[β] [$C_{63}H_{104}N_2O_{29}$]	MS, NMR, [α]D	Saponin	Undetm.	Undetm.	Undetm.	*M. sarassinorum*	SSW	[226]
(–)-Sarasinoside L **255**[β] [$C_{62}H_{98}N_2O_{28}$]	MS, NMR, [α]D	Saponin	Undetm.	Undetm.	Undetm.	*M. sarassinorum*	SSW	[226]
(–)-Sarasinoside M **256**[β] [$C_{62}H_{100}N_2O_{27}$]	MS, NMR, [α]D	Saponin	Cytotoxic<br>Cardiovascular	K562, A549<br>Na^+/K^+–ATPase	LC_{50} = 7.7 – 12.1 µM<br>LC_{50} > 100 µg/mL	*M. sarassinorum*	SSW	[226, 227]
(–)-Sarasinoside S **257**[β] [$C_{62}H_{96}N_2O_{26}$]	UV, IR, MS, NMR, [α]D	Saponin	Antidiabetic	PTP1B	NA (IC_{50} = 15.2 – 16.0 µM)	*Petrosia* sp.	NSW	[228]

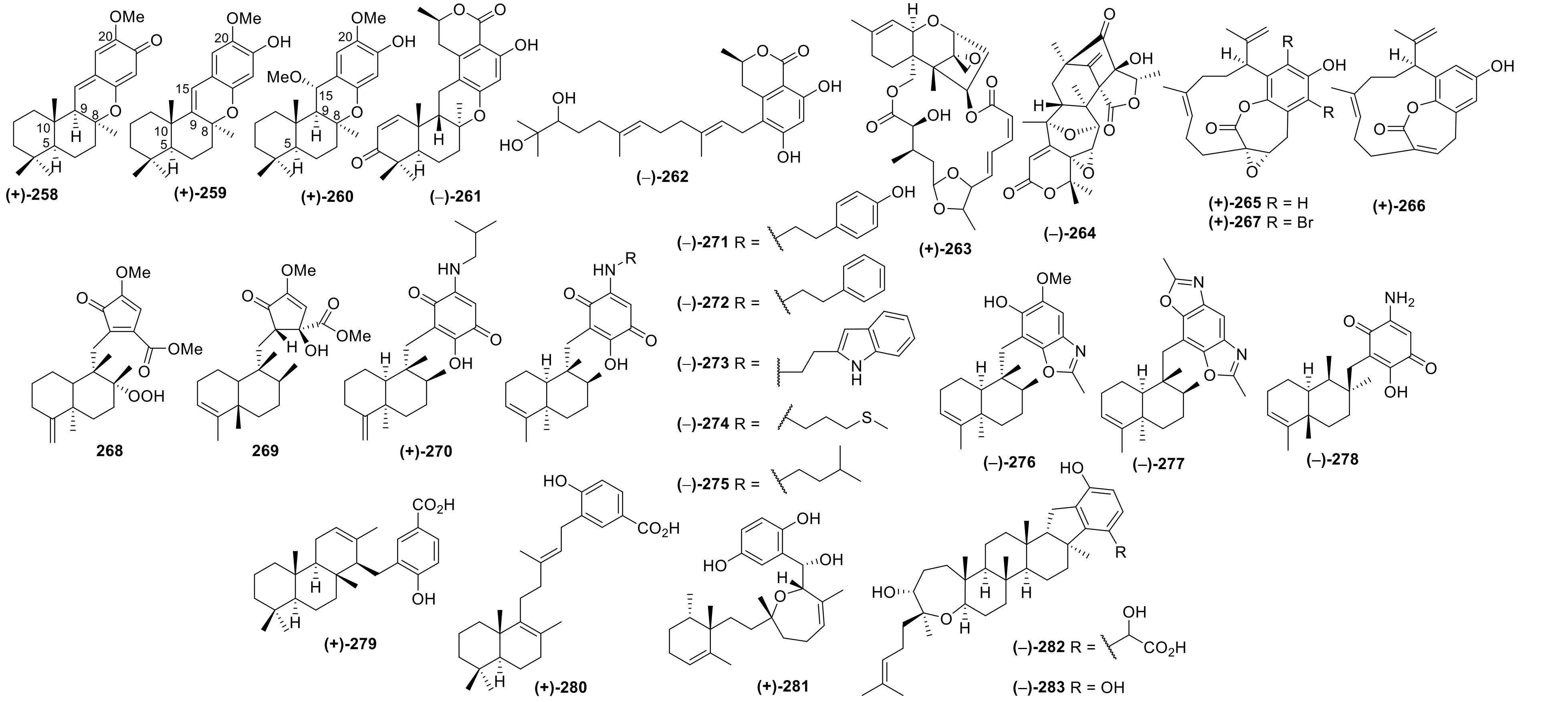

Figure S7: Structures of marine meroterpenoids from Indonesian waters found in 1970–2017.

Table S7: Marine meroterpenoids from Indonesian waters found in 1970–2017.

Compound	Structure Elucidation	Chemistry Type	Drug Class	Biological Activity		Source of Organism	Province	Ref
				Cell/Enzyme/Micro-organism/Insect/Others	Activity			
(+)-5*S*,8*S*,9*R*,10*S*-20-methoxypuupehenone **258**[β] [$C_{22}H_{30}O_3$]	MS, NMR, $[\alpha]_D$	Merosesqui-terpene	Undetm.	Undetm.	Undetm.	*Hyrtios* sp.	GTO	[229]
(+)-5*S*,8*S*, 10*S*-20-methoxy-9, 15-ene-puupehenol **259**[β] [$C_{22}H_{30}O_3$]	MS, NMR, $[\alpha]_D$	Merosesqui-terpene	Antiatherosclerotic	HepG2 transf. SR-B1	EC_{50} = 3.05 μM	*Hyrtios* sp.	GTO	[229, 230]
(+)-5*S*,8*S*,9*R*,10*S*-15,20-dimethoxy puupehenol **260**[β] [$C_{23}H_{34}O_4$]	MS, NMR, $[\alpha]_D$	Merosesqui-terpene	Undetm.	Undetm.	Undetm.	*Hyrtios* sp.	GTO	[229]
(–)-Verruculide A **261**[β] [$C_{25}H_{30}O_5$]	UV, IR, MS, NMR, ECD, $[\alpha]_D$, Mol. Mod.	Merosesqui-terpene	Antidiabetic	PTP1B	IC_{50} = 8.4 μM	*P. verruculo-sum* TPU1311 (symbiont) *P. aurata* (host)	NSW	[231]
(–)-Verruculide B **262**[β] [$C_{25}H_{36}O_6$]	UV, IR, MS, NMR, ECD, $[\alpha]_D$, Mol. Mod.	Merosesqui-terpene	Antidiabetic	PTP1B	40% (23.1 μM)	*P. verruculo-sum* TPU1311 (symbiont) *P. aurata* (host)	NSW	[231]
(+)-Roridin R **263**[β] [$C_{29}H_{38}O_9$]	UV, IR, MS, NMR, $[\alpha]_D$	Merosesqui-terpene	Cytotoxic	L1210	IC_{50} = 0.45 μM	*Myrothecium* sp. TUF 02F6 (symbiont) a sponge (host)	NSW	[232]
(–)-Citreonigrin A **264**[β] [$C_{25}H_{28}O_8$]	UV, MS, NMR, $[\alpha]_D$, X-ray	Merosesqui-terpene[♣]	Anticancer	Various protein kinases enzyme	NA	*P. citreonigrum* (symbiont) *P. purpurea* (host)	BLI	[233, 234]
(+)-Floresolide A **265**[β] [$C_{21}H_{24}O_4$]	MS, NMR, $[\alpha]_D$, X-ray	Merosesqui-terpene[♣]	Cytotoxic	KB		*Aplidium* sp.	ENT	[235, 236]
(+)-Floresolide B **266**[β] [$C_{21}H_{24}O_3$]	MS, NMR, $[\alpha]_D$	Merosesqui-terpene	Cytotoxic	KB	IC_{50} = 1 – 10 μg/mL	*Aplidium* sp.	ENT	[235, 236]
(+)-Floresolide C **267**[β] [$C_{21}H_{22}Br_2O_5$]	MS, NMR, $[\alpha]_D$	Merosesqui-terpene	Cytotoxic	KB		*Aplidium* sp.	ENT	[235, 236]

Table S7: *Cont.*

Compound	Structure Elucidation	Chemistry Type	Drug Class	Biological Activity: Cell/Enzyme/Micro-organism/Insect/Others	Biological Activity: Activity	Source of Organism	Province	Ref
Dactylospongenone G **268**[α,β] [$C_{23}H_{32}O_6$]	UV, MS, NMR	Merosesqui-terpene	Undetm.	Undetm.	Undetm.	*D. elegans*	MLU	[237]
Dactylospongenone H **269**[α,β] [$C_{25}H_{34}O_5$]	UV, MS, NMR	Merosesqui-terpene	Undetm.	Undetm.	Undetm.	*D. elegans*	MLU	[237]
(+)-5-*epi*-Smenospongorine **270**[β] [$C_{25}H_{37}NO_3$]	UV, IR, MS, NMR, $[\alpha]_D$	Merosesqui-terpene	Anticancer	Pseudo-perooxidase hemoglobin (K562)	2 μM	*D. elegans*	ENT	[238]
(–)-5-*epi*-Nakijiquinone S **271**[β] [$C_{29}H_{37}NO_4$]	UV, MS, NMR, $[\alpha]_D$	Merosesqui-terpene	Cytotoxic	L5178Y	IC_{50} = 1.7 μM	*D. metachromia*	MLU	[239]
(–)-5-*epi*-Nakijiquinone Q **272**[β] [$C_{29}H_{37}NO_3$]	UV, MS, NMR, $[\alpha]_D$	Merosesqui-terpene	Cytotoxic	L5178Y	IC_{50} = 1.1 μM	*D. metachromia*	MLU	[237, 239]
			Antibacterial	*S. aureus* ATCC 25923	MIC = 25 μM			
				S. aureus ATCC 700699, *E. faecium* ATCC 35667, *E. faecium* ATCC 700221	MIC = 50 μM			
				E. faecalis ATCC 29212, *E. faecalis* ATCC 51299	MIC = 100 μM			
(–)-5-*epi*-Nakijiquinone T **273**[β] [$C_{31}H_{38}N_2O_3$]	UV, MS, NMR, $[\alpha]_D$	Merosesqui-terpene	Cytotoxic	L5178Y	IC_{50} = 3.7 μM	*D. metachromia*	MLU	[239]
(–)-5-*epi*-Nakijiquinone U **274**[β] [$C_{25}H_{37}NO_3S$]	UV, MS, NMR, $[\alpha]_D$	Merosesqui-terpene	Cytotoxic	L5178Y	IC_{50} = 1.8 μM	*D. metachromia*	MLU	[239]
(–)-5-*epi*-Nakijiquinone N **275**[β] [$C_{26}H_{39}NO_3$]	UV, MS, NMR, $[\alpha]_D$	Merosesqui-terpene	Cytotoxic	L5178Y	IC_{50} = 1.3 μM	*D. metachromia*	MLU	[239]
			Anticancer	AKTK1, Aurora-B, METwt, NEK2, NEK6, PIM1, PLK1	IC_{50} = 29.9 − 73.2 μM			
				ALK	IC_{50} = 0.97 μM			
				ARK5, AXL, MEK1 wt, PRK1	IC_{50} > 100 μM			
				FAK, IGFI-R, SRC, VEGF-R2	IC_{50} = 1.94 − 3.03 μM			
(–)-5-*epi*-Nakijinol C **276**[β] [$C_{24}H_{33}NO_3$]	UV, MS, NMR, $[\alpha]_D$	Merosesqui-terpene[♣]	Cytotoxic	L5178Y	IC_{50} > 10.0 μM	*D. metachromia*	MLU	[239]
			Anticancer	AKT1, ARK5, Aurora-B, MEKI wt, MET wt, NEK6, PIM1, PLK1, PRK1	IC_{50} > 100 μM			
				ALK	IC_{50} = 3.38 μM			
				AXL, FAK, SRC	IC_{50} = 7.78 − 19.7 μM			
				IGF1-R, VEGF-R2	IC_{50} = 3.31 − 3.66 μM			

Table S7: *Cont.*

Compound	Structure Elucidation	Chemistry Type	Drug Class	Biological Activity: Cell/Enzyme/Micro-organism/Insect/Others	Biological Activity: Activity	Source of Organism	Province	Ref
(–)-5-*epi*-Nakijinol D **277**[β] [$C_{25}H_{32}N_2O_2$]	UV, MS, NMR, $[\alpha]_D$	Merosesqui-terpene	Cytotoxic	L5178Y	IC_{50} > 10.0 μM	*D. metachromia*	MLU	[239]
(–)-Dysideamine **278**[β] [$C_{21}H_{29}NO_3$]	UV, MS, NMR, $[\alpha]_D$	Merosesqui-terpene	Neuro disease	HT22<br>Neuro 2A	43% (HT22 surv. 10 μM) (IAA ind.)<br>40%, 3.0 μM; 25%, 10 μM (AChe incr.)	*Dysidea* sp.	UEP	[240]
(+)-Makassaric acid **279**[β] [$C_{27}H_{38}O_3$]	UV, MS, NMR, $[\alpha]_D$	Mero diterpene	Anti-inflammatory	MK-2	IC_{50} = 20 μM	*Acantho-dendrilla* sp.	SSW	[241, 242]
(+)-Subersic acid **280**[β] [$C_{27}H_{38}O_3$]	UV, MS, NMR, $[\alpha]_D$	Mero diterpene	Anti-inflammatory	MK-2	IC_{50} = 9.6 μM	*Acantho-dendrilla* sp.	SSW	[241, 242]
(+)-Halioxepine **281**[β] [$C_{26}H_{38}O_4$]	UV, IR, MS, NMR, $[\alpha]_D$, CT	Mero diterpene[♣]	Cytotoxic<br>Antioxidant	NBT-T2<br>DPPH	IC_{50} = 4.8 μg/mL<br>IC_{50} = 3.2 μg/mL	*Haliclona* sp.	SES	[243]
(–)-Haliclotriol A **282**[β] [$C_{38}H_{56}O_6$]	UV, IR, MS, NMR, $[\alpha]_D$	Mero triterpene[♣]	Cytotoxic	Various murine and human cancer	NA	*Haliclona* sp.	NMU	[244]
(–)-Haliclotriol B **283**[β] [$C_{36}H_{54}O_4$]	UV, IR, MS, NMR, $[\alpha]_D$	Mero triterpene	Antibacterial	*B. subtilis, S. aureus*	1 mg/disk	*Haliclona* sp.	NMU	[244]

Footnote: **1. Activity** (**HT22** murine hippocampal neuronal, **AChe** acetylcholinestrase, **AKT1** protein kinase B, **ALK** anaplastic lymphoma kinase, **ARK5** AMPK-related protein kinase 5, **AXL** tyrosine-protein kinase, **FAK** focal adhesion kinase, **IGF1-R** insulin like growth factor 1 receptor, **MEK1** mitogen-activated protein kinase kinase, **MET** tyrosine-protein kinase, **MK-2** mitogen-activated protein kinase-activated protein kinase 2, **NEK2** serine/threonine-protein kinase, **NEK6** serine/threonine-protein kinase, **PIM1** serine/threonine-protein kinase, **PLK1** serine/threonine-protein kinase**, PRK1** serine/threonine-protein kinase, **SRC** non-receptor tyrosine kinase, **VEGF-R2** vascular endothelial growth factor receptor 2, **DPPH** 1,1-diphenyl-2-picrylhydrazyl, **IAA** iodoacetic acid, **surv.** survived, **incr.** Increased, **ind.** induced).

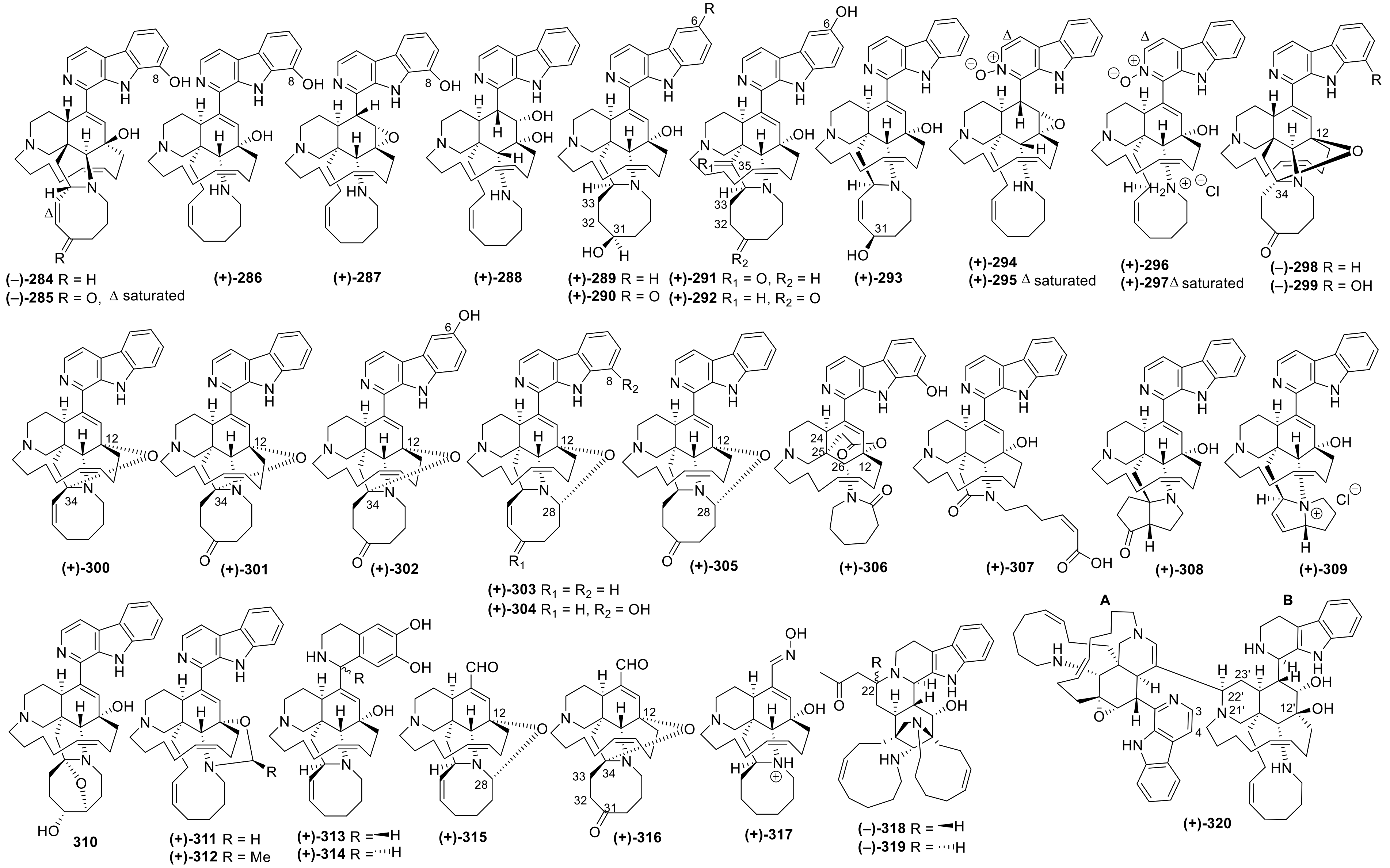

Figure S8: *Cont.*

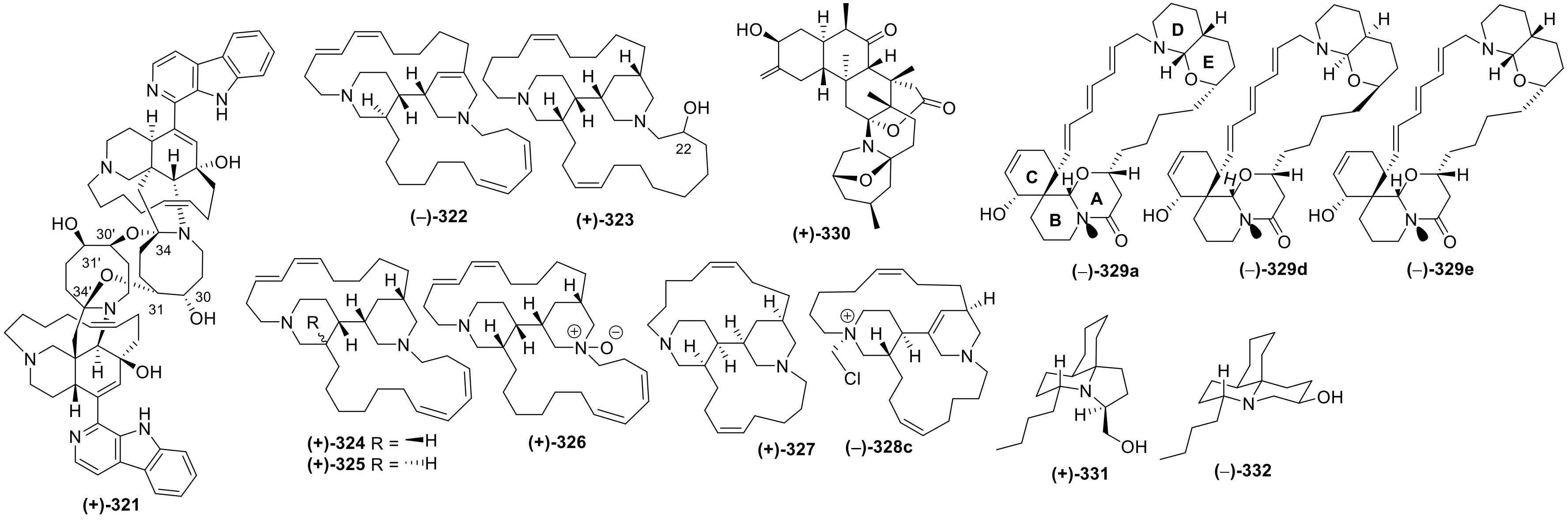

Figure S8: Structures of marine piperidine alkaloids from Indonesian waters found in 1970–2017.

Table S8: Marine piperidine alkaloids from Indonesian waters found in 1970–2017.

Compound	Structure Elucidation	Chemistry Type	Drug Class	Biological Activity		Source of Organism	Province	Ref
				Cell/Enzyme/Micro-organism/Insect/Others	Activity			
(–)-8-Hydroxymanzamine A **284**[β,η] [$C_{36}H_{44}N_4O_2$]	UV, IR, MS, NMR, [α]D	Piperidine Alkaloid	Antiparasite	*T. gondii*	71% (1 μM) [38% hc.]	A sponge (*Petrosiidae*)	NSW	[245 – 248]
				P. berghei	9 – 12 days (100 μmoles/kg, wo. tox.)			
			Antibacterial	*M. tuberculosis* H37Rv	MIC = 3.13 μg/mL			
			Antiinsecticidal	WCR	100% (2 mM)			
				WTPB	25% (2 mM)			
			Antifungal	*S. nodorum*	92% (10 μg/mL)			
				F. culmorum, P. recondita	0% (10 μg/mL)			
				P. infestans, P. grisei	22 – 41% (10 μg/mL)			
			Anti-inflammatory	B_2	IC_{50} > 30 μM (SA)			
(–)-Manzamine F **285**[β,η] [$C_{36}H_{44}N_4O_3$]	UV, IR, MS, NMR, [α]D	Piperidine Alkaloid	Antiparasite	*T. gondii*	37% (10 μM, wo. tox.)	A sponge (*Petrosiidae*)	NSW	[245, 248]
				P. berghei	NA (100 μmoles/kg, wo. tox.)			
			Antibacterial	*M. tuberculosis* H37Rv	98 – 99% (MIC < 12.5 μg/mL)			
			Antiinsecticidal	WCR	100% (3 mM)			
				WTPB	0% (3 mM)			
			Antifungal	*S. nodorum, P. infestans*	79 – 80 % (10 μg/mL)			
				P. grisei, F. culmorum	19 – 31% (10 μg/mL)			
				P. recondita	0% (10 μg/mL)			
(+)-8-Hydroxymanzamine J **286**[β,η] [$C_{36}H_{46}N_4O_2$]	UV, IR, MS, NMR, [α]D	Piperidine Alkaloid	Antibacterial	*S. aureus*, MRSA	IC_{50} = 3.0 – 5.0 μg/mL	*Acanthostro-ngylophora* sp.	NSW	[249]
				M. intracellulare	IC_{50} = 0.45 μg/mL			
			Antifungal	*C. neoformans*	IC_{50} = 3.5 μg/mL			
(+)-8-Hydroxymanzamine B **287**[β,η] [$C_{36}H_{46}N_4O_2$]	UV, IR, MS, NMR, [α]D	Piperidine Alkaloid	Undetm.	Undetm.	Undetm.	*Acanthostro-ngylophora* sp.	NSW	[250]
(+)-11-Hydroxymanzamine J **288**[β,θ] [$C_{36}H_{48}N_4O_2$]	UV, IR, MS, NMR, [α]D	Piperidine Alkaloid	Cytotoxic	A549, K562	LC_{50} = 6.2 – 8.2 μM	*Acanthostro-ngylophora* sp.	JSCR	[251]
			Antibacterial	*S. aureus* (ATCC 6538p), *P. hauseri* (NBRC 3851), *B. subtilis* (ATCC 6633), *K. rhizophila* (NBRC 12708)	MIC = 2.0 – 4.0 ng/mL			

Table S8: *Cont.*

Compound	Structure Elucidation	Chemistry Type	Drug Class	Biological Activity: Cell/Enzyme/Micro-organism/Insect/Others	Biological Activity: Activity	Source of Organism	Province	Ref
(+)-11-Hydroxymanzamine J **288**[β,θ] [$C_{36}H_{48}N_4O_2$]	UV, IR, MS, NMR, [α]D	Piperidine Alkaloid	Antibacterial<br>Hypercholesterolemic<br>Cardiovascular	*S. enterica* (ATCC 14028), *E. coli* (ATCC 35270)<br>Isocytrate lyase<br>Na^+/K^+-ATPase	MIC = 8.0 – 16.0 ng/mL<br>IC_{50} = 27 μM<br>IC_{50} > 150 μM	*Acanthostro-ngylophora* sp.	JSCR	[251]
(+)-32,33-Dihydro-31-hydroxymanzamine A **289**[β,θ] [$C_{36}H_{46}N_4O_2$]	UV, IR, MS, NMR, [α]D, Mol. Mod., CT, X-ray	Piperidine Alkaloid	Antiparasite<br>Cytostatic	*P. falciparum* (D6 clone), (W2 clone), *L. donovani*<br>V79	NA<br>NA (4.76 μg/mL)	A sponge (*Petrosiidae*)	NSW	[252]
(+)-32,33-Dihydro-6,31-dihydroxymanzamine A **290**[β,θ] [$C_{36}H_{46}N_4O_3$]	UV, IR, MS, NMR, [α]D, CT	Piperidine Alkaloid	Undetm.	Undetm.	Undetm.	A sponge (*Petrosiidae*)	NSW	[252]
(+)-32,33-dihydro-6-hydroxy manzamine A-35-one **291**[β,θ] [$C_{36}H_{44}N_4O_3$]	UV, IR, MS, NMR, [α]D	Piperidine Alkaloid	Antiparasite<br>Cytostatic	*P. falciparum* (D6 clone), (W2 clone), *L. donovani*<br>V79	NA<br>NA (4.76 μg/mL)	A sponge (*Petrosiidae*)	NSW	[252]
(+)-6-Hydroxymanzamine E **292**[β,η] [$C_{36}H_{44}N_4O_3$]	UV, IR, MS, NMR, [α]D	Piperidine Alkaloid	Antibacterial<br>Antifungal<br>Antiparasite<br>Cytostatic	*M. tuberculosis* H37Rv<br>*M. intracellulare*<br>*C. neoformans*<br>*P. falciparum* (D6 clone), (W2 clone)<br>*L. donovani*<br>V79	IC_{50} = 0.4 μg/mL<br>IC_{50} = 3.5 μg/mL<br>IC_{50} = 5.5 μg/mL<br>IC_{50} = 0.78 – 0.87 μg/mL<br>IC_{50} = 2.5 – 4.3 μg/mL<br>IC_{50} = 4.3 μg/mL	*Acanthostro-ngylophora* sp.	NSW	[249, 250]
(+)-31-Hydroxymanzamine A **293**[β,θ] [$C_{36}H_{44}N_4O_2$]	UV, IR, MS, NMR, [α]D, CT	Piperidine Alkaloid	Cytotoxic<br>Antibacterial<br>Hypercholesterolemic<br>Cardiovascular	A549, K562<br>*S. aureus* (ATCC 6538p), *P. hauseri* (NBRC 3851)<br>*B. subtilis* (ATCC 6633), *K. rhizophila* (NBRC 12708)<br>*S. enterica* (ATCC 14028)<br>*E. coli* (ATCC 35270)<br>Isocytrate lyase<br>Na^+/K^+-ATPase	LC_{50} = 5.8 – 7.2 μM<br>MIC = 13 – 25 ng/mL<br>MIC = 6.3 ng/mL<br>MIC = 1.6 ng/mL<br>MIC > 100 ng/mL<br>IC_{50} =140 μM<br>IC_{50} > 150 μM	*Acanthostro-ngylophora* sp.	JSCR	[251]
(+)-Manzamine B N-oxide **294**[β,η] [$C_{36}H_{46}N_4O_2$]	UV, IR, MS, NMR, [α]D	Piperidine Alkaloid	Cytotoxic	A549, K562	LC_{50} = 9.8 – 12.0 μM	*Acanthostro-ngylophora* sp.	JSCR	[251]

Table S8: *Cont.*

Compound	Structure Elucidation	Chemistry Type	Drug Class	Biological Activity: Cell/Enzyme/Micro-organism/Insect/Others	Biological Activity: Activity	Source of Organism	Province	Ref
(+)-Manzamine B N-oxide **294**$^{\beta,\eta}$ [$C_{36}H_{46}N_4O_2$]	UV, IR, MS, NMR, $[\alpha]_D$	Piperidine Alkaloid	Antibacterial	*S. aureus* (ATCC 6538p), *P. hauseri* (NBRC 3851), *E. coli* (ATCC 35270)	MIC > 100 ng/mL	*Acanthostro-ngylophora* sp.	JSCR	[251]
				B. subtilis (ATCC 6633), *K. rhizophila* (NBRC 12708)	MIC = 100 ng/mL			
				S. enterica (ATCC 14028)	MIC = 50 ng/mL			
			Hypercholesterolemic<br>Cardiovascular	Isocytrate lyase<br>Na^+/K^+-ATPase	IC_{50} > 150 μM			
			Cytotoxic	A549, K562	LC_{50} = 5.2 – 5.8 μM			
(+)-3,4-Dihydromanzamine B N-oxide **295**$^{\beta,\eta}$ [$C_{36}H_{48}N_4O_2$]	UV, IR, MS, NMR, $[\alpha]_D$	Piperidine Alkaloid	Antibacterial	*S. aureus* (ATCC 6538p), *P. hauseri* (NBRC 3851)	MIC = 13 – 25 ng/mL	*Acanthostro-ngylophora* sp.	JSCR	[251]
				B. subtilis (ATCC 6633), *K. rhizophila* (NBRC 12708), *S. enterica* (ATCC 14028)	MIC = 3.1 – 6.3 ng/mL			
				E. coli (ATCC 35270)	MIC > 100 ng/mL			
			Hypercholesterolemic	Isocytrate lyase	IC_{50} = 70 μM			
			Cardiovascular	Na^+/K^+-ATPase	IC_{50} > 150 μM			
			Cytotoxic	A549, K562	LC_{50} = 4.7 – 8.1 μM			
(+)-Manzamine J N-oxide-HCl **296**$^{\beta,\iota}$ [$C_{36}H_{47}N_4O_2$]	UV, IR, MS, NMR, $[\alpha]_D$	Piperidine Alkaloid	Antibacterial	*S. aureus* (ATCC 6538p), *S. enterica* (ATCC 14028)	MIC = 32 ng/mL	*Acanthostro-ngylophora* sp.	JSCR	[251]
				B. subtilis (ATCC 6633), *K. rhizophila* (NBRC 12708), *P. hauseri* (NBRC 3851), *E. coli* (ATCC 35270)	MIC > 100 ng/mL			
			Hypercholesterolemic	Isocytrate lyase	IC_{50} = 26 μM			
			Cardiovascular	Na^+/K^+-ATPase	IC_{50} > 150 μM			
			Cytotoxic	A549, K562	LC_{50} = 5.7 – 9.5 μM			
(+)-3,4-Dihydromanzamine J N-oxide-HCl **297**$^{\beta,\iota}$ [$C_{36}H_{49}N_4O_2$]	UV, IR, MS, NMR, $[\alpha]_D$	Piperidine Alkaloid	Antibacterial	*S. aureus* (ATCC 6538p), *B. subtilis* (ATCC 6633), *K. rhizophila* (NBRC 12708), *P. hauseri* (NBRC 3851)	MIC = 32 – 64 ng/mL	*Acanthostro-ngylophora* sp.	JSCR	[251]
				S. enterica (ATCC 14028), *E. coli* (ATCC 35270)	MIC > 100 ng/mL			

Table S8: *Cont.*

Compound	Structure Elucidation	Chemistry Type	Drug Class	Biological Activity: Cell/Enzyme/Micro-organism/Insect/Others	Biological Activity: Activity	Source of Organism	Province	Ref
(+)-3,4-Dihydromanzamine J N-oxide-HCl **297**[β,ι] [$C_{36}H_{49}N_4O_2$]	UV, IR, MS, NMR, $[\alpha]_D$	Piperidine Alkaloid	Hypercholesterolemic	Isocytrate lyase	$IC_{50} > 150$ µM	*Acanthostrongylophora* sp.	JSCR	[251]
			Cardiovascular	Na^+/K^+-ATPase	$IC_{50} = 110$ µM			
(–)-12,34-Oxamanzamine E **298**[β,η] [$C_{36}H_{42}N_4O_2$]	UV, IR, MS, NMR, $[\alpha]_D$	Piperidine Alkaloid	Antibacterial	*M. tuberculosis* H37Rv	MIC = 128 µg/mL	A sponge (*Petrosiidae*)	NSW	[246, 252]
			Antiparasite	*P. falciparum* (D6 clone), (W2 clone)	NA			
(–)-12,34-Oxamanzamine F **299**[β,η] [$C_{36}H_{42}N_4O_3$]	UV, IR, MS, NMR, $[\alpha]_D$	Piperidine Alkaloid	Antifungal	*C. neoformans*	NA	A sponge (*Petrosiidae*)	NSW	[246]
			Antiparasite	*P. falciparum* (D6 clone), (W2 clone)	$IC_{50} = 0.84 – 1.10$ µg/mL			
				L. donovani	NA			
			Cytostatic	V79	NA (4.76 µg/mL)			
			Antibacterial	*S. aureus*, MRSA, *M. intracelulare*	NA			
				M. tuberculosis H37Rv	MIC = 12.5 µg/mL			
(+)-12,34-Oxamanzamine A **300**[β,θ] [$C_{36}H_{42}N_4O$]	UV, IR, MS, NMR, $[\alpha]_D$	Piperidine Alkaloid	Antiviral	HIV-1	$IC_{50} = 14.9$ µM	A sponge (*Petrosiidae*)	NSW	[246]
			Antiparasite	*P. falciparum* (D6 clone)	$IC_{50} = 4.76$ µg/mL			
				(W2 clone)	NA			
(+)-12,34-Oxamanzamine E **301**[β,θ] [$C_{36}H_{42}N_4O_2$]	UV, IR, MS, NMR, $[\alpha]_D$	Piperidine Alkaloid	Antiparasite	*P. falciparum* (D6 clone), (W2 clone), *L. donovani*	NA	*Acanthostrongylophora* sp.	NSW	[249, 250, 252]
			Cytostatic	V79	NA (4.76 µg/mL)			
			Antibacterial	*S. aureus*, MRSA, *M. intracellulare*	NA			
			Antifungal	*C. neoformans*	NA			
			Antiviral	HIV-1	$EC_{50} = 17.5$ µM			
			Antiatherosclerotic	Human monocyte-derived macrophage	NA			
(+)-12,34-Oxa-6-hydroxymanzamine E **302**[β,η] [$C_{36}H_{42}N_4O_3$]	UV, IR, MS, NMR, $[\alpha]_D$	Piperidine Alkaloid	Antiparasite	*P. falciparum* (D6 clone), (W2 clone), *L. donovani*	NA	*Acanthostrongylophora* sp.	NSW	[250]

Table S8: *Cont.*

Compound	Structure Elucidation	Chemistry Type	Drug Class	Biological Activity: Cell/Enzyme/Micro-organism/Insect/Others	Biological Activity: Activity	Source of Organism	Province	Ref
(+)-12,28-Oxamanzamine A **303**$^{\beta,\theta}$ [$C_{36}H_{42}N_4O$]	UV, IR, MS, NMR, $[\alpha]_D$, Mol. Mod.	Piperidine Alkaloid	Antibacterial	*S. aureus*, MRSA, *M. intracellulare*	NA	A sponge (*Petrosiidae*)	NSW	[250, 253]
			Antiviral	HIV	EC_{50} = 22.2 μM			
			Antifungal	*C. neoformans*	NA			
			Antiparasite	*P. falciparum* (D6 clone), (W2 clone)	NA			
				L. donovani	IC_{50} = 7.8 μg/mL<br>IC_{90} = 50 μg/mL			
			Cytostatic	V79	NA (4.76 μg/mL)			
(+)-12,28-Oxa-8-hydroxymanzamine A **304**$^{\beta,\theta}$ [$C_{36}H_{42}N_4O_2$]	UV, IR, MS, NMR, $[\alpha]_D$	Piperidine Alkaloid	Antibacterial	*S. aureus*, MRSA, *M. intracellulare*	NA	A sponge (*Petrosiidae*)	NSW	[253]
			Antifungal	*C. neoformans*	NA			
(+)-12,28-Oxamanzamine E **305**$^{\beta,\theta}$ [$C_{36}H_{42}N_4O_2$]	UV, IR, MS, NMR, $[\alpha]_D$	Piperidine Alkaloid	Antiparasite	*P. falciparum* (D6 clone), (W2 clone)	NA	*Acanthostro-ngylophora* sp.	NSW	[250]
				L. donovani	IC_{50} = 18 μg/mL<br>IC_{90} = 40 μg/mL			
			Cytostatic	V79	NA (4.76 μg /mL)			
			Antibacterial	*S. aureus*, MRSA, *M. intracellulare*	NA			
			Antifungal	*C. neoformans*	NA			
(+)-Acantholactone **306**$^{\beta,\eta}$ [$C_{36}H_{42}N_4O_4$]	UV, MS, NMR, $[\alpha]_D$, Mol. Mod, ECD	Piperidine Alkaloid♠	Undetm.	Undetm.	Undetm.	*Acanthostro-ngylophora* sp.	NSW	[254]
(+)-Acantholactam **307**$^{\beta,\eta}$ [$C_{36}H_{42}N_4O_4$]	UV, MS, NMR, $[\alpha]_D$, ECD	Piperidine Alkaloid♠	Cytotoxic	HeLa	IC_{50} > 50 μM	*A. ingens*	NSW	[255]
			Anticancer	Chymotripsin-like activity	IC_{50} = 33 μM			
			Antiatherosclerotic	Human monocyte-derived macrophage	NA (20 μM)			
(+)-Acanthomanzamine C **308**$^{\beta,\eta}$ [$C_{36}H_{42}N_4O_2$]	UV, MS, NMR, $[\alpha]_D$	Piperidine Alkaloid♠	Undetm.	Undetm.	Undetm.	*A. ingens*	NSW	[256]
(+)-Kepulauamine A **309**$^{\beta,\iota}$ [$C_{36}H_{43}N_4O$]	UV, IR, MS, NMR, $[\alpha]_D$	Piperidine Alkaloid♦	Cytotoxic	A549, K562	LC_{50} = 4.6 – 7.2 μM	*Acanthostro-ngylophora* sp.	JSCR	[251]

Table S8: *Cont.*

Compound	Structure Elucidation	Chemistry Type	Drug Class	Biological Activity		Source of Organism	Province	Ref
				Cell/Enzyme/Micro-organism/Insect/Others	Activity			
(+)-Kepulauamine A **309**[β,ι]<br>[$C_{36}H_{43}N_4O$]	UV, IR, MS, NMR, $[\alpha]_D$	Piperidine Alkaloid	Antibacterial	*S. aureus* (ATCC 6538p), *P. hauseri* (NBRC 3851)<br>*B. subtilis* (ATCC 6633), *E. coli* (ATCC 35270)<br>*K. rhizophila* (NBRC 12708), *S. enterica* (ATCC 14028)	MIC = 8.0 ng/mL<br>MIC = 32 – 64 ng/mL<br>MIC = 16 ng/mL	*Acanthostro-ngylophora* sp.	JSCR	[251]
Pre-neo-Kauluamine **310**[β,η]<br>[$C_{36}H_{44}N_4O_3$]	MS, NMR	Piperidine Alkaloid	Hypercholesterolemic<br>Cardiovascular<br>Cytotoxic<br>Anticancer<br>Antiatherosclerotic	Isocytrate lyase<br>Na^+/K^+-ATPase<br>HeLa<br>Chymotripsin-like activity<br>Human monocyte-derived macrophage	IC_{50} >150 μM<br>IC_{50} >150 μM<br>IC_{50} = 16 μM<br>IC_{50} = 0.34 μM<br>91% (20 μM)	*Acanthostro-ngylophora* sp.	NSW	[255]
(+)-Acanthomanzamine D **311**[β,η]<br>[$C_{37}H_{46}N_4O$]	UV, MS, NMR, $[\alpha]_D$	Piperidine Alkaloid[♣]	Cytotoxic<br>Anticancer<br>Antiatherosclerotic	HeLa<br>Chymotripsin-like activity<br>Human monocyte-derived macrophage	IC_{50} = 15 μM<br>IC_{50} = 0.63 μM<br>73% (20 μM)	*A. ingens*	NSW	[256]
(+)-Acanthomanzamine E **312**[β,η]<br>[$C_{38}H_{48}N_4O$]	UV, MS, NMR, $[\alpha]_D$	Piperidine Alkaloid	Cytotoxic<br>Anticancer<br>Antiatherosclerotic	HeLa<br>Chymotripsin-like activity<br>Human monocyte-derived macrophage	IC_{50} > 20 μM<br>IC_{50} = 1.5 μM<br>61% (20 μM)	*A. ingens*	NSW	[256]
(−)-Acanthomanzamine A **313**[β,η]<br>[$C_{34}H_{47}N_3O_3$]	UV, MS, NMR, $[\alpha]_D$, ECD, Mol. Mod.	Piperidine Alkaloid[♣]	Cytotoxic<br>Anticancer<br>Antiatherosclerotic	HeLa<br>Chymotripsin-like activity<br>Human monocyte-derived macrophage	IC_{50} = 4.2 μM<br>IC_{50} = 4.1 μM<br>48% (20 μM)	*A. ingens*	NSW	[256]
(+)-Acanthomanzamine B **314**[β,η]<br>[$C_{34}H_{47}N_3O_3$]	UV, MS, NMR, $[\alpha]_D$, ECD, Mol. Mod.	Piperidine Alkaloid	Cytotoxic<br>Anticancer<br>Antiatherosclerotic	HeLa<br>Chymotripsin-like activity<br>Human monocyte-derived macrophage	IC_{50} = 5.7 μM<br>IC_{50} = 7.8 μM<br>73% (20 μM)	*A. ingens*	NSW	[256]
(+)-12,28-Oxaircinal A **315**[β,η]<br>[$C_{26}H_{36}N_2O_2$]	UV, IR, MS, NMR, $[\alpha]_D$	Piperidine Alkaloid	Undetm.	Undetm.	Undetm.	*Acanthostro-ngylophora* sp.	NSW	[250]

Table S8: *Cont.*

Compound	Structure Elucidation	Chemistry Type	Drug Class	Biological Activity: Cell/Enzyme/Micro-organism/Insect/Others	Biological Activity: Activity	Source of Organism	Province	Ref
(+)-31-Keto-12,34-oxa-32,33-dihydroircinal A **316**[β,η,θ] [$C_{26}H_{36}N_2O_3$]	UV, IR, MS, NMR, $[\alpha]_D$	Piperidine Alkaloid	Antibacterial	*S. aureus*, MRSA, *M. intracellulare*	NA	A sponge (*Petrosiidae*)	NSW	[253]
			Antifungal	*C. neoformans*	NA			
(+)-Ircinal E **317**[β,θ] [$C_{26}H_{42}N_3O_2$]	MS, NMR, $[\alpha]_D$	Piperidine Alkaloid	Cytotoxic	L5178Y	IC_{50} = 21.7 μM	*A. ingens*	MLU	[257]
(−)-Manadomanzamine A **318**[β,η] [$C_{39}H_{54}N_4O_2$]	UV, IR, MS, NMR, $[\alpha]_D$, ECD, Mol. Mod.	Piperidine Alkaloid♣	Antibacterial	*M. tuberculosis* H37Rv (ATCC 27294)	MIC = 1.9 μg/mL	*Acanthostro-ngylophora* sp.	NSW	[73]
			Antiviral	HIV-1 LAV	EC_{50} = 7.0 μg/mL			
			Antifungal	*C. albicans*	IC_{50} = 20 μg/mL			
				C. neoformans	NA			
			Cytotoxic	A549, H-116	IC_{50} = 2.5 – 5.0 μg/mL			
(−)-Manadomanzamine B **319**[β,η] [$C_{39}H_{54}N_4O_2$]	UV, IR, MS, NMR, $[\alpha]_D$, ECD, Mol. Mod.	Piperidine Alkaloid	Antibacterial	*M. tuberculosis* H37Rv (ATCC 27294)	MIC = 1.5 μg/mL	*Acanthostro-ngylophora* sp.	NSW	[73]
			Antiviral	HIV-1 LAV	EC_{50} = 16.5 μg/mL			
			Antifungal	*C. albicans*	NA			
				C. neoformans	IC_{50} = 3.5 μg/mL			
			Cytotoxic	A549, H-116	IC_{50} ≥ 5.0 μg/mL			
(+)-Kauluamine **320**[β,η] [$C_{72}H_{94}N_8O_3$]	IR, MS, NMR, $[\alpha]_D$	Piperidine Alkaloid♣	Immuno suppressive activity	MLR	IC_{50} = 1.57 μg/mL	*Prianos* sp.	NSW	[258]
				LcV, LcV/MLR	IC_{50} > 16.0 μg/mL			
(+)-*neo*-Kauluamine **321**[β,η] [$C_{72}H_{88}N_8O_6$]	UV, IR, MS, NMR, $[\alpha]_D$, Mol. Mod.	Piperidine Alkaloid♣	Cytotoxic	Human lung cancer, human colon carcinoma	IC_{50} = 1.0 μg/mL	A sponge (*Petrosiidae*)	NSW	[245, 249, 250, 255]
				A549, HeLa, K562	IC_{50} = 5.4 − 13 μM			
				V79	NA (4.7 μg/mL)			
			Anticancer	Chymotripsin-like activity	IC_{50} = 0.13 μM			
			Antiatherosclerotic	Human monocyte-derived macrophage	92% (20 μM)			
			Antibacterial	*M. tuberculosis* H37Rv (ATCC 27294)	MIC = 2.0 μg/mL			
				P. falciparum (D6 clone), (W2 clone)	IC_{50} = 1.70 – 2.80 μg/mL			
				L. donovani	IC_{50} = 4.2 – 8.2 μg/mL			

Table S8: *Cont.*

Compound	Structure Elucidation	Chemistry Type	Drug Class	Biological Activity: Cell/Enzyme/Micro-organism/Insect/Others	Biological Activity: Activity	Source of Organism	Province	Ref
(–)-Halicyclamine A **322**[β,η] [$C_{32}H_{50}N_2$]	IR, MS, NMR, $[\alpha]_D$	Piperidine Alkaloid	Antibacterial	*M. smegmatis* (aerobic), *M. smegmatis* (hypoxic)	MIC = 2.5 μg/mL	*Haliclona* sp.	PUA	[259–262]
				M. bovis BCG (aerobic), *M. bovis* BCG (hypoxic)	MIC = 1.0 μg/mL			
				M. tubercolosis (aerobic), *M. tubercolosis* (hypoxic)	MIC = 5.0 μg/mL			
				M. tubercolosis H37Rv ATCC25618, *M. tubercolosis* H37Rv STR ATCC35820 (streptomycin resist.), *M. avium* ATCC35712, *M. aurum* ATCC23366	MIC = 6.25 μg/mL			
				M.. tubercolosis H37Rv ETH ATCC35837 (ethambutol resist.), *M. tubercolosis* H37Rv INH ATCC35822 (isoniazid resist.), *M. tubercolosis* H37Rv RIF ATCC35838 (rifampicin), *M. tubercolosis* Kurono RIF ATCC35761, *M. kansasii* ATCC35775	MIC = 3.13 μg/mL			
				M. fortuitum ATCC9820	MIC = 25 μg/mL			
(+)-22-Hydroxyhaliclona cyclamine B **323**[β,η] [$C_{32}H_{56}N_2O$]	MS, NMR, $[\alpha]_D$	Piperidine Alkaloid	Cytotoxic	P-388	IC_{50} = 0.45 μg/mL	*Haliclona* sp.	ENT	[263]
			Antibacterial	*M. smegmatis* (aerobic)	MIC = 12.5 μg/mL			
				M. smegmatis (hypoxic), *M. bovis* BCG (aerobic)	MIC = 25 μg/mL			
				M. bovis BCG (hypoxic)	MIC = 50 μg/mL			
(+)-Tetradehydro haliclonacyclamine A **324**[β,η] [$C_{32}H_{52}N_2$]	MS, NMR, $[\alpha]_D$, X-ray	Piperidine Alkaloid	Cytotoxic	P-388	IC_{50} = 1.80 μg/mL	*Halichondria* sp.	BLI	[264]
(+)-2-*epi*-Tetradehydro haliclonacyclamine **325**[β,η] [$C_{32}H_{52}N_2$]	MS, NMR, $[\alpha]_D$	Piperidine Alkaloid	Undetm.	Undetm.	Undetm.	*Halichondria* sp.	BLI	[264]

Table S8: *Cont.*

Compound	Structure Elucidation	Chemistry Type	Drug Class	Biological Activity: Cell/Enzyme/Micro-organism/Insect/Others	Biological Activity: Activity	Source of Organism	Province	Ref
(+)-Tetradehydro haliclonacyclamine A mono-N-oxide **326**^{β,η} [$C_{32}H_{52}N_2O$]	MS, NMR, $[α]_D$	Piperidine Alkaloid	Undetm.	Undetm.	Undetm.	*Halichondria* sp.	BLI	[264]
(−)-Acanthocyclamine A **327**^{β,η} [$C_{26}H_{44}N_2$]	MS, NMR, $[α]_D$, X-ray	Piperidine-Alkaloid	Undetm.	Undetm.	Undetm.	*A. ingens*	SES	[265]
(−)-Chloromethyl halicyclamine B **328c**^{ε,η} [$C_{27}H_{44}ClN_2$]	MS, NMR, $[α]_D$, ECD, Mol. Mod.	Piperidine-Alkaloid	Anticancer	CK1δ/ε	IC_{50} = 6 μM	*A. ingens*	SSW	[266]
(−)-Upenamide **329a**^{β,η} [$C_{32}H_{46}N_2O_4$]	MS, NMR, $[α]_D$	Piperidine-Alkaloid♦	Undetm.	Undetm.	Undetm.	*Echinochalina* sp.	EKM	[267]
(−)-Upenamide **329d**^{η} [$C_{32}H_{46}N_2O_4$]	TS	Piperidine-Alkaloid	Undetm.	Undetm.	Undetm.			[268]
(−)-Upenamide **329e**^{η} [$C_{32}H_{46}N_2O_4$]	TS	Piperidine-Alkaloid	Undetm.	Undetm.	Undetm.			[268]
(+)-Lobozoanthamine **330**^{β,η} [$C_{30}H_{43}NO_5$]	IR, MS, NMR, $[α]_D$, CT	Piperidine-Alkaloid	Cytotoxic	AGS, C6	IC_{50} > 50 μM	*Lobophytum* sp.	NSW	[269]
(+)-Polycitorol A **331**^{β,θ} [$C_{17}H_{31}NO$]	IR, MS, NMR, $[α]_D$	Piperidine-Alkaloid	Undetm.	Undetm.	Undetm.	An ascidian (Polycito-ridae)	ENT	[270]
(+)-Polycitorol B **332**^{β,θ} [$C_{17}H_{31}NO$]	IR, MS, NMR, $[α]_D$	Piperidine-Alkaloid	Undetm.	Undetm.	Undetm.	An ascidian (Polycito-ridae)	ENT	[270]

Footnote: 1. Structure (^{η}molecule isolated as freebase, ^{θ}molecule isolated as TFA salt, ^{ι}molecule isolated as HCl salt); 2**. Activity** (**B2** murine neonatal brain microglia, **AGS** human stomach adenocarcinoma, **H-116** human colorectal adenocarcinoma, **HIV** human immunodeficiency virus, **D6 clone** *Plasmodium falciparum* chloroquine-sensitive, **W2 clone** *Plasmodium falciparum* chlorine-resistant, **MRSA** methicillin-resistant *Staphylococcus aureus*, **CK1δ/ε** protein kinase, **hc.** host cell, **wo.** without, **tox.** toxicity).

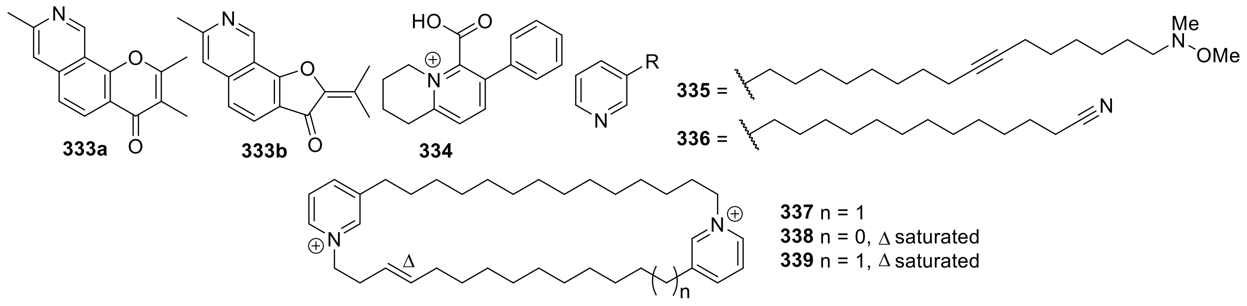

Figure S9: Structures of marine pyridine alkaloids from Indonesian waters found in 1970–2017.

Table S9: Marine pyridine alkaloids from Indonesian waters found in 1970–2017.

Compound	Structure Elucidation	Chemistry Type	Drug Class	Biological Activity		Source of Organism	Province	Ref
				Cell/Enzyme/Micro-organism/Insect/Others	Activity			
Aspergillitine **333a**[β,η] [$C_{15}H_{13}NO_2$]	UV, MS, NMR	Pyridine Alkaloid	Antibacterial<br>Antifungal	*B. subtilis*<br>*E. coli*<br>*S. cerevisiae*	7 – 8 mm (5 – 10 μg/disk)<br>NA<br>NA	*A. versicolor* (symbiont) *X. exigua* (host)	BLI	[74, 271, 272]
TMC-120B **333b**[δ,η] [$C_{15}H_{13}NO_2$]	TS	Pyridine Alkaloid	Undetm.	Undetm.	Undetm.			[74, 271, 272]
Clathryimine A **334**[β,κ] [$C_{16}H_{16}NO_2$]	UV, IR, MS, NMR, CT	Pyridine Alkaloid[♦]	Undetm.	Undetm.	Undetm.	*C. basilana*	UEP	[273]
N-methylniphatyne A **335**[β,η] [$C_{23}H_{38}N_2O$]	UV, IR, MS, NMR	Pyridine Alkaloid	Anticancer	PANC-1	IC_{50} = 16 μM (natural, Glu-def. Med.<br>IC_{50} = 17 μM (synthetic, Glu-def. Med.)<br>IC_{50} > 100 μM (natural, Gen. Glu. Med.)<br>IC_{50} > 100 μM (synthetic, Gen. Glu. Med.)	*Xestospongia* sp.	UEP	[274]
3-Dodecyl pyridine **336**[β,η] [$C_{18}H_{28}N_2$]	MS, NMR	Pyridine Alkaloid	Cytotoxic	HeLa, A549, MCF7	IC_{50} = 33.2 – 48.4 μM	*Haliclona* sp.	UEP	[275]
Haliclocyclamine A **337**[β,κ] [$C_{35}H_{56}N_2$]	UV, IR, MS, NMR	Pyridine Alkaloid	Antibacterial	*M. smegmatis*	10 – 17 mm (5 – 10 μg/disk)	*Haliclona* sp.	NSW	[276]
Haliclocyclamine B **338**[β,κ] [$C_{38}H_{64}N_2$]	UV, IR, MS, NMR	Pyridine Alkaloid	Antibacterial	*M. smegmatis*	7 – 10 mm (5 – 10 μg/disk)	*Haliclona* sp.	NSW	[276]
Haliclocyclamine C **339**[β,κ] [$C_{39}H_{66}N_2$]	UV, IR, MS, NMR	Pyridine Alkaloid	Antibacterial	*M. smegmatis*	9 – 13 mm (5 – 10 μg/disk)	*Haliclona* sp.	NSW	[276]

Footnote: **1. Activity** (**PANC-1** human pancreatic carcinoma, **Glu-def. Med** Glucose-deficient Medium, **Gen.** General).

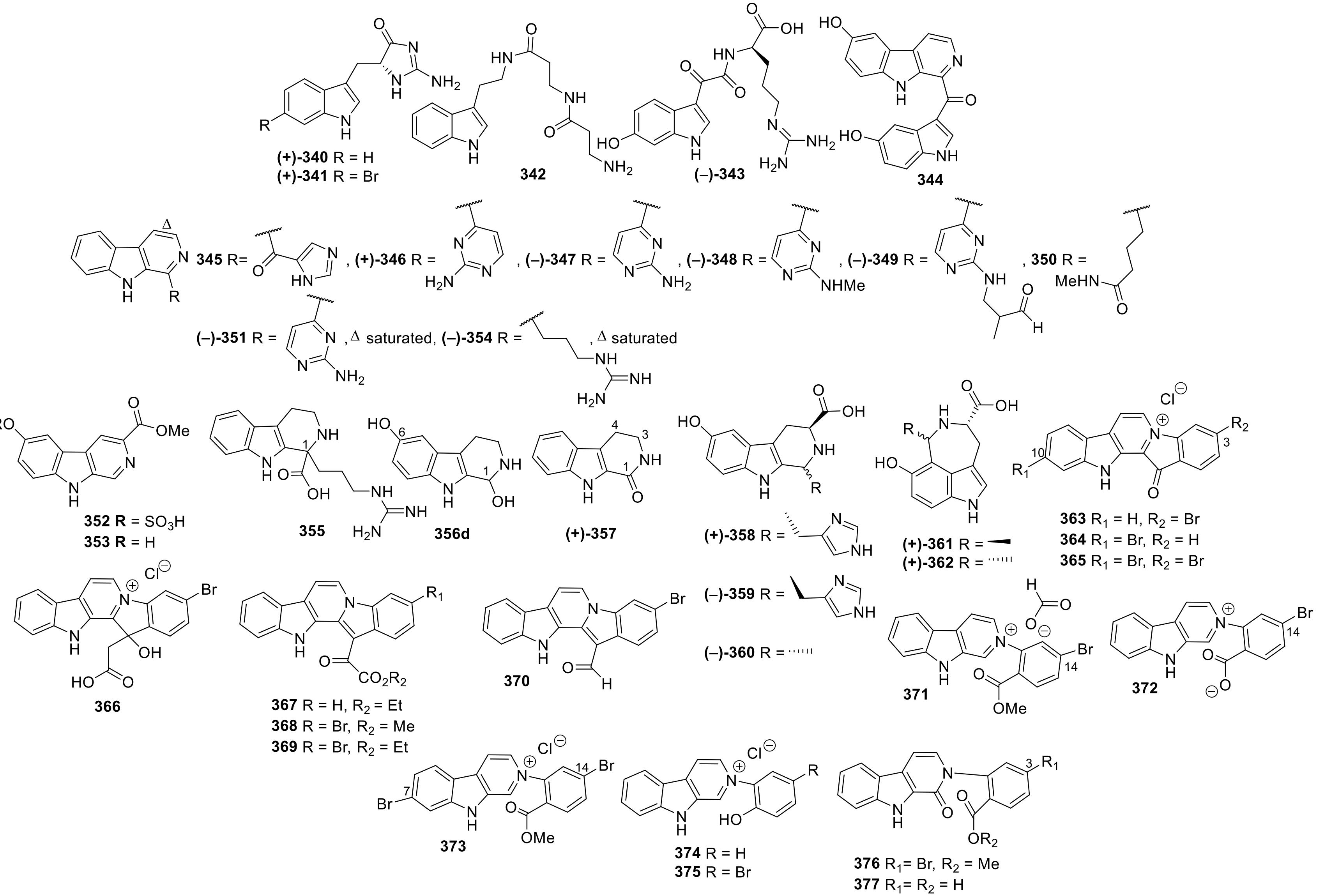

Figure S10: Structures of marine indole alkaloids from Indonesian waters found in 1970–2017.

Table S10: Marine indole alkaloids from Indonesian waters found in 1970–2017.

Compound	Structure Elucidation	Chemistry Type	Drug Class	Biological Activity: Cell/Enzyme/Micro-organism/Insect/Others	Biological Activity: Activity	Source of Organism	Province	Ref
(+)-2-Amino-1,5-dihydro-5-(1H-indol-3-ylmethyl)-4H-imidazol-4-one **340**[β,η] [$C_{12}H_{12}N_4O$]	IR, MS, NMR, $[\alpha]_D$, TS	Indole Alkaloid	Undetm.	Undetm.	Undetm.	*H. aurora*	BLI	[277]
(+)-2-amino-5-[(6-bromo-1H-indol-3-yl)methyl]-3,5-dihydro-3-methyl-4H-imidazol-4-one **341**[β,η] [$C_{13}H_{13}BrN_4O$]	IR, MS, NMR, $[\alpha]_D$, TS	Indole Alkaloid	Undetm.	Undetm.	Undetm.	*H. aurora*	BLI	[277]
Leptoclinidamide **342**[β,θ] [$C_{16}H_{22}N_4O_2$]	UV, IR, MS, NMR	Indole Alkaloid	Cytotoxic	HCT15, Jurkat	NA (30 μM)	*L. dubius*	NSW	[278]
			Antifungal	*M. hiemalis* IAM608, *S. cerevisiae* IAM 1438T	NA (250 μg/disk)			
			Antibacterial	*S. aureus* IAM 12544T, *E. coli* IAM 12119T	NA (250 μg/disk)			
(−)-Leptoclinidamine B **343**[β,θ] [$C_{16}H_{19}N_5O_5$]	UV, IR, MS, NMR, $[\alpha]_D$, CT	Indole Alkaloid	Cytotoxic	HCT15, Jurkat	NA (30 μM)	*L. dubius*	NSW	[278]
			Antifungal	*M. hiemalis* IAM608, *S. cerevisiae* IAM 1438T	NA (250 μg/disk)			
			Antibacterial	*S. aureus* IAM 12544T	NA (250 μg/disk)			
				E. coli IAM 12119T	NA (250 μg/disk)			
Hyrtiosulawesine **344**[β,η] [$C_{20}H_{12}N_3O_3$]	UV, MS, NMR	Indole Alkaloid	Anti-inflammatory	Phospholipase A_2 (*A. mellifera*)	IC_{50} > 1166 μM	*Hyrtios* sp.	SSW	[279–281]
			Antiparasite	*P. falciparum* (FcB1)	IC_{50} = 1.3 ± 0.2 μM			
				P. falciparum (W2 clone)	NA			
				L. donovani	IC_{50} = 35 μg/mL IC_{90} > 50 μg/mL			
			Cytotoxic	COLO-205	79.2% (10 μM)			
				V79	NA (4.76 μg/mL)			
Des-*N*-Methylxesto-manzamine A **345**[β,η] [$C_{15}H_{10}N_4O$]	UV, MS, NMR	Indole Alkaloid	Antiparasite	*P. falciparum* (D6 clone), *P. falciparum* (W2 clone)	NA	A sponge (*Petrosiidae*)	NSW	[252]
				L. donovani	IC_{50} = 35 μg/mL IC_{90} > 50 μg/mL			
			Cytotoxic	V79	NA (4.76 μg/mL)			

Table S10: *Cont.*

Compound	Structure Elucidation	Chemistry Type	Drug Class	Biological Activity: Cell/Enzyme/Micro-organism/Insect/Others	Biological Activity: Activity	Source of Organism	Province	Ref
(+)-Ingenine A **346**[β,η] [$C_{15}H_{11}N_5$]	UV, IR, MS, NMR, [α]D	Indole Alkaloid	Cytotoxic	L5178Y	ED_{50} < 10 μg/mL	*A. ingens*	SSW	[282]
(–)-Ingenine B **347**[β,η] [$C_{15}H_{11}N_5$]	UV, IR, MS, NMR, [α]D	Indole Alkaloid	Cytotoxic	L5178Y	ED_{50} = 9.1 μg/mL	*A. ingens*	SSW	[282]
(–)-Ingenine C **348**[β,η] [$C_{16}H_{13}N_5$]	UV, IR, MS, NMR, [α]D	Indole Alkaloid	Cytotoxic	MCF7<br>A549<br>HCT116	IC_{50} = 4.33 μM<br>W<br>IC_{50} = 6.05 μM	*A. ingens*	UEP	[283]
(–)-Ingenine D **349**[β,η] [$C_{19}H_{17}N_5O$]	UV, IR, MS, NMR, [α]D	Indole Alkaloid	Cytotoxic	MCF7, HCT116<br>A549	IC_{50} = 2.90 – 3.35 μM<br>W	*A. ingens*	UEP	[283]
Ingenine E **350**[β,η] [$C_{16}H_{18}N_3O$]	UV, IR, MS, NMR	Indole Alkaloid	Cytotoxic	A549, MCF7<br>HCT116	IC_{90} = 2.15 – 3.50 μg/mL<br>IC_{90} = 0.67 μg/mL	*A. ingens*	UEP	[284]
(–)-Acanthomine A **351**[β,η] [$C_{15}H_{13}N_5$]	UV, IR, MS, NMR, [α]D	Indole Alkaloid	Cytotoxic	L5178Y<br>HeLa, PC12<br>*A. salina*<br>A549, MCF7<br>HCT116	IC_{50} = 4.7 μg/mL<br>NA<br>25% (10 μg/mL, 24 h)<br>50% (10 μg/mL, 48 h)<br>IC_{50} = 1.92 – 2.81 μM<br>IC_{50} = 0.59 μM	*A. ingens*	NSW	[285]
Variabine A **352**[β,η] [$C_{13}H_{10}O_6N_2S$]	UV, MS, NMR	Indole Alkaloid	Anticancer	Chymotripsin-like activity<br>Ubc13 (E2)–Uev1A	IC_{50} = 16 μM<br>IC_{50} = 20 μM	*L. variabilis*	NSW	[286]
Variabine B **353**[β,η] [$C_{13}H_{10}O_6N_2S$]	UV, MS, NMR	Indole Alkaloid	Anticancer	Chymotripsin-like activity, Ubc13 (E2)–Uev1A, E1, P53-Hdm2 (E3)	NA (16 – 20 μM)	*L. variabilis*	NSW	[286]
Trypargimine **354**[β,η] [$C_{15}H_{19}N_5$]	UV, MS, NMR	Indole Alkaloid	Undetm.	Undetm.	Undetm.	*Eudistoma* sp.	SSW	[287]
1-Carboxytrypargine **355**[β,η] [$C_{16}H_{21}N_5O_2$]	UV, MS, NMR, ECD	Indole Alkaloid	Undetm.	Undetm.	Undetm.	*Eudistoma* sp.	SSW	[287]
1,6-Dihydroxy-1,2,3,4-tetrahydro- β-Carboline **356d** [$C_{11}H_{12}N_2O_2$]	Undetm.	Indole Alkaloid	Undetm.	Undetm.	Undetm.	*Hyrtios* sp.	SSW	[279]
(+)-1,2,3,4-Tetrahydronorharman-1-one **357**[β,θ] [$C_{11}H_{10}N_2O$]	UV, IR, MS, NMR, [α]D	Indole Alkaloid	Antiparasite	*P. falciparum* (D6 clone), (W2 clone), *L. donovani*	NA	A sponge (*Petrosiidae*)	NSW	[252, 284]

Table S10: *Cont.*

Compound	Structure Elucidation	Chemistry Type	Drug Class	Biological Activity: Cell/Enzyme/Micro-organism/Insect/Others	Biological Activity: Activity	Source of Organism	Province	Ref
(+)-1,2,3,4-Tetrahydronorharman-1-one **357**[β,θ] [$C_{11}H_{10}N_2O$]	UV, IR, MS, NMR, $[\alpha]_D$	Indole Alkaloid	Cytotoxic	V79<br>L5178Y<br>HeLa, PC12<br>*A. salina*<br>A549, MCF7, HCT116<br>A549, MGC-803, Bel-7404, NCI-H460, HepG2	NA (4.76 µg /mL)<br>IC_{50} > 10 µg/mL<br>NA<br>20% (10 µg/mL, 24 h)<br>40% (10 µg/mL, 48 h)<br>IC_{50} = 7.45 – 10.11 µg/mL<br>IC_{50} = 12.54 ± 1.17 – 33.46 ± 0.71 µM	A sponge (*Petrosiidae*)	NSW	[252, 284]
(+)-Hyrtioreticulin A **358**[β,θ] [$C_{16}H_{16}N_4O_3$]	UV, IR, MS, NMR, $[\alpha]_D$	Indole Alkaloid	Anticancer	E1	IC_{50} = 2.4 µM	*H. reticulatus*	NSW	[288]
(–)-Hyrtioreticulin B **359**[β,θ] [$C_{16}H_{16}N_4O_3$]	UV, IR, MS, NMR, $[\alpha]_D$	Indole Alkaloid	Anticancer	E1	IC_{50} = 35 µM	*H. reticulatus*	NSW	[288]
(–)-Hyrtioreticulin E **360**[β,θ] [$C_{13}H_{14}N_2O_3$]	UV, IR, MS, NMR, $[\alpha]_D$, ECD, CT	Indole Alkaloid	Anticancer	E1	NA (100 µM)	*H. reticulatus*	NSW	[288]
(+)-Hyrtioreticulin C **361**[β,θ] [$C_{13}H_{14}N_2O_3$]	UV, IR, MS, NMR, $[\alpha]_D$	Indole Alkaloid	Anticancer	E1	NA (100 µM)	*H. reticulatus*	NSW	[288]
(–)-Hyrtioreticulin D **362**[β,θ] [$C_{13}H_{14}N_2O_3$]	UV, IR, MS, NMR, $[\alpha]_D$	Indole Alkaloid	Anticancer	E1	NA (100 µM)	*H. reticulatus*	NSW	[288]
3-Bromofascaplysin **363**[β,ι] [$C_{18}H_{10}BrN_2O$]	MS, NMR	Indole Alkaloid	Cytotoxic	C38–L1210<br>C38–CFU-GM<br>HT116/H125–CEM<br>HOP-62, COLO-205, U251, SK-MEL-5<br>NCI-H23, NCI-H322M, NCI-H522, HCC-2998, HCT116, SF295, M14, OVCAR-4, SNB-19, MALME-3M, UACC-62, IGROV1, OVCAR-8, UO-31, HS 578T, BT-549<br>RXF-393, CAKI-1, SN12C, OVCAR-3<br>HL-60	-150 z.u. (6.4 µg/disk)<br>0 z.u. (6.4 µg/disk)<br>200/150 z.u. (6.4 µg/disk)<br>NA<br>IC_{50} = 0.49 − 0.91 µM<br>IC_{50} = 1.6 – 4.4 µM<br>35.8% apop. (0.25 µM), IC_{50} = 549 nM	*F. reticulata*	UEP	[289 – 292]

Table S10: *Cont.*

Compound	Structure Elucidation	Chemistry Type	Drug Class	Biological Activity: Cell/Enzyme/Micro-organism/Insect/Others	Biological Activity: Activity	Source of Organism	Province	Ref
3-Bromofascaplysin **363**[β,ι] [$C_{18}H_{10}BrN_2O$]	MS, NMR	Indole Alkaloid	Cytotoxic	THP-1, MDA-MB-231, SK-MEL-28<br>HeLa, DLD-1, SNU-C4, J86 P+C141	IC_{50} = 521 − 785 nM<br>IC_{50} = 238 − 337 nM	*F. reticulata*	UEP	[289 – 292]
10-Bromofascaplysin **364**[β,ι] [$C_{18}H_{10}BrN_2O$]	MS, NMR	Indole Alkaloid	Cytotoxic	C38–L1210<br>C38–CFU-GM<br>HT116/H125–CEM<br>HT116/H125–CEM<br>HL-60<br>J86 P+C141, THP-1<br>HeLa, MDA-MB-231, DLD-1, SNU-C4<br>SK-MEL-28	-100 z.u. (3.4 μg/disk)<br>100 z.u. (3.4 μg/disk)<br>200/350 z.u. (3.4 μg/disk)<br>200/300 z.u. (0.8 μg/disk)<br>36.1% apop. (0.25 μM), IC_{50} = 142 nM<br>IC_{50} = 144 − 161 nM<br>IC_{50} = 86 − 173 nM<br>IC_{50} > 1000 nM	*F. reticulata*	CSW	[289 – 292]
3-Bromohomofascaplysins B-1 **365**[β,ι] [$C_{18}H_9Br_2N_2O$]	MS, NMR	Indole Alkaloid	Undetm.	Undetm.	Undetm.	*F. reticulata, Didemnum* sp.	CSW	[289]
Homofascaplysate A **366**[β,κ] [$C_{20}H_{14}BrN_2O_3$]	MS, NMR	Indole Alkaloid	Undetm.	Undetm.	Undetm.	*F. reticulata*	CSW	[292]
Homofascaplysin B-1 **367**[β,κ] [$C_{22}H_{16}N_2O_3$]	MS, NMR	Indole Alkaloid	Undetm.	Undetm.	Undetm.	*F. reticulata*	CSW	[292]
3-Bromohomofascaplysin B **368**[β,κ] [$C_{21}H_{13}BrN_2O_3$]	MS, NMR	Indole Alkaloid	Undetm.	Undetm.	Undetm.	*F. reticulata*	CSW	[292]
3-Bromohomofascaplysin B-1 **369**[β,κ] [$C_{22}H_{13}BrN_2O_3$]	MS, NMR	Indole Alkaloid	Undetm.	Undetm.	Undetm.	*F. reticulata, Didemnum* sp.	CSW	[292]
3-Bromohomofascaplysin C **370**[β,κ] [$C_{19}H_{11}BrN_2O$]	MS, NMR	Indole Alkaloid	Undetm.	Undetm.	Undetm.	*F. reticulata, Didemnum* sp.	CSW	[292]
14-Bromoreticulatine **371**[β,κ] [$C_{19}H_{14}BrN_2O_2$]	MS, NMR	Indole Alkaloid	Cytotoxic	C38–L1210<br>C38–CFU-GM<br>HT116/H125–CEM	50 z.u. (200 μg/disk)<br>150 z.u. (200 μg/disk)<br>-/- z.u. (200 μg/disk)	*F. reticulata*	UEP	[289, 292]

Table S10: *Cont.*

Compound	Structure Elucidation	Chemistry Type	Drug Class	Biological Activity: Cell/Enzyme/Micro-organism/Insect/Others	Biological Activity: Activity	Source of Organism	Province	Ref
14-Bromoreticulatate **372**[β,κ] [$C_{18}H_{11}BrN_2O_2$]	MS, NMR	Indole Alkaloid	Cytotoxic	C38–L1210	150 z.u. (60 µg/disk)	*F. reticulata*	UEP	[289]
7,14-Dibromoreticulatine **373**[β,κ] [$C_{19}H_{13}Br_2N_2O_2$]	MS, NMR	Indole Alkaloid	Cytotoxic	C38–L1210, C38–CFU-GM<br>HT116/H125–CEM	-100 z.u. (84 µg/disk)<br>0/200 z.u. (84 µg/disk)	*F. reticulata*/ *Didemnum* sp.	CSW	[292]
Reticulatol **374**[β,κ] [$C_{17}H_{13}N_2O$]	MS, NMR	Indole Alkaloid	Cytotoxic	C38–L1210<br>C38–CFU-GM	0 z.u. (64 µg/disk)<br>-900/-600 z.u. (64 µg/disk)	*F. reticulata*	CSW	[292]
14-Bromoreticulatol **375**[β,κ] [$C_{17}H_{12}BrN_2O$]	MS, NMR	Indole Alkaloid	Cytotoxic	C38–L1210, C38–CFU-GM<br>HT116/H125–CEM	-50 z.u. (84 µg/disk)<br>-250/-/200 z.u. (84 µg/disk)	*F. reticulata*	CSW	[292]
3-Bromosecofascaplysin A **376**[β,κ] [$C_{19}H_{13}BrN_2O_3$]	MS, NMR	Indole Alkaloid	Undetm.	Undetm.	Undetm.	*F. reticulata*	CSW	[292]
3-Bromosecofascaplysin B **377**[β,κ] [$C_{18}H_{12}N_2O_3$]	MS, NMR	Indole Alkaloid	Undetm.	Undetm.	Undetm.	*F. reticulata*	CSW	[292]

Footnote: **1. Structure** ([κ]molecule isolated as salt).**Activity** (**C38** murine colon adenocarcinoma, **CFU-GM** murine colony-forming unit-granulocyte macrophage, **J86 P+C141** murine skin epidermal, **Bel-7404** human hepatocarcinoma, **BT-549** human breast cancer, **CAKI-1** human renal cancer, **COLO-205** human colon carcinoma, **CEM** human leukemia, **H125** human lung cancer, **HCC-2998** human colon carcinoma, **HCT15** human colon carcinoma, **HCT116** human colon carcinoma, **HOP-62** human non-small cell lung cancer, **HT116** human colon cancer, **HS 578T** human breast cancer, **IGROV1** human ovarian cancer, **M14** human melanoma cancer, **MALME-3M** human melanoma cancer, **MGC-803** human gastric cancer, **NCI-H23** human non-small cell lung cancer, **NCI-H322M** human non-small cell lung cancer, **NCI-H522** human non-small cell lung cancer, **OVCAR-4** human ovarian cancer, **OVCAR-8** human ovarian cancer, **RXF-393** human renal cancer, **SF-295** human CNS cancer, **SK-MEL-5** human melanoma cancer, **SK-MEL-28** human melanoma cancer, **SNB-19** human CNS cancer, **SN12C** human renal cancer, **SNU-C4** human adenocarcinoma, **THP-1** human leukemia monocyte, **U251** human CNS cancer, **UACC-62** human melanoma cancer, **UO-31** human renal cancer, **E1** ubiquitin-inhibiting enzyme, **FcB1** chloroquine-resistant strain, **z.u.** zone unit, **apop.** apoptosis).

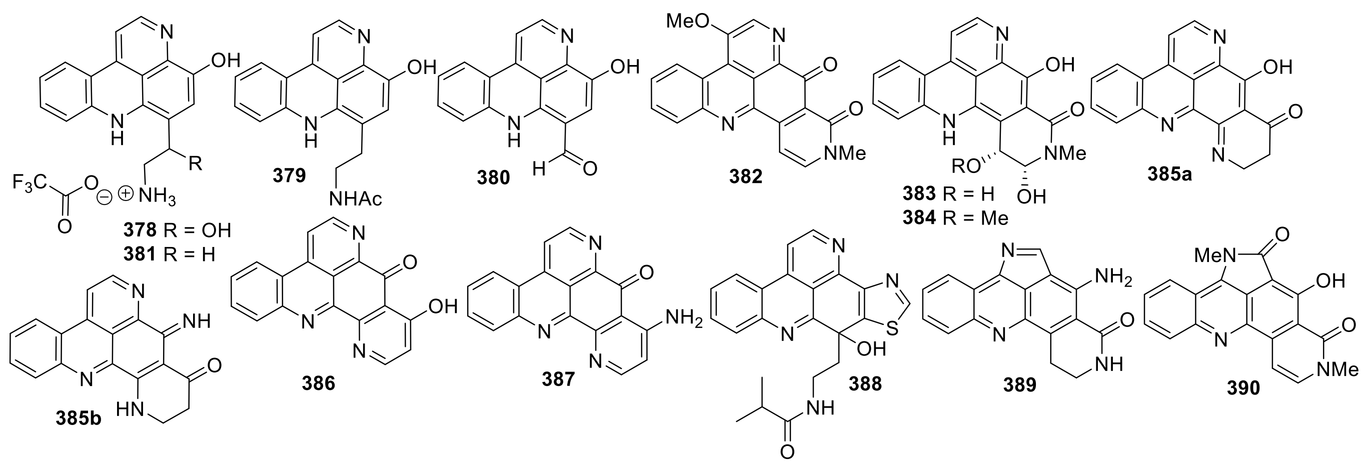

Figure S11: Structures of marine acridine alkaloids from Indonesian waters found in 1970–2017.

Table S11: Marine acridine alkaloids from Indonesian waters found in 1970–2017.

Compound	Structure Elucidation	Chemistry Type	Drug Class	Biological Activity: Cell/Enzyme/Micro-organism/Insect/Others	Biological Activity: Activity	Source of Organism	Province	Ref
Styelsamine A **378**[β,κ] [$C_{17}H_{16}N_3O_2$]	UV, MS, NMR	Acridine Alkaloid	Cytotoxic	HCT116	IC_{50} = 33 μM	*E. latericius*	SSW	[75 – 78, 293]
Styelsamine B **379**[β,κ] [$C_{19}H_{17}N_3O_2$]	UV, MS, NMR	Acridine Alkaloid	Cytotoxic	HCT116<br>Calf thymus DNA binding aff.<br>Human tumor cells (57 cells)[ν]	IC_{50} = 89 μM<br>K_{app} = 5.33 x 10^6 M^{-1}<br>GI_{50} = 3.2 μM	*E. latericius*	SSW	[75 – 78, 293]
Styelsamine C **380**[β,η] [$C_{16}H_{10}N_2O_2$]	UV, MS, NMR	Acridine Alkaloid	Cytotoxic<br>Antibacterial	HCT116<br>*L. anguilarum*	IC_{50} = 2.6 μM<br>MIC (micromolar level)	*E. latericius*	SSW	[75 – 78, 293]
Styelsamine D **381**[β,κ] [$C_{17}H_{16}N_3O$]	UV, MS, NMR	Acridine Alkaloid	Cytotoxic	HCT116	IC_{50} = 1.6 μM	*E. latericius*	SSW	[75 – 78, 293]
5-Methoxyneoamphimedine **382**[β,κ] [$C_{20}H_{13}N_3O_3$]	MS, NMR	Acridine Alkaloid	Cytotoxic	C38–L1210<br>C38–CFU-GM<br>HT116/H125–CEM	> 700 z.u. (25 μg/disk)<br>> 800 z.u. (25 μg/disk)<br>-250/-150 z.u. (25 μg/disk)	*X.* cf. *carbonaria*	UEP	[294]
Neoamphimedine Y **383**[β,κ] [$C_{19}H_{15}N_3O_4$]	NMR	Acridine Alkaloid	Cytotoxic	Undetm.	Undetm.	*X.* cf. *carbonaria*	UEP	[294]
Neoamphimedine Z **384**[β,κ] [$C_{21}H_{19}N_3O_4$]	NMR	Acridine Alkaloid	Cytotoxic	Undetm.	Undetm.	*X.* cf. *carbonaria*	UEP	[294]
Labuanine **385a**[β,η] [$C_{18}H_{11}N_3O_2$]	IR, MS, NMR, CT	Acridine Alkaloid	Anticancer	Neuro 2A	50% neuritogenesis (1 μM)	*B. fortis*	ENT	[295 – 297]
Ecionine A **385b**[δ,κ] [$C_{18}H_{12}N_4O$]	UV, LCMS, MS, NMR	Acridine Alkaloid	Cytotoxic<br>Anticancer	K562<br>TSU-Pr1-B1, TSU-Pr1,<br>TSU-Pr1-B2, 5637<br>K562	5 μg/mL<br>IC_{50} = 6.48 – 6.49 μM<br>IC_{50} = 3.55 – 3.66 μM<br>42% (25 ng/mL)	*Biemna* sp.	JPN	[296 – 297]
**386**[β,η] [$C_{18}H_9N_3O_2$]	UV, MS, NMR	Acridine Alkaloid	Anticancer<br>Cytotoxic	Neuro 2A<br>P-388<br>A549, HT-29<br>MEL-28	50% neuritogenesis (3 μM)<br>IC_{50} = 4.18 μM<br>IC_{50} = 0.03 – 0.40 μM<br>IC_{50} = 0.17 μM	*B. fortis*	ENT	[295 – 297]

Table S11: *Cont.*

Compound	Structure Elucidation	Chemistry Type	Drug Class	Biological Activity		Source of Organism	Province	Ref
				Cell/Enzyme/Micro-organism/Insect/Others	Activity			
**387**$^{\beta,\eta}$ [$C_{18}H_{10}N_4O$]	UV, MS, NMR	Acridine Alkaloid	Anticancer	Neuro 2A	> 50% neuritogenesis (0.03 μM) ACHe and G2/M (0.03 μM, 48 h)	*B. fortis*	ENT	[295 – 297]
Sagitol C **388**$^{\beta,\eta}$ [$C_{22}H_{20}N_4O_2S$]	UV, MS, NMR	Acridine Alkaloid	Cytotoxic	L5178Y, HeLa<br>PC12	ED_{50} = 0.7 – 0.9 μM<br>ED_{50} = 2.3 μM	*Oceanapia sp.*	MLU	[298]
Plakinidine D **389**$^{\beta,\eta}$ [$C_{17}H_{12}N_4O$]	UV, MS, NMR, MS	Acridine Alkaloid	Cytotoxic	HCT116	5 μg/mL	*Didemnum* sp.	SSW	[299]
Alpkinidine **390**$^{\beta,\lambda}$ [$C_{19}H_{13}N_3O_3$]	NMR, X-ray	Acridine Alkaloid$^{♣}$	Cytotoxic	C38–L1210, C38–CFU-GM<br>C38–CFU-GM	300 z.u. (120 μg/disk)<br>300 z.u. (120 μg/disk)	*X* cf. *carbonaria*	UEP	[294]

Footnote: **1. Structure** ($^{\lambda}$molecule isolated as HCO_2 salt); 2. Activity ($^{\nu}$activity tested as freebase, **5637** human bladder carcinoma).

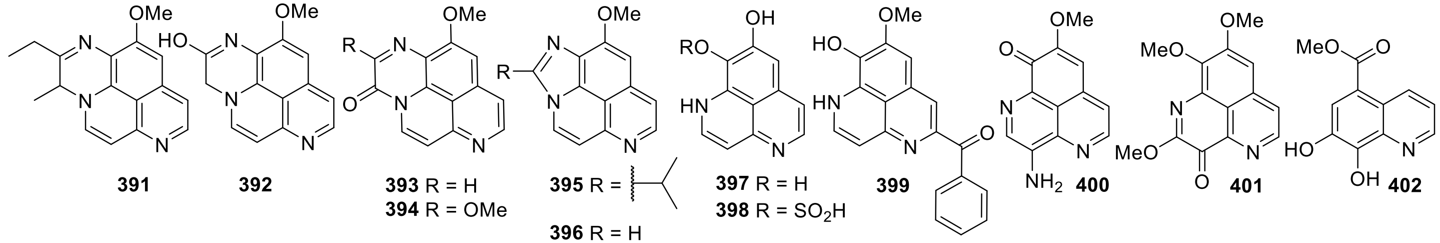

Figure S12: Structures of marine quinoline and isoquinoline alkaloids from Indonesian waters found in 1970–2017.

Table S12: Marine quinoline and isoquinoline alkaloids from Indonesian waters found in 1970–2017.

Compound	Structure Elucidation	Chemistry Type	Drug Class	Biological Activity		Source of Organism	Province	Ref
				Cell/Enzyme/Micro-organism/Insect/Others	Activity			
**391**[β,η]<br>$[C_{17}H_{17}N_3O]$	UV, IR, MS, NMR	Qunoline & Isoquinoline[♠]	Antibacterial<br>Antifungal<br>Cytotoxic	*S. aureus*, *E. coli*, *V. anguilarum*<br>*C. tropicalis*<br>KB	MIC > 100 μg/mL<br>MIC > 100 μg/mL<br>ID_{50} > 10 μg/mL	*Xestospongia* sp.	JSCR	[300]
**392**[β,η]<br>$[C_{14}H_{11}N_3O_2]$	UV, IR, MS, NMR	Qunoline & Isoquinoline	Antibacterial<br>Antifungal<br>Cytotoxic	*S. aureus*, *E. coli*, *V. anguilarum*<br>*C. tropicalis*<br>KB	MIC > 100 μg/mL<br>MIC > 100 μg/mL<br>ID_{50} > 10 μg/mL	*Xestospongia* sp.	JSCR	[300]
11-Methoxy-3*H*-[1,6]naphthyridino[6,5,4-*def*]quinoxalin-3-one **393**[β,η]<br>$[C_{14}H_9N_3O_2]$	UV, MS, NMR	Qunoline & Isoquinoline	Cytotoxic	L5178Y	NA (10 μg/mL)	*A. suberitoides*	MLU	[301]
2,11-Dimethoxy-3*H*-[1,6]naphthyridino[6,5,4-*def*]-quinoxalin-3-one **394**[β,η]<br>$[C_{15}H_{11}N_3O_3]$	UV, MS, NMR	Qunoline & Isoquinoline	Cytotoxic	L5178Y	NA (10 μg/mL)	*A. suberitoides*	MLU	[301]
**395**[β,η]<br>$[C_{16}H_{15}N_3O]$	IR, MS, NMR	Qunoline & Isoquinoline[♠]	Antibacterial<br>Antifungal<br>Cytotoxic	*S. aureus*, *E. coli*, *V. anguilarum*<br>*C. tropicalis*<br>KB	MIC > 100 μg/mL<br>MIC > 100 μg/mL<br>ID_{50} > 10 μg/mL	*Xestospongia* sp.	JSCR	[300]
**396**[β,η]<br>$[C_{13}H_9N_3O]$	UV, IR, MS, NMR	Qunoline & Isoquinoline	Antibacterial<br>Antifungal<br>Cytotoxic	*S. aureus*, *E. coli*<br>*M. smegmatis* (aerobic)<br>*M. smegmatis* (hypoxic)<br>*C. tropicalis*<br>KB<br>L5178Y	MIC > 100 μg/mL<br>MIC = 25 μg/mL<br>MIC = 12.5 μg/mL<br>MIC > 100 μg/mL<br>ID_{50} > 10 μg/mL<br>IC_{50} = 13.5 μM	*Xestospongia* sp.	JSCR	[300 – 302]
Bisdemethylaaptamine **397**[ζ,θ]<br>$[C_{11}H_8N_2O_2]$	UV, IR, NMR, MS	Qunoline & Isoquinoline	Antifungal<br>Antibacterial	*C. neoformans*, *C. albicans*<br>*S. aureus*, *S. pneumoniae*, *M. luteus*<br>*E. faecalis*	MIC = 32 – 64 μg/mL<br>MIC = 4 – 8 μg/mL<br>MIC = 8 – 16 μg/mL	*Aaptos* sp.	NSW	[303, 304]

Table S12: *Cont.*

Compound	Structure Elucidation	Chemistry Type	Drug Class	Biological Activity: Cell/Enzyme/Micro-organism/Insect/Others	Biological Activity: Activity	Source of Organism	Province	Ref
Bisdemethylaaptamine **397**[ζ,θ] [$C_{11}H_8N_2O_2$]	UV, IR, NMR, MS	Qunoline & Isoquinoline	Antibacterial	*E. coli*<br>*E. cloacae*<br>*S. maltophilia*<br>*N. gonorrhoeae*	MIC = 16 − 64 µg/mL<br>NA<br>MIC = 32 µg/mL<br>MIC < 0.5 µg/mL	*Aaptos* sp.	NSW	[302, 304]
			Antiparasite	*P. falciparum* (D6 clone), (W2 clone)	NA			
			Cytotoxic	P-388, BXPC-3, MCF7, SF268, NCI-H460, KM-20L2<br>DU-145	EC_{50} = 0.12 – 0.80 µg/mL<br>EC_{50} = 1.10 µg/mL			
Bisdemethylaaptamine-9-*O*-sulfate **398**[β,θ] [$C_{11}H_8N_2O_4S$]	UV, IR, NMR, MS	Qunoline & Isoquinoline	Undetm.	Undetm.	Undetm.	*Aaptos* sp.	NSW	[303, 304]
5-Benzoyldemethyl aaptamine **399**[β,η] [$C_{19}H_{14}N_2O_3$]	UV, NMR, MS	Qunoline & Isoquinoline	Cytotoxic	L5178Y	IC_{50} = 5.5 µM	*A. suberitoides*	MLU	[301]
			Antibacterial	*M. smegmatis* (aerobic)<br>*M. smegmatis* (hypoxic)	MIC = 6.25 µg/mL<br>MIC = 1.5 µg/mL			
3-Aminodemethyl (oxy)aaptamine **400**[β,η] [$C_{12}H_9N_3O_2$]	UV, NMR, MS	Qunoline & Isoquinoline	Cytotoxic	L5178Y	64% (10 µg/mL)	*A. suberitoides*	MLU	[301, 302]
2-Methoxy-3-oxoaaptamine **401**[β,η] [$C_{14}H_{12}N_2O_4$]	UV, NMR, MS	Qunoline & Isoquinoline	Antibacterial	*M. smegmatis* (aerobic), *M. smegmatis* (hypoxic)	MIC = 6.25 µg/mL	*Aaptos* sp.	ENT	[302]
Aaptoline **402**[β,θ] [$C_{11}H_9NO_4$]	UV, IR, MS, NMR	Qunoline & Isoquinoline	Undetm.	Undetm.	Undetm.	*A. suberitoides*	NSW	[305]

Footnote: **1. Statistic** (**ID_{50}** 50% of the infective dose); **2. Activity** (**BXPC-3** human pancreas adenocarcinoma, **DU-145** human prostate cancer, **KM-20L2** human colon carcinoma).

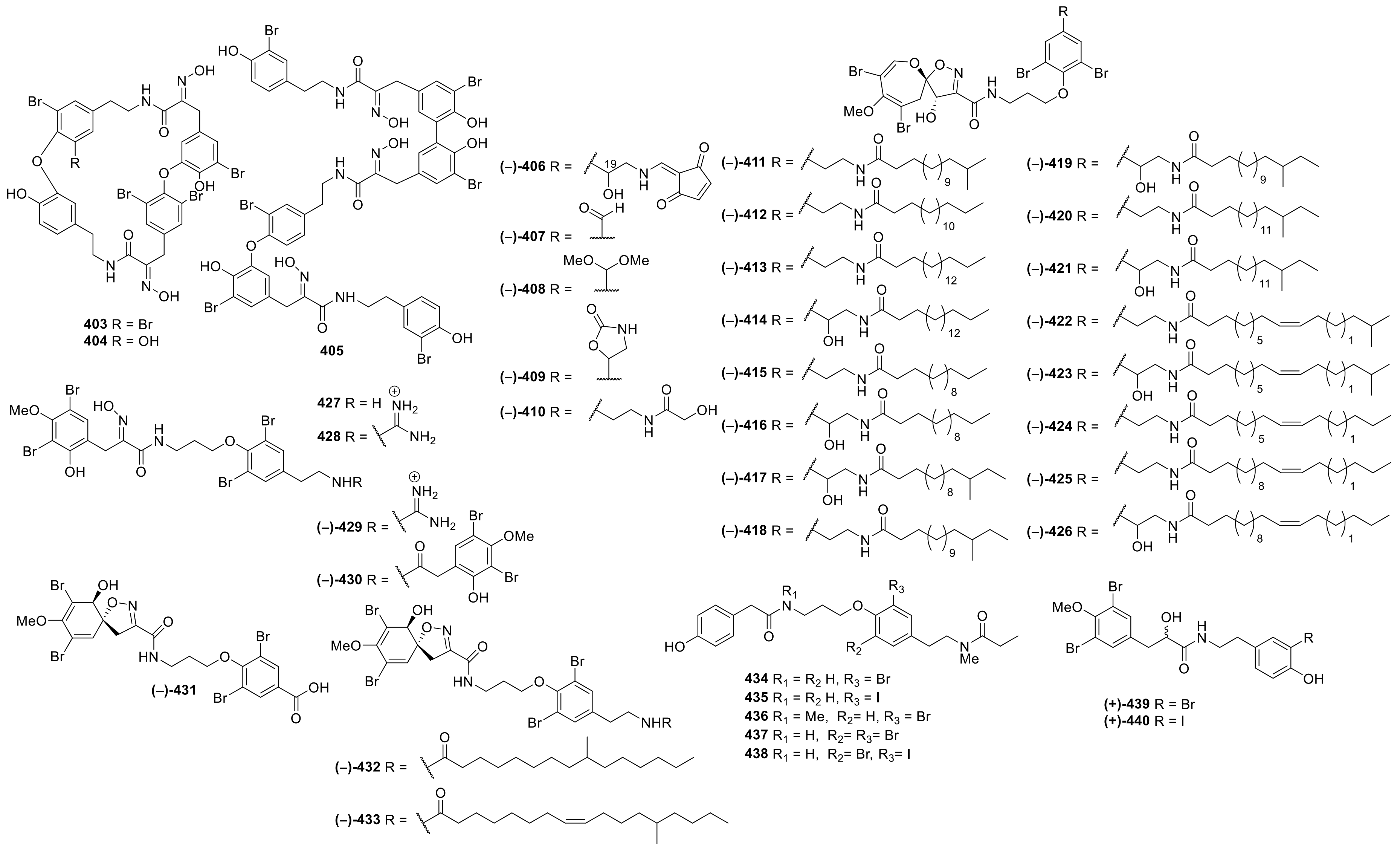

Figure S13: Structures of marine tyrosine alkaloids from Indonesian waters found in 1970–2017.

Table S13: Marine tyrosine alkaloids from Indonesian waters found in 1970–2017.

Compound	Structure Elucidation	Chemistry Type	Drug Class	Biological Activity		Source of Organism	Province	Ref
				Cell/Enzyme/Micro-organism/Insect/Others	Activity			
Bastadin 16 **403**[β,η] [$C_{34}H_{27}Br_5N_4O_8$]	UV, IR, MS, NMR	Tyrosine Alkaloid	Cytotoxic	Human Sup-T_1 cancer cell	$IC_{50} = 1 \times 10^{-12}$	*I. basta*	NSW	[306 – 310]
				MCF7, SK-MEL-28, A549, HS683, B16F10, U373	IC_{50} = 4 – 11 μM			
				L5178Y	IC_{50} = 1.9 μM			
			Anticancer	Aur-A, Aur-B, EGF-R,	IC_{50} = 1.3 – 2.9 μM			
				CDK4/CycD1, ERBB2	IC_{50} = 4.0 – 4.5 μM			
			Antifouling	*B. improvisus*	10 μM			
			Toxicity	*B. improvisus*	10 μM			
			Alzheimer	Apoliprotein E modulatory activity	NA (40 μM)			
Bastadin 17 **404**[β,η] [$C_{34}H_{28}Br_4N_4O_9$]	UV, IR, MS, NMR	Tyrosine Alkaloid	Undetm.	Undetm.	Undetm.	*I. basta*	NSW	[306]
Sesquibastadin 1 **405**[β,η] [$C_{51}H_{44}Br_6O_{12}$]	UV, IR, MS, NMR	Tyrosine Alkaloid[♣]	Cytotoxic	L5178Y	NA	*I. basta*	MLU	[311]
			Anticancer	ARK5, B-RAF VE, Aur-A, Aur-B, CDK4/CycD1, FLT3, INS-R, COT, ERBB2, VEGF-R3	IC_{50} = 1.3 – 2.6 μM			
				CDK2/CycA, PLK1	IC_{50} = 6.4 – 6.5 μM			
				EGF-R, MET, IGF1-R, SAK, SRC, TIE2, VEGF-R2	IC_{50} = 0.6 – 1.0 μM			
				EPHB4, FAK, PDGFR-β	IC_{50} = 3.4 – 4.0 μM			
(−)-19-Hydroxy psammaplysin E **406**[α,β] [$C_{27}H_{25}Br_4N_3O_9$]	MS, NMR, $[\alpha]_D$, CT	Tyrosine Alkaloid	Antiparasite	*P. falciparum* (3D7)	IC_{50} = 6.4 ± 1.4 μM	*A. strongylata*	BLI	[312]
(−)-Psammaplysin K **407**[β] [$C_{20}H_{18}Br_4N_2O_7$]	MS, NMR, $[\alpha]_D$	Tyrosine Alkaloid	Antiparasite	*P. falciparum* (3D7)	NA (10 μM)	*A. strongylata*	BLI	[312]
(−)-Psammaplysin K dimethoxy acetal **408**[β] [$C_{24}H_{24}Br_4N_2O_8$]	MS, NMR, $[\alpha]_D$	Tyrosine Alkaloid	Undetm.	Undetm.	Undetm.	*A. strongylata*	BLI	[312]
(−)-Psammaplysin L **409**[β] [$C_{22}H_{21}Br_4N_3O_8$]	MS, NMR, $[\alpha]_D$	Tyrosine Alkaloid	Antiparasite	*P. falciparum* (3D7)	NA (10 μM)	*A. strongylata*	BLI	[312]
(−)-Psammaplysin M **410**[β] [$C_{23}H_{25}Br_4N_3O_8$]	MS, NMR, $[\alpha]_D$	Tyrosine Alkaloid	Antiparasite	*P. falciparum* (3D7)	NA (10 μM)	*A. strongylata*	BLI	[312]

Table S13: *Cont.*

Compound	Structure Elucidation	Chemistry Type	Drug Class	Biological Activity: Cell/Enzyme/Micro-organism/Insect/Others	Biological Activity: Activity	Source of Organism	Province	Ref
(−)-Psammaplysin N **411**[β] [$C_{37}H_{53}Br_4N_3O_7$]	MS, NMR, [α]D	Tyrosine Alkaloid	Antiparasite	*P. falciparum* (3D7)	NA (10 μM)	*A. strongylata*	BLI	[312]
(−)-Psammaplysin O **412**[β] [$C_{37}H_{53}Br_4N_3O_7$]	MS, NMR, [α]D	Tyrosine Alkaloid	Undetm.	Undetm.	Undetm.	*A. strongylata*	BLI	[312]
(−)-Psammaplysin P **413**[β] [$C_{39}H_{57}Br_4N_3O_7$]	MS, NMR, [α]D	Tyrosine Alkaloid	Undetm.	Undetm.	Undetm.	*A. strongylata*	BLI	[312]
(−)-19-Hydroxy psammaplysin P **414**[α,β] [$C_{39}H_{57}Br_4N_3O_8$]	MS, NMR, [α]D	Tyrosine Alkaloid	Antiparasite	*P. falciparum* (3D7)	NA (10 μM)	*A. strongylata*	BLI	[312]
(−)-Psammaplysin Q **415**[β] [$C_{35}H_{49}Br_4N_3O_7$]	MS, NMR, [α]D	Tyrosine Alkaloid	Undetm.	Undetm.	Undetm.	*A. strongylata*	BLI	[312]
(−)-19-Hydroxy psammaplysin Q **416**[α,β] [$C_{35}H_{49}Br_4N_3O_8$]	MS, NMR, [α]D	Tyrosine Alkaloid	Undetm.	Undetm.	Undetm.	*A. strongylata*	BLI	[312]
(−)-Psammaplysin R **417**[β] [$C_{37}H_{53}Br_4N_3O_8$]	MS, NMR, [α]D,	Tyrosine Alkaloid	Undetm.	Undetm.	Undetm.	*A. strongylata*	BLI	[312]
(−)-Psammaplysin S **418**[β] [$C_{38}H_{55}Br_4N_3O_7$]	MS, NMR, [α]D	Tyrosine Alkaloid	Undetm.	Undetm.	Undetm.	*A. strongylata*	BLI	[312]
(−)-19-Hydroxy psammaplysin S **419**[α,β] [$C_{38}H_{55}Br_4N_3O_8$]	MS, NMR, [α]D	Tyrosine Alkaloid	Undetm.	Undetm.	Undetm.	*A. strongylata*	BLI	[312]
(−)-Psammaplysin T **420**[β] [$C_{40}H_{59}Br_4N_3O_7$]	MS, NMR, [α]D	Tyrosine Alkaloid	Antiparasite	*P. falciparum* (3D7)	NA (10 μM)	*A. strongylata*	BLI	[312]
(−)-19-Hydroxy psammaplysin T **421**[α,β] [$C_{40}H_{59}Br_4N_3O_8$]	MS, NMR, [α]D	Tyrosine Alkaloid	Undetm.	Undetm.	Undetm.	*A. strongylata*	BLI	[312]
(−)-Psammaplysin U **422**[β] [$C_{38}H_{53}Br_4N_3O_7$]	MS, NMR, [α]D	Tyrosine Alkaloid	Undetm.	Undetm.	Undetm.	*A. strongylata*	BLI	[312]
(−)-19-Hydroxy psammaplysin U **423**[α,β] [$C_{38}H_{53}Br_4N_3O_8$]	MS, NMR, [α]D	Tyrosine Alkaloid	Undetm.	Undetm.	Undetm.	*A. strongylata*	BLI	[312]
(−)-Psammaplysin V **424**[β] [$C_{37}H_{51}Br_4N_3O_7$]	MS, NMR, [α]D	Tyrosine Alkaloid	Antiparasite	*P. falciparum* (3D7)	NA (10 μM)	*A. strongylata*	BLI	[312]

Table S13: *Cont.*

Compound	Structure Elucidation	Chemistry Type	Drug Class	Biological Activity: Cell/Enzyme/Micro-organism/Insect/Others	Biological Activity: Activity	Source of Organism	Province	Ref
(−)-Psammaplysin W **425**[β] [$C_{40}H_{57}Br_4N_3O_8$]	MS, NMR, $[\alpha]_D$	Tyrosine Alkaloid	Undetm.	Undetm.	Undetm.	*A. strongylata*	BLI	[312]
(−)-19-Hydroxy psammaplysin W **426**[α,β] [$C_{40}H_{57}Br_4N_3O_8$]	MS, NMR, $[\alpha]_D$	Tyrosine Alkaloid	Undetm.	Undetm.	Undetm.	*A. strongylata*	BLI	[312]
Purpuramine M **427**[β] [$C_{21}H_{23}Br_4N_3O_5$]	UV, IR, NMR, MS	Tyrosine Alkaloid	Alzheimer<br>Cytotoxic	BACE1<br>A2780S, A2780S CP5, U251MG	36% (42 μM)<br>IC_{50} = 20 – 50 μM	A sponge (Aplysi-nellidae)	EKM	[313]
Purpuramine N **428**[β,ι] [$C_{22}H_{26}Br_4N_5O_5$]	UV, IR, NMR, MS	Tyrosine Alkaloid	Undetm.	Undetm.	Undetm.	A sponge (Aplysi-nellidae)	EKM	[313]
(−)-Araplysillin VII **429**[β,κ] [$C_{22}H_{26}Br_4N_5O_5$]	UV, IR, NMR, MS, $[\alpha]_D$	Tyrosine Alkaloid	Alzheimer<br>Cytotoxic	BACE1<br>A2780S, A2780S CP5, U251MG, A549, MCF7	40% (39.6 μM)<br>Active > 50 μM	A sponge (Aplysi-nellidae)	EKM	[313]
(−)-Araplysillin VIII **430**[β] [$C_{30}H_{29}Br_6N_3O_8$]	UV, IR, NMR, MS, $[\alpha]_D$	Tyrosine Alkaloid	Undetm.	Undetm.	Undetm.	A sponge (Aplysi-nellidae)	EKM	[313]
(−)-Araplysillin IX **431**[β] [$C_{20}H_{18}Br_4N_2O_7$]	UV, IR, NMR, MS, $[\alpha]_D$	Tyrosine Alkaloid	Alzheimer<br>Cytotoxic	BACE1<br>A2780S, A2780S CP5, U251MG, A549, MCF7	35% (41.9 μM)<br>> 50 μM	A sponge (Aplysi-nellidae)	EKM	[313]
(−)-Araplysillin X **432**[β] [$C_{37}H_{53}Br_4N_3O_6$]	UV, IR, NMR, MS, $[\alpha]_D$	Tyrosine Alkaloid	Alzheimer<br>Cytotoxic	BACE1<br>A2780S, A2780S CP5, U251MG, A549, MCF7	70% (31.4 μM)<br>> 50 μM	A sponge (Aplysi-nellidae)	EKM	[313]
(−)-Araplysillin XI **433**[β] [$C_{39}H_{56}Br_4N_3O_6$]	UV, IR, NMR, MS, $[\alpha]_D$	Tyrosine Alkaloid	Alzheimer<br>Cytotoxic	BACE1<br>A2780S, A2780S CP5, U251MG, A549, MCF7	60% (30.6 μM)<br>> 50 μM	A sponge (Aplysi-nellidae)	EKM	[313]
Enisorine A **434**[β] [$C_{23}H_{29}BrN_2O_4$]	UV, IR, MS, NMR	Tyrosine Alkaloid	Antibacterial	*Y. pseudotubercolosis*	60 μM (> 50% type III secretion system, T3SS)	*I.* cf. *iota*	CSW	[314]
Enisorine B **435**[β] [$C_{23}H_{29}IN_2O_4$]	UV, IR, MS, NMR	Tyrosine Alkaloid	Antibacterial	*Y. pseudotubercolosis*	120 μM (> 50% type III secretion system, T3SS)	*I.* cf. *iota*	CSW	[314]
Enisorine C **436**[β] [$C_{24}H_{31}BrN_2O_4$]	UV, IR, MS, NMR	Tyrosine Alkaloid	Antibacterial	*Y. pseudotubercolosis*	30 μM (> 50% type III secretion system, T3SS)	*I.* cf. *iota*	CSW	[314]
Enisorine D **437**[β] [$C_{23}H_{28}Br_2N_2O_4$]	UV, IR, MS, NMR	Tyrosine Alkaloid	Antibacterial	*Y. pseudotubercolosis*	120 μM (> 50% type III secretion system, T3SS)	*I.* cf. *iota*	CSW	[314]

Table S13: *Cont.*

Compound	Structure Elucidation	Chemistry Type	Drug Class	Biological Activity: Cell/Enzyme/Micro-organism/Insect/Others	Biological Activity: Activity	Source of Organism	Province	Ref
Enisorine E **438**[β] [$C_{23}H_{28}BrIO_4N_2$]	UV, IR, MS, NMR	Tyrosine Alkaloid	Antibacterial	*Y. pseudotubercolosis*	30 μM (> 50% type III secretion system, T3SS)	*I.* cf. *iota*	CSW	[314]
(+)-1-O-Methyl hemi-bastadinol 2 **439**[β] [$C_{18}H_{18}Br_3NO_4$]	UV, IR, MS, NMR, $[\alpha]_D$	Tyrosine Alkaloid	Antibacterial	*Y. pseudotubercolosis*	30 μM (> 50% type III secretion system, T3SS)	*I* cf. *iota*	CSW	[314]
(+)-1-O-Methyl hemi-bastadinol 4 **440**[β] [$C_{18}H_{18}Br_2INO_4$]	UV, IR, MS, NMR, $[\alpha]_D$	Tyrosine Alkaloid	Antibacterial	*Y. pseudotubercolosis*	60 μM (> 50% type III secretion system, T3SS)	*I.* cf. *iota*	CSW	[314]

Footnote: **1. Activity** (**B16F10** murine melanoma cancer, **A2780S** human ovarian carcinoma, **CP5** human ovarian carcinoma cisplatin resistant, **HS683** human oligodendroglioma, **Sup-T1** human T cell lymphoma, **U251MG** human glioma, **U373** human astroglioma, **3D7** *Plasmodium falciparum* chloroquine-sensitive strain).

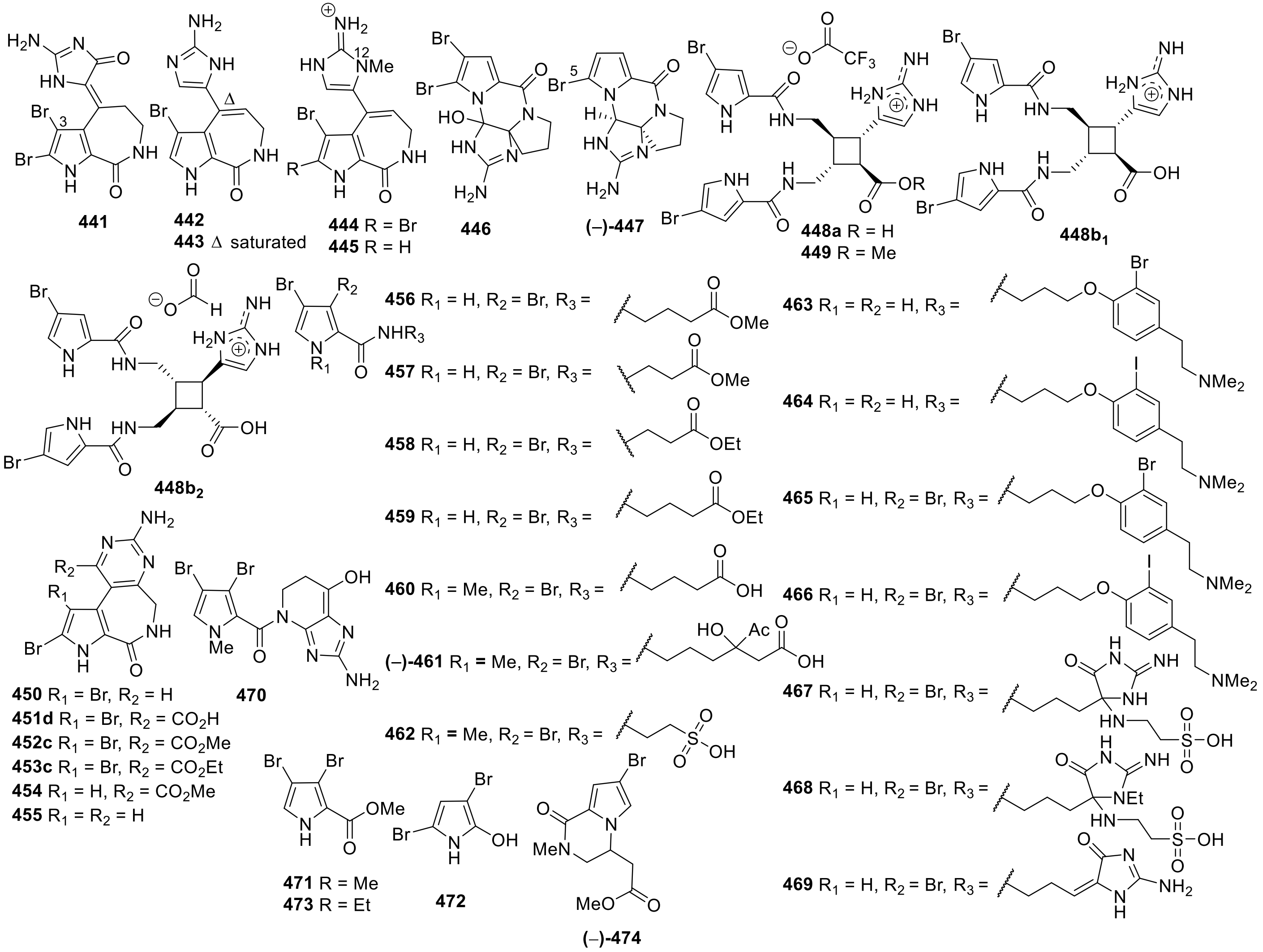

Figure S14: Structures of marine pyrrole alkaloids from Indonesian waters found in 1970–2017.

Table S14: Marine pyrrole alkaloids from Indonesian waters found in 1970–2017.

Compound	Structure Elucidation	Chemistry Type	Drug Class	Biological Activity: Cell/Enzyme/Micro-organism/Insect/Others	Biological Activity: Activity	Source of Organism	Province	Ref
(*Z*)-3-Bromohymenialdisine **441**[β] [$C_{11}H_9Br_2N_5O_2$]	UV, NMR, MS	Pyrrole Alkaloid	Antiinsecticidal	*S. littoralis*	NA (300 μg/mL)	*A. carteri*	UEP	[315, 316]
			Cytotoxic	L5178Y	ED_{50} = 3.9 μg/mL<br>60.5% (10 μg/mL)			
			Anticancer	Aurora-A, CDK4-CycD1, FAK, VEGF-R2, SAK, PDGFR-β	20% < residual kinase activity ≤ 60%			
				Aurora-B, SRC, COT, PLK1	60% < residual kinase activity ≤ 80%			
Debromostevensine **442**[β,θ] [$C_{11}H_{10}BrN_5O$]	UV, MS, NMR	Pyrrole Alkaloid	Cytotoxic	MONO-MAC 6<br>L5178Y	NA<br>7.5% (10 μg/mL)	*S. carteri*	MLU	[317]
Debromohymenin **443**[β,θ] [$C_{11}H_{12}BrN_5O$]	UV, ECD, NMR, MS	Pyrrole Alkaloid	Cytotoxic	MONO-MAC 6	NA	*S. carteri*	MLU	[317]
12-*N*-Methyl stevensine **444**[β,θ] [$C_{12}H_{12}Br_2N_5O$]	IR, MS, NMR	Pyrrole Alkaloid	Cytotoxic	L5178Y	EC_{50} = 3.5 μg/mL<br>IC_{50} = 8.75 μM	*Stylissa* sp.	EKM	[318]
			Anticancer	CLK-1, CDK5, CK-1<br>Cdk9/cyclin T, GSK-3, CDK1<br>Cdk2/A	IC_{50} = 4.1 – 6 μM<br>IC_{50} = 1.3 – 3.2 μM<br>IC_{50} > 10 μM			
12-*N*-Methyl-2-debromostevensine **445**[β,η] [$C_{12}H_{13}BrN_5O$]	IR, MS, NMR	Pyrrole Alkaloid	Cytotoxic	L5178Y	8.1% (10 μg/mL)	*Stylissa* sp.	EKM	[318]
(−)-Dibromohydroxy phakellin **446**[β,η] [$C_{11}H_{11}Br_2N_5O_2$]	UV, MS, NMR, $[\alpha]_D$	Pyrrole Alkaloid	Cytotoxic	L5178Y	112.8% (23 mM)	*A. linnaei*	JSCR	[125]
			Antibacterial	*S. epidermidis*	MIC > 45 μM			
(−)-5-Bromophakelline **447**[β,θ] [$C_{11}H_{12}BrN_5O$]	UV, MS, NMR, $[\alpha]_D$	Pyrrole Alkaloid	Antibacterial	*M. smegmatis*	NA (50 μg/disk)	*Agelas* sp.	NSW	[319]
(−)-Nakamuric acid **448a**[β,θ] [$C_{20}H_{21}Br_2N_8O_3$]	UV, MS, NMR, $[\alpha]_D$	Pyrrole Alkaloid[♠]	Antibacterial	*B. subtilis* 168<br>*S. aureus* ATCC25923, ATCC43300	9 mm (0.2 μmol/disk)<br>MIC = 16 μg/mL	*A. nakamurai*	MLU	[80]
(−)-Nakamuric acid **448b**$_1$[γ,λ] [$C_{20}H_{22}Br_2N_7O_4$]	TS	Pyrrole Alkaloid	Undetm.	Undetm.	Undetm.			[34]

Table S14: *Cont.*

Compound	Structure Elucidation	Chemistry Type	Drug Class	Biological Activity		Source of Organism	Province	Ref
				Cell/Enzyme/Micro-organism/Insect/Others	Activity			
(−)-Nakamuric acid **448b2**[γ,λ] [$C_{20}H_{22}Br_2N_7O_4$]	UV, MS, NMR, $[\alpha]_D$, ECD	Pyrrole Alkaloid	Cytotoxic	PC9	IC_{50} > 10 µg/mL	*Agelas* sp.	PRC	[34]
(−)-Nakamuric acid methyl ester **449**[β] [$C_{21}H_{24}Br_2N_7O_4$]	UV, MS, NMR, $[\alpha]_D$	Pyrrole Alkaloid	Antibacterial	*B. subtilis* 168	9 mm (0.2 µmol/disk)	*A. nakamurai*	MLU	[80]
Latonduine A **450**[β,η] [$C_{10}H_7Br_2N_5O$]	UV, MS, NMR, CT, TS	Pyrrole Alkaloid[♣]	Cytotoxic	L5178Y	EC_{50} = 9.0 µg/mL<br>IC_{50} = 26.81 µM	*S. carteri*	SSW	[45, 317, 320, 321]
			Cystic fibrosis	BHK (wild type)	45% F508del-CTFR corr. (1 µM, 24 h)			
				Salivary secretion *in vivo* assay in F508del-CFTR homo. mice	9% F508del-CTFR corr. (50 mg/kg, 2 days)			
				Ex vivo mouse assay (intestinal ileal epithelia from F508del-CFTR homo. mice)	2.5% F508del-CTFR corr. (10 µM, 4 h)			
				PARP-3	EC_{50} = 400 pM			
Latonduine B **451d**[β,η] [$C_{11}H_7Br_2N_5O_3$]	Undetm.	Pyrrole Alkaloid	Undetm.	Undetm.	Undetm.	*S. carteri*	SSW	[45]
Latonduin B methyl ester **452c**[ε,η] [$C_{12}H_9Br_2N_5O_3$]	UV, MS, NMR	Pyrrole Alkaloid	Cytotoxic	L5178Y	1.7% (10 µg/mL)	*S. carteri*	SSW	[45, 317, 320, 321]
			Cystic fibrosis	BHK (wild type)	15% F508del-CTFR corr. (1 µM, 24 h)			
Latonduin B ethyl ester **453c**[ε,η] [$C_{13}H_{11}Br_2N_5O_3$]	UV, MS, NMR	Pyrrole Alkaloid	Cytotoxic	L5178Y	1.7% (10 µg/mL)	*S. carteri*	SSW	[45, 317, 320, 321]
			Anticancer	CLK-1, CDK5, GSK-3, DYRK1A, CK-1, CDK1, Cdk9/cyclin T	IC_{50} > 10 µM			
			Cystic fibrosis	BHK (wild type)	30% F508del-CTFR corr. (1 µM, 24 h)			
3-Debromolatonduine B methyl ester **454**[β,η] [$C_{12}H_{10}BrN_5O_3$]	IR, MS, NMR	Pyrrole Alkaloid	Cytotoxic	L5178Y	10.2% (10 µg/mL)	*Stylissa* sp.	EKM	[317, 321]

Table S14: *Cont.*

Compound	Structure Elucidation	Chemistry Type	Drug Class	Biological Activity: Cell/Enzyme/Micro-organism/Insect/Others	Biological Activity: Activity	Source of Organism	Province	Ref
3-Debromo latonduine A **455**[β,η] [$C_{10}H_8BrN_5O$]	IR, MS, NMR	Pyrrole Alkaloid	Cytotoxic<br>Anticancer	L5178Y<br>CLK-1, DYRK1A<br>GSK-3, CK-1<br>CDK1, CDK2/A, CDK5, CDK9/cyclin T	6.6% (10 μg/mL)<br>IC_{50} = 1.7 − 2 μM<br>IC_{50} = 0.21 − 0.78 μM<br>IC_{50} > 10 μM	*Stylissa* sp.	EKM	[317, 321]
Acanthamide A **456**[β,λ] [$C_{10}H_{12}Br_2N_2O_3$]	UV, MS, NMR	Pyrrole Alkaloid	Undetm.	Undetm.	Undetm.	*Acantho-stylotella* sp.	BLI	[322]
Acanthamide B **457**[β,λ] [$C_9H_{10}Br_2N_2O_3$]	UV, MS, NMR	Pyrrole Alkaloid	Undetm.	Undetm.	Undetm.	*Acantho-stylotella* sp.	BLI	[322]
Acanthamide C **458**[β,λ] [$C_{10}H_{12}Br_2N_2O_3$]	UV, MS, NMR	Pyrrole Alkaloid	Undetm.	Undetm.	Undetm.	*Acantho-stylotella* sp.	BLI	[322]
Acanthamide D **459**[β,λ] [$C_{11}H_{14}Br_2N_2O_3$]	UV, MS, NMR	Pyrrole Alkaloid	Undetm.	Undetm.	Undetm.	*Acantho-stylotella* sp.	BLI	[322]
4-(4,5-dibromo-1-methyl pyrrole-2-carboxamido)-butanoic acid **460**[β,η] [$C_{10}H_{12}Br_2N_2O_3$]	UV, MS, NMR	Pyrrole Alkaloid	Cytotoxic<br>Antibacterial	L5178Y<br>*S. epidermidis*	107.5% (23 mM)<br>MIC > 54 μM	*A. linnaei*	JSCR	[125]
(−)-Agelanin B **461**[β,η] [$C_{14}H_{18}Br_2N_2O_5$]	UV, MS, NMR, $[\alpha]_D$	Pyrrole Alkaloid	Cytotoxic<br>Antibacterial	L5178Y<br>*S. epidermidis*	92.8% (23 mM)<br>MIC > 44 μM	*A. linnaei*	JSCR	[125]
Mauritamide D **462**[β,η] [$C_8H_{10}Br_2N_2O_4S$]	UV, MS, NMR	Pyrrole Alkaloid	Cytotoxic<br>Antibacterial	L5178Y<br>*S. epidermidis*	117.6% (23 mM)<br>MIC > 52 μM	*A. linnaei*	JSCR	[125]
Agelanesin A **463**[β,η] [$C_{18}H_{23}Br_2N_3O_2$]	UV, NMR, MS	Pyrrole Alkaloid	Cytotoxic<br>Antibacterial	L5178Y<br>*S. epidermidis*	IC_{50} = 9.55 μM<br>MIC > 42 μM	*A. linnaei*	JSCR	[125]
Agelanesin B **464**[β,η] [$C_{18}H_{23}BrIN_3O_2$]	UV, MS, NMR	Pyrrole Alkaloid	Cytotoxic<br>Antibacterial	L5178Y<br>*S. epidermidis*	IC_{50} = 9.25 μM<br>MIC > 38 μM	*A. linnaei*	JSCR	[125]
Agelanesin C **465**[β,η] [$C_{18}H_{22}Br_3N_3O_2$]	UV, NMR, MS	Pyrrole Alkaloid	Cytotoxic<br>Antibacterial	L5178Y<br>*S. epidermidis*	IC_{50} = 16.76 μM<br>MIC > 36 μM	*A. linnaei*	JSCR	[125]
Agelanesin D **466**[β,η] [$C_{18}H_{22}Br_2IN_3O_2$]	UV, MS, NMR	Pyrrole Alkaloid	Cytotoxic<br>Antibacterial	L5178Y<br>*S. epidermidis*	IC_{50} = 13.06 μM<br>MIC > 33 μM	*A. linnaei*	JSCR	[125]
(−)-Mauritamide B **467**[β,η] [$C_{13}H_{18}Br_2N_6O_5S$]	UV, MS, NMR, $[\alpha]_D$	Pyrrole Alkaloid	Cytotoxic<br>Antibacterial	L5178Y<br>*S. epidermidis*	92.5% (23 mM)<br>MIC > 37 μM	*A. linnaei*	JSCR	[125]
(−)-Mauritamide C **468**[β,η] [$C_{15}H_{22}Br_2N_6O_5S$]	UV, MS, NMR, $[\alpha]_D$	Pyrrole Alkaloid	Cytotoxic<br>Antibacterial	L5178Y<br>*S. epidermidis*	98.6% (23 mM)<br>MIC > 35 μM	*A. linnaei*	JSCR	[125]

Table 14. *Cont.*

Compound	Structure Elucidation	Chemistry Type	Drug Class	Biological Activity: Cell/Enzyme/Micro-organism/Insect/Others	Biological Activity: Activity	Source of Organism	Province	Ref
Dispacamide E **469**[β,η] [$C_{11}H_{11}Br_2N_5O_2$]	MS, NMR	Pyrrole Alkaloid	Cytotoxic	L5178Y	27.2% (10 µg/mL)	*A. linnaei*	PUA	[321]
Agelanin A **470**[β,η] [$C_{12}H_{11}Br_2N_5O_2$]	UV, MS, NMR	Pyrrole Alkaloid	Cytotoxic<br>Antibacterial	L5178Y<br>*S. epidermidis*	98.9% (23 mM)<br>MIC > 48 µM	*A. linnaei*	JSCR	[125]
Methyl 3,4-dibromo-1H-pyrrole-2-carboxylate **471**[β] [$C_6H_5Br_2NO_2$]	UV, MS, NMR	Pyrrole Alkaloid	Undetm.	Undetm.	Undetm.	*Acantho-stylotella* sp.	BLI	[322]
3,5-Dibromo-1H-pyrrole-2-carboxylic acid **472**[β,η] [$C_4H_3Br_2NO$]	UV, MS, NMR	Pyrrole Alkaloid	Undetm.	Undetm.	Undetm.	*Acantho-stylotella* sp.	BLI	[322]
Ethyl 3,4-dibromo-1H-pyrrole-2-carboxylate **473**[β,η] [$C_7H_7Br_2NO_2$]	MS, NMR	Pyrrole Alkaloid	Cytotoxic	L5178Y	77.2% (10 µg/mL)	*S. massa*	PUA	[321]
(−)-Longamide C **474**[β,η] [$C_{11}H_{13}BrN_2O_3$]	UV, MS, NMR, $[\alpha]_D$	Pyrrole Alkaloid	Cytotoxic	Undetm.	Undetm.	*A. nakamurai*	JSCR	[125]

Footnote: **1. Activity** (**MONO-MAC 6** human monocytic leukemia cells, **PC9** human lung cancer, **BHK** baby hamster kidney cells, **Aurora-A** serine/threonine-protein kinase, **Aurora-B** serine/threonine-protein kinase, **CDK2** cyclin-dependent kinase 2, **CDK4** cyclin-dependent kinase 4, **CDK5** cyclin-dependent kinase 5, **CDK9** cyclin-dependent kinase 9, **CK-1** creatine kinase 1, **COT** cancer Osaka thyroid oncogene, **DYRK1A** dual specificity tyrosine-phosphorylation regulated kinase 1A, **GSK-3** glycogen synthase kinase 3, **SRC** non-receptor tyrosine kinase, **PARP-3** poly(ADP-ribose) polymerase 3, **PDGFR** platelet-derived growth factor receptor, **SAK** serine/threonine-protein kinase, **CTFR** cystic fibrosis transmembrane conducatnce regulator, **corr**. correction).

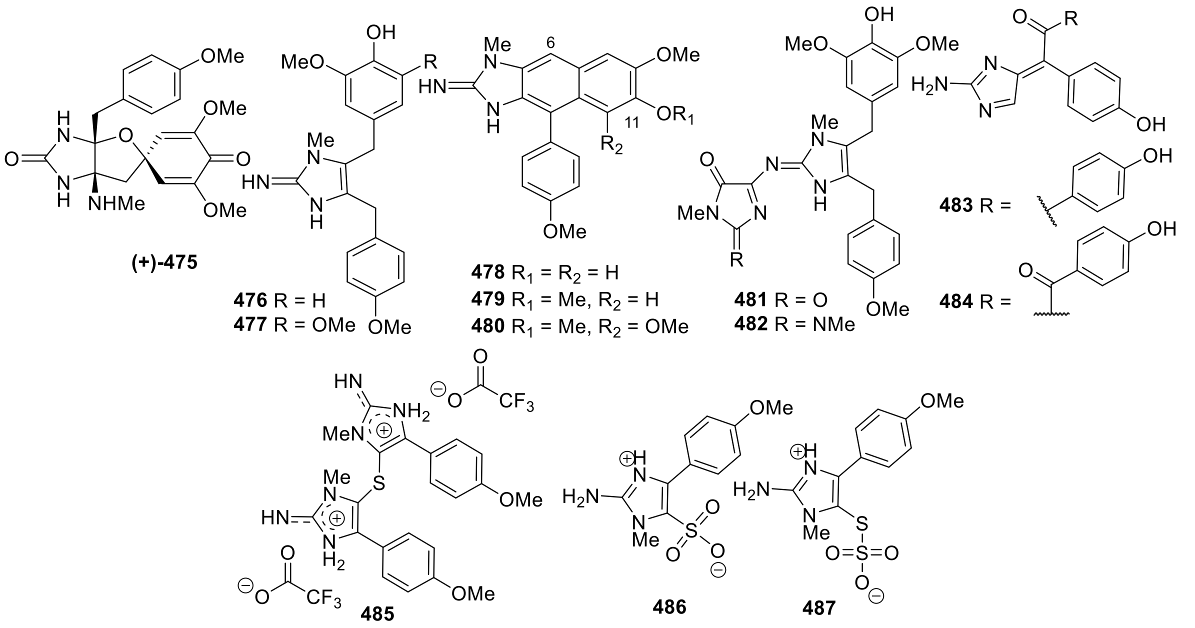

Figure S15: Structures of marine imidazole alkaloids from Indonesian waters found in 1970–2017.

Table S15: Marine imidazole alkaloids from Indonesian waters found in 1970–2017.

Compound	Structure Elucidation	Chemistry Type	Drug Class	Biological Activity		Source of Organism	Province	Ref
				Cell/Enzyme/Micro-organism/Insect/Others	Activity			
(+)-Spironaamidine **475**[β,η]<br>[$C_{21}H_{25}N_3O_6$]	MS, NMR, $[\alpha]_D$	Imidazole Alkaloid♠	Antibacterial	*B. cereus*	12 mm (10 mg/disk)	*L. micrographis*	NSW	[323]
Naamine F **476**[β,θ]<br>[$C_{20}H_{23}N_3O_3$]	UV, MS, NMR	Imidazole Alkaloid	Undetm.	Undetm.	Undetm.	*L. chagosensis*	SSW	[324]
Naamine G **477**[β,θ]<br>[$C_{21}H_{25}N_3O_4$]	UV, MS, NMR	Imidazole Alkaloid	Cytotoxic<br><br><br>Antifungal	L5178Y, HeLa<br>PC12<br>*A. salina*<br>*C. herbarum*	29 – 46% (10 µg/mL)<br>NA<br>10% (10 µg/mL)<br>20 mm (20 µg/disk)	*L. chagosensis*	SSW	[324, 325]
Kealiinine A **478**[β,η]<br>[$C_{20}H_{19}N_3O_3$]	UV, MS, NMR	Imidazole Alkaloid	Cytotoxic<br>Antifungal	*A. salina*<br>*C. herbarum*	50% (10 µg/mL)<br>NA (20 µg)	*L. chagosensis*	SSW	[324, 326]
Kealiinine B **479**[β,η]<br>[$C_{21}H_{21}N_3O_3$]	UV, MS, NMR	Imidazole Alkaloid	Cytotoxic	MCF10A, MCF7, T47D, MDA-MB-231	IC_{50} = 10.0 – 13.3 µM	*L. chagosensis*	SSW	[324, 326, 327]
Kealiinine C **480**[β,η]<br>[$C_{22}H_{23}N_3O_4$]	UV, MS, NMR	Imidazole Alkaloid	Cytotoxic	MCF10A, MCF7, T47D, MDA-MB-231	IC_{50} > 50 µM	*L. chagosensis*	SSW	[324, 326, 327]
Naamidine H **481**[β,η]<br>[$C_{25}H_{27}N_5O_6$]	UV, IR, MS, NMR	Imidazole Alkaloid	Cytotoxic<br>Antibacterial	HeLa<br>*E. coli*<br>*B. cereus*	IC_{50} = 5.6 µg/mL<br>NA (50 µg/disk)<br>8 mm (10 mg/disk)	*L. chagosensis*	NSW	[328]
Naamidine I **482**[β,η]<br>[$C_{26}H_{30}N_6O_5$]	UV, IR, MS, NMR	Imidazole Alkaloid	Cytotoxic<br>Antibacterial	HeLa<br>*E. coli*	IC_{50} = 15 µg/mL<br>NA (50 µg/disk)	*L. chagosensis*	NSW	[328]
Lissodendrin A **483**[β,η]<br>[$C_{17}H_{13}N_3O_3$]	UV, MS, NMR	Imidazole Alkaloid♣	Cytotoxic	L5178Y	NA (10 µg/mL)	*Lisso-dendoryx* (*Acantho-doryx*) *fibrosa*	MLU	[81]
Lissodendrin B **484**[β,η]<br>[$C_{17}H_{13}N_3O_4$]	UV, MS, NMR	Imidazole Alkaloid♦	Cytotoxic	L5178Y	NA (10 µg/mL)	*Lisso-dendoryx* (*Acantho-doryx*) *fibrosa*	MLU	[81]

Table S15: *Cont.*

Compound	Structure Elucidation	Chemistry Type	Drug Class	Biological Activity: Cell/Enzyme/Micro-organism/Insect/Others	Biological Activity: Activity	Source of Organism	Province	Ref
Polycarpaurine A **485**[β,θ] [$C_{22}H_{26}N_6O_2S_2$]	UV, IR, MS, NMR	Imidazole Alkaloid[♠]	Cytotoxic	V79	EC_{50} = 6.8 µM	*P. aurata*	NSW	[329, 330]
			Antiviral[µ] *in vitro*	TMV	NA (100 µg/mL)<br>14 ± 2% (500 µg/mL)<br>28 ± 2% inactivation (500 µg/mL)			
			Antiviral[µ] *in vivo*	TMV	33 ± 2% cur. (500 µg/mL)<br>24 ± 2% protection (500 µg/mL)			
			Antifungal *in vitro*[µ]	*F. oxysporium f.sp.cucumeris*, *C. arachidicola* Hori, *P. piricola*, *A. solani*, *F. graminearum*, *F. moniliforme*, *S. sclerotiorum*, *P. capsici*, *R. cerealis*, *B.maydis*, *W. Anthracnose*, *P. infestans*, *R. solani*, *B. cinerea*	6 ± 2 − 48 ± 1% (50 mg/kg)			
			Antifungal *in vivo*[µ]	*P. capsici*, *R. cerealis*, *B. graminis f. sp. tritici*, *S. sclerotiorum*, *R. solani*, *B. cinerea*, *C. cassiicola*	8 ± 2 − 30 ± 1% (200 mg/kg)			
Polycarpaurine B **486**[β,θ] [$C_{22}H_{26}N_6O_2S_2$]	UV, IR, MS, NMR	Imidazole Alkaloid	Cytotoxic	V79	EC_{50} > 10 µM	*P. aurata*	NSW	[329, 330]
Polycarpaurine C **487**[β,θ] [$C_{11}H_{13}N_3O_4S_2$]	UV, IR, MS, NMR	Imidazole-Alkaloid[♦]	Cytotoxic	V79	EC_{50} = 8.6 µM	*P. aurata*	NSW	[329, 330]
			Antiviral *in vitro*	TMV	NA (100 µg/mL)<br>23 ± 2% (500 µg/mL)<br>30 ± 2% inactivation (500 µg/mL)<br>15 ± 1% cur. (500 µg/mL)<br>34 ± 2% protection (500 µg/mL) (*in vivo*)			

Table S15: *Cont.*

Compound	Structure Elucidation	Chemistry Type	Drug Class	Biological Activity		Source of Organism	Province	Ref
				Cell/Enzyme/Micro-organism/Insect/Others	Activity			
Polycarpaurine C **487**[β,θ] [$C_{11}H_{13}N_3O_4S_2$]	UV, IR, MS, NMR	Imidazole-Alkaloid♦	Antifungal *in vitro*	*F. oxysporium f.sp.cucumeris, C. arachidicola* Hori, *P. piricola, A. solani, F. graminearum, F. moniliforme, S. sclerotiorum, P. capsici, R. cerealis, R. solani, B. maydis, W. Anthracnose, P. infestans, B. cinerea*	2 ± 2 − 40 ± 2% (50 mg/kg)	*P. aurata*	NSW	[329, 330]
			Antifungal *in vivo*	*P. capsici, R. cerealis, B. graminis f. sp. tritici, S. sclerotiorum, R. solani, B. cinerea, C. cassiicola*	0 − 30 ± 2% (200 mg/kg)			
				TMV	34 ± 2% protection (500 µg/mL) (*in vivo*)			

Footnote: **1. Activity** ([µ]Activity tested chloride salt, **MCF10A** human normal breast cell, **T47D** human breast adenocarcinoma, **TMV** tobacco mosaic virus, **cur.** curative).

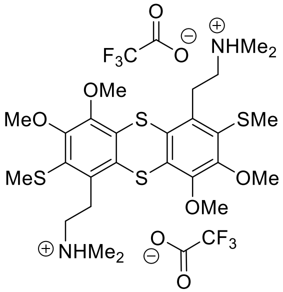

488

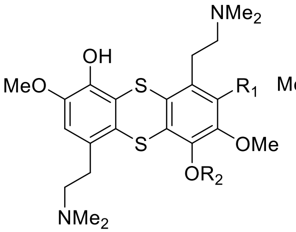

489 R_1 = SMe, R_2 = Me
491 R_1 = H, R_2 = Me

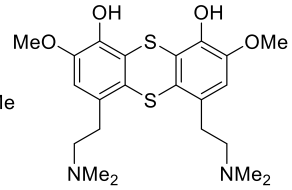

490

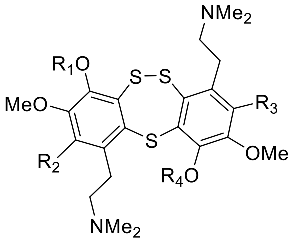

492 $R_1 = R_4$ = Me, $R_2 = R_3$ = SMe
493 $R_1 = R_2 = R_3 = R_4$ = H
494 $R_1 = R_2$ = H, R_3 = SMe, R_4 = Me

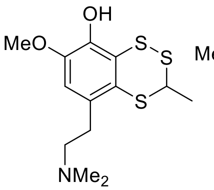

(–)-495

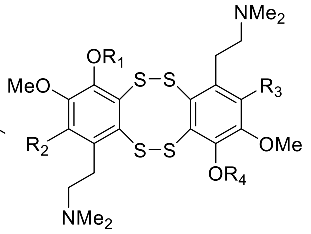

496 $R_1 = R_2 = R_3 = R_4$ = H
499 R_1 = Me, $R_2 = R_3$ = SMe,R_4 = Me

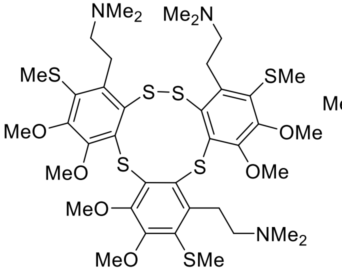

(–)-498

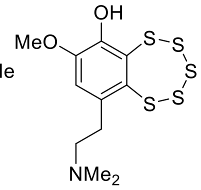

499

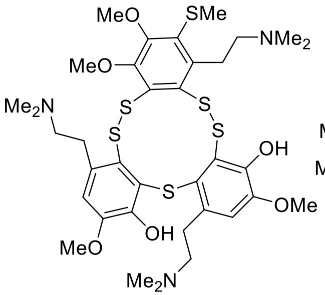

500

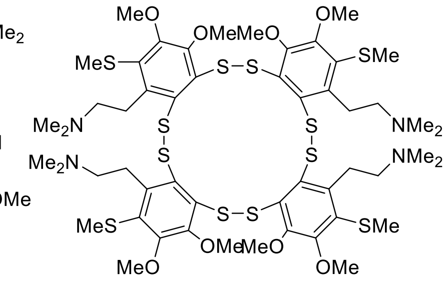

501

Figure S16: Structures of marine polysulfur aromatic alkaloids from Indonesian waters found in 1970–2017.

Table S16: Marine polysulfur aromatic alkaloids from Indonesian waters found in 1970–2017.

Compound	Structure Elucidation	Chemistry Type	Drug Class	Biological Activity: Cell/Enzyme/Micro-organism/Insect/Others	Biological Activity: Activity	Source of Organism	Province	Ref
Lissoclibadin 3 **488**[β,θ] [$C_{26}H_{38}N_2O_4S_5$]	UV, IR, MS, NMR	Polysulfur Aromatic Alkaloid	Antifungal	*M. hiemalis* IAM 6088	NA (20 − 50 µg/disk)	L. cf. *badium*	NSW	[331 – 333, 334]
				S. cerevisiae IAM 1438T	7.8 mm (50 µg/disk) NA (20 µg/disk)			
			Antibacterial	*S. aureus* IAM 12544T	10 − 13.8 mm (20 − 50 µg/disk)			
				E. coli IAM 12119T	10.8 mm (50 µg/disk) NA (20 µg/disk)			
			Cytotoxic	V79	IC_{50} = 0.34 µM			
				HCT-15, HeLa-S3	IC_{50} = 13.2 ± 1.4 – 16.0 ± 1.8 µM			
				T-47D, MDA-MB-231, NCI-H460, ACHN, UO31, DLD-1, HCT116, MALME-3M	IC_{50} = 1.22 − 3.59 µM			
				PC3, HL-60	IC_{50} = 5.50 − 7.02 µM			
Lissoclibadin 6 **489**[β,θ] [$C_{24}H_{34}N_2O_4S_3$]	UV, IR, MS, NMR	Polysulfur Aromatic Alkaloid	Antifungal	*M. hiemalis* IAM 6088	NA (20 − 50 µg/disk)	L. cf. *badium*	NSW	[335]
				S. cerevisiae IAM 1438T	7.8 mm (50 µg/disk) NA (20 µg/disk)			
			Antibacterial	*S. aureus* IAM 12544T	10 − 13.8 mm (20 − 50 µg/disk)			
				E. coli IAM 12119T	10.8 (50 µg/disk) NA (20 µg/disk)			
			Cytotoxic	V79	EC_{50} = 0.06 µM			
Lissoclibadin 11 **490**[β,θ] [$C_{24}H_{34}N_2O_4S_3$]	UV, IR, MS, NMR	Polysulfur Aromatic Alkaloid	Cytotoxic	V79, L1210	IC_{50} > 20 µM	L. cf. *badium*	NSW	[336]
Lissoclibadin 12 **491**[β,θ] [$C_{22}H_{30}N_2O_4S_2$]	UV, IR, MS, NMR	Polysulfur Aromatic Alkaloid	Cytotoxic	V79	IC_{50} = 7.90 µM	L. cf. *badium*	NSW	[336]
				L1210	IC_{50} > 20 µM			
Lissoclibadin 2 **492**[β,θ] [$C_{26}H_{38}N_2O_4S_5$]	UV, IR, MS, NMR, Mol. Mod.	Polysulfur Aromatic Alkaloid	Antifungal	*M. hiemalis* IAM 6088	13.8 mm (50 µg/disk) NA (20 µg/disk)	L. cf. *badium*	NSW	[331, 332, 334]

Table S16: *Cont.*

Compound	Structure Elucidation	Chemistry Type	Drug Class	Biological Activity: Cell/Enzyme/Micro-organism/Insect/Others	Biological Activity: Activity	Source of Organism	Province	Ref
Lissoclibadin 2 **492**[β,θ] [$C_{26}H_{38}N_2O_4S_5$]	UV, IR, MS, NMR, Mol. Mod.	Polysulfur Aromatic Alkaloid	Antibacterial	*R. atlantica* TUF-D	12.2 − 28.2 mm (5 − 50 μg/disk)	*L.* cf. *badium*	NSW	[331, 332, 334]
				S. cerevisiae IAM 1438T, *E. coli* IAM 12119T	NA (20, 50 μg/disk)			
				S. aureus IAM 12544T	NA (50 μg/disk)			
			Cytotoxic	T-47D, MDA-MB-231, NCI-H460, PC3, ACHN, UO31, DLD-1, HCT116, MALME-3M	IC_{50} = 0.10 − 0.77 μM			
				HL-60	20 − 90% (1 − 10 μM) IC_{50} = 0.21 μM			
				V79	IC_{50} = 0.08 μM			
			Anti-inflammatory	IL-8 (PMA, HL-60)	3 − 10 μM			
Lissoclibadin 4 **493**[β,θ] [$C_{22}H_{30}N_2O_4S_3$]	UV, IR, MS, NMR, Mol. Mod.	Polysulfur Aromatic Alkaloid	Antifungal	*M. hiemalis* IAM 6088	NA (20, 50 μg/disk)	*L.* cf. *badium*	NSW	[333, 335]
				S. cerevisiae IAM 1438T	NA (20, 50 μg/disk)			
			Antibacterial	*S. aureus* IAM 12544T	10.8 mm (20 μg/disk)			
				E. coli IAM 12119T	15.7 mm (50 μg/disk) NA (20 μg/disk)			
			Cytotoxic	V79	EC_{50} = 0.71 μM			
				HCT-15, HeLa-S3	IC_{50} = 17.2 ± 5.2 − 17.8 ± 5.3 μM			
Lissoclibadin 5 **494**[β,θ] [$C_{24}H_{34}N_2O_4S_4$]	UV, IR, MS, NMR	Polysulfur Aromatic Alkaloid	Antifungal	*M. hiemalis* IAM 6088	NA (20, 50 μg/disk)	*L.* cf. *badium*	NSW	[335]
				S. cerevisiae IAM 1438T	NA (20, 50 μg/disk)			
			Antibacterial	*S. aureus* IAM 12544T	10.8 − 13.1 mm (20 − 50 μg/disk)			
				E. coli IAM 12119T	15.7 mm (50 μg/disk)			
				E. coli IAM 12119T	NA (20 μg/disk)			
			Cytotoxic	V79	EC_{50} = 0.71 μM			
(−)-Lissoclibadin 13 **495**[β,θ] [$C_{13}H_{19}NO_2S_3$]	UV, IR, MS, NMR	Polysulfur Aromatic Alkaloid	Cytotoxic	V79, L1210	IC_{50} = 0.44 − 2.20 μM	*L.* cf. *badium*	NSW	[336]

Table S16: *Cont.*

Compound	Structure Elucidation	Chemistry Type	Drug Class	Biological Activity		Source of Organism	Province	Ref
				Cell/Enzyme/Micro-organism/Insect/Others	Activity			
Lissoclibadin 7 **496**[β,θ] [$C_{22}H_{30}N_2O_4S_4$]	UV, IR, MS, NMR	Polysulfur Aromatic Alkaloid	Antifungal	*M. hiemalis* IAM 6088	NA (20 − 50 µg/disk)	*L.* cf. *badium*	NSW	[333, 335]
				S. cerevisiae IAM 1438T	16.8 mm (50 µg/disk) NA (20 µg/disk)			
			Antibacterial	*S. aureus* IAM 12544T	9.4 − 13.1 mm (20 − 50 µg/disk)			
				E. coli IAM 12119T	9.9 mm (50 µg/disk) NA (20 µg/disk)			
			Cytotoxic	V79	EC_{50} = 0.17 µM			
				HCT-15, HeLa-S3	IC_{50} = 14.2 ± 1.3 – 15.7 ± 1.2 µM			
Lissoclibadin 10 **497**[β,θ] [$C_{26}H_{38}N_2O_4S_6$]	UV, IR, MS, NMR	Polysulfur Aromatic Alkaloid	Undetm.	Undetm.	Undetm.	*L.* cf. *badium*	NSW	[336]
(−)-Lissoclibadin 1 **498**[β,θ] [$C_{39}H_{57}N_3O_6S_7$]	UV, IR, MS, NMR, Mol. Mod	Polysulfur Aromatic Alkaloid♣	Antifungal	*M. hiemalis* IAM 6088	NA (20 − 50 µg/disk)	*L.* cf. *badium*	NSW	[330 – 333]
			Antibacterial	*R. atlantica* TUF-D	15.2 − 28.2 mm (20 − 50 µg/disk) NA (5 µg/disk)			
				S. cerevisiae IAM 1438T	NA (20 − 50 µg/disk)			
				S. aureus IAM 12544T	NA (50 µg/disk)			
				E. coli IAM 12119T	NA (20 − 50 µg/disk)			
			Cytotoxic	HCT-15, HeLa-S3, MCF7, NCI-H28	IC_{50} = 4.0 ± 1.8 – 7.6 ± 4.0 µM			
				V79, HL-60, T-47D, MDA-MB-231, NCI-H460, PC3, ACHN, UO31, DLD-1, HCT116, MALME-3M	IC_{50} = 0.13 – 0.82 µM			
				HCT-15	Apop. 5 µM (24 h) caspa-se-dependent pathway			
				Nude mice (HCT)	60% (25 mg/kg per day wo. signt. secon. adv. eff)			
Lissoclibadin 14 **499**[β,θ] [$C_{11}H_{15}NO_2S_5$]	UV, IR, MS, NMR	Polysulfur Aromatic Alkaloid♣	Cytotoxic	V79, L1210	IC_{50} = 0.70 − 1.80 µM	*L.* cf. *badium*	NSW	[336]
				HCT-15, HeLa-S3, MCF7, NCI-H28	IC_{50} = 4.2 ± 2.4 – 6.4 ± 2.7 µM			

Table S16: *Cont.*

Compound	Structure Elucidation	Chemistry Type	Drug Class	Biological Activity: Cell/Enzyme/Micro-organism/Insect/Others	Biological Activity: Activity	Source of Organism	Province	Ref
Lissoclibadin 9 **500**[β,θ] [$C_{35}H_{49}N_3O_6S_6$]	UV, IR, MS, NMR, Mol. Mod.	Polysulfur Aromatic Alkaloid	Cytotoxic	V79, L1210	IC_{50} = 0.38 − 0.63 μM	*L.* cf. *badium*	NSW	[336]
Lissoclibadin 8 **501**[β,θ] [$C_{52}H_{76}N_4O_8S_{12}$]	UV, IR, MS, NMR, Mol. Mod.	Polysulfur Aromatic Alkaloid[♣]	Cytotoxic	V79, L1210<br>HCT-15, HeLa-S3, MCF7, NCI-H28	IC_{50} = 0.14 – 2.00 μM<br>IC_{50} = 4.9 ± 1.9 – 11.8 ± 3.1 μM	*L.* cf. *badium*	NSW	[336]

Footnote: **1. Activity (ACHN** human renal carcinoma, **DLD-1** human colorectal adenocarcinoma, **HeLa-S3** human cervical adenocarcinoma, **MALME-3M** human melanoma cancer, **NCI-H28** human non-small cell lung cancer, **IL-8** interleukin 8, **PMA** phorbol myristate acetate).

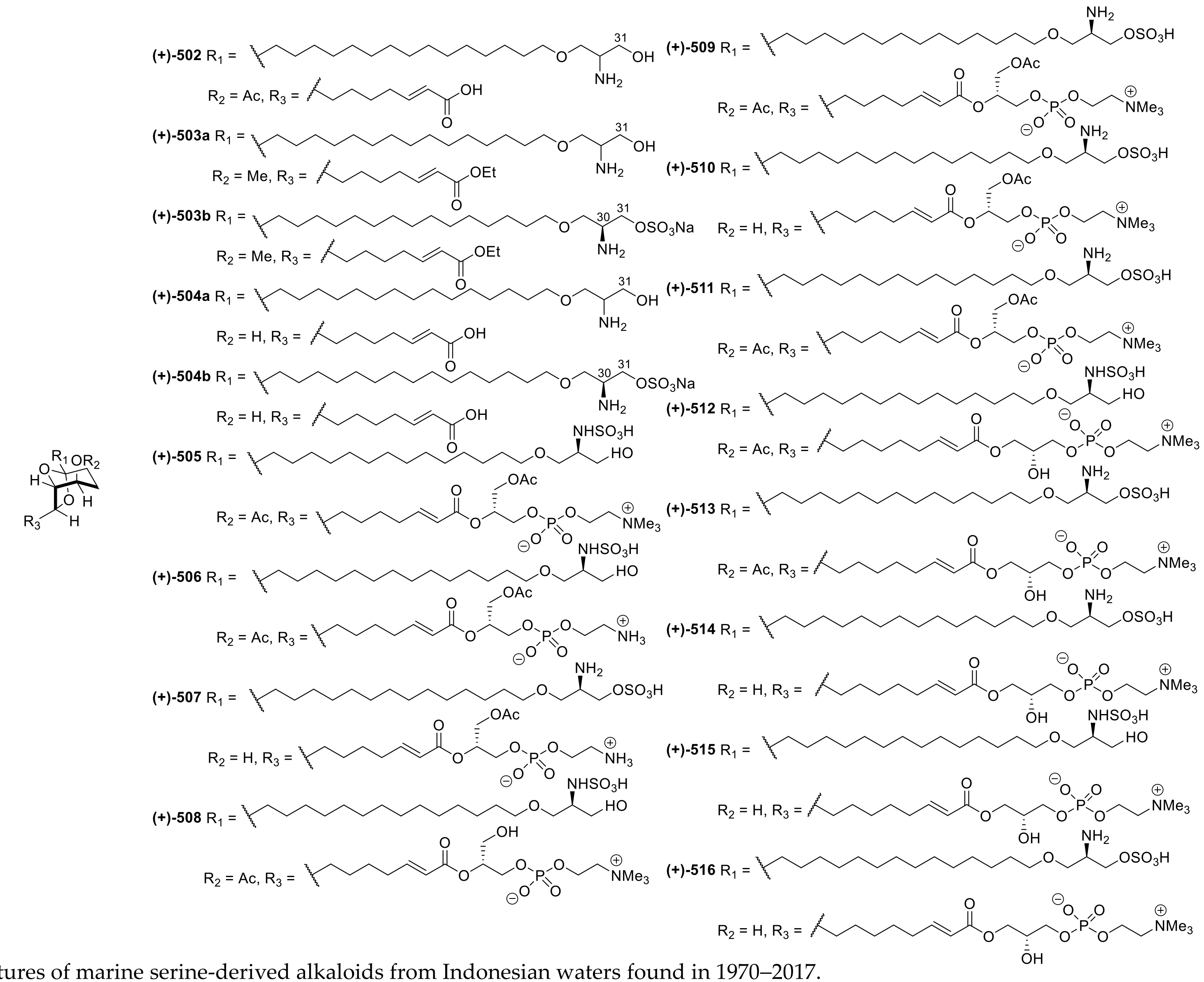

Figure S17: Structures of marine serine-derived alkaloids from Indonesian waters found in 1970–2017.

Table S17: Marine serine-derived alkaloids from Indonesian waters found in 1970–2017.

Compound	Structure Elucidation	Chemistry Type	Drug Class	Biological Activity		Source of Organism	Province	Ref
				Cell/Enzyme/Micro-organism/Insect/Others	Activity			
(+)-Didemniserinolipid A **502**$^{\beta}$ [$C_{33}H_{59}NO_8$]	UV, IR, MS, NMR, $[\alpha]_D$	Serine Alkaloid♣	Cytotoxic	P-388, A549, HT-29	IC_{50} > 20 µg/mL	*Didemnum* sp.	NMU	[337]
(+)-Didemniserinolipid B **503a**$^{\beta}$ [$C_{34}H_{63}NO_7$]	UV, IR, MS, NMR, $[\alpha]_D$	Serine Alkaloid	Cytotoxic	P-388, A549, HT-29	IC_{50} > 20 µg/mL	*Didemnum* sp.	NMU	[337]
(+)-Didemniserinolipid B **503b**$^{\gamma}$ [$C_{34}H_{62}NNaO_{10}S$]	TS	Serine Alkaloid	Undetm.	Undetm.	Undetm.			[338, 339]
(+)-Didemniserinolipid C **504a**$^{\beta}$ [$C_{31}H_{57}NO_7$]	UV, IR, MS, NMR, $[\alpha]_D$	Serine Alkaloid	Cytotoxic	P-388, A549, HT-29	IC_{50} > 20 µg/mL	*Didemnum* sp.	NMU	[337]
(+)-Didemniserinolipid C **504b**$^{\gamma}$ [$C_{31}H_{56}NNaO_{10}S$]	TS	Serine Alkaloid	Undetm.	Undetm.	Undetm.			[339]
(+)-Siladenoserinol A **505**$^{\beta}$ [$C_{43}H_{79}N_2O_{17}PS$]	UV, IR, MS, NMR, ECD, $[\alpha]_D$, CT, TS	Serine Alkaloid♦,♠	Anticancer	p53-Hdm	IC_{50} = 2.0 − 17 µM (natural) IC_{50} = 17 µM (synthetic)	A tunicate (Didem-nidae)	NSW	[340, 341]
(+)-Siladenoserinol B **506**$^{\beta}$ [$C_{40}H_{73}N_2O_{17}PS$]	UV, IR, MS, NMR, $[\alpha]_D$	Serine Alkaloid	Anticancer	p53-Hdm	IC_{50} = 2.0 µM	A tunicate (Didem-nidae)	NSW	[340]
(+)-Siladenoserinol C **507**$^{\beta}$ [$C_{38}H_{71}N_2O_{16}PS$]	UV, IR, MS, NMR, $[\alpha]_D$	Serine Alkaloid	Anticancer	p53-Hdm	IC_{50} = 4.0 µM	A tunicate (Didem-nidae)	NSW	[340]
(+)-Siladenoserinol D **508**$^{\beta}$ [$C_{41}H_{77}N_2O_{16}PS$]	UV, IR, MS, NMR, $[\alpha]_D$	Serine Alkaloid	Anticancer	p53-Hdm	IC_{50} = 7.7 µM	A tunicate (Didem-nidae)	NSW	[340]
(+)-Siladenoserinol E **509**$^{\beta}$ [$C_{41}H_{77}N_2O_{16}PS$]	UV, IR, MS, NMR, $[\alpha]_D$	Serine Alkaloid	Anticancer	p53-Hdm	IC_{50} = 18.0 µM	A tunicate (Didem-nidae)	NSW	[340]
(+)-Siladenoserinol F **510**$^{\beta}$ [$C_{41}H_{77}N_2O_{16}PS$]	UV, IR, MS, NMR, $[\alpha]_D$	Serine Alkaloid	Anticancer	p53-Hdm	IC_{50} = 29.0 µM	A tunicate (Didem-nidae)	NSW	[340]

Table S17: *Cont.*

Compound	Structure Elucidation	Chemistry Type	Drug Class	Biological Activity: Cell/Enzyme/Micro-organism/Insect/Others	Biological Activity: Activity	Source of Organism	Province	Ref
(+)-Siladenoserinol G **511**[β] [$C_{43}H_{79}N_2O_{17}PS$]	UV, IR, MS, NMR, $[\alpha]_D$	Serine Alkaloid	Anticancer	p53-Hdm	IC_{50} = 53.0 μM	A tunicate (Didem-nidae)	NSW	[340]
(+)-Siladenoserinol H **512**[β] [$C_{41}H_{77}N_2O_{16}PS$]	UV, IR, MS, NMR, $[\alpha]_D$	Serine Alkaloid	Anticancer	p53-Hdm	IC_{50} = 2.5 μM	A tunicate (Didem-nidae)	NSW	[340]
(+)-Siladenoserinol I **513**[β] [$C_{41}H_{77}N_2O_{16}PS$]	UV, IR, MS, NMR, $[\alpha]_D$	Serine Alkaloid	Anticancer	p53-Hdm	IC_{50} = 9.3 μM	A tunicate (Didem-nidae)	NSW	[340]
(+)-Siladenoserinol J **514**[β] [$C_{36}H_{69}N_2O_{15}PS$]	UV, IR, MS, NMR, $[\alpha]_D$	Serine Alkaloid	Anticancer	p53-Hdm	IC_{50} = 11.0 μM	A tunicate (Didem-nidae)	NSW	[340]
(+)-Siladenoserinol K **515**[β] [$C_{39}H_{75}N_2O_{15}PS$]	UV, IR, MS, NMR, $[\alpha]_D$	Serine Alkaloid	Anticancer	p53-Hdm	IC_{50} = 13.0 μM	A tunicate (Didem-nidae)	NSW	[340]
(+)-Siladenoserinol L **516**[β] [$C_{39}H_{75}N_2O_{15}PS$]	UV, IR, MS, NMR, $[\alpha]_D$	Serine Alkaloid	Anticancer	p53-Hdm	IC_{50} = 55.0 μM	A tunicate (Didem-nidae)	NSW	[340]

Footnote: **1. Activity** (**p53** tumor protein).

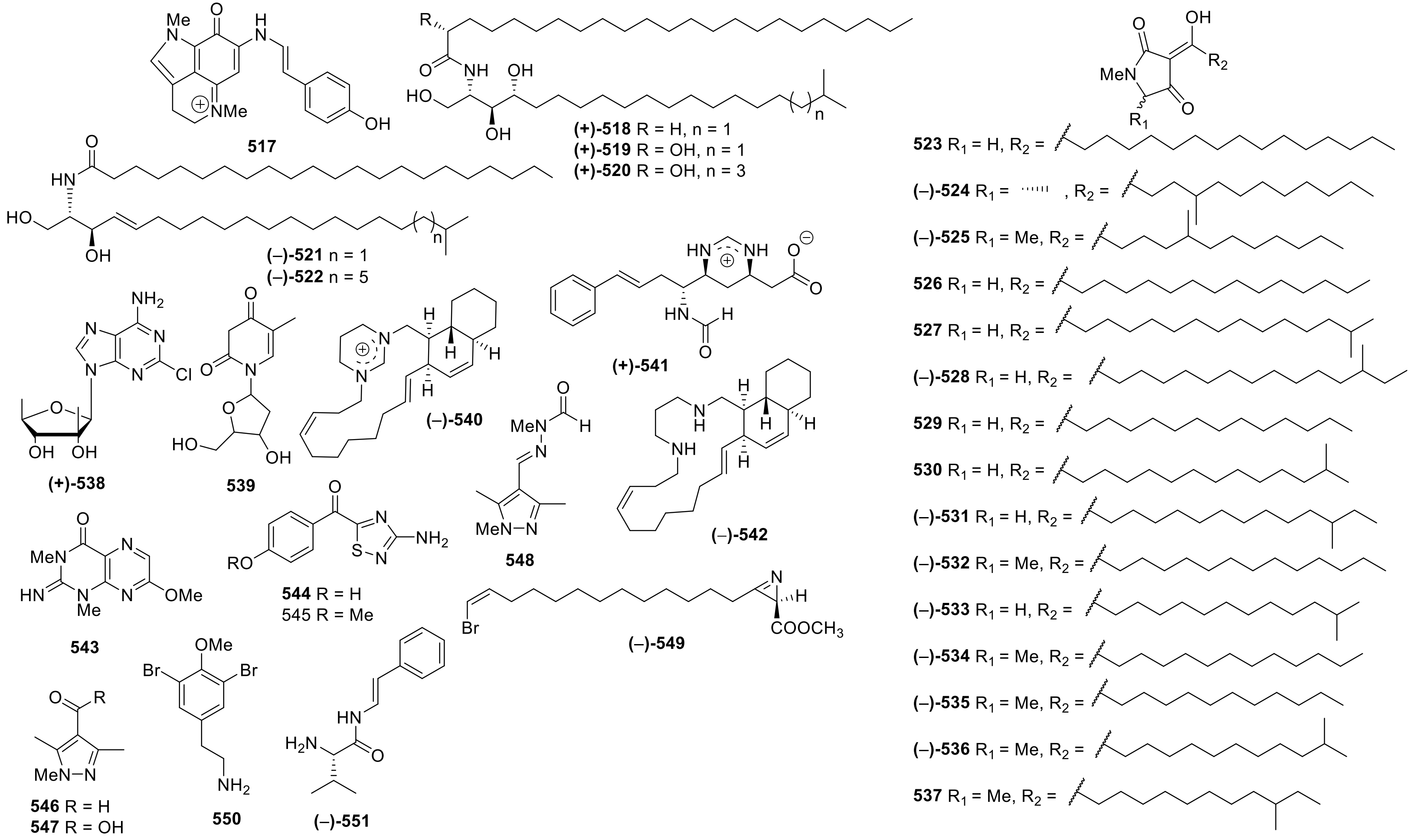

Figure S18: Structures of other marine alkaloids from Indonesian waters found in 1970–2017.

Table S18: Other marine alkaloids from Indonesian waters found in 1970–2017.

Compound	Structure Elucidation	Chemistry Type	Drug Class	Biological Activity: Cell/Enzyme/Micro-organism/Insect/Others	Biological Activity: Activity	Source of Organism	Province	Ref
Makavulamine G **517**$^{\beta,\kappa}$ [$C_{20}H_{20}N_3O_2$]	UV, IR, MS, NMR	Pyrroloimino-quinone Alkaloid	Cytotoxic	P-388, A549, HT-29, MCF7, KB	IC_{50} = 0.35 − 0.5 µg/mL	*Histodermella* sp.	NSW	[342–344]
			Antifungal	*A. oryzae*, *C. albicans*, *P. notatum*, *S cerevisiae*, *T. mentagrophytes*	NA			
			Antiviral	Herpes simplex 1, Herpes simplex 2, Polio virus	NA			
			Immuno modulatory	MLR	IC_{50} = 0.28 µg/mL			
				Murine resting lymphocyte	IC_{50} = 24.4 µg/mL			
			Anticancer	DNA topoisomerase-I	IC_{50} = 3.0 µM			
				DNA topoisomerase-II	NA			
				RNA, DNA	IC_{50} = 15 µM			
				protein synthesis	IC_{50} = 21 µM			
			Neurodisease	Affinity to *L. stagnalis* AchBP (radioligand assay)	K_i = 0.55 ± 0.01 µM			
				Affinity to *T. california* nAChR (radioligand assay)	K_i = 1.60 ± 0.17 µM			
				Affinity to human nAChR (radioligand assay)	K_i = 18 ± 2 µM			
			Electro-physiological activity	Murine muscle nAChR	IC_{50} = 3.3 ± 0.3 µM			
				Human nAChR expressed in *X. laevis*	40% (10 µM)			
			Stimulator at initial stage of development of agricultural plant	*H. vulgare* (root)	Active			
				F. esculentum Moench (root)	Active			
(+)-Strepsiamide A **518**$^{\beta}$ [$C_{43}H_{87}NO_4$]	IR, MS, NMR, $[\alpha]_D$, CT	Ceramide	Cytotoxic	L5178Y	ED_{50} = 7.6 µg/mL	*S. lendenfeldi*	UEP	[345]
				HeLa	4% (10 µg/mL) 3% (3 µg/mL)			
				PC12	NA			
(+)-Strepsiamide B **519**$^{\beta}$ [$C_{43}H_{87}NO_4$]	IR, MS, NMR, $[\alpha]_D$, CT	Ceramide	Cytotoxic	L5178Y	ED_{50} > 10 µg/mL	*S. lendenfeldi*	UEP	[345]
				HeLa, PC12	NA			

Table S18: *Cont.*

Compound	Structure Elucidation	Chemistry Type	Drug Class	Biological Activity: Cell/Enzyme/Micro-organism/Insect/Others	Biological Activity: Activity	Source of Organism	Province	Ref
(+)-Strepsiamide C **520**[β] [$C_{44}H_{89}NO_4$]	IR, MS, NMR, [α]D, CT	Ceramide	Cytotoxic	L5178Y<br>HeLa, PC12	68% (10 μg/mL)<br>NA	*S. lendenfeldi*	UEP	[345]
(−)-Iotrochotamide I **521**[β] [$C_{43}H_{85}NO_3$]	IR, MS, NMR, [α]D, CT	Ceramide	Undetm.	Undetm.	Undetm.	*I. purpurea*	SSW	[346]
(−)-Iotrochotamide II **522**[β] [$C_{47}H_{93}NO_3$]	IR, MS, NMR, [α]D, CT	Ceramide	Undetm.	Undetm.	Undetm.	*I. purpurea*	SSW	[346]
Melophlin A **523**[β] [$C_{21}H_{37}NO_3$]	UV, IR, MS, NMR, CT	Tetramic acid Alkaloid	Cytotoxic	HL-60<br>L1210, KB-3-1, A498, U-937, L-929<br>Revers. the morp. H-*ras* transf. NIH3T3 fibroblast to normal<br>Modulator signal transduction (Ras)<br>Arrest. NIH3T3 (G1)<br>Mg and Zn complex	0.2 − 0.4 μg/mL<br>IC_{50} = 8.55 − 26 μM<br>5 μg/mL<br>Dynamins II and I- like protein<br>1 μg/mL<br>Cytotoxic in various cancer cells	*M. sarassinorum*	SSW	[84 – 87, 347]
			Antibacterial	*M. smegmatis* (Glucose), *M. bovis* BCG (Glucose), *M. bovis* BCG (Palmitate)<br>*M. smegmatis* (Propionate), *M. bovis* BCG (Propionate),<br>*M. smegmatis* (Palmitate)	MIC = 25 μg/mL<br>MIC = 0.8 – 3.0 μg/mL<br>MIC = 12.5 μg/mL			
			Anti-inflammatory	V79<br>IL-8 (PMA, HL-60)	ED_{50} = 27.2 μM<br>NA			
(−)-Melophlin B **524**[β] [$C_{19}H_{33}NO_3$]	UV, IR, MS, NMR, ECD, [α]D, CT	Tetramic acid Alkaloid	Cytotoxic	HL-60<br>Revers. the morp. H-*ras* transf. NIH3T3 fibroblast to normal<br>Arrest. NIH3T3 at G1 phase	0.2 − 0.4 μg/mL<br>5 μg/mL<br>1 μg/mL	*M. sarassinorum*	SSW	[347]

Table S18: *Cont.*

Compound	Structure Elucidation	Chemistry Type	Drug Class	Biological Activity		Source of Organism	Province	Ref
				Cell/Enzyme/Micro-organism/Insect/Others	Activity			
(−)-Melophlin C **525**[β] $[C_{19}H_{33}NO_3]$	UV, IR, MS, NMR, ECD, $[\alpha]_D$, CT	Tetramic acid Alkaloid	Cytotoxic	HL-60, HeLa, TF-1<br>*A. salina*	NA<br>IC_{50} = 36.6 µg/mL	*M. sarassinorum*	SSW	[348]
			Antibacterial	*S. aureus*<br>*S. aureus*, *B. subtilis*	18 mm (5 µg/disk)<br>11 − 16 mm (5 − 10 µg/disk)			
			Antifungal	*C. albicans*	15 mm (10 µg/disk)			
			Antiinsecticidal	*S. littoralis*	M			
Melophlin D **526**[β] $[C_{20}H_{35}NO_3]$	UV, IR, MS, NMR	Tetramic acid Alkaloid	Undetm.	Undetm.	Undetm.	*M. sarassinorum*	SSW	[348]
Melophlin E **527**[β] $[C_{21}H_{37}NO_3]$	UV, IR, MS, NMR	Tetramic acid Alkaloid	Cytotoxic	HL-60, HeLa, TF-1	NA	*M. sarassinorum*	SSW	[348]
(−)-Melophlin F **528**[β] $[C_{21}H_{37}NO_3]$	UV, IR, MS, NMR, $[\alpha]_D$	Tetramic acid Alkaloid	Undetm.	Undetm.	Undetm.	*M. sarassinorum*	SSW	[348]
Melophlin G **529**[β] $[C_{19}H_{33}NO_3]$	UV, IR, MS, NMR	Tetramic acid Alkaloid	Cytotoxic	HL-60, HeLa, TF-1<br>*A. salina*	NA<br>IC_{50} = 48.8 µg/mL	*M. sarassinorum*	SSW	[348]
			Antibacterial	*S. aureus*<br>*B. subtilis*	9 − 10 mm (5 − 10 µg/disk)<br>15 mm (10 µg/disk)			
			Antiinsecticidal	*S. littoralis*	Moderate			
Melophlin H **530**[β] $[C_{20}H_{35}NO_3]$	UV, IR, MS, NMR	Tetramic acid Alkaloid	Cytotoxic	HL-60, HeLa, TF-1	NA	*M. sarassinorum*	SSW	[348]
(−)-Melophlin I **531**[β] $[C_{20}H_{35}NO_3]$	UV, IR, MS, NMR, $[\alpha]_D$	Tetramic acid Alkaloid	Cytotoxic	HL-60, HeLa, TF-1<br>*A. salina*	NA<br>IC_{50} = 52.6 µg/mL	*M. sarassinorum*	SSW	[348]
			Antibacterial	*S. aureus*, *B. subtilis*	9 mm (5 − 10 µg/disk)			
			Antiinsecticidal	*S. littoralis*	M			
(−)-Melophlin J **532**[β] $[C_{20}H_{35}NO_3]$	UV, IR, MS, NMR, $[\alpha]_D$, CT	Tetramic acid Alkaloid	Undetm.	Undetm.	Undetm.	*M. sarassinorum*	SSW	[348]
Melophlin K **533**[β] $[C_{19}H_{34}NO_3]$	UV, IR, MS, NMR,	Tetramic acid Alkaloid	Undetm.	Undetm.	Undetm.	*M. sarassinorum*	SSW	[348]
(−)-Melophlin L **534**[β] $[C_{19}H_{34}NO_3]$	UV, IR, MS, NMR, $[\alpha]_D$, CT	Tetramic acid Alkaloid	Undetm.	Undetm.	Undetm.	*M. sarassinorum*	SSW	[348]
(−)-Melophlin M **535**[β] $[C_{18}H_{31}NO_3]$	UV, IR, MS, NMR, $[\alpha]_D$, CT	Tetramic acid Alkaloid	Cytotoxic	HL-60, HeLa, TF-1	NA	*M. sarassinorum*	SSW	[348]
Melophlin N **536**[β] $[C_{19}H_{33}NO_3]$	UV, IR, MS, NMR, CT	Tetramic acid Alkaloid	Cytotoxic	HL-60, HeLa, TF-1	NA	*M. sarassinorum*	SSW	[348]

Table S18: *Cont.*

Compound	Structure Elucidation	Chemistry Type	Drug Class	Biological Activity: Cell/Enzyme/Micro-organism/Insect/Others	Biological Activity: Activity	Source of Organism	Province	Ref
Melophlin O **537**$^{\beta}$ [$C_{19}H_{33}NO_3$]	UV, IR, MS, NMR, CT	Tetramic acid Alkaloid	Undetm.	Undetm.	Undetm.	*M. sarassinorum*	SSW	[348]
(+)-Kumusine **538**$^{\beta}$ [$C_{11}H_{14}ClN_5O_3$]	UV, IR, MS, NMR, ECD, [α]D	Nucleoside Alkaloid♦	Imuuno suppressive activity<br>Cytotoxic	MLR<br>LcV<br>P-388, HT-29<br>A549, *C. aethiops* kidney cells<br>Myeloma cells	IC_{50} = 0.195 μg/mL<br>IC_{50} = 5.0 μg/mL, potency > 256<br>IC_{50} = 5.0 μg/mL<br>IC_{50} = 2.5 μg/mL<br>IC_{50} = 0.18 μg/mL	*Theonella* sp.	NSW	[349, 350]
1-(Tetrahydro-4-hydroxy-5-(hydroxymethyl)furan-2-yl)-5-methyl pyrimidine-2,4(1H,3H)-dione **539**$^{\beta}$ [$C_{11}H_{15}NO_5$]	IR, MS, NMR	Nucleoside Alkaloid	Cytotoxic	T47D, HeLa, Raji cells	IC_{50} = 5.8 − 7.9 μg/mL	*Kaliapsis* sp.	BLI	[351]
(−)-Neopetrocyclamine A **540**$^{\beta}$ [$C_{26}H_{41}N_2$]	IR, MS, NMR, [α]D, Mol. Mod.	Formamido Alkaloid♦	Cytotoxic	UO-31, A498, SF295	GI_{50} > 20 μg/mL	*N. exigua*	EKM	[352]
(+)-Lanesoic acid **541**$^{\beta}$ [$C_{17}H_{21}N_3O_3$]	UV, IR, MS, NMR, [α]D, QCC	Formamido Alkaloid♦	Cytotoxic	A549 (ATCC CCL-185), HT-29 (ATCC HTB-38), MDA-MB-231 (ATCC HTB-26)<br>PSN-1 (ATCC CRM-CRL-3211	NA<br>IC_{50} = 8.9 μg/mL	*Theonella* sp.	CSW	[353]
(−)-Neopetrocyclamine B **542**$^{\beta}$ [$C_{25}H_{42}N_2$]	IR, MS, NMR, [α]D	Polycylic diamine Alkaloid	Cytotoxic	UO-31, A498, SF295	GI_{50} > 20 μg/mL	*N. exigua*	EKM	[352]
1,3,*O*^{7}-Trimethyl isoxanthopterin **543**$^{\beta,\theta}$ [$C_9H_{11}N_5O_2$]	UV, IR, MS, NMR	Pterin Alkaloid	Undetm.	Undetm.	Undetm.	*Eudistoma* sp.	SSW	[354]
Polycarpathiamine A **544**$^{\beta}$ [$C_9H_7N_3O_2S$]	UV, IR, MS, NMR, CT	Thiadiazole Alkaloid♦	Cytotoxic	L5178Y	IC_{50} = 0.41 μM	*P. aurata*	MLU	[46]
Polycarpathiamine B **545**$^{\beta}$ [$C_{10}H_9N_3O_2S$]	UV, IR, MS, NMR	Thiadiazole Alkaloid	Cytotoxic	L5178Y	NA	*P. aurata*	MLU	[46]
Cinachyrazole A **546**$^{\zeta}$ [$C_7H_{10}N_2O$]	UV, MS, NMR	Pyrazole Alkaloid	Cytotoxic	L5178Y	NA (IC_{50} > 10 μM)	*Cinachyrella* sp.	MLU	[355]
Cinachyrazole B **547**$^{\zeta}$ [$C_7H_{10}N_2O_2$]	UV, MS, NMR	Pyrazole Alkaloid	Cytotoxic	L5178Y	NA (IC_{50} > 10 μM)	*Cinachyrella* sp.	MLU	[355]

Table 18. *Cont.*

Compound	Structure Elucidation	Chemistry Type	Drug Class	Biological Activity: Cell/Enzyme/Micro-organism/Insect/Others	Biological Activity: Activity	Source of Organism	Province	Ref
Cinachyrazole C **548**[β] [$C_9H_{14}N_4O$]	UV, MS, NMR	Pyrazole Alkaloid[♦]	Cytotoxic	L5178Y	NA (IC_{50} > 10 μM)	*Cinachyrella* sp.	MLU	[355]
(−)-Debromoantazirine **549**[β] [$C_{17}H_{28}BrNO_2$]	IR, MS, NMR, $[\alpha]_D$	Azirine Alkaloid[♠]	Cytotoxic	NBT-T2	IC_{50} = 4.7 μg/mL	*Dysidea* sp.	PUA	[355]
3′,5′-dibromo-4′-methoxyphenethylamine **550**[β,θ] [$C_9H_{11}Br_2NO$]	UV, MS, NMR, CT	Simple Amine/Amide Alkaloid	Undetm.	Undetm.	Undetm.	*Eudistoma* sp.	SSW	[287]
(−)-(*E*)-2-Amino-3-methyl-N-styrlbutanamide **551**[β] [$C_{13}H_{18}N_2O$]	UV, MS, NMR, $[\alpha]_D$	Simple Amine/Amide Alkaloid[♦]	Antibacterial	*E. faecium* BM4147-1, *S. aureus* ATCC29213 *K. pneumoniae* ATCC 12657, *E. aerogenes* ATCC 13048	MIC > 50 MIC > 100	*C. basilana*	MLU	[357]

__Footnote:__ **1. Activity** (**NIH3T3** murine fibroblast, **TF-1** human erythroleukemia, **U-937** human leukemia, **Raji cells** human Burkitt's lymphoma, **PSN-1** human pancreas carcinoma, **A498** human kidney carcinoma, **Revers.** reversing, **Arres.** arresting).

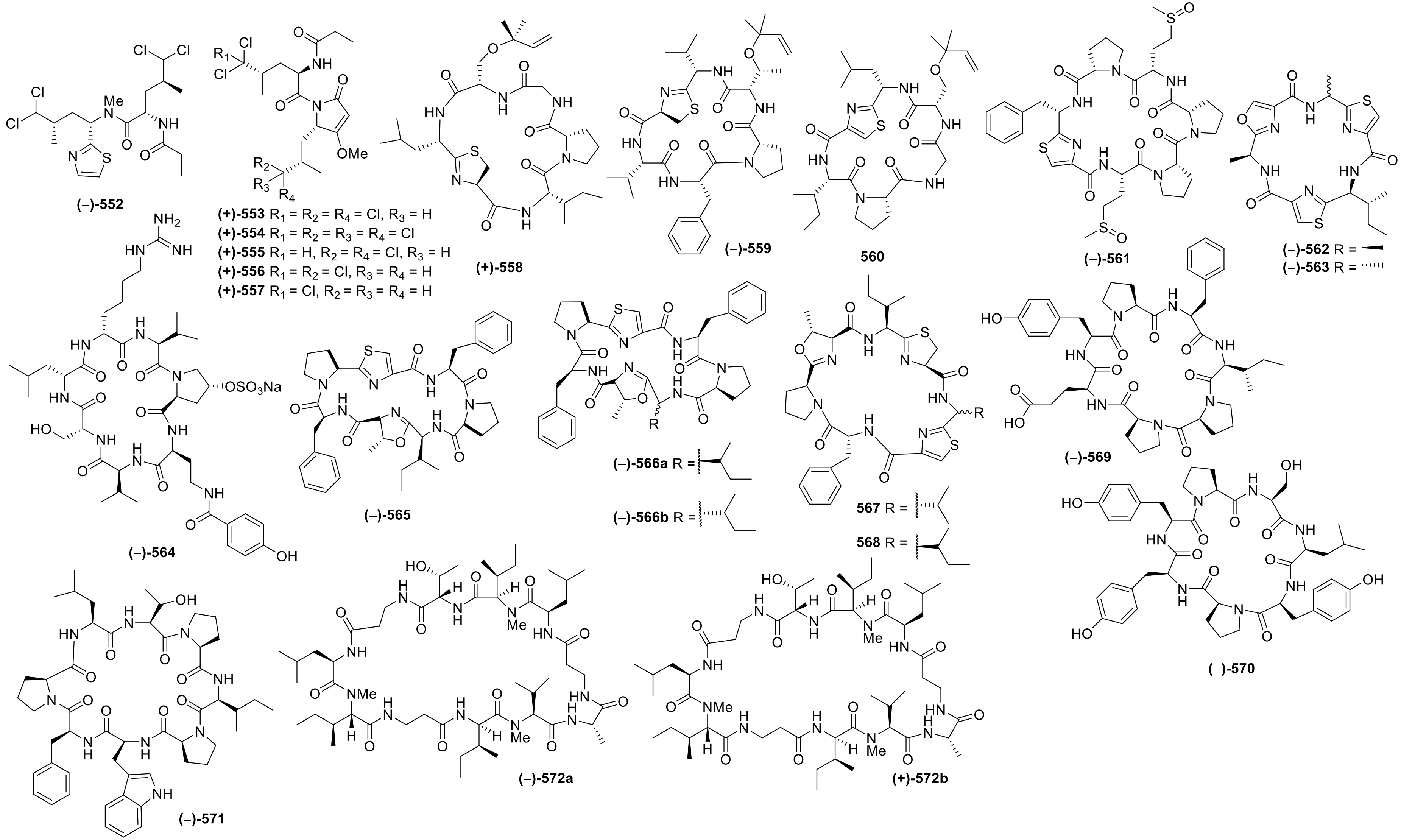

Figure S19: *Cont.*

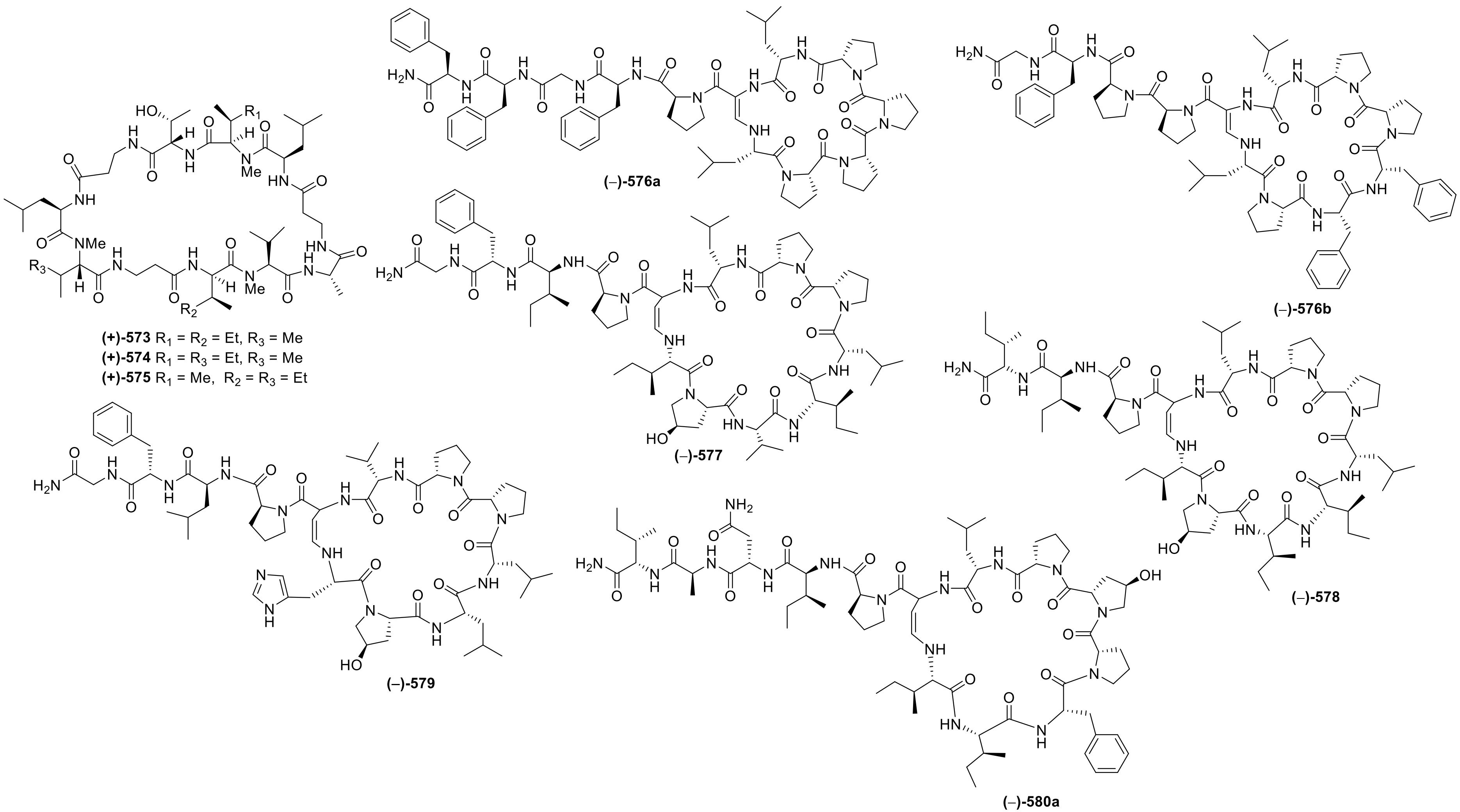

Figure S19: *Cont.*

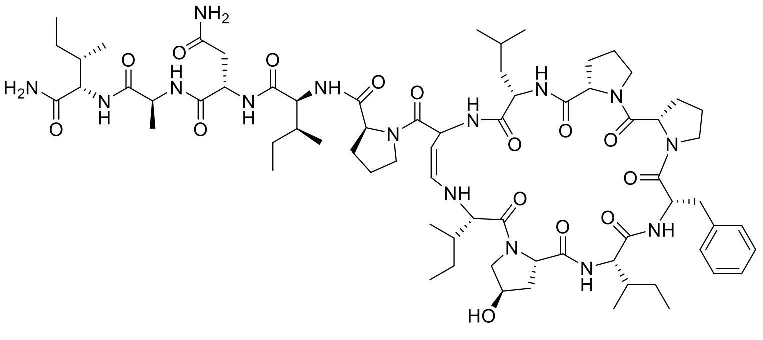

(–)-580b

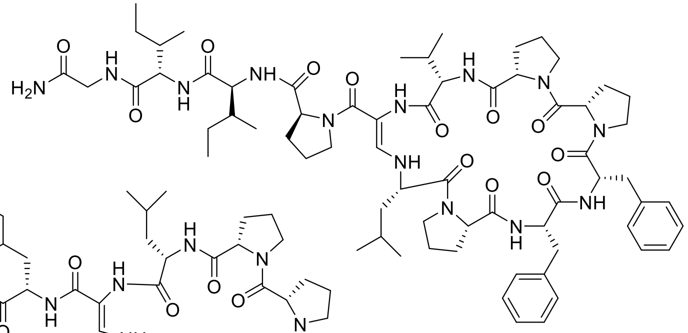

(–)-581

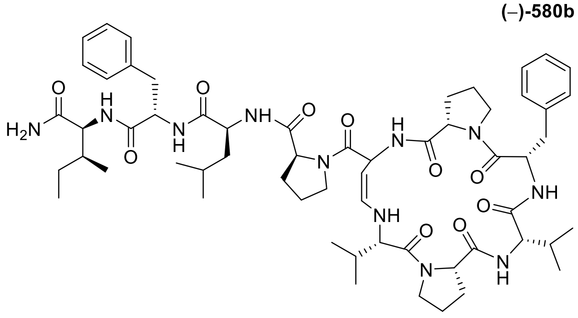

(–)-582a

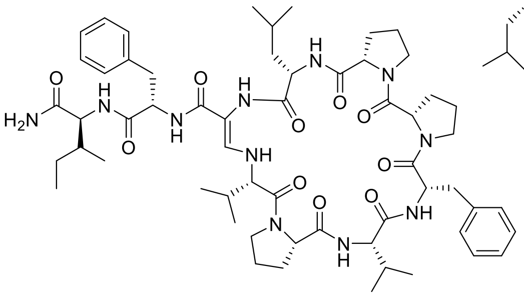

(–)-582b

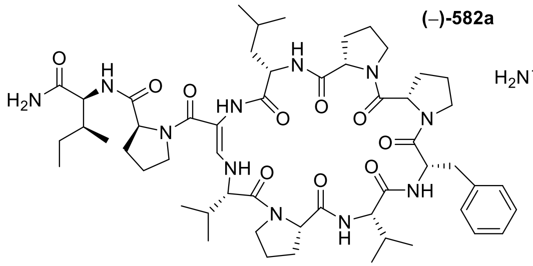

(–)-583

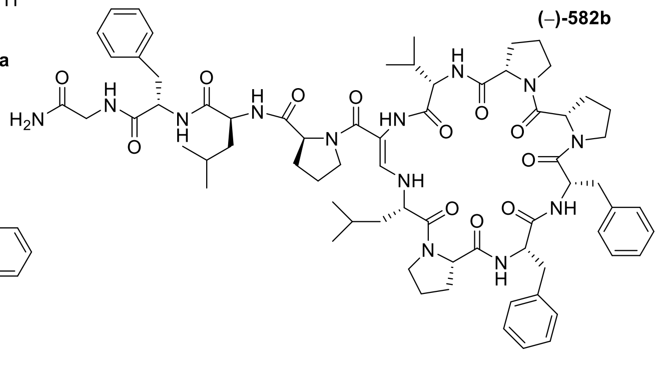

(–)-584

Figure S19: *Cont.*

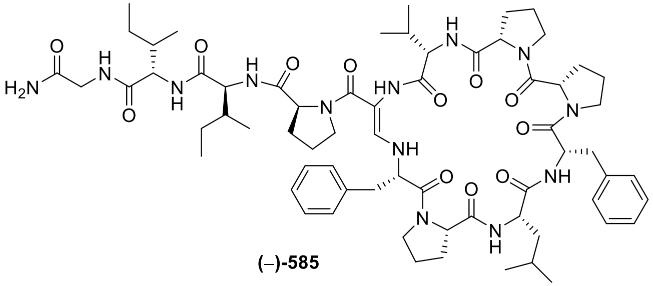

(–)-585

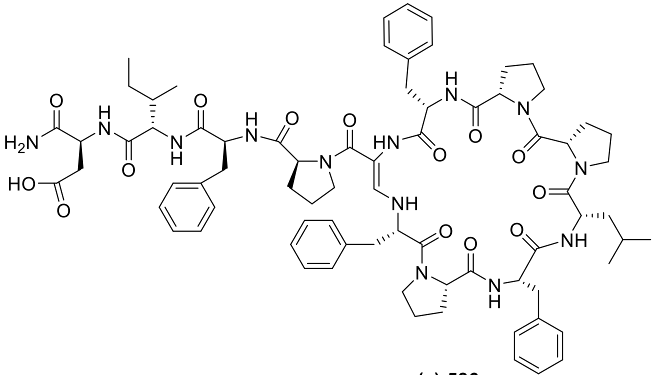

(–)-586

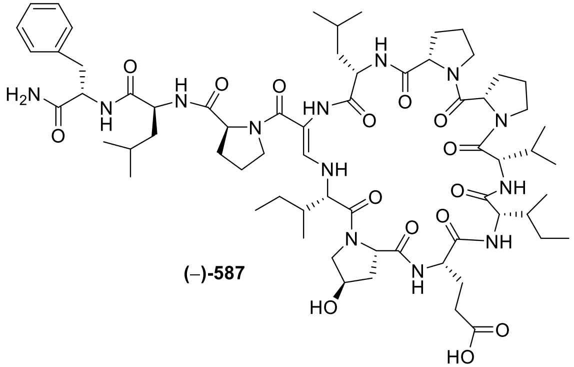

(–)-587

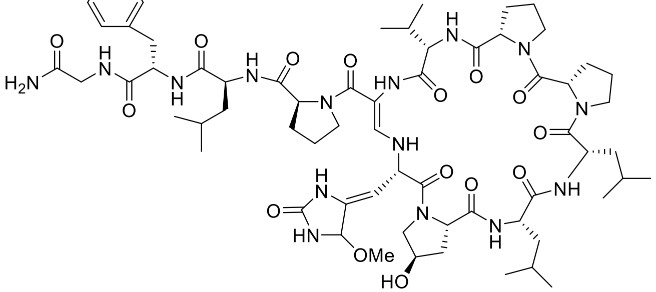

(–)-588

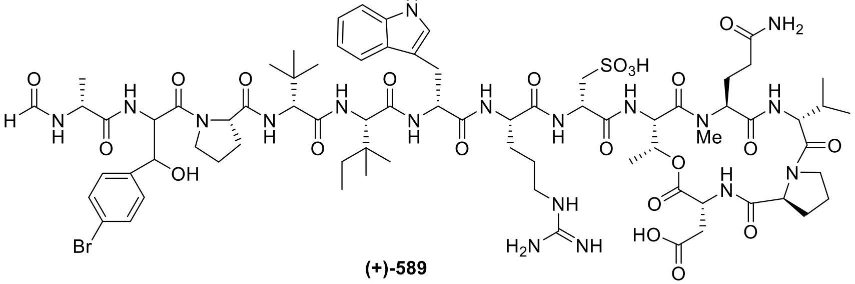

(+)-589

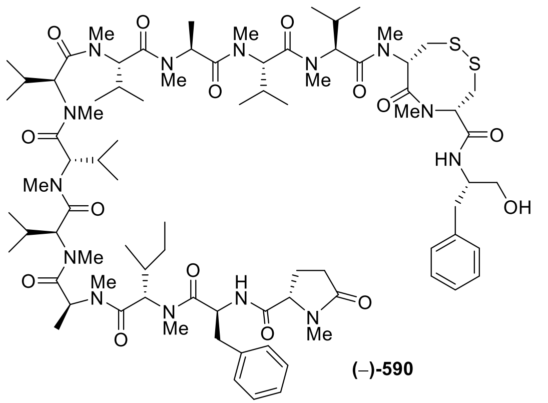

(–)-590

Figure S19: *Cont.*

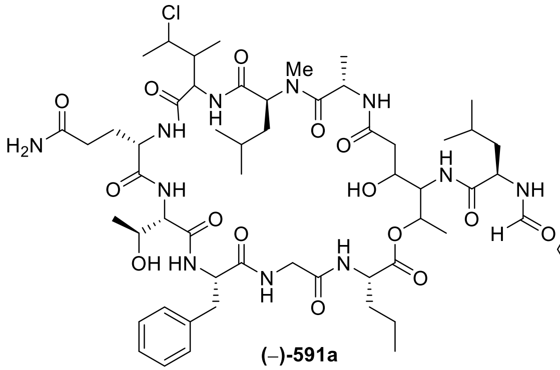

(–)-**591a**

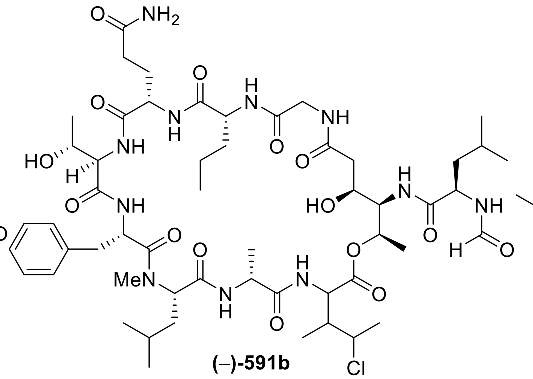

(–)-**591b**

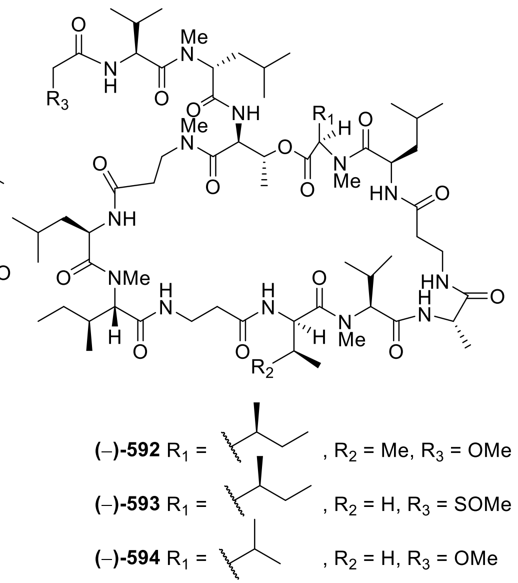

(–)-**592** R_1 = , R_2 = Me, R_3 = OMe

(–)-**593** R_1 = , R_2 = H, R_3 = SOMe

(–)-**594** R_1 = , R_2 = H, R_3 = OMe

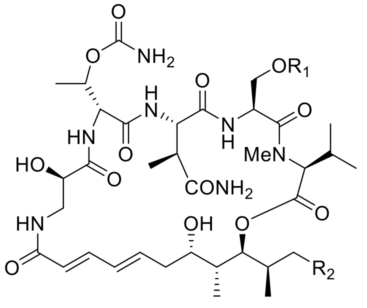

(–)-**595** R_1 = PO_3H_2, R_2 = Et
(–)-**596** R_1 = PO_3H_2, R_2 = Me
(–)-**597** R_1 = H, R_2 = Et

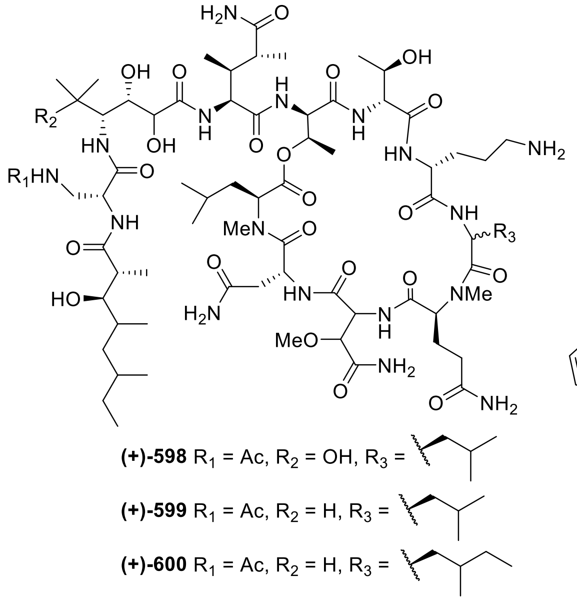

(+)-**598** R_1 = Ac, R_2 = OH, R_3 =

(+)-**599** R_1 = Ac, R_2 = H, R_3 =

(+)-**600** R_1 = Ac, R_2 = H, R_3 =

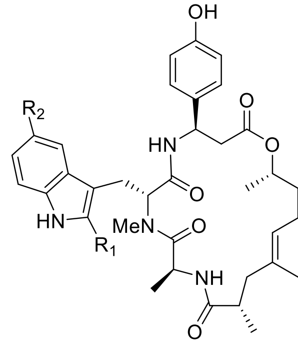

(–)-**601** R_1 = R_2 = H
(–)-**602** R_1 = R_2 = Br

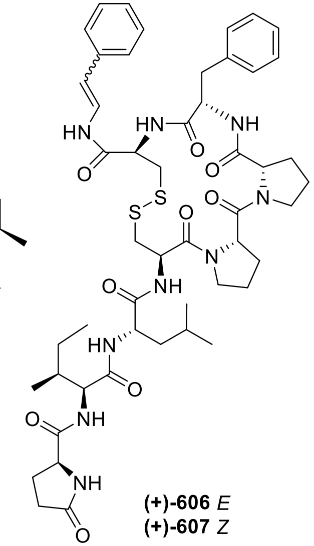

(+)-**606** *E*
(+)-**607** *Z*

Figure S19: *Cont.*

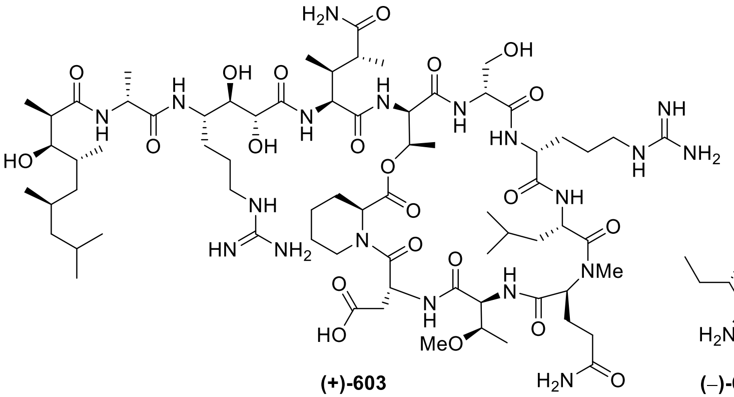

(+)-603

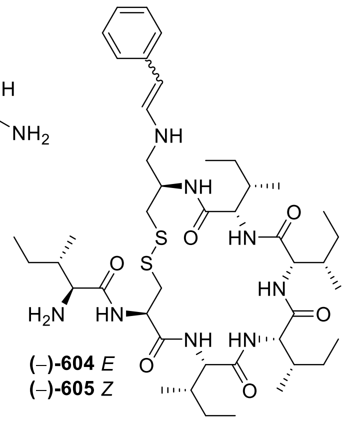

(–)-604 *E*
(–)-605 *Z*

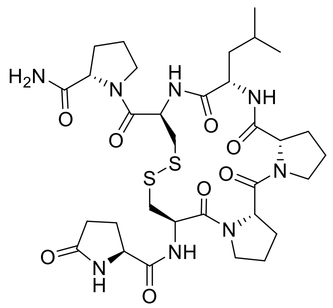

(–)-608

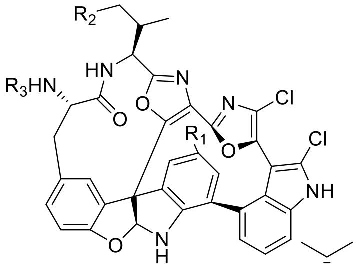

(–)-609 $R_1 = R_2 = H$, $R_3 =$ 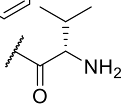

(–)-610 $R_1 = Cl$, $R_2 = Me$, $R_3 = NH_2$

(–)-611 $R_1 = H$, $R_2 = Me$, $R_3 = NH_2$

Figure S19: Structures of marine peptides from Indonesian waters found in 1970–2017.

Table S19: Marine peptides from Indonesian waters found in 1970–2017.

Compound	Structure Elucidation	Chemistry Type	Drug Class	Biological Activity		Source of Organism	Province	Ref
				Cell/Enzyme/Micro-organism/Insect/Others	Activity			
(−)-Dysithiazolamide **552**[β] [$C_{18}H_{27}Cl_4N_3O_2S$]	UV, IR, MS, NMR, QCC, TS	Linear Dipeptide	Undetm.	Undetm.	Undetm.	*Dysidea* sp.	NMU	[358, 359
(+)-Sintokamide A **553**[β] [$C_{18}H_{25}Cl_5N_2O_4$]	UV, MS, NMR, [α]D, X-ray, TS	Linear Dipeptide[♠]	Anticancer	AR NTD in LNCaP<br>AR-positive<br>Cytotoxic	5 μg/mL<br>Active<br>NA (10 μg/mL)	*Dysidea* sp.	CJV	[360 – 362]
(+)-Sintokamide B **554**[β] [$C_{18}H_{24}Cl_6N_2O_4$]	UV, MS, NMR, [α]D , TS	Linear Dipeptide	Undetm.	Undetm.	Undetm.	*Dysidea* sp.	CJV	[360, 361]
(+)-Sintokamide C **555**[β] [$C_{18}H_{26}Cl_4N_2O_4$]	UV, MS, NMR, [α]D, TS	Linear Dipeptide	Anticancer	Androgen receptor-expressing LNCaP	IC_{50} = 35 μM	*Dysidea* sp.	CJV	[360, 363]
(+)-Sintokamide D **556**[β] [$C_{18}H_{26}Cl_4N_2O_4$]	UV, MS, NMR, [α]D	Linear Dipeptide	Anticancer	Undetm.	Undetm.	*Dysidea* sp.	CJV	[360]
(+)-Sintokamide E **557**[β] [$C_{18}H_{27}Cl_3N_2O_4$]	UV, MS, NMR, [α]D, TS	Linear Dipeptide	Undetm.	Undetm.	Undetm.	*Dysidea* sp.	CJV	[360, 361]
(+)-Keenamide **558**[β] [$C_{30}H_{48}N_6O_6S$]	UV, MS, NMR, [α]D	Cyclo hexapeptide	Cytotoxic	P-388, A549, MEL-28<br>HT-29	IC_{50} = 2.5 μg/mL<br>IC_{50} = 5.0 μg/mL	*P. forskalii*	NSW	[364, 365]
			Antiparasite	*P. falciparum* (D6 clone), (W2 clone)	NA			
			Antibacterial	MRSA, *M. intracellulaire*	NA			
			Antifungal	*C. albicans*, *C. glabrata*, *C. krusei*, *C. neofromans*	NA			
			Anti-inflammatory	COX-2 (rat neonatal microglia)	NA			
(−)-Mollamide B **559**[β] [$C_{36}H_{52}N_6O_6S$]	UV, MS, NMR, [α]D, Mol. Mod.	Cyclo hexapeptide	Antibacterial	MRSA, *M. intracellulaire*	NA	*D. molle*	NSW	[364, 365]
			Antifungal	*C. albicans*, *C. glabrata*, *C. krusei*, *C. neofromans*	NA			
			Anti-inflammatory	COX-2 (rat neonatal microglia)	NA			
			Antiparasite	*P. falciparum* (D6 clone), (W2 clone)<br>*L. donovani*	IC_{50} = 2.0 – 2.1 μg/mL<br>IC_{50} = 18 – 35 μg/mL			
			Antiviral	HIV-1 (human PBM cells)	EC_{50} = 48.7 μM			
			Cytotoxic	H460<br>MCF7, SF268	29% (100 μM)<br>42-44% (100 μM)			

Table S19: *Cont.*

Compound	Structure Elucidation	Chemistry Type	Drug Class	Biological Activity: Cell/Enzyme/Micro-organism/Insect/Others	Biological Activity: Activity	Source of Organism	Province	Ref
Mollamide C **560**$^{\beta}$ [$C_{33}H_{46}N_6O_6S$]	UV, MS, NMR, $[\alpha]_D$, CT	Cyclo hexapeptide	Anti-inflammatory	COX-2 (rat neonatal microglia)	NA	*D. molle*	NSW	[365]
			Cytotoxic	L1210<br>CCRF-CEM, C38<br>HCT116<br>H125, MCF7, LNCap	100 z.u. (CFU-GM)<br>NA<br>250 z.u. (CFU-GM)<br>NA			
(−)-Waiakeamide **561**$^{\beta}$ [$C_{37}H_{49}N_7O_8S_3$]	UV, MS, NMR, $[\alpha]_D$, CT	Cyclo hexapeptide♠	Cytotoxic	P-388	NA	*I. dendroides*	NSW	[366]
(−)-Bistramide M **562**$^{\beta}$ [$C_{21}H_{24}N_6O_4S_2$]	UV, MS, NMR, $[\alpha]_D$, CT	Cyclo hexapeptide	Cytotoxic	MDA-MB-231, HT-29, A549	GI_{50} = 9.1 – 18 μM<br>TGI > 20.0 μM<br>LC_{50} > 20.0 μM	*L. bistratum*	SRWP	[367]
(−)-Bistramide N **563**$^{\beta}$ [$C_{21}H_{24}N_6O_4S_2$]	UV, MS, NMR, $[\alpha]_D$, CT	Cyclo hexapeptide	Cytotoxic	MDA-MB-231	GI_{50} > 20.0 μM<br>TGI > 20.0 μM<br>LC_{50} > 20.0 μM	*L. bistratum*	SRWP	[367]
				HT-29, A549, PSN-1	GI_{50} = 11.0 – 15.0 μM<br>TGI > 20.0 μM<br>LC_{50} > 20.0 μM			
(−)-Cupolamide A **564**$^{\beta}$ [$C_{42}H_{66}N_{11}NaO_{14}S$]	UV, MS, NMR, $[\alpha]_D$, CT	Cyclo heptapeptide♠	Cytotoxic	P-388	IC_{50} = 7.5 μg/mL	*T. cupola*	NSW	[368]
(−)-*cis*, *cis*-Ceratospongamide **565**$^{\beta}$ [$C_{41}H_{49}N_7O_6S$]	UV, MS, NMR, $[\alpha]_D$, Mol. Mod., CT, X-ray, TS	Cyclo heptapeptide	Anti-inflammatory	Phospholipase A_2 (HEPG2/ IL-1β)	NA	*C. spongiosum*	NSW	[369 – 373]
			Cytotoxic	*A. salina*	LD_{50} = 13–19 μM (*ca*)			
(−)-*trans*, *trans*-Ceratospongamide **566a**$^{\beta}$ [$C_{41}H_{49}N_7O_6S$]	UV, MS, NMR, $[\alpha]_D$, Mol. Mod., CT, TS	Cyclo heptapeptide	Anti-inflammatory	Phospholipase A_2 (HEPG2/ IL-1β)<br>Phospholipase A_2 (HEPG2/ IL-1β)	ED_{50} = 32 nM<br>50% red. (reporter)<br>90% red. (plasmid)	*C. spongiosum*	NSW	[369 – 373]
			Cytotoxic	*A. salina*	LD_{50} = 13 – 19 μM (*ca*)			
(−)-*trans*, *trans*-[D-*allo*-ile] Ceratospongamide **566b**$^{\gamma}$ [$C_{41}H_{49}N_7O_6S$]	UV, MS, NMR (^{1}H, GCOSY, TOCSY, (ROESY), $[\alpha]_D$, Mol. Mod., X-ray, TS	Cyclo heptapeptide	Anti-inflammatory	Undetm.	Undetm.			[373]
Lissoclinamide 9 **567**$^{\beta}$ [$C_{35}H_{45}N_7O_5S_2$]	UV, IR, MS, NMR, ECD, Mol. Mod.	Cyclo heptapeptide	Metal binding selectivity	Selective (Cu^{2+})	Less selectivity	*L. patella*	NMU	[374, 375]

Table S19: *Cont.*

Compound	Structure Elucidation	Chemistry Type	Drug Class	Biological Activity: Cell/Enzyme/Micro-organism/Insect/Others	Biological Activity: Activity	Source of Organism	Province	Ref
Lissoclinamide 10 **568**[β] [$C_{36}H_{49}N_7O_5S_2$]	UV, IR, MS, NMR, ECD, Mol. Mod.	Cyclo heptapeptide	Metal binding selectivity	Selective (Cu^{2+}, excess Zn^{2+})	High selectivity	*L. patella*	NMU	[374, 375]
(−)-Carteritin A **569**[β] [$C_{44}H_{57}N_7O_{10}$]	UV, IR, MS, NMR, $[\alpha]_D$, CT	Cyclo heptapeptide	Cytotoxic	HeLa, HCT116, RAW264	IC_{50} = 0.70 – 1.50 μM	*S. carteri*	NSW	[376]
(−)-Carteritin B **570**[β] [$C_{46}H_{57}N_7O_{11}$]	UV, IR, MS, NMR, $[\alpha]_D$, CT	Cyclo heptapeptide	Cytotoxic	HeLa, HCT116, RAW264	IC_{50} > 50 μM	*S. carteri*	NSW	[376]
(−)-Stylissamide X **571**[β] [$C_{51}H_{69}N_9O_9$]	UV, IR, MS, NMR, $[\alpha]_D$, CT	Cyclo octapeptide	Anticancer	HeLa (migration)<br>HeLa (via.)<br>HeLa-EGF induced	0.1-10 μM (wound-healing)<br>75% (10 μM) (chemotaxicell chamber)<br>0.1 – 10 μM	*Stylissa* sp.	PUA	[377]
(−)-Barangamide A **572a**[β] [$C_{54}H_{97}N_{11}O_{12}$]	UV, IR, MS, NMR, $[\alpha]_D$, CT	Cyclo undecapeptide	Cytotoxic	L1210	NA (10 μg/mL)	*T. swinhoei*	SSW	[378]
(+)-Barangamide A **572b**[γ] [$C_{54}H_{97}N_{11}O_{12}$]	UV, IR, MS, NMR, $[\alpha]_D$, CT	Cyclo undecapeptide	Cytotoxic	MLR	NA (100 μg/mL)	*T. swinhoei*	SSW	[379]
(+)-Barangamide B **573**[β] [$C_{53}H_{95}N_{11}O_{12}$]	UV, IR, MS, NMR, $[\alpha]_D$, CT	Cyclo undecapeptide	Undetm.	Undetm.	Undetm.	*T. swinhoei*	SSW	[379]
(+)-Barangamide C **574**[β] [$C_{53}H_{95}N_{11}O_{12}$]	UV, IR, MS, NMR, $[\alpha]_D$, CT	Cyclo undecapeptide	Undetm.	Undetm.	Undetm.	*T. swinhoei*	SSW	[379]
(+)-Barangamide D **575**[β] [$C_{53}H_{95}N_{11}O_{12}$]	UV, IR, MS, NMR, $[\alpha]_D$, CT	Cyclo undecapeptide	Undetm.	Undetm.	Undetm.	*T. swinhoei*	SSW	[379]
(−)-Callyaerin G **576a**[β] [$C_{69}H_{91}N_{13}O_{12}$]	UV, IR, MS, NMR, $[\alpha]_D$, CT	Cyclohexapep-tide (penta peptide sc.)	Cytotoxic	L5178Y<br>HeLa, PC12	ED_{50} = 0.41 − 0.53 μg/mL<br>ED_{50} = 3.8 − 4.43 μM	*C. aerizusa*	MLU	[380, 381]
(−)-Callyaerin G **576b**[γ] [$C_{69}H_{91}N_{13}O_{12}$]	NMR (COSY, TOCSY, ROESY)	Cyclohexapep-tide (penta peptide sc.)	Cytotoxic<br>Antibacterial	THP-1, MRC-5<br>*M. tubercolosis*	IC_{50} > 10 μM<br>MIC_{90} > 100 μM	*C aerizusa*	SSW	[382]
(−)-Callyaerin A **577**[β] [$C_{70}H_{110}N_{14}O_{14}$]	UV, MS, NMR, $[\alpha]_D$, CT, TS	Cyclooctapep-tide (tetra-peptide sc.)	Cytotoxic<br>Antifungal<br>Antibacterial	L5178Y<br>*C. albicans*<br>*S. aureus, B. subtilis*<br>*E. coli*<br>*M. tubercolosis*	ED_{50} = 3.61 μM<br>25 – 30 mm (5 − 10 μL)<br>0 − 9 mm (5 − 10 μL)<br>10 – 15 mm (5 − 10 μL)<br>MIC_{90} = 2 μM<br>MIC_{100} = 6 μM	*C. aerizusa*	MLU	[381 – 383]

Table S19: *Cont.*

Compound	Structure Elucidation	Chemistry Type	Drug Class	Biological Activity: Cell/Enzyme/Micro-organism/Insect/Others	Biological Activity: Activity	Source of Organism	Province	Ref
(−)-Callyaerin A **577**[β] [$C_{70}H_{110}N_{14}O_{14}$]	UV, MS, NMR, $[\alpha]_D$, CT, TS	Cyclooctapeptide (tetrapeptide sc.)	Cytotoxic	THP-1, MRC-5	IC_{50} = 20 – 50 μM<br>IC_{90} = 40 – 50 μM	*C. aerizusa*	MLU	[381 – 383]
(−)-Callyaerin B **578**[β] [$C_{66}H_{111}N_{13}O_{13}$]	UV, MS, NMR, $[\alpha]_D$, CT	Cyclo octapeptide (tripeptide sc.)	Cytotoxic	*A. salina*	15% (20 μg/mL)<br>35% (50 μg/mL)	*C. aerizusa*	MLU	[381, 382]
				L5178Y	ED_{50} = 4.14 μM			
				HeLa, PC12	ED_{50} > 8 μM			
				THP-1, MRC-5	IC_{50} = 2 – 5 μM<br>IC_{90} = 6 – 30 μM			
			Antifungal	*C. albicans*	15 mm (5 −10 μL)			
			Antibacterial	*S. aureus*	7 – 10 mm (5 – 10 μL)			
				B. subtilis	0 mm (5 – 10 μL)			
				E coli	11 mm (5 – 10 μL)			
				M. tubercolosis	MIC_{90} = 5 μM<br>MIC_{100} = 10 μM			
(−)-Callyaerin C **579**[β] [$C_{64}H_{95}N_{15}O_{13}$]	UV, MS, NMR, $[\alpha]_D$, CT	Cyclohepta peptide (tetrapeptide sc.)	Cytotoxic	L5178Y	ED_{50} = 2.92 μM	*C. aerizusa*	MLU	[381, 382]
				THP-1, MRC-5	IC_{50} > 100 μM,<br>IC_{90} > 100 μM			
			Antifungal	*C. albicans*	0 mm (5 – 10 μL)			
			Antibacterial	*S. aureus*	7 – 10 mm (5 μL)			
				B. subtilis, E. coli	0 mm (5 – 10 μL)			
				M. tubercolosis	MIC_{90} = 40 μM<br>MIC_{100} = 100 μM			
(−)-Callyaerin D **580a**[β] [$C_{70}H_{109}N_{15}O_{15}$]	UV, MS, NMR, $[\alpha]_D$, CT	Cyclohepta-peptide (penta-peptide sc.)	Cytotoxic	L5178Y	ED_{50} = 3.03 μM	*C. aerizusa*	MLU	[382]
				THP-1, MRC-5	IC_{50} > 10 μM			
			Antibacterial	*S. aureus, E. coli*	0 mm (5 – 10 μL)			
				B. subtilis	12 mm (5 – 10 μL)			
				M. tubercolosis	MIC_{90} > 100 μM			
			Antifungal	*C. albicans*	0 – 7 mm (5 – 10 μL)			
(−)-Callyaerin D **580b**[γ] [$C_{70}H_{109}N_{15}O_{15}$]	HMBC, ROESY, ESIMS	Cyclohepta-peptide (pentapeptide sc.)	Antibacterial	*M. tubercolosis*	MIC_{90} > 100 μM	*C. aerizusa*	MLU	[381]

Table S19: *Cont.*

Compound	Structure Elucidation	Chemistry Type	Drug Class	Biological Activity: Cell/Enzyme/Micro-organism/Insect/Others	Biological Activity: Activity	Source of Organism	Province	Ref
(−)-Callyaerin E **581**[β] [$C_{66}H_{95}N_{13}O_{12}$]	UV, MS, NMR, [α]D, CT	Cyclohepta-peptide (tetra peptide sc.)	Cytotoxic	*A. salina*<br>L5178Y<br>HeLa, PC12	45 – 70% (20 – 50 μg/mL)<br>ED_{50} = 0.39 μM<br>ED_{50} = 3.4 – 3.8 μM	*C. aerizusa*	MLU	[382]
			Antibacterial	*S. aureus*<br>*B. subtilis*<br>*E. coli*<br>*M. tubercolosis*	9 – 10 mm (5 – 10 μL)<br>15 –17 mm (5 – 10 μL)<br>9 – 11 mm (5 – 10 μL)<br>MIC_{90} = 100 μM			
			Antifungal	*C. albicans*	20 mm (5 – 10 μL)			
(−)-Callyaerin F **582a**[β] [$C_{59}H_{85}N_{11}O_{10}$]	UV, MS, NMR, [α]D, chemical transformation	Cyclo pentapeptide (tetrapeptide sc.)	Cytotoxic	L5178Y, HeLa, PC12<br>THP-1, MRC-5	ED_{50} > 9 μM<br>IC_{50} > 10 μM	*C. aerizusa*	MLU	[381]
			Antibacterial	*S. aureus, B. subtilis*<br>*E. coli*	0 – 9 mm (5 – 10 μL)<br>0 mm (5 – 10 μL)			
			Antifungal	*C. albicans*	0 mm (5 –10 μL)			
(−)-Callyaerin F **582b**[γ] [$C_{58}H_{83}N_{11}O_{10}$]	MS, NMR (ROESY)	Cyclo pentapeptide (dipeptide sc.)	Antibacterial	*M. tubercolosis*	MIC_{90} = 50 μM	*C. aerizusa*	SSW	[382]
			Cytotoxic	THP-1, MRC-5	IC_{50} > 10 μM			
(−)-Callyaerin H **583**[β] [$C_{54}H_{81}N_{11}O_{10}$]	UV, MS, NMR, [α]D, CT	Cyclo heptapeptide (dipeptide sc.)	Cytotoxic	*A. salina*<br>L5178Y	30 – 55% (20 – 50 μg/mL)<br>ED_{50} = 0.48 μM	*C. aerizusa*	MLU	[381]
(−)-Callyaerin I **584**[β] [$C_{69}H_{93}N_{13}O_{12}$]	UV, MS, NMR, [α]D, CT	Cyclo heptapeptide (tetrapeptide sc.)	Antibacterial	*M. tubercolosis*	MIC_{90} > 100 μM	*C. aerizusa*	MLU	[382]
			Cytotoxic	THP-1, MRC-5	IC_{50} > 10 μM			
(−)-Callyaerin J **585**[β] [$C_{66}H_{95}N_{13}O_{12}$]	UV, MS, NMR, [α]D, CT	Cyclo heptapeptide (tetrapeptide sc.)	Antibacterial	*M. tubercolosis*	MIC_{90} > 100 μM	*C. aerizusa*	MLU	[382]
			Cytotoxic	THP-1, MRC-5	IC_{50} > 10 μM			
(−)-Callyaerin K **586**[β] [$C_{75}H_{95}N_{13}O_{12}$]	UV, MS, NMR, [α]D, CT	Cyclo heptapeptide (tetrapeptide sc.)	Antibacterial	*M. tubercolosis*	MIC_{90} > 100 μM	*C. aerizusa*	MLU	[382]
			Cytotoxic	THP-1, MRC-5	IC_{50} > 10 μM			
(−)-Callyaerin L **587**[β] [$C_{66}H_{101}N_{13}O_{15}$]	UV, MS, NMR, [α]D, CT	Cyclo octapeptide (tripeptide sc.)	Antibacterial	*M. tubercolosis*	MIC_{90} > 100 μM	*C. aerizusa*	MLU	[382]
			Cytotoxic	THP-1, MRC-5	IC_{50} > 10 μM			

Table S19: *Cont.*

Compound	Structure Elucidation	Chemistry Type	Drug Class	Biological Activity: Cell/Enzyme/Micro-organism/Insect/Others	Biological Activity: Activity	Source of Organism	Province	Ref
(−)-Callyaerin M **588**[β] [$C_{64}H_{95}N_{15}O_{15}$]	UV, MS, NMR, [α]D, CT	Cyclo octapeptide (tetrapeptide sc.)	Antibacterial	*M. tubercolosis*	MIC_{90} > 100 μM	*C. aerizusa*	MLU	[382]
			Cytotoxic	THP-1, MRC-5	IC_{50} > 10 μM			
(+)-Microspinosamide **589**[β] [$C_{75}H_{109}BrN_{18}O_{22}S$]	UV, IR, MS, NMR, [α]D, CT	Cyclo depsipeptide (octapeptide sc.)♠	Antiviral	CEM-SS infected HIV-1	EC_{50} = 0.2 μg/mL	*S. microspinosa*	NSW	[384]
			Cytotoxic	CEMS-SS	EC_{50} ~ 3.0 μg/mL			
(−)-Kendarimide **590**[β] [$C_{83}H_{134}N_{14}O_{15}S_2$]	UV, IR, MS, NMR, [α]D, CT	Linear trideca-peptide♠	Cytotoxic	KB-C2, P-gp type	87% (KB-C2, 0.1 μg/mL colchine) (6 μM)	*Haliclona* sp.	SES	[385, 386]
				KB-3-1	NA in KB-3-1 (6 μM)			
(−)-Cyclolithistide A **591a**[β] [$C_{54}H_{86}ClN_{11}O_{15}$]	UV, IR, MS, NMR, [α]D, CT	Cyclo depsipeptide♠	Antifungal	*C. albicans* (ATCC 24433)	90% (20 μg/disk)	*T. swinhoei*	NSW	[387]
			Antibacterial	*E. coli, B. subtilis*	NA			
			Cytotoxic	NCI 60 cell line	NA			
(−)-Cyclolithistide A **591b**[γ] [$C_{54}H_{86}ClN_{11}O_{15}$]	UV, MS, GC-MS, NMR, HMBC, [α]D, CT	Cyclo depsipeptide	Undetm.	Undetm.	Undetm.	*D. japonica*	JPN	[388]
(−)-Theonellapeptolide IIe **592**[β] [$C_{70}H_{125}N_{13}O_{16}$]	IR, MS, NMR, [α]D, CT	Cyclo depsipeptide	Cytotoxic	L1210	NA	*T. swinhoei*	SSW	[379]
(−)-Sulfinyltheonellapeptolide **593**[β] [$C_{69}H_{123}N_{13}O_{16}S$]	MS, NMR, [α]D, CT	Cyclo depsipeptide♦	Cytostatic	HepG2	IC_{50} = 3 μM	*T. swinhoei*	NSW	[389]
(−)-Theonellapeptolide If **594**[β] [$C_{69}H_{121}N_{13}O_{16}$]	MS, NMR, [α]D, CT	Cyclo depsipeptide	Cytostatic	HepG2	IC_{50} = 3 μM	*T. swinhoei*	NSW	[389]
(−)-Celebeside A **595**[β] [$C_{37}H_{62}N_7O_{16}P$]	UV, IR, MS, NMR, [α]D, Mol. Mod., CT	Cyclo depsipeptide♠	Antiviral	HIV-1 SF162 envelope	IC_{50} = 1.9 ± 0.4 μg/mL	*S. mirabilis*	UEP	[390]
			Cytotoxic	HCT-116	IC_{50} = 8.8 ± 3.0 μg/mL			
			Antibacterial	*B. subtilis, E coli, P. aeruginosa, S. aureus*	NA (50 μg/disk)			
			Antifungal	*C. albicans*	NA (50 μg/disk)			
(−)-Celebeside B **596**[β] [$C_{36}H_{60}N_7O_{16}P$]	UV, IR, MS, NMR, [α]D, Mol. Mod., CT	Cyclo depsipeptide	Antibacterial	*B. subtilis, E coli, P. aeruginosa, S. aureus*	NA (50 μg/disk)	*S. mirabilis*	UEP	[390]
			Antifungal	*C. albicans*	NA (50 μg/disk)			

Table S19: *Cont.*

Compound	Structure Elucidation	Chemistry Type	Drug Class	Biological Activity: Cell/Enzyme/Micro-organism/Insect/Others	Biological Activity: Activity	Source of Organism	Province	Ref
(−)-Celebeside C **597**$^{\beta}$ [$C_{37}H_{61}N_7O_{13}$]	UV, IR, MS, NMR, [α]D, Mol. Mod., CT	Cyclo depsipeptide	Antiviral	HIV-1 SF162 envelope	IC_{50} > 50 μg/mL	*S. mirabilis*	UEP	[390]
			Cytotoxic	HCT-116	IC_{50} > 25 μg/mL			
			Antibacterial	*B. subtilis, E coli, P. aeruginosa, S. aureus*	NA (50 μg/disk)			
			Antifungal	*C. albicans*	NA (50 μg/disk)			
(+)-Theopapuamide B **598**$^{\beta}$ [$C_{71}H_{125}N_{17}O_{24}$]	UV, IR, MS, NMR, [α]D, CT	Cyclo depsipeptide	Antiviral	HIV-1 SF162 envelope	IC_{50} = 0.8 ± 0.3 μg/mL	*S. mirabilis*	UEP	[390]
			Cytotoxic	HCT-116	IC_{50} = 2.1 ± 0.7 μg/mL			
			Antifungal	*C. albicans, C. albicans* (amphotericin B-resistant)	10 mm (5 μg/disk)			
(+)-Theopapuamide C **599**$^{\beta}$ [$C_{71}H_{125}N_{17}O_{23}$]	UV, IR, MS, NMR, [α]D, CT	Cyclo depsipeptide	Cytotoxic	HCT-116	IC_{50} = 4.0 ± 1.7 μg/mL	*S. mirabilis*	UEP	[390]
			Antifungal	*C. albicans, C. albicans* (amphotericin B-resistant)	10 mm (5 μg/disk)			
(+)-Theopapuamide D **600**$^{\beta}$ [$C_{72}H_{127}N_{17}O_{23}$]	UV, IR, MS, NMR, [α]D, CT	Cyclo depsipeptide	Cytotoxic	HCT-116	IC_{50} = 2.1 ± 0.9 μg/mL	*S. mirabilis*	UEP	[390]
(−)-Jaspamide Q **601**$^{\beta}$ [$C_{36}H_{46}N_4O_6$]	UV, MS, NMR, [α]D	Cyclo depsipeptide	Cytotoxic	L5178Y	IC_{50} < 0.1 μg/mL	*J. splendens*	EKM	[391]
(−)-Jaspamide R **602**$^{\beta}$ [$C_{36}H_{44}Br_2N_4O_6$]	UV, MS, NMR, [α]D	Cyclo depsipeptide	Cytotoxic	L5178Y	IC_{50} < 0.1 μg/mL	*J. splendens*	EKM	[391]
(+)-Daedophamide **603**$^{\beta}$ [$C_{71}H_{125}N_{19}O_{22}$]	UV, IR, MS, NMR, [α]D	Cyclo depsipeptide	Cytotoxic	MDA-MB-231, HT-29, A549, PSN-1	GI_{50} = 0.2 − 0.6 μM<br>TGI = 0.3 − 0.8 μM<br>LC_{50} = 0.6 − 1.3 μM	*Daedalopelta* sp.	ENT	[392]
(−)-Microcionamide C **604**$^{\beta}$ [$C_{44}H_{74}N_8O_6S_2$]	UV, MS, NMR, [α]D	Cyclo pentapeptide$^{\spadesuit}$	Cytotoxic	A2780, Jurkat, Ramos, Nomo-1, HL-60	IC_{50} = 0.45 – 1.90 μM	*C. basilana*	MLU	[357]
				Apop. (caspase activation)	1 μM			
				Starvation-ind. autophagy	10 μM			
			Antibacterial	*E. faecium* BM4147-1	MIC = 12.5 μM			
				S. aureus ATCC29213	MIC = 6.3 μM			
				M. tubercolosis H37Rv, *K. pneumoniae* ATCC 12657, *E. aerogenes* ATCC 13048, *E. coli* ATCC 25922, *P. aeruginosa* ATCC 27853, *A. baumannii* 09987	MIC > 100			

Table S19: *Cont.*

Compound	Structure Elucidation	Chemistry Type	Drug Class	Biological Activity: Cell/Enzyme/Micro-organism/Insect/Others	Biological Activity: Activity	Source of Organism	Province	Ref
(−)-Microcionamide D **605**[β] [$C_{44}H_{74}N_8O_6S_2$]	UV, MS, NMR, $[\alpha]_D$	Cyclo pentapeptide	Cytotoxic	A2780, Ramos, Jurkat, Nomo-1, HL-60<br>Apop. (caspase activation)<br>starvation-ind. autophagy	IC_{50} = 0.53 – 2.50 μM<br>1 μM<br>10 μM	*C. basilana*	MLU	[357]
(+)-Gombamide B **606**[β] [$C_{50}H_{67}N_9O_9S_2$]	UV, MS, NMR, $[\alpha]_D$	Cyclo tetrapeptide (tripeptide sc)	Cytotoxic<br>Antibacterial	A2780, Ramos, Jurkat, Nomo-1, HL-60<br>*E. faecium* BM4147-1, *S. aureus* ATCC29213<br>*M. tubercolosis* H37Rv, *K. pneumoniae* ATCC 12657, *E. aerogenes* ATCC 13048, *A. baumannii* 09987, *P. aeruginosa* ATCC 27853	NA<br>MIC > 50<br>MIC > 100	*C. basilana*	MLU	[357]
(+)-Gombamide C **607**[β] [$C_{50}H_{67}N_9O_9S_2$]	UV, MS, NMR, $[\alpha]_D$	Cyclo tetrapeptide (tripeptide sc)	Cytotoxic<br>Antibacterial	Ramos, Jurkat, Nomo-1, HL-60<br>*E. faecium* BM4147-1, *S. aureus* ATCC29213<br>*M. tubercolosis* H37Rv, *K. pneumoniae* ATCC 12657, *E. aerogenes* ATCC 13048, *A. baumannii* 09987, *P. aeruginosa* ATCC 27853	NA<br>MIC > 50<br>MIC > 100	*C. basilana*	MLU	[357]
(−)-Gombamide D **608**[β] [$C_{32}H_{48}N_8O_8S_2$]	UV, MS, NMR, $[\alpha]_D$	Cyclo tetrapeptide	Cytotoxic<br>Antibacterial	Ramos, Jurkat, Nomo-1, HL-60<br>*E. faecium* BM4147-1, *S. aureus* ATCC29213<br>*K. pneumoniae* ATCC 12657, *E. aerogenes* ATCC 13048, *P. aueruginosa* ATCC 13048	NA<br>MIC > 50<br>MIC > 100	*C. basilana*	MLU	[357]
(−)-Diazonamide C **609**[β] [$C_{40}H_{35}Cl_2N_7O_5$]	MS, NMR, $[\alpha]_D$	Macrocyclic peptide	Cytotoxic	A549, MDA-MB-231, HT-29	GI_{50} = 1.8 – 2.2 μM	*Diazona* sp.	PUA	[393]
(−)-Diazonamide D **610**[β] [$C_{36}H_{27}Cl_3N_6O_4$]	MS, NMR, $[\alpha]_D$	Macrocyclic peptide	Cytotoxic	A549, HT-29, MDA-MB-231	GI_{50} = 2.9 – 3.1 μM	*Diazona* sp.	PUA	[393]

Table S19: *Cont.*

Compound	Structure Elucidation	Chemistry Type	Drug Class	Biological Activity		Source of Organism	Province	Ref
				Cell/Enzyme/Micro-organism/Insect/Others	Activity			
(−)-Diazonamide E **611**[β] [$C_{36}H_{27}BrCl_2N_6O_4$]	MS, NMR, $[\alpha]_D$	Macrocyclic peptide	Cytotoxic	A549, HT-29, MDA-MB-231	GI_{50} = 1.8 − 2.2 μM	*Diazona* sp.	PUA	[393]

Footnote: **1. Statisctic** (**TGI** total growth inhibition); **2. Activity (HepG2** human hepatocarcinoma**, LNCaP** in human prostate carcinoma, **MRC-5** human fetal lung fibroblast, **Nomo-1** human adult monocyclic leukemia, **Ramos** human Burkitt lymphoma, **RAW 264** murine macrophage, **AR NTD** androgen receptor N-terminus domain, **HIV-1** human immunodeficiency virus 1.

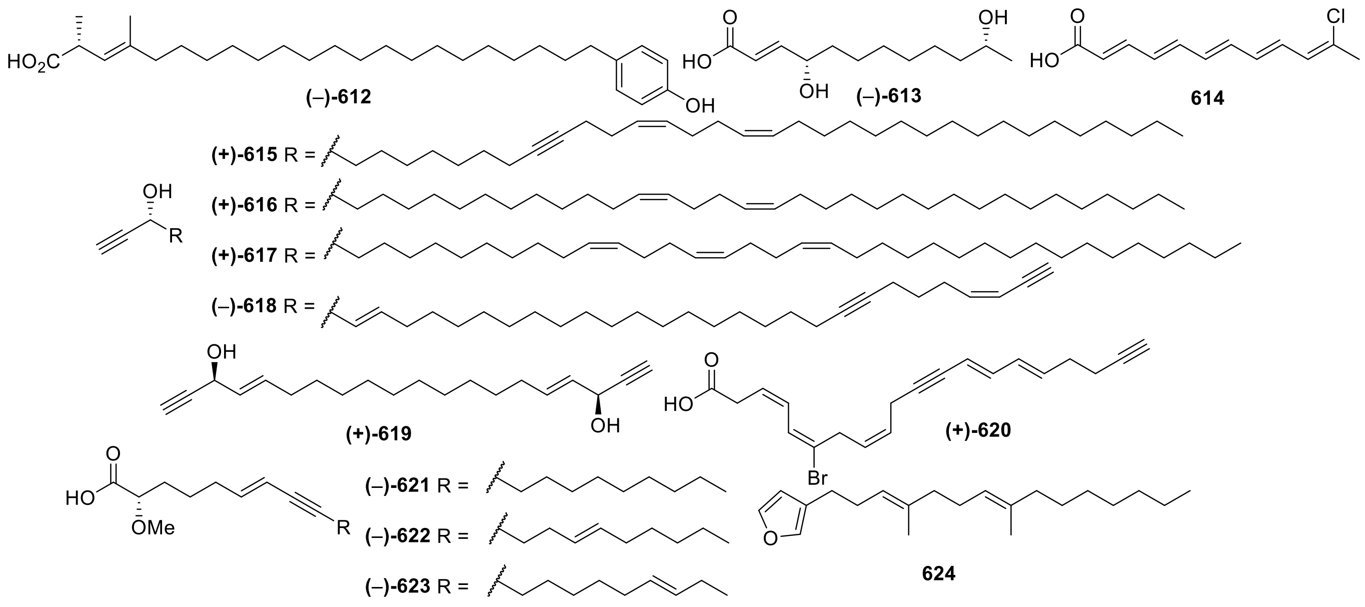

Figure S20: Structures of marine fatty acids and linear molecules from Indonesian waters found in 1970–2017.

Table S20: Marine fatty acids and linear molecules from Indonesian waters found in 1970–2017.

Compound	Structure Elucidation	Chemistry Type	Drug Class	Biological Activity		Source of Organism	Province	Ref
				Cell/Enzyme/Micro-organism/Insect/Others	Activity			
(−)-Elenic acid **612**[β] [$C_{30}H_{50}O_3$]	UV, IR MS, NMR, $[\alpha]_D$, CT, TS	Fatty acid♠	Cytotoxic	P-388, A549, MEL-28	IC_{50} = 5 μg/mL	*Plakinastrella* sp.	NSW	[394 – 396]
				Topoisomerase II	0.1 μg/mL			
(−)-*seco*-Patulolide C **613**[β] [$C_{12}H_{22}O_4$]	UV, MS, NMR, $[\alpha]_D$, CT, TS	Fatty acid	Cytotoxic	A549, HeLa, SMMC-7721	IC_{50} > 50 μM	A fungus (symbiont) a sponge (host)	SSW	[397 – 399]
			Antihyperlipidemic	HepG2	IC_{50} = 13.1 μM			
			Antibacterial	Gram-positive, Gram-negative bacteria	NA (250 μg/disk)			
Aurantoic acid **614**[β] [$C_{12}H_{13}ClO_2$]	UV, MS, NMR	Fatty acid	Cytotoxic	C6, HeLa, H9c2	IC_{50} > 70 μM	*T. swinhoei*	NSW	[212]
(+)-Lembehyne A **615**[β] [$C_{36}H_{62}O$]	IR, MS, NMR, $[\alpha]_D$, CT, TS	Poly acetylenene	Neuro disease	Neurite outgrowth (PC12)	2 μg/mL	*Haliclona* sp.	NSW	[400 – 403]
				Neuritogenesis (Neuro2A)	*ca.* 60% inducer (3.0 μg/mL)			
				Enhancer cyclic dependent kinase inhibitor (p21/WAF1)	G1 arres.			
(+)-Lembehyne B **616**[β] [$C_{36}H_{66}O$]	IR, MS, NMR, $[\alpha]_D$, CT, TS	Poly acetylenene	Neuro disease	Neuritogenesis (Neuro2A)	*ca.* 60% inducer (3.0 μg/mL)	*Haliclona* sp.	NSW	[401 – 407]
			Cytotoxic	Jurkat	IC_{50} = 2.0 μM, 72.6% apop. (2.0 μM)			
				HL-60	IC_{50} = 2.2 μM			
				K562	IC_{50} = 3.0 μM			
(+)-Lembehyne C **617**[β] [$C_{36}H_{66}O$]	IR, MS, NMR, $[\alpha]_D$, CT, TS	Poly acetylenene	Neuro disease	Neuritogenesis (Neuro2A)	*ca.* 60% inducer (3.0 μg/mL)	*Haliclona* sp.	NSW	[404]
(−)-**618**[β] [$C_{31}H_{48}O$]	IR, MS, NMR, $[\alpha]_D$, CT	Poly acetylenene	Cytotoxic	NBT-T2	5 – 10 μg/mL	*Callyspongia* sp.	ENT	[408]
(+)-(3*S*,18*S*,4*E*,16*E*)-eicosa-1,19-diyne-3,18-diol-4,16-diene **619**[β] [$C_{20}H_{30}O_2$]	IR, MS, NMR, $[\alpha]_D$, CT	Poly acetylenene	Cytotoxic	*A. salina*	LD_{50} = 2.0 μg/mL	*C. pseudo-reticulata*	SSW	[409]
**620**[β] [$C_{20}H_{21}BrO_2$]	IR, MS, NMR, $[\alpha]_D$	Poly acetylenene♠	Cytotoxic	NBT-T2	IC_{50} = 36 μg/mL	*Haliclona* sp.	ENT	[410]

Table S20: *Cont.*

Compound	Structure Elucidation	Chemistry Type	Drug Class	Biological Activity: Cell/Enzyme/Micro-organism/Insect/Others	Biological Activity: Activity	Source of Organism	Province	Ref
(−)-Cinachylenic acid B **621**[β] [$C_{19}H_{32}O_3$]	UV, IR, MS, NMR, $[\alpha]_D$	Poly acetylenene	Cytotoxic	L5178Y	IC_{50} = 0.3 μM	*Cinachyrella* sp.	MLU	[355]
(−)-Cinachylenic acid C **622**[β] [$C_{19}H_{30}O_3$]	UV, IR, MS, NMR, $[\alpha]_D$	Poly acetylenene	Cytotoxic	L5178Y	IC_{50} = 0.3 μM	*Cinachyrella* sp.	MLU	[355]
(−)-Cinachylenic acid D **623**[β] [$C_{19}H_{30}O_3$]	UV, IR, MS, NMR, $[\alpha]_D$	Poly acetylenene	Cytotoxic	L5178Y	IC_{50} = 0.3 μM	*Cinachyrella* sp.	MLU	[355]
3-[(3*E*,7*E*)-4,8-dimethylpentadeca-3,7dienyl] furan **624**[β] [$C_{21}H_{34}O$]	UV, IR, MS, NMR	Furanolipid	Antitumor	HIF-1 (T47D)	NA (10 μM)	*Lendenfeldia* sp.	UEP	[411]

Footnote: : **1. Activity (HIF-1** hypoxia-inducible factor-1).

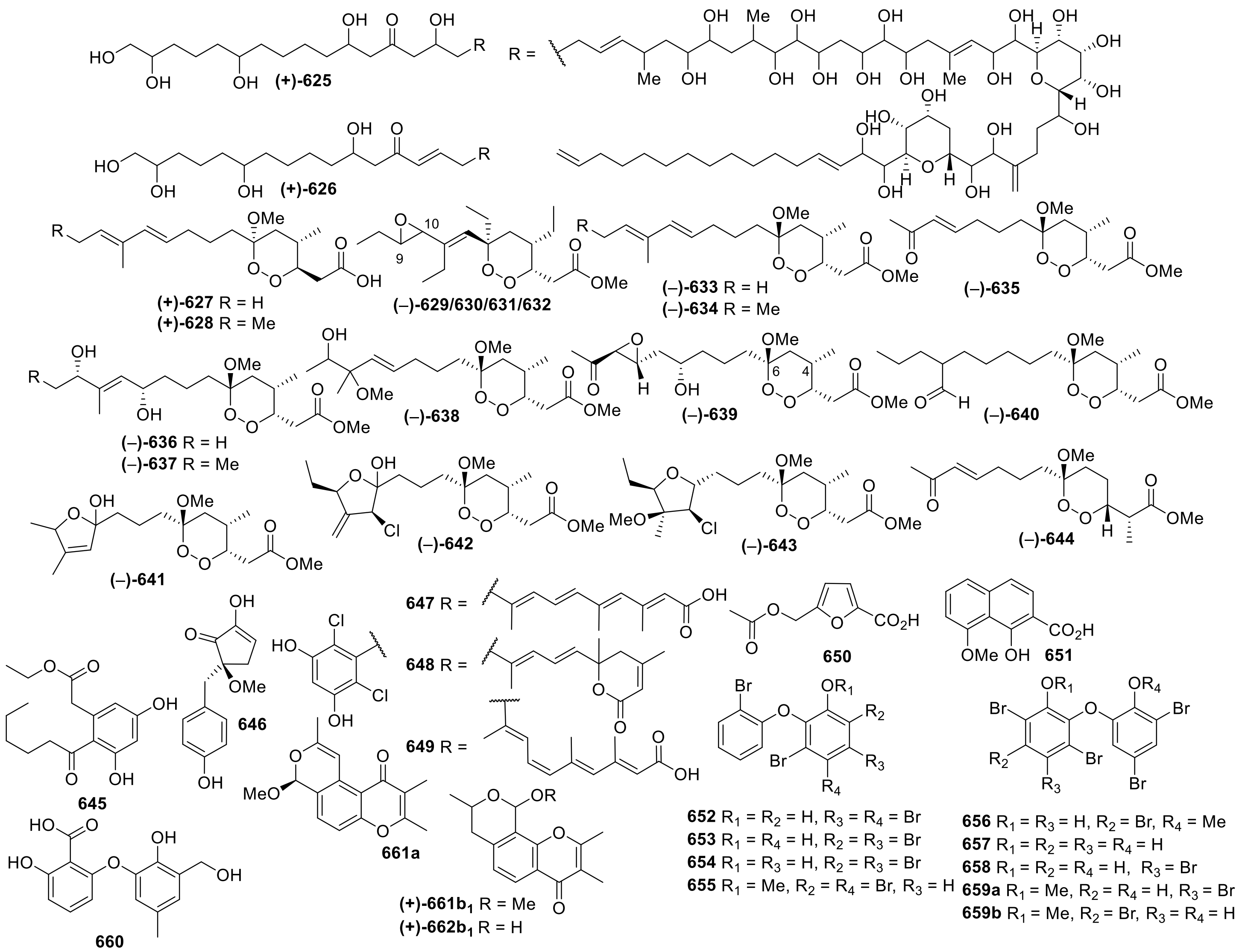

Figure S21: *Cont.*

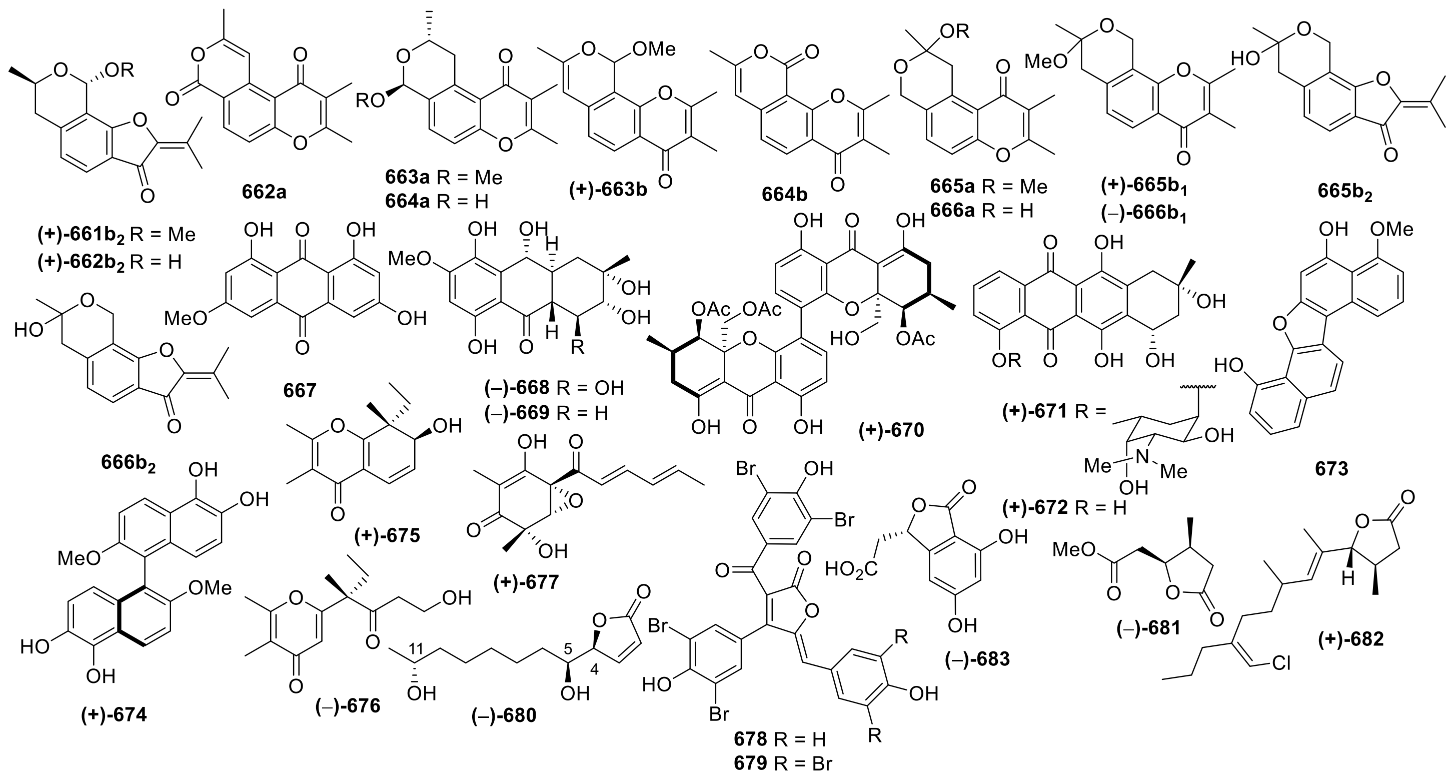

Figure S21: *Cont.*

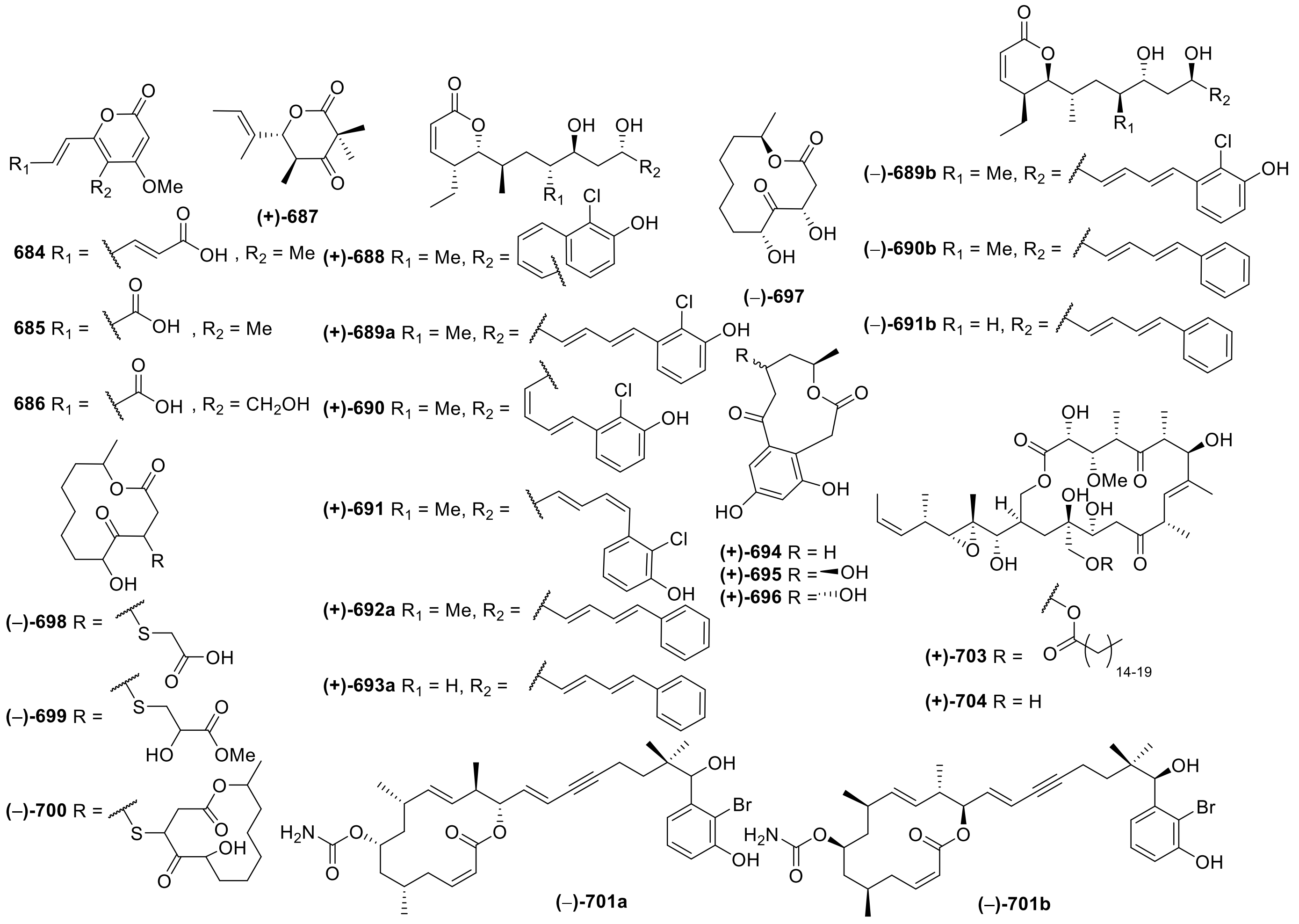

Figure S21: *Cont.*

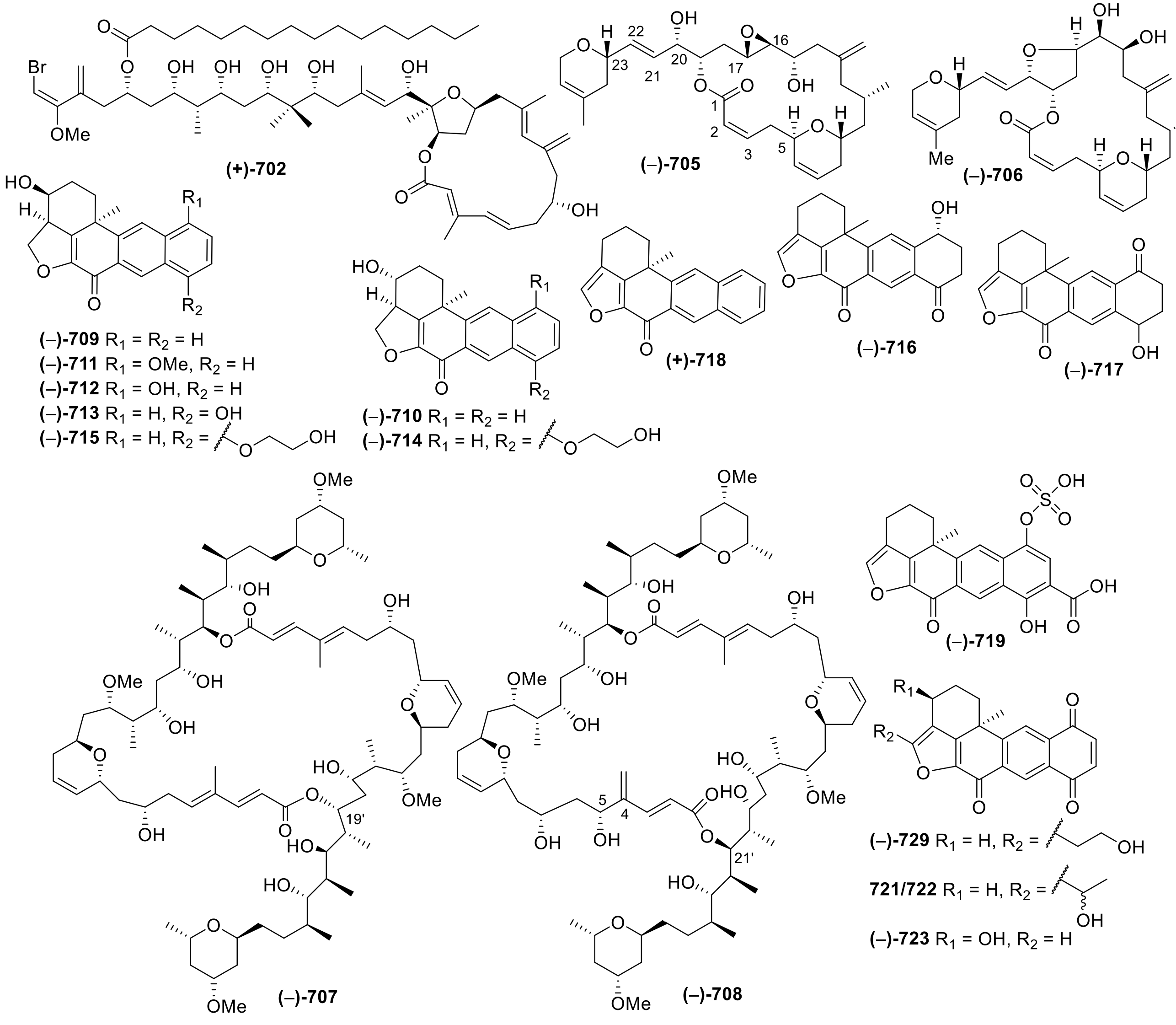

Figure S21: *Cont.*

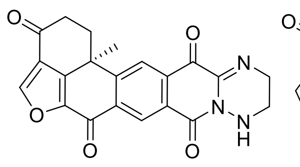

(+)-724

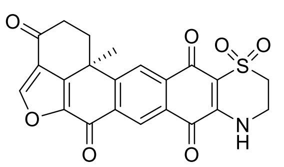

(+)-725

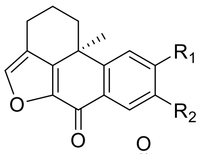

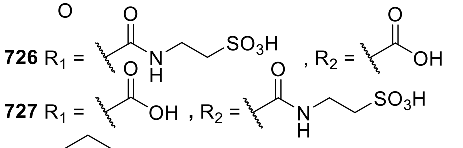

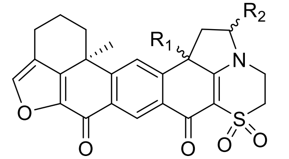

(–)-728 R_1 = OH, R_2 = OH
(+)-729 R_1 = OH, R_2 = OH

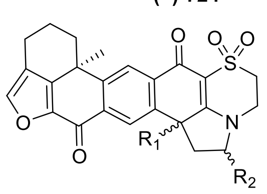

(+)-730 R_1 = OH, R_2 = OH
(+)-731 R_1 = OH, R_2 = OH
732/733 R_1 = R_2 = OH

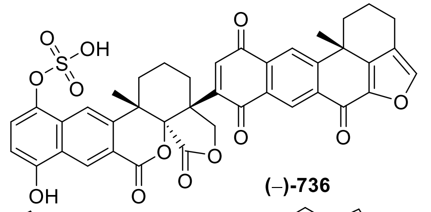

(–)-**736**

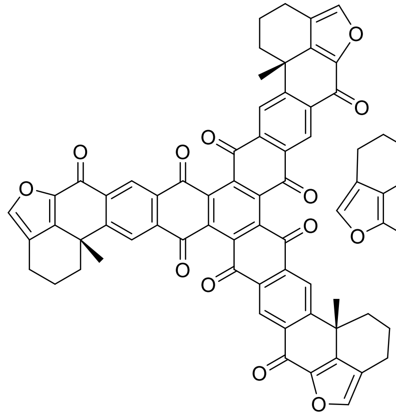

(+)-734

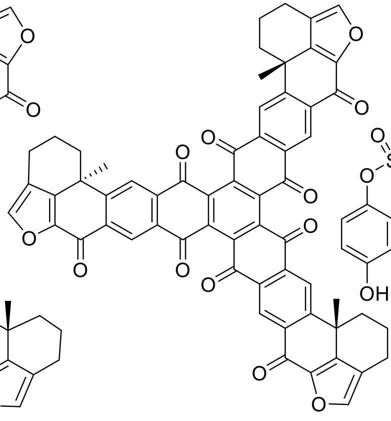

(–)-**735**

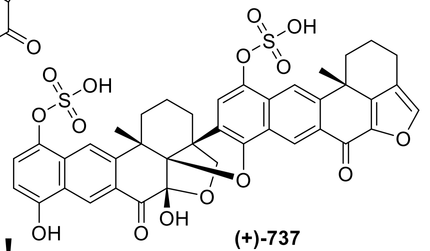

(+)-737

Figure S21: *Cont.*

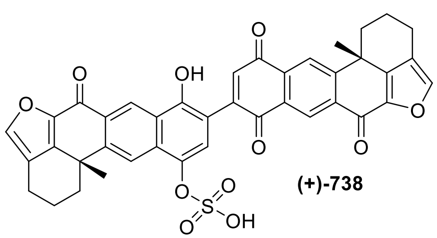

(+)-738

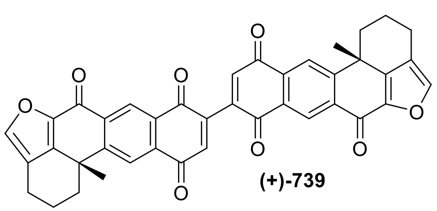

(+)-739

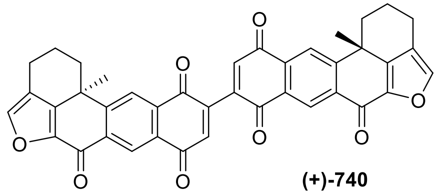

(+)-740

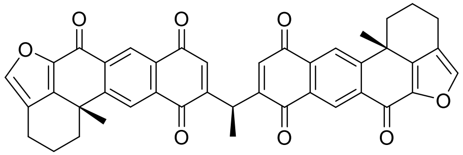

(+)-741

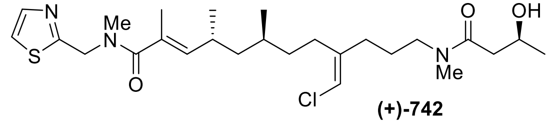

(+)-742

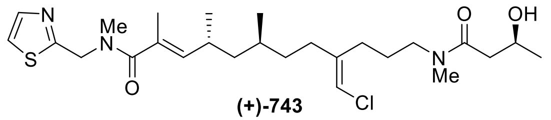

(+)-743

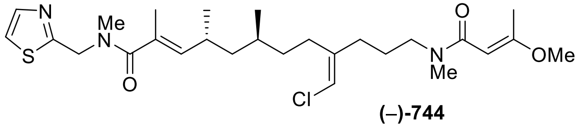

(–)-744

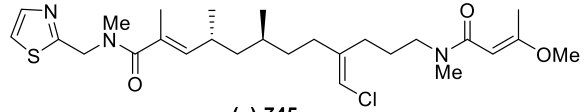

(–)-745

Figure S21: Structures of polyketides from Indonesian waters found in 1970–2017.

Table S21: Marine polyketides from Indonesian waters found in 1970–2017.

Compound	Structure Elucidation	Chemistry Type	Drug Class	Biological Activity		Source of Organism	Province	Ref
				Cell/Enzyme/Micro-organism/Insect/Others	Activity			
(+)-Karatungiol A **625**[β] [$C_{73}H_{132}O_{28}$]	UV, IR, MS, NMR, [α]D, CT	Polyol	Antifungal	*A. niger* NBRC4407<br>*T. foetus*	12 µg/disk<br>1 µg/mL	*Amphi-dinium* sp.	NSW	[44]
(+)-Karatungiol B **626**[β] [$C_{73}H_{130}O_{27}$]	UV, IR, MS, NMR, [α]D	Polyol	Undetm.	Undetm.	Undetm.	*Amphi-dinium* sp.	NSW	[44]
(+)-Manadic acid A **627**[β] [$C_{17}H_{28}O_5$]	UV, IR, MS, NMR, [α]D	Cyclic peroxide	Cytotoxic	P-388, A459, HT-29, LcV, MEL-28<br>MLR	IC_{50} = 0.5 – 5 µM<br>IC_{50} = 0.015 µM	*Plakortis* sp.	NSW	[412]
(+)-Manadic acid B **628**[β] [$C_{18}H_{30}O_5$]	UV, IR, MS, NMR, [α]D, CT	Cyclic peroxide	Cytotoxic	P-388, A549, HT-29, MEL-28<br>MLR, LcV	IC_{50} = 0.5 – 5 µM<br>NA	*Plakortis* sp.	NSW	[412]
(−)-(9*S*, 10*S*)-Plakorstatin 1 **629**/(−)-(9*R*, 10*R*)-Plakorstatin 1 **630**[α,β] [$C_{19}H_{32}O_5$]	MS, NMR, [α]D	Cyclic peroxide	Cytotoxic	P-388<br>BXPC-3, MCF7, NCI-H460, KM-20L2, DU-145<br>SF268	ED_{50} = 1.1 µg/mL<br>GI_{50} > 10 µg/mL<br>GI_{50} > 1.8 µg/mL	*P. nigra*	NSW	[413]
(−)-(9*R*, 10*R*)-Plakorstatin 1 **631**/(−)-(9*S*, 10*S*)-Plakorstatin 1 **632**[α,β] [$C_{19}H_{32}O_5$]	MS, NMR, [α]D	Cyclic peroxide	Cytotoxic	P-388<br>BXPC-3, KM-20L2<br>MCF7<br>SF268, DU-145<br>NCI-H460	ED_{50} = 0.91 µg/mL<br>GI_{50} = 6.4 – 6.7 µg/mL<br>GI_{50} = 3.8 µg/mL<br>GI_{50} = 1.6 – 1.7 µg/mL<br>GI_{50} > 10 µg/mL	*P. nigra*	NSW	[413]
(−)-Manadoperoxide A **633**[β] [$C_{18}H_{30}O_5$]	UV, IR, MS, NMR, [α]D, Mol. Mod., CT	Cyclic peroxide	Antiparasite	*P. falciparum* (D10), (W2)	IC_{50} = 3.74 ± 0.92 − 6.88 ± 0.37 µM	*P.* cfr. *simplex*	NSW	[414]
(−)-Manadoperoxide B **634**[β] [$C_{19}H_{32}O_5$]	UV, IR, MS, NMR, [α]D, Mol. Mod.	Cyclic peroxide	Antiparasite	*P. falciparum* (D10), (W2)<br>*T. brucei rhodesiense*<br>*L. donovani*	IC_{50} = 3.69 ± 0.88 − 6.76 ± 0.32 µM<br>IC_{50} = 0.003 µg/mL<br>IC_{50} = 0.589 µg/mL	*P.* cfr. *simplex*	NSW	[414, 415]
(−)-Manadoperoxide C **635**[β] [$C_{16}H_{26}O_6$]	UV, IR, MS, NMR, [α]D, Mol. Mod	Cyclic peroxide	Antiparasite	*P. falciparum* (D10), (W2)<br>*T. brucei rhodesiense*<br>*L. donovani*	IC_{50} = 2.33 ± 0.48 – 4.54 ± 0.66 µM<br>IC_{50} = 0.678 µg/mL<br>IC_{50} = 3.24 µg/mL	*P.* cfr. *simplex*	NSW	[414, 415]
(−)-Manadoperoxide D **636**[β] [$C_{18}H_{32}O_7$]	UV, IR, MS, NMR, [α]D, Mol. Mod	Cyclic peroxide	Antiparasite	*P. falciparum* (D10), (W2)<br>*T. brucei rhodesiense*	IC_{50} = 7.93 ± 0.68 − 10.38 ± 0.76 µM<br>IC_{50} = 19.2 − 36.7 µg/mL	*P.* cfr. *simplex*	NSW	[414, 415]
(−)-Manadoperoxide E **637**[β] [$C_{19}H_{34}O_7$]	MS, NMR, [α]D, CT	Cyclic peroxide	Undetm.	Undetm.	Undetm.	*P.* cfr. *simplex*	NSW	[415]

Table S21: *Cont.*

Compound	Structure Elucidation	Chemistry Type	Drug Class	Biological Activity		Source of Organism	Province	Ref
				Cell/Enzyme/Micro-organism/Insect/Others	Activity			
(−)-Manadoperoxide F **638**[β] [$C_{19}H_{34}O_7$]	MS, NMR, $[\alpha]_D$	Cyclic peroxide	Antiparasite	*T. brucei rhodesiense*<br>*L. donovani*	IC_{50} = 0.792 µg/mL<br>IC_{50} = 5.73 µg/mL	*P.* cfr. *simplex*	NSW	[415]
(−)-Manadoperoxide G **639**[β] [$C_{18}H_{30}O_8$]	MS, NMR, $[\alpha]_D$	Cyclic peroxide	Antiparasite	*T. brucei rhodesiense*<br>*L. donovani*	IC_{50} = 1.84 µg/mL<br>IC_{50} = 3.22 µg/mL	*P.* cfr. *simplex*	NSW	[415]
(−)-Manadoperoxide H **640**[β] [$C_{19}H_{34}O_6$]	MS, NMR, $[\alpha]_D$	Cyclic peroxide	Antiparasite	*T. brucei rhodesiense*<br>*L. donovani*	IC_{50} = 0.375 µg/mL<br>IC_{50} = 2.44 µg/mL	*P.* cfr. *simplex*	NSW	[415]
(−)-Manadoperoxide I **641**[β] [$C_{18}H_{30}O_7$]	MS, NMR, $[\alpha]_D$	Cyclic peroxide	Antiparasite	*T. brucei rhodesiense*<br>*L. donovani*	IC_{50} = 0.062 µg/mL<br>IC_{50} = 0.633 µg/mL	*P.* cfr. *simplex*	NSW	[415]
(−)-Manadoperoxide J **642**[β] [$C_{19}H_{31}ClO_7$]	MS, NMR, $[\alpha]_D$	Cyclic peroxide	Undetm.	Undetm.	Undetm.	*P.* cfr. *simplex*	NSW	[415]
(−)-Manadoperoxide K **643**[β] [$C_{20}H_{35}ClO_7$]	MS, NMR, $[\alpha]_D$	Cyclic peroxide	Antiparasite	*T. brucei rhodesiense*<br>*L. donovani*	IC_{50} = 0.087 µg/mL<br>IC_{50} = 1.89 µg/mL	*P.* cfr. *simplex*	NSW	[415]
(−)-Peroxyplakoric ester C **644**[β] [$C_{16}H_{26}O_6$]	MS, NMR, $[\alpha]_D$	Cyclic peroxide	Antiparasite	*T. brucei rhodesiense*<br>*L. donovani*	IC_{50} = 30.9 µg/mL<br>IC_{50} = 43.4 µg/mL	*P.* cfr. *simplex*	NSW	[415]
14,15-*seco*-Curvularin **645**[β] [$C_{16}H_{22}O_5$]	MS, NMR	Aromatic Polyketide[♠]	Antibacterial	*B. subtilis*	20% (200 µg/disk)	A fungus (symbiont) *S. vagabunda* (host)	CSW	[416]
(−)-Vidalenolone **646**[β] [$C_{13}H_{14}O_4$]	UV, IR, MS, NMR, $[\alpha]_D$, ECD, QCC	Aromatic Polyketide	Anticancer	Fyn-SH2	NA	*Vidalia* sp.	UEP	[417, 418]
Cosmochlorin A **647**[β] [$C_{18}H_{18}Cl_2O_4$]	UV, IR, MS, NMR	Aromatic Polyketide	Antibacterial	*S. aureus* NRBC 13276<br>*C. albicans* ATCC 2019	MIC = 15.6 µg/mL<br>MIC = 125 µg/mL	*C. vilior* IM2-155 (symbiont) *S. alba* (host)	WJV	[419]
			Antifungal	*A. clavatus* F318a, *T. harzianum* NBRC 33016	MIC = 15.6 − 62.5 µg/mL			
				V. dahliae Klebahn NBRC 9470	MIC > 125 µg/mL			
			Cytotoxic	HL-60	IC_{50} = 73.7 µM			
			Growth restore activity	*S. cerevisiae* YNS17 (0.3 M $CaCl_2$)	5 µg			
				GSK-3β	IC_{50} = 62.5 µM			
Cosmochlorin B **648**[β] [$C_{18}H_{18}Cl_2O_4$]	UV, IR, MS, NMR	Aromatic Polyketide	Cytotoxic	HL-60	IC_{50} = 53.6 µM	*C. vilior* IM2-155 (symbiont) *S. alba* (host)	WJV	[419]
			Growth restore activity	*S. cerevisiae* YNS17 (0.3 M $CaCl_2$)	1.25-5 µg			
				GSK-3β	IC_{50} = 60.6 µM			

Table S21: *Cont.*

Compound	Structure Elucidation	Chemistry Type	Drug Class	Biological Activity: Cell/Enzyme/Micro-organism/Insect/Others	Biological Activity: Activity	Source of Organism	Province	Ref
Cosmochlorin C **649**[β] [$C_{18}H_{18}Cl_2O_4$]	UV, IR, MS, NMR	Aromatic Polyketide	Antibacterial	*S. aureus* NRBC 13276	MIC = 15.6 μg/mL	*C. vilior* IM2-155 (symbiont) *S. alba* (host)	WJV	[419]
			Antifungal	*C. albicans* ATCC 2019	MIC = 125 μg/mL			
				A. clavatus F318a	MIC = 62.5 μg/mL			
				T. harzianum NBRC 33016	MIC = 15.6 μg/mL			
				V. dahliae Klebahn NBRC 9470	MIC > 125 μg/mL			
Acetyl sumiki's acid **650**[β] [$C_8H_8O_5$]	MS, NMR	Aromatic Polyketide	Antibacterial	*B. subtilis, S. aureus*	7 mm (5 μg)	*C. herbarum* (symbiont) *C. aerizusa* (host)	BLI	[420]
				E. coli, C. albicans	NA			
2-Carboxy-8-methoxy-naphthalene-1-ol **651**[β] [$C_{12}H_{10}O_4$]	MS, NMR	Aromatic Polyketide	Antibacterial	*S. aureus, P. aeruginosa, E. coli*	NA (100 μg/disk)	*M. sterillium*	UEP	[421]
			Antifungal	*C. maltosa*	NA (200 μg/disk)			
			Cytotoxic	5637 (ATCC HTB-9)	NA (IC_{50} = 0.34 mM)			
3,4,5-Tribromo-2-(2′-bromophenoxy)phenol **652**[β] [$C_{12}H_6Br_4O_2$]	UV, MS, NMR	Aromatic polyketide	Antibacterial	*B. subtilis*	MIC = 6.25 μg/mL	*D. herbacea*	WST	[422]
			Antifungal	*C. cucumerinum*	8.0 − 13.0 mm (25 − 50 mmol)			
			Cytotoxic	*A. salina*	LC_{50} = 3.30 ± 0.51 μg/mL			
3,5,6-Tribromo-2-(2′-bromophenoxy)phenol **653/** 3,4,6-Tribromo-2-(2′-bromophenoxy)phenol **654**[α,β] [$C_{12}H_6Br_4O_2$]	UV, MS, NMR	Aromatic polyketide	Antibacterial	*B. subtilis*	MIC = 1.56 μg/mL	*D herbacea*	WST	[422]
			Antifungal	*C. cucumerinum*	8.0 − 13.0 mm (25 − 50 mmol)			
			Cytotoxic	*A. salina*	LC_{50} = 3.30 ± 0.35 μg/mL			
3,5,6-Tribromo-1-(2′-bromophenoxy)-2-benzene methyl ether **655**[β] [$C_{13}H_8Br_4O_2$]	UV, MS, NMR	Aromatic polyketide	Antibacterial	*B. subtilis*	MIC = 104.00 μg/mL	*D. herbacea*	WST	[422]
			Cytotoxic	*A.salina*	LC_{50} = 26.25 ± 0.42 μg/mL			
**656**[β] [$C_{13}H_7Br_5O_3$]	UV, MS, NMR, CT	Aromatic polyketide	Antibacterial	*B. subtilis*	6 − 14 mm (0.1 − 10 μg/disk)	*L. herbacea*	BTN	[423]

Table S21: *Cont.*

Compound	Structure Elucidation	Chemistry Type	Drug Class	Biological Activity		Source of Organism	Province	Ref
				Cell/Enzyme/Micro-organism/Insect/Others	Activity			
**657**[β] [$C_{12}H_6Br_4O_3$]	UV, MS, NMR, CT	Aromatic Polyketide[♠]	Antibacterial	*B. subtilis* NBT-T2	7-20 mm (0.1 – 10 μg/disk) IC_{50}>15 μg/mL	*L. herbacea*	BTN	[423]
**658**[β] [$C_{13}H_7Br_5O_3$]	UV, MS, NMR, chemical transformation	Aromatic Polyketide	Antibacterial	*B. subtilis*	7 – 17 mm (0.1 – 10 μg/disk)	*L. herbacea*	BTN	[423]
			Cytotoxic	NBT-T2	IC_{50}>15 μg/mL			
**659a**[β] [$C_{13}H_7Br_5O_3$]	UV, MS, NMR	Aromatic Polyketide	Antibacterial	*B. subtilis*	6 – 13 mm (0.1 – 10 μg/disk)	*L. herbacea*	BTN	[423]
**659b**[δ] [$C_{13}H_7Br_5O_3$]	X-ray	Aromatic Polyketide	Anticancer	Mc1-1	IC_{50}>10 μg/mL	*L. herbacea*	PNG	[423, 424]
			Antibacterial	*B. subtilis*	6 – 13 mm (0.1 – 10 μg/disk)			
2-hydroxy-6-(2′-hydroxy-3′-hydroxymethyl-5-methylphenoxy)-benzoic acid **660**[β] [$C_{15}H_{14}O_6$]	UV, MS, NMR	Aromatic Polyketide[♠]	Antidiabetic	PTP1B, TCPTP, VHR	IC_{50}>35 μg/mL	*P. albobiver-ticillium* (symbiont) a tunicate (host)	NSW	[425]
				CD45	IC_{50}>43 μg/mL			
Aspergione A **661a**[β] [$C_{16}H_{16}O_4$]	UV, MS, NMR	Aromatic Polyketide	Undetm.	Undetm.	Undetm.	*A. versicolor* (symbiont) *X. exigua* (host)	BLI	[271]
(+)-Aspergione A **661b$_1$**[γ] [$C_{16}H_{18}O_4$]	UV, MS, NMR, $[\alpha]_D$	Aromatic Polyketide	Undetm.	Undetm.	Undetm.	*A. versicolor* (symbiont) *X. exigua* (host)	BLI	[272]
(+)-Aspergione A = (+)-Ustusorane C **661b$_2$**[δ] [$C_{16}H_{18}O_4$]	UV, MS, NMR, $[\alpha]_D$, TS	Aromatic Polyketide	Cytotoxic	A549, HL-60	IC_{50}> 100 μM	*A. ustus* 094102	PRC	[426, 427]
Aspergione B **662a**[β] [$C_{15}H_{12}O_4$]	UV, MS, NMR	Aromatic Polyketide	Undetm.	Undetm.	Undetm.	*A. versicolor* (symbiont) *X. exigua* (host)	BLI	[271]

Table S21: *Cont.*

Compound	Structure Elucidation	Chemistry Type	Drug Class	Biological Activity: Cell/Enzyme/Micro-organism/Insect/Others	Biological Activity: Activity	Source of Organism	Province	Ref
(+)-Aspergione B **662b1**[γ] [$C_{15}H_{16}O_4$]	UV, MS, NMR, $[\alpha]_D$	Aromatic Polyketide	Undetm.	Undetm.	Undetm.	*A. versicolor* (symbiont) *X. exigua* (host)	BLI	[272]
(+)-Aspergione B = (+)-pseudodeflectusin **662b2**[δ] [$C_{15}H_{16}O_4$]	TS	Aromatic Polyketide	Undetm.	Undetm.	Undetm.			[427, 428]
Aspergione C **663a**[β] [$C_{16}H_{18}O_4$]	UV, MS, NMR	Aromatic Polyketide	Undetm.	Undetm.	Undetm.	*A. versicolor* (symbiont) *X. exigua* (host)	BLI	[271]
(+)-Aspergione C **663b**[γ] [$C_{16}H_{16}O_4$]	UV, MS, NMR, $[\alpha]_D$	Aromatic Polyketide	Antibacterial	*B. subtilis, E. coli*	NA	*A. versicolor* (symbiont) *X. exigua* (host)	BLI	[272]
			Antifungal	*S. cerevisiae*	NA			
Aspergione D **664a**[β] [$C_{15}H_{16}O_4$]	UV, MS, NMR	Aromatic Polyketide	Undetm.	Undetm.	Undetm.	*A. versicolor* (symbiont) *X. exigua* (host)	BLI	[271]
Aspergione D **664b**[γ] [$C_{15}H_{12}O_4$]	UV, MS, NMR, $[\alpha]_D$	Aromatic Polyketide	Undetm.	Undetm.	Undetm.	*A. versicolor* (symbiont) *X. exigua* (host)	BLI	[272]
Aspergione E **665a**[β] [$C_{16}H_{18}O_4$]	UV, MS, NMR	Aromatic Polyketide	Undetm.	Undetm.	Undetm.	*A. versicolor* (symbiont) *X. exigua* (host)	BLI	[271]
(+)-Aspergione E **665b1**[γ] [$C_{16}H_{18}O_4$]	UV, MS, NMR, $[\alpha]_D$	Aromatic Polyketide	Antibacterial	*B. subtilis, E. coli*	NA	*A. versicolor* (symbiont) *X. exigua* (host)	BLI	[272]
			Antifungal	*S. cerevisiae*	NA			

Table S21: *Cont.*

Compound	Structure Elucidation	Chemistry Type	Drug Class	Biological Activity: Cell/Enzyme/Micro-organism/Insect/Others	Biological Activity: Activity	Source of Organism	Province	Ref
Aspergione E = pergillin **665b₂**[δ] [$C_{15}H_{16}O_4$]	TS	Aromatic Polyketide	Undetm.	Undetm.	Undetm.			[429]
Aspergione F **666a**[β] [$C_{15}H_{16}O_4$]	UV, MS, NMR	Aromatic Polyketide	Undetm.	Undetm.	Undetm.	*A. versicolor* (symbiont) *X. exigua* (host)	BLI	[271]
(−)-Aspergione F **666b₁**[γ] [$C_{16}H_{18}O_4$]	UV, MS, NMR, [α]D	Aromatic Polyketide	Undetm.	Undetm.	Undetm.	*A. versicolor* (symbiont) *X. exigua* (host)	BLI	[272]
Aspergione F = pergillin **666b₂** = **666b₂**[δ] [$C_{15}H_{16}O_4$]	TS	Aromatic Polyketide	Undetm.	Undetm.	Undetm.			[429]
Lunatin **667**[β] [$C_{15}H_{10}O_6$]	MS, NMR	Aromatic Polyketide	Antibacterial<br>Antifungal	*S. aureus*<br>*E. coli*<br>*E. coli* HBI-101<br>*B. subtilis*<br>*C. albicans*	8.5 – 10.0 mm (5 – 10 μg)<br>9.0 – 11.0 mm (5 – 10 μg)<br>8.0 – 10.5 mm (5 – 10 μg)<br>7.5 – 9.0 mm (5 – 10 μg)<br>NA	*C. lunata* (symbiont) *N. olemda* (host)	BLI	[430]
(−)-Tetrahydrobostrycin **668**[β] [$C_{16}H_{20}O_8$]	UV, IR, MS, NMR, [α]D	Aromatic Polyketide	Antibacterial	*S. aureus*, *E. coli*	9.2 – 15 mm (100 μg/disk)	*Aspergillus* sp.	NSW	[431]
(−)-1-Deoxytetrahydro bostrycin **669**[β] [$C_{16}H_{20}O_7$]	UV, IR, MS, NMR, [α]D	Aromatic Polyketide	Antibacterial<br>Antifungal	*S. aureus*<br>*S. cerevisiae*, *M. hiemalis*	12 mm (100 μg/disk)<br>NA (100 μg/disk)	*Aspergillus* sp. (symbiont) an alga (host)	NSW	[431]
(+)-12-O-Deacetyl-phomoxanthone A **670**[β] [$C_{36}H_{36}O_{15}$]	UV, IR, MS, NMR, ECD, [α]D, CT	Aromatic Polyketide	Antifungal	*T. harzianum* NBRC 33016, *V. dahliae* Klebahn NBRC 9470, *D. medusae* Nitschke NBRC 30895<br>*S. sclerotiorum* de Bary NBRC 103652, *B. cinerea* Persoon NBRC 100717	NA<br>11–12 mm (30 μg)	*Phomopsis* sp. (symbiont) *R. mucronata* (host)	JSCR	[432 – 433]

Table S21: *Cont.*

Compound	Structure Elucidation	Chemistry Type	Drug Class	Biological Activity: Cell/Enzyme/Micro-organism/Insect/Others	Biological Activity: Activity	Source of Organism	Province	Ref
(+)-12-O-Deacetyl-phomoxanthone A **670**[β] [$C_{36}H_{36}O_{15}$]	UV, IR, MS, NMR, ECD, [α]D, CT	Aromatic Polyketide	Antibacterial	*S. aureus* NBRC 13276<br>*P. aeruginosa* ATCC 15442	9 mm (30 µg)<br>NA	*Phomopsis* sp. (symbiont) *R. mucronata* (host)	JSCR	[432–433]
			Cytotoxic	L5178Y	IC_{50} = 2.8 µM			
(+)-Komodoquinone A **671**[β] [$C_{28}H_{33}NO_9$]	UV, IR, MS, NMR, [α]D, CT	Aromatic Polyketide[♠]	Anticancer	Neuro 2A	1 µg/mL (morp. cng. mltplr. proc.)<br>1 µg/mL (G1 phase)	*Streptomyces* sp. KS3	ENT	[434, 435]
(+)-Komodoquinone B **672**[β] [$C_{19}H_{16}O_7$]	UV, IR, MS, NMR, [α]D, CT	Aromatic Polyketide	Anticancer	Neuro 2A	NA (3 µg/mL)	*Streptomyces* sp. KS3		
Xylarianaphthol-1 **673**[β] [$C_{21}H_{14}O_4$]	IR, MS, NMR, TS	Aromatic Polyketide[♠]	Anticancer	Transfected MG63	Activator p21 promoter (0.3 µM)	A fungus (*Xylariales*, symbiont) a sponge *(host)*	UEP	[436]
(+)-(*S*)-2,2'-dimethoxy-1,1'-binaphthyl-5,5',6,6'-tetraol **674**[β] [$C_{22}H_{18}O_6$]	UV, IR, MS, NMR, [α]D, ECD	Aromatic Polyketide	Antitumor	HIF-1 (T47D)	IC_{50} = 4.3 µM	*Lendenfeldia* sp.	UEP	[411]
			Cytotoxic	MDA-MB-231 − T47D (hypoxic)	IC_{50} = 7.0 − 8.3 µM			
(+)-Spiciferol A **675**[β] [$C_{14}H_{18}O_3$]	UV, MS, NMR, [α]D	Aromatic Polyketide	Undetm.	Undetm.	Undetm.	*D. hawaiien-sis* (symbiont) *C. aerizusa* (host)	BLI	[437]
(−)-Butoxyl spiciferin A **676**[β] [$C_{14}H_{20}O_4$]	UV, MS, NMR, [α]D	Aromatic Polyketide	Undetm.	Undetm.	Undetm.	*D. hawaiien-sis* (symbiont) *C. aerizusa* (host)	BLI	[437]
(+)-Epoxysorbicillinol **677**[β] [$C_{14}H_{16}O_5$]	UV, IR, MS, NMR, [α]D, ECD	Vertinoid Polyketide[♠]	Undetm.	Undetm.	Undetm.	*T. longibra-chiatums* (symbiont) *Halichlona* (host)	UEP	[438]

Table S21: *Cont.*

Compound	Structure Elucidation	Chemistry Type	Drug Class	Biological Activity: Cell/Enzyme/Micro-organism/Insect/Others	Biological Activity: Activity	Source of Organism	Province	Ref
Cadiolide A **678**[β] [$C_{24}H_{12}Br_4O_6$]	UV, IR, MS, NMR	γ-Lactone[♣]	Cytotoxic	HCT116	NA	*Botryllus* sp.	SSW	[439]
Cadiolide B **679**[β] [$C_{24}H_{10}Br_6O_6$]	UV, IR, MS, NMR	γ-Lactone	Cytotoxic	HCT116	NA	*Botryllus* sp.	SSW	[439]
(−)-*iso*-Cladospolide B **680**[β] [$C_{12}H_{20}O_4$]	IR, MS, NMR, [α]D, TS	γ-Lactone	Antibacterial	Gram-positive bacteria, Gram-negative bacteria	NA (250 µg/disk)	A fungus (symbiont) a sponge (host)	SSW	[397, 398, 440, 441]
(−)-Herbaric acid **681**[β] [$C_{10}H_8O_6$]	MS, NMR, [α]D, TS	γ-Lactone	Cytotoxic	HL-60<br>*A. salina*	NA<br>NA	*C. herbarum* (symbiont) *A. aerophoba* (host)	BLI	[440, 442]
(+)-Biaketide **682**[β] [$C_{17}H_{27}Cl_2O$]	IR, MS, NMR, [α]D	γ-Lactone[♣]	Cytotoxic	NBT-T2	IC_{50} = 8.3 µg/mL	*Dysidea* sp.	PUA	[356]
(−)-Plakofuranaolactone **683**[β] [$C_8H_{12}O_4$]	MS, NMR, [α]D, QCC	γ-Lactone	Antibacterial (Quorum quenching agent)	*E. coli* pSB1075, *E. coli* pSB401, *C. violeaceum* CV026), pyocyanin, total protease activity	0.781 – 200 µM	*Plakortis cf. lita*	NSW	[443]
Herbarin A **684**[β] [$C_{12}H_{12}O_5$]	MS, NMR	δ-Lactone	Cytotoxic	*A. salina*	75% (50 µg)<br>85% (100 µg)	*C. herbarum* (symbiont) *C aerizusa* (host)	BLI	[430]
Herbarin B **685**[β] [$C_{10}H_{10}O_5$]	MS, NMR	δ-Lactone	Cytotoxic	*A. salina*	65% (50 µg)<br>80% (100 µg)	*C. herbarum* (symbiont) *C. aerizusa* (host)	BLI	[430]
**686**[β] [$C_{14}H_{20}O_4$]	UV, IR, MS, NMR	δ-Lactone	Growth restore activity	*S. cerevisiae* YNS17 (0.3 M $CaCl_2$)	1.56-25 µg	*Fusarium* sp. (symbiont) *R. mucronata* (host)	JSCR	[444]

Table S21: *Cont.*

Compound	Structure Elucidation	Chemistry Type	Drug Class	Biological Activity		Source of Organism	Province	Ref
				Cell/Enzyme/Micro-organism/Insect/Others	Activity			
(+)-Helicascolide C **687**[β] [$C_{12}H_{18}O_3$]	UV, IR, MS, NMR, CD, $[\alpha]_D$, X-ray, TS	δ-Lactone	Antifungal<br><br>Antibacterial<br><br>Cytotoxic	*C. cucumerinum*<br>*C. maltosa*<br>*E. coli, P. aeruginosa*<br>*S. aureus, B. subtilis*<br>5637	78.5 mm² (200 μg/disk)<br>NA (200 μg/disk)<br>NA (200 μg/disk)<br>NA (300 μg/disk)<br>NA (IC_{50} = 1.18 mM)	*D. eschsholzii* (symbiont) *Gracilaria* sp. (host)	SSW	[445–447]
(+)-Bitungolide A **688**[β] [$C_{25}H_{33}ClO_5$]	UV, IR, MS, NMR, $[\alpha]_D$, X-ray	δ-Lactone	Cytotoxic<br>Antidiabetic	3Y1<br>VHR<br>PTP-S2, PP1 and PP2A	10 μg/mL<br>IC_{50} > 10 μg/mL<br>IC_{50} > 30 μg/mL	*T.* cf. *swinhoei*	NSW	[448]
(+)-Bitungolide B **689a**[β] [$C_{25}H_{33}ClO_5$]	UV, IR, MS, NMR, $[\alpha]_D$	δ-Lactone	Cytotoxic<br>Antidiabetic	3Y1<br>VHR<br>PTP-S2, PP1 and PP2A	10 μg/mL<br>IC_{50} > 10 μg/mL<br>IC_{50} > 30 μg/mL	*T.* cf. *swinhoei*	NSW	[448]
(−)-Bitungolide B **689b**[γ] [$C_{25}H_{33}ClO_5$]	TS	δ-Lactone	Undetm.	Undetm.	Undetm.			[449]
(+)-Bitungolide C **690**[β] [$C_{25}H_{33}ClO_5$]	UV, IR, MS, NMR, $[\alpha]_D$	δ-Lactone	Cytotoxic<br>Antidiabetic	3Y1<br>VHR<br>PTP-S2, PP1 and PP2A	10 μg/mL<br>IC_{50} > 10 μg/mL<br>IC_{50} > 30 μg/mL	*T.* cf. *swinhoei*	NSW	[448]
(+)-Bitungolide D **691**[β] [$C_{25}H_{33}ClO_5$]	UV, IR, MS, NMR, $[\alpha]_D$	δ-Lactone	Cytotoxic<br>Antidiabetic	3Y1<br>VHR<br>PTP-S2, PP1 and PP2A	10 μg/mL<br>IC_{50} > 10 μg/mL<br>IC_{50} > 30 μg/mL	*T.* cf. *swinhoei*	NSW	[448]
(+)-Bitungolide E **692a**[β] [$C_{25}H_{34}O_4$]	UV, IR, MS, NMR, $[\alpha]_D$	δ-Lactone	Cytotoxic<br>Antidiabetic	3Y1<br>VHR<br>PTP-S2, PP1 and PP2A	10 μg/mL<br>IC_{50} > 10 μg/mL<br>IC_{50} > 30 μg/mL	*T.* cf. *swinhoei*	NSW	[448]
(−)-Bitungolide E **692b**[γ] [$C_{25}H_{34}O_4$]	TS	δ-Lactone	Undetm.	Undetm.	Undetm.			[449, 450]
(+)-Bitungolide F **693a**[β] [$C_{24}H_{32}O_5$]	UV, IR, MS, NMR, $[\alpha]_D$	δ-Lactone	Cytotoxic<br>Antidiabetic	3Y1<br>VHR<br>PTP-S2, PP1 and PP2A	10 μg/mL<br>IC_{50} > 10 μg/mL<br>IC_{50} > 30 μg/mL	*T.* cf. *swinhoei*	NSW	[448]
(−)-Bitungolide F **693b**[γ] [$C_{25}H_{34}O_4$]	TS	δ-Lactone	Undetm.	Undetm.	Undetm.			[451]

Table S21: *Cont.*

Compound	Structure Elucidation	Chemistry Type	Drug Class	Biological Activity: Cell/Enzyme/Micro-organism/Insect/Others	Biological Activity: Activity	Source of Organism	Province	Ref
(+)-Xestodecalactone A **694**[β] [$C_{14}H_{16}O_5$]	UV, IR, MS, NMR, [α]D, ECD, QCC, TS	Macrolactone	Antibacterial	*B. subtilis*	NA	*P.* cf. *montanense* (symbiont) *X. exigua* (host)	BLI	[452–454]
				S. aureus	NA			
				E. coli	NA			
(+)-Xestodecalactone B **695**[β] [$C_{14}H_{16}O_6$]	UV, IR, MS, NMR, [α]D, ECD, QCC, TS	Macrolactone	Antifungal	*C. albicans*	7 mm (20 μmol) 12 mm (50 μmol) 25 mm (100 μmol)	*P.* cf. *montanense* (symbiont) *X. exigua* (host)	BLI	[452, 455]
			Antibacterial	*B. subtilis, S. aureus, E. coli*	NA			
(+)-Xestodecalactone C **696**[β] [$C_{14}H_{16}O_6$]	UV, IR, MS, NMR, [α]D, ECD, QCC, TS	Macrolactone	Antibacterial	*B. subtilis, S. aureus, E. coli*	NA	*P.* cf. *montanense* (symbiont) *X. exigua* (host)	BLI	[452, 456–458]
(−)-Pandangolide 1 **697**[β] [$C_{12}H_{20}O_5$]	UV, IR, MS, NMR, [α]D	Macrolactone	Antibacterial	Gram-positive bacteria, Gram-negative bacteria	NA (250 μg)	A fungus (symbiont) a sponge (host)	SSW	[397, 459]
(−)-Pandangolide 2 **698**[β] [$C_{14}H_{22}O_6S$]	UV, IR, MS, NMR, [α]D	Macrolactone	Antibacterial	Gram-positive bacteria, Gram-negative bacteria	NA (250 μg)	A fungus (symbiont) a sponge (host)	SSW	[397]
(−)-Pandangolide 3 **699**[β] [$C_{16}H_{26}O_7S$]	MS, NMR, [α]D	Macrolactone	Antibacterial	Gram-positive bacteria, Gram-negative bacteria	NA	*C. herbarum* (symbiont) *C. aerizusa* (host)	BLI	[420]
(−)-Pandangolide 4 **700**[β] [$C_{24}H_{38}O_8S$]	MS, NMR, [α]D	Macrolactone[♠]	Antibacterial	Gram-positive bacteria, Gram-negative bacteria	NA	*C. herbarum* (symbiont) *C. aerizusa* (host)	BLI	[420]

Table S21: *Cont.*

Compound	Structure Elucidation	Chemistry Type	Drug Class	Biological Activity		Source of Organism	Province	Ref
				Cell/Enzyme/Micro-organism/Insect/Others	Activity			
(−)-Callyspongiolide **701a**$^{\beta}$ [$C_{33}H_{44}BrNO_6$]	UV, IR, MS, NMR, $[\alpha]_D$, CT	Macrolactone♣	Cytotoxic	L5178Y	IC_{50} = 320 nM	*Callyspongia* sp.	MLU	[460]
				Jurkat, Ramos B	IC_{50} = 60 – 70 nM (48 h)			
				Induc. hypodiploid nuclei (Jurkat), (Ramos B)	IC_{50} = 50 – 80 nM (48 h)			
(−)-Callyspongiolide **701b**$^{\gamma}$ [$C_{33}H_{44}BrNO_6$]	TS	Macrolactone	Cytotoxic	MCF7, SH-SY5Y, HT-29, H1299, PC3	IC_{50} = 0.119 – 0.467 µM			[460, 461, 462]
				HeLa, RKO	IC_{50} = 3.55 – 3.56 µM			
				Jurkat	IC_{50} = 0.0111 µM			
(+)-Phormidolide **702**$^{\beta}$ [$C_{59}H_{97}BrO_{12}$]	UV, IR, MS, NMR, $[\alpha]_D$, CT, Mol. Mod.	Macrolactone♣	Cytotoxic	*A. salina*	LD_{50} = 1.5 µM	*Phormidium* sp.	UEP	[463]
				NCI *in vitro* 60-cell line	NA			
(+)-**703**$^{\alpha,\beta}$ [$C_{48}H_{80}O_{14}$]	IR, MS, NMR, $[\alpha]_D$, CT	Macrolactone	Cytotoxic	NBT-T2	IC_{50} = 4.7 ng/mL	*Candida-spongia* sp.	ENT	[464]
(+)-**704**$^{\beta}$ [$C_{32}H_{50}O_{13}$]	IR, MS, NMR, $[\alpha]_D$, CT	Macrolactone	Cytotoxic	NBT-T2	IC_{50} = 19 ng/mL	*Candida-spongia* sp.	ENT	[464]
(−)-Laulimalide = (−)-Fijianolide B **705**$^{\beta}$ [$C_{30}H_{42}O_7$]	UV, IR, MS, NMR, $[\alpha]_D$, X-ray, CT, TS	Macrolactone♣	Cytotoxic	P-388, KB, A549, MEL-28, HT	IC_{50} = 10-50 ng/mL	*Hyattella* sp. *C. lochi*	NSW	[465 – 478]
				HT-29	IC_{50} = 6.9 nM (72 – 96 h)			
					IC_{50} = 5.74 ± 0.58 nM (48 h)			
				MDA-MB-435	IC_{50} = 2.3 ± 0.2 nM (72 – 96 h)			
				SK-OV-3	IC_{50} = 11.53 ± 0.53 nM (48 h)			
				MCF7	IC_{50} = 7 ng/mL, IC_{50} = 3.8 nM IC_{50} = 11.6 ± 0.5 nM (72 h)			
				MaTu	IC_{50} = 3.8 nM			
				HCT116, PC-3M	IC_{50} = 5.9 ± 0.3 – 7.8 ± 0.8 nM (72 h)			
				NCI/ADR	IC_{50} = 36 nM			
				MaTu/ADR	IC_{50} = 6.0 nM			
				SKVLB-1	IC_{50} = 1210 ± 490 nM (48 h)			
				Potency tubulin polymerization	EC_{50} = 4.0 ± 0.5 µM EC_{50} = 4.32 ± 0.4 µM			

Table S21: *Cont.*

Compound	Structure Elucidation	Chemistry Type	Drug Class	Biological Activity: Cell/Enzyme/Micro-organism/Insect/Others	Biological Activity: Activity	Source of Organism	Province	Ref
(−)-Isolaulimalide = (−)-Fijianolide A **706**β [$C_{30}H_{42}O_7$]	UV, IR, MS, NMR, $[\alpha]_D$, CT, TS	Macrolactone	Cytotoxic	MDA-MB-435<br>MCF7, PC-3M, HCT116	IC_{50} = 1970 ± 97 – 2650 ± 1384 nM (48 h)<br>IC_{50} = 4400 ± 300 – 4900 ± 200 nM (72 h)	*Hyatella* sp.<br>*C. lochi*	NSW	[465, 471, 476, 479]
(−)-Isoswinholide B **707**β [$C_{78}H_{132}O_{20}$]	MS, NMR, $[\alpha]_D$	Macrolactone	Cytostatic	HepG2	IC_{50} = 1500 nM	*T. swinhoei*	NSW	[480]
(−)-Swinholide K **708**β [$C_{78}H_{132}O_{21}$]	MS, NMR, $[\alpha]_D$	Macrolactone	Cytostatic	HepG2	IC_{50} = 15 nM	*T. swinhoei*	NSW	[480]
(−)-Xestosaprol L **709**β [$C_{20}H_{18}O_3$]	UV, IR, MS, NMR, $[\alpha]_D$	Quinone	Alzheimer	BACE1	IC_{50} = 98 ± 8 μM	*Xestospongia* sp.	EKM	[481]
(−)-Xestosaprol I **710**β [$C_{20}H_{18}O_3$]	UV, IR, MS, NMR, $[\alpha]_D$	Quinone	Alzheimer	BACE1	IC_{50} = 163 ± 11 μM	*Xestospongia* sp.	EKM	[481]
(−)-Xestosaprol J **711**β [$C_{21}H_{20}O_4$]	UV, IR, MS, NMR, $[\alpha]_D$	Quinone	Alzheimer	BACE1	IC_{50} = 90 ± 5 μM	*Xestospongia* sp.	EKM	[481]
(−)-Xestosaprol K **712**β [$C_{20}H_{18}O_4$]	UV, IR, MS, NMR, $[\alpha]_D$	Quinone	Alzheimer	BACE1	IC_{50} = 93 ± 4 μM	*Xestospongia* sp.	EKM	[481]
(−)-Xestosaprol G **713**β [$C_{20}H_{18}O_4$]	UV, IR, MS, NMR, $[\alpha]_D$	Quinone	Alzheimer	BACE1	IC_{50} = 155 ± 15 μM	*Xestospongia* sp.	EKM	[481]
(−)-Xestosaprol H **714**β [$C_{22}H_{22}O_5$]	UV, IR, MS, NMR, $[\alpha]_D$	Quinone	Alzheimer	BACE1	IC_{50} = 82 ± 3 μM	*Xestospongia* sp.	EKM	[481]
(−)-Xestosaprol F **715**β [$C_{22}H_{22}O_5$]	UV, IR, MS, NMR, $[\alpha]_D$	Quinone	Alzheimer	BACE1	IC_{50} = 135 ± 11 μM	*Xestospongia* sp.	EKM	[481]
(−)-Xestosaprol D **716**β [$C_{20}H_{18}O_4$]	UV, IR, MS, NMR, $[\alpha]_D$	Quinone	Antibacterial<br>Cytotoxic<br>Anticancer<br>Alzheimer	VRE, *E. coli*, MRSA, *S. aureus*<br>SKOV-3<br>PKCδ<br>BACE1	IC_{50} > 50 μg/mL<br>IC_{50} > 50 μg/mL<br>NA<br>IC_{50} = 30 μg/mL	*Xestospongia* sp.	EKM	[482]
(−)-Xestosaprol E **717**β [$C_{20}H_{18}O_4$]	UV, IR, MS, NMR, $[\alpha]_D$	Quinone	Antibacterial<br>Cytotoxic<br>Anticancer	VRE, *E. coli*, MRSA, *S. aureus*<br>SKOV-3<br>PKCδ	IC_{50} > 50 μg/mL<br>IC_{50} > 50 μg/mL<br>NA	*Xestospongia* sp.	EKM	[482]
(+)-Xestosaprol M **718**β [$C_{20}H_{16}O_2$]	UV, IR, MS, NMR, $[\alpha]_D$	Quinone	Alzheimer	BACE1	IC_{50} = 104 ± 8 μM	*Xestospongia* sp.	EKM	[481]

Table S21: *Cont.*

Compound	Structure Elucidation	Chemistry Type	Drug Class	Biological Activity: Cell/Enzyme/Micro-organism/Insect/Others	Biological Activity: Activity	Source of Organism	Province	Ref
(−)-14-Carboxyxestoquinol sulfate **719**$^{\beta}$ [$C_{21}H_{16}O_9S$]	UV, IR, MS, NMR, $[\alpha]_D$	Quinone	Anticancer	CDK1, CDK2, CDK5, CDK9, CK1, CLK1, DYRK1A, GSK3	IC_{50} > 10 μM	*Xestospongia* sp.	NSW	[483]
			Antibacterial	*S. aureus* ATCC 6538, *E. coli* ATCC 8739	NA			
			Antioxidant	DPPH	NA			
(−)-1-(2-Hydroxyethyl) xestoquinone **720**$^{\beta}$ [$C_{22}H_{18}O_5$]	UV, IR, MS, NMR, $[\alpha]_D$	Quinone	Anticancer	USP7	IC_{50} = 1.4 μM	*P. alfiani*	NSW	[484]
1-(1-hydroxyethyl)-xestoquinone **721**/**722**$^{\alpha,\beta}$ [$C_{22}H_{18}O_5$]	MS, NMR	Quinone	Undetm.	Undetm.	Undetm.	*P. alfiani*	NSW	[484]
(−)-3*S*-3-Hydroxy xestoquinone **723**$^{\beta}$	UV, IR, MS, NMR, $[\alpha]_D$, ECD	Quinone	Undetm.	Undetm.	Undetm.	*P. alfiani*	NSW	[484]
(+)-Noelaquinone **724**$^{\beta}$ [$C_{21}H_{15}N_3O_5$]	UV, IR, MS, NMR, $[\alpha]_D$	Quinone♠	Undetm.	Undetm.	Undetm.	*Xestospongia* sp.	EKM	[485]
(+)-3-Ketoadociquinone B **725**$^{\beta}$ [$C_{21}H_{15}N_3O_5$]	UV, IR, MS, NMR, $[\alpha]_D$	Quinone	Anticancer	Recombinant human Cdc25B catalytic domain	IC_{50} = 0.13 ± 0.02 μM	*Xestospongia* sp.	NSW	[486]
				Recombinant human Cdc25B full length	IC_{50} = 0.21 ± 0.01 μM			
				Recombinant human VHR	IC_{50} = 9.0 ± 0.2 μM			
				Recombinant human PTP1B	IC_{50} = 3.9 ± 0.2 μM			
Xestoadociaquinones A **726**/ B **727**$^{\alpha,\beta}$ [$C_{20}H_{19}NO_8S$]	MS, NMR	Quinone	Undetm.	Undetm.	Undetm.	*Xestospongia* sp.	NSW	[483]
(−)-Xestoadociaminal A = Petroquinone I **728**$^{\beta}$ [$C_{24}H_{21}NO_7S$]	UV, IR, MS, NMR, ECD, $[\alpha]_D$	Quinone♦	Anticancer	USP7	IC_{50} > 5.0 μM	*Xestospongia* sp. *P. alfiani*	NSW	[483, 484]
Xestoadociaminal B = (+)-Petroquinone J **729**$^{\beta}$ [$C_{24}H_{21}NO_7S$]	UV, IR, MS, NMR, ECD, $[\alpha]_D$	Quinone	Anticancer	USP7	IC_{50} > 5.0 μM	*Xestospongia* sp. *P. alfiani*	NSW	[483, 484]
(+)-Petroquinone K **730**$^{\beta}$ [$C_{24}H_{21}NO_7S$]	UV, IR, MS, NMR, ECD, $[\alpha]_D$	Quinone	Anticancer	USP7	IC_{50} > 5.0 μM	*P. alfiani*	NSW	[484]
(+)-Petroquinone L **731**$^{\beta}$ [$C_{24}H_{21}NO_7S$]	UV, IR, MS, NMR, ECD, $[\alpha]_D$	Quinone	Anticancer	USP7	IC_{50} > 5.0 μM	*P. alfiani*	NSW	[484]

Table S21: *Cont.*

Compound	Structure Elucidation	Chemistry Type	Drug Class	Biological Activity		Source of Organism	Province	Ref
				Cell/Enzyme/Micro-organism/Insect/Others	Activity			
Xestoadociaminals C **732**/ D **733**[α,β] [$C_{24}H_{23}NO_7S$]	MS, NMR	Quinone	Undetm.	Undetm.	Undetm.	*Xestospongia* sp.	NSW	[483]
(+)-Petroquinone A **734**[β] [$C_{60}H_{36}O_{12}$]	UV, IR, MS, NMR, ECD, [α]D	Quinone[♠]	Anticancer	USP7	IC_{50} = 0.75 μM	*P. alfiani*	NSW	[484]
(−)-Petroquinone B **735**[β] [$C_{60}H_{36}O_{12}$]	UV, IR, MS, NMR, ECD, [α]D	Quinone	Anticancer	USP7	IC_{50} = 0.36 μM	*P. alfiani*	NSW	[484]
(−)-Petroquinone C **736**[β] [$C_{40}H_{30}O_{13}S$]	UV, IR, MS, NMR, ECD, [α]D	Quinone[♣]	Anticancer	USP7	IC_{50} = 2.0 μM	*P. alfiani*	NSW	[484]
(+)-Petroquinone D **737**[β] [$C_{40}H_{32}O_{15}S_2$]	UV, IR, MS, NMR, ECD, [α]D	Quinone	Anticancer	USP7	IC_{50} > 5.0 μM	*P. alfiani*	NSW	[484]
(+)-Petroquinone E **738**[β] [$C_{40}H_{28}O_{11}S$]	UV, IR, MS, NMR, ECD, [α]D	Quinone	Anticancer	USP7	IC_{50} = 1.2 μM	*P. alfiani*	NSW	[484]
(+)-Petroquinone F **739**[β] [$C_{40}H_{26}O_8$]	UV, IR, MS, NMR, ECD, [α]D	Quinone	Anticancer	USP7	IC_{50} = 0.35 μM	*P. alfiani*	NSW	[484]
(+)-Petroquinone G **740**[β] [$C_{40}H_{26}O_8$]	UV, IR, MS, NMR, ECD, [α]D	Quinone	Anticancer	USP7	IC_{50} = 0.47 μM	*P. alfiani*	NSW	[484]
(+)-Petroquinone H **741**[β] [$C_{42}H_{30}O_8$]	UV, IR, MS, NMR, ECD, [α]D	Quinone	Anticancer	USP7	IC_{50} = 0.49 μM	*P. alfiani*	NSW	[484]
(+)-Biakamide A **742**[β] [$C_{26}H_{42}ClN_3O_3S$]	UV, IR, MS, NMR, [α]D, ECD, CT, TS	Halogenated polyketide[♠]	Cytostatic	PANC-1	IC_{50} = 1.0 μM (Glu.-Def. Med.) IC_{50} > 100 μM (Gen. Glu. Med.)	*Petrosas-pongia* sp.	PUA	[487]
(+)-Biakamide B **743**[β] [$C_{26}H_{42}ClN_3O_3S$]	UV, IR, MS, NMR, [α]D, CT, TS	Halogenated polyketide	Cytostatic	PANC-1	IC_{50} = 4.0 μM (Glu.-Def. Med.) IC_{50} > 100 μM (Gen. Glu. Med.)	*Petrosas-pongia* sp	PUA	[487]
(−)-Biakamide C **744**[β] [$C_{27}H_{42}ClN_3O_3S$]	UV, IR, MS, NMR, [α]D, CT, TS	Halogenated polyketide	Cytostatic	PANC-1	IC_{50} = 0.5 μM (Glu.-Def. Med.) IC_{50} = 50 μM (Gen. Glu. Med.)	*Petrosas-pongia* sp	PUA	[487]

Table S21: *Cont.*

Compound	Structure Elucidation	Chemistry Type	Drug Class	Biological Activity		Source of Organism	Province	Ref
				Cell/Enzyme/Micro-organism/Insect/Others	Activity			
(−)-Biakamide D **745**[β] [$C_{27}H_{42}ClN_3O_3S$]	UV, IR, MS, NMR, $[\alpha]_D$, CT, TS	Halogenated polyketide	Cytostatic	PANC-1	IC_{50} = 0.5 μM (Glu.-Def. Med.) IC_{50} = 35 μM (Gen. Glu. Med.)	*Petrosas-pongia* sp	PUA	[487]

Footnote: **1. Activity** (**3Y1** murine normal fibroblast, **H1299** human lung carcinoma, **MaTu** human breast adenocarcinoma, **MaTu/ADR** human multi-drug resistant breast adenocarcinoma, **MG63** human osteosarcoma, **NCI/ADR** human multi-drug resistant breast adenocarcinoma, **PC-3M** human prostate carcinoma, **RKO** human colorectal adenocarcinoma, **SH-SY5Y** human neuroblastoma, **SK-OV-3** human ovary adenocarcinoma, **CDK1** protein kinase, **CD45** protein tyrosine phosphatase, **PP1** protein serine/threonine phosphatase, **PKCδ** protein kinase C, **PP2A** protein serine/threonine phosphatase, **PTP-S2** protein tyrosine phosphatase, **TCPTP** protein tyrosine phosphatase, **VHR** protein tyrosine phosphatase, **D10 clone** *Plasmodium falciparum* chloroquine-sensitive, **VRE** Vancomycin-resistant enterococci); **2. Geography** (**PRC** People's Republic China).

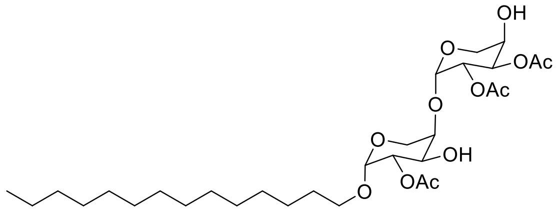

(+)-746

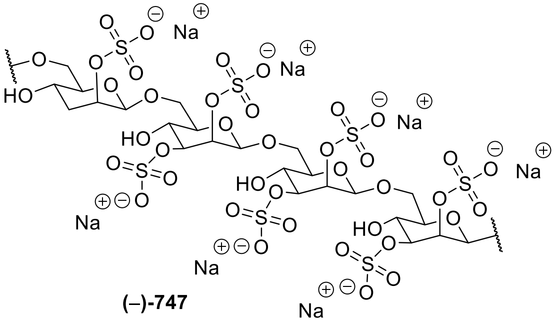

(–)-747

Figure S22: Structures of carbohydrates from Indonesian waters found in 1970–2017.

Table S22: Marine carbohydrates from Indonesian waters found in 1970–2017.

Compound	Structure Elucidation	Chemistry Type	Drug Class	Biological Activity: Cell/Enzyme/Micro-organism/Insect/Others	Biological Activity: Activity	Source of Organism	Province	Ref
(+)-Sinularioside **746**[β] [$C_{30}H_{52}O_{12}$]	MS, NMR, $[\alpha]_D$, CT	Carbohydrate	Anti-inflammatory	JJI (LPS/ NO_2^-)	58% (30 μM)	*Sinularia* sp.	NSW	[88]
(−)-Kakelokelose **747**[β]	IR, MS, NMR, CT	Carbohydrate	Antiviral	HIV-1	100% (0.3 μg/mL)	*D. molle*	NSW	[488]

Footnote: 1. **Activity** (**JJI** murine macrophage, **LPS** lipopolysaccharide).
